# Supplementary figures and images for: miR-138-5p ameliorates intestinal barrier disruption caused by acute superior mesenteric vein thrombosis injury by inhibiting the NLRP3/HMGB1 axis (part 1 of 2)
Source: PeerJ. 2024 Feb 21;12:e16692. doi: 10.7717/peerj.16692 (PMC10893868; doi:10.7717/peerj.16692)

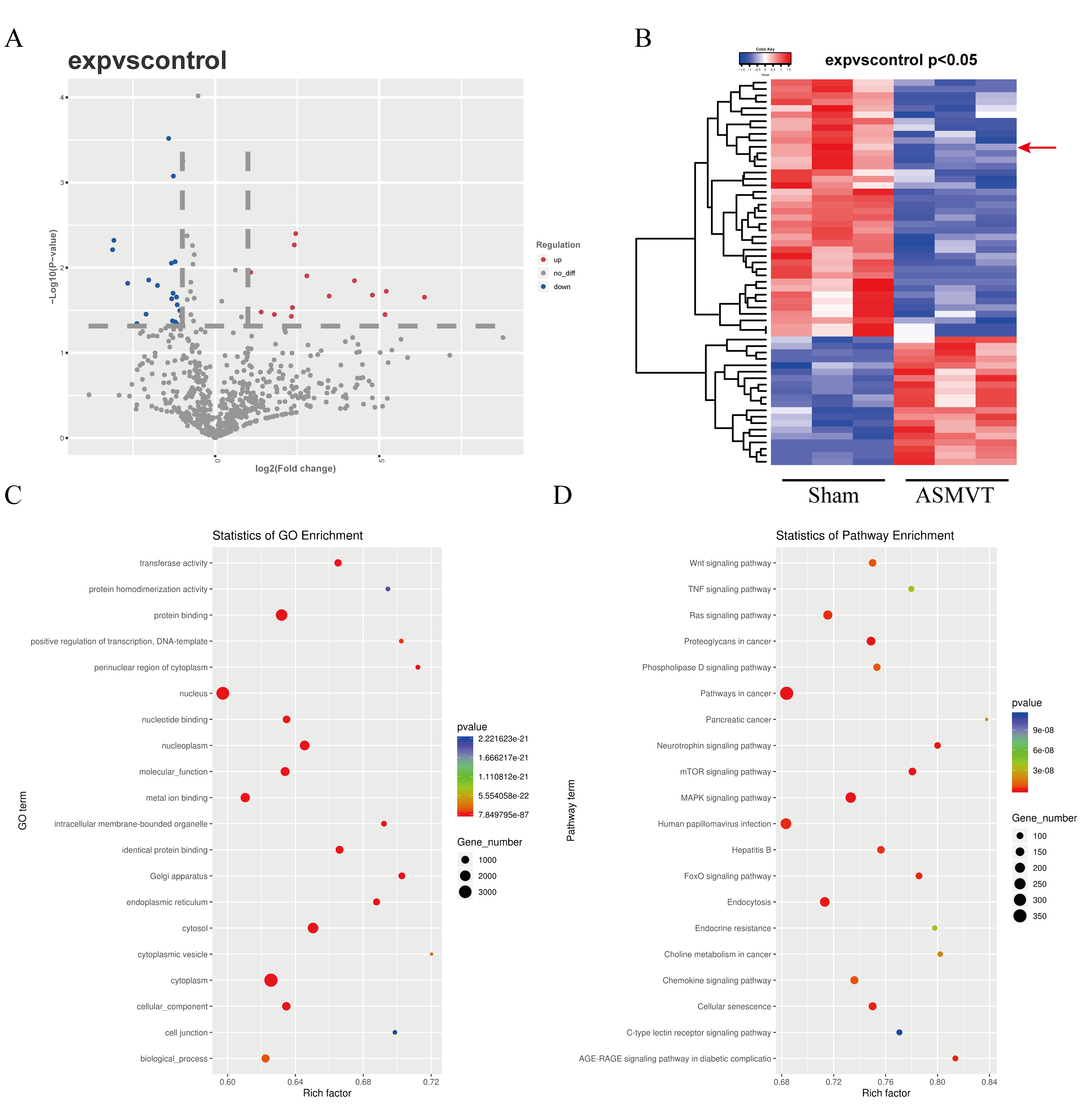

Supplement: Figure S1 — (A) Volcano plot; (B) Heatmap; (C) Gene Ontology; (D) KEGG pathway. miRNAs, microRNAs; RNA-Seq, transcriptome sequencing; KEGG, Kyoto Encyclopedia of Genes and Genomes. [file peerj-12-16692-s002.png]

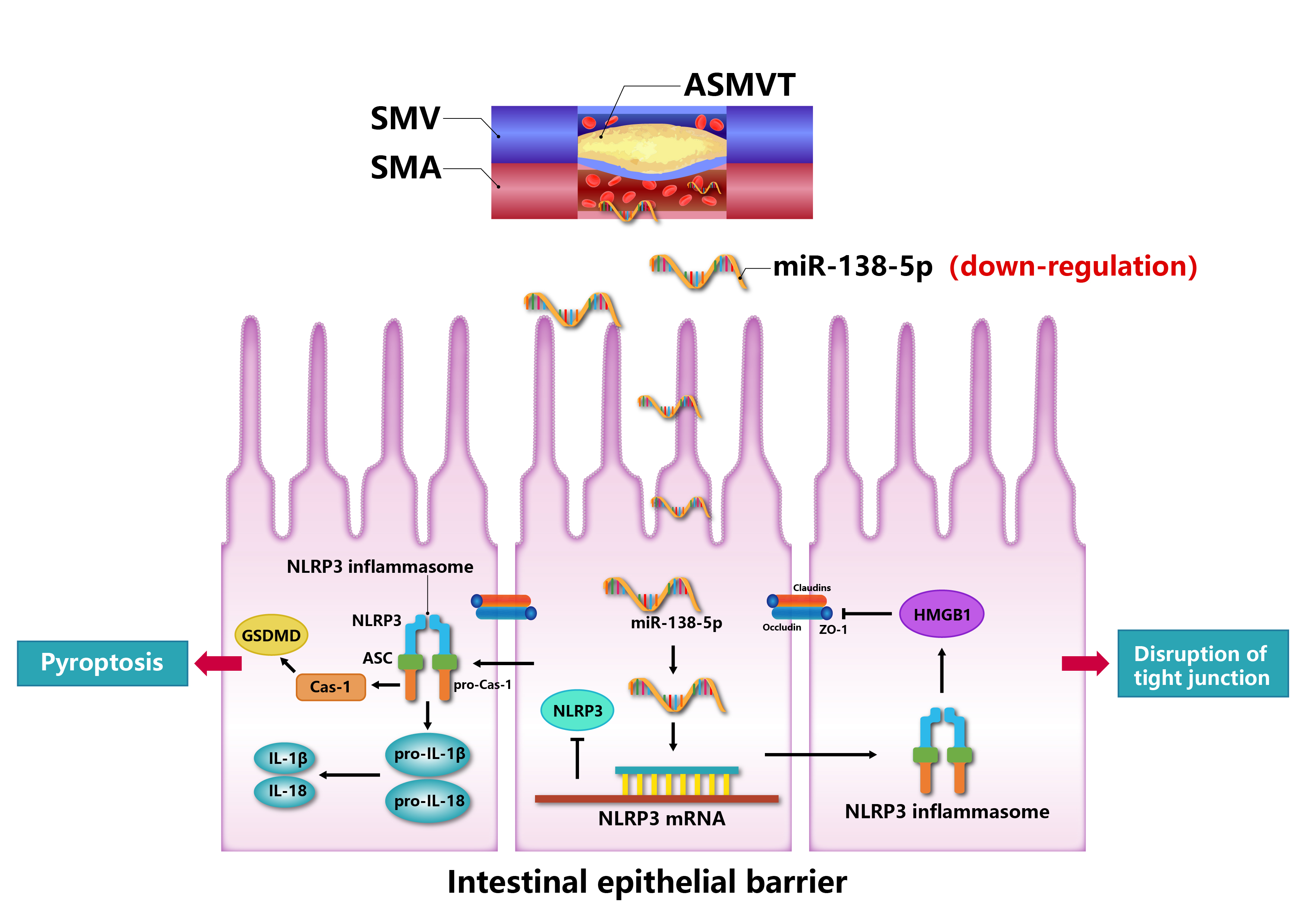

Supplement: Supplemental Information 3 [file peerj-12-16692-s003.png]

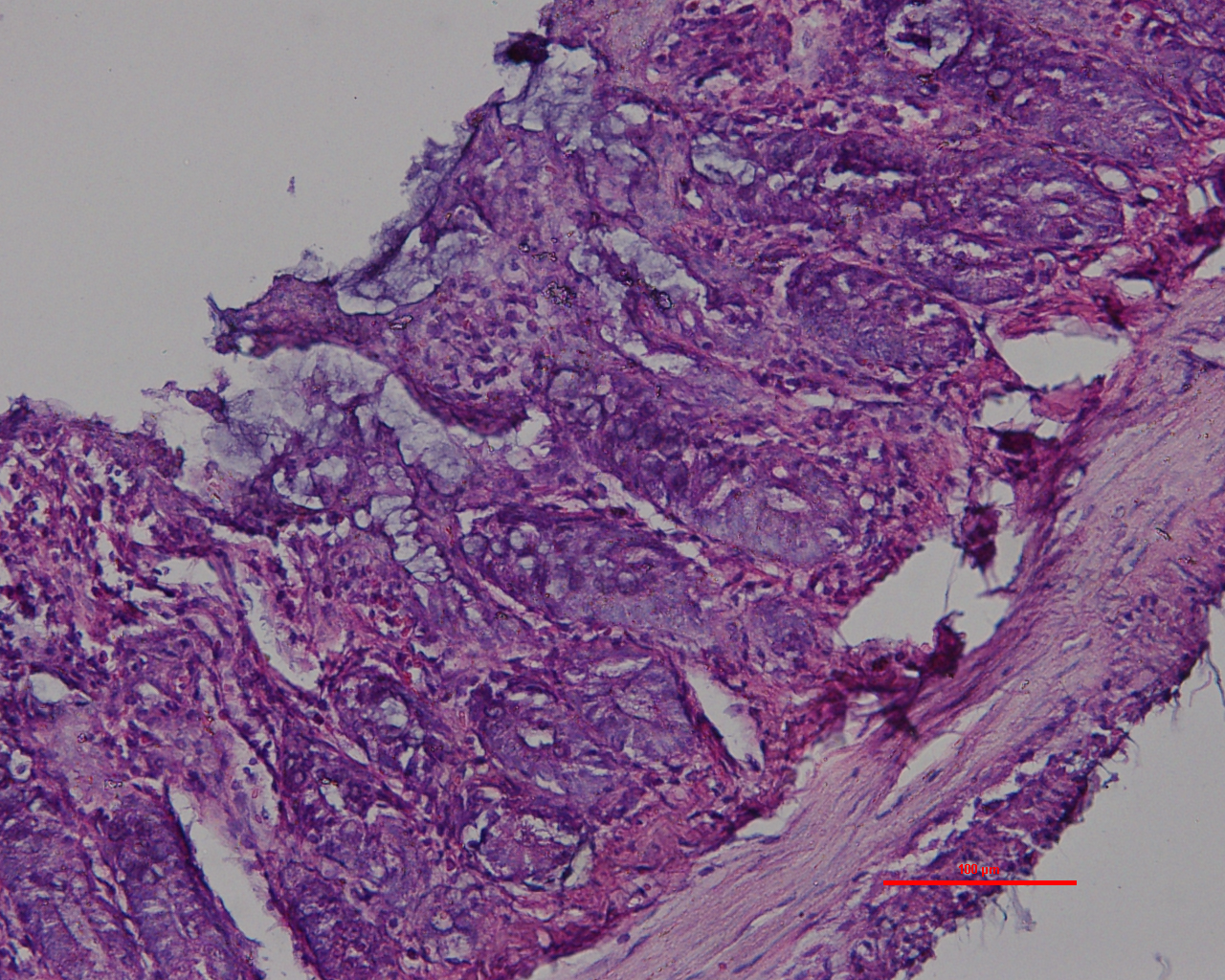

Supplement: Supplemental Information 5 [file peerj-12-16692-s005.zip › original data-figure 1/1A/ASMVT.tif]

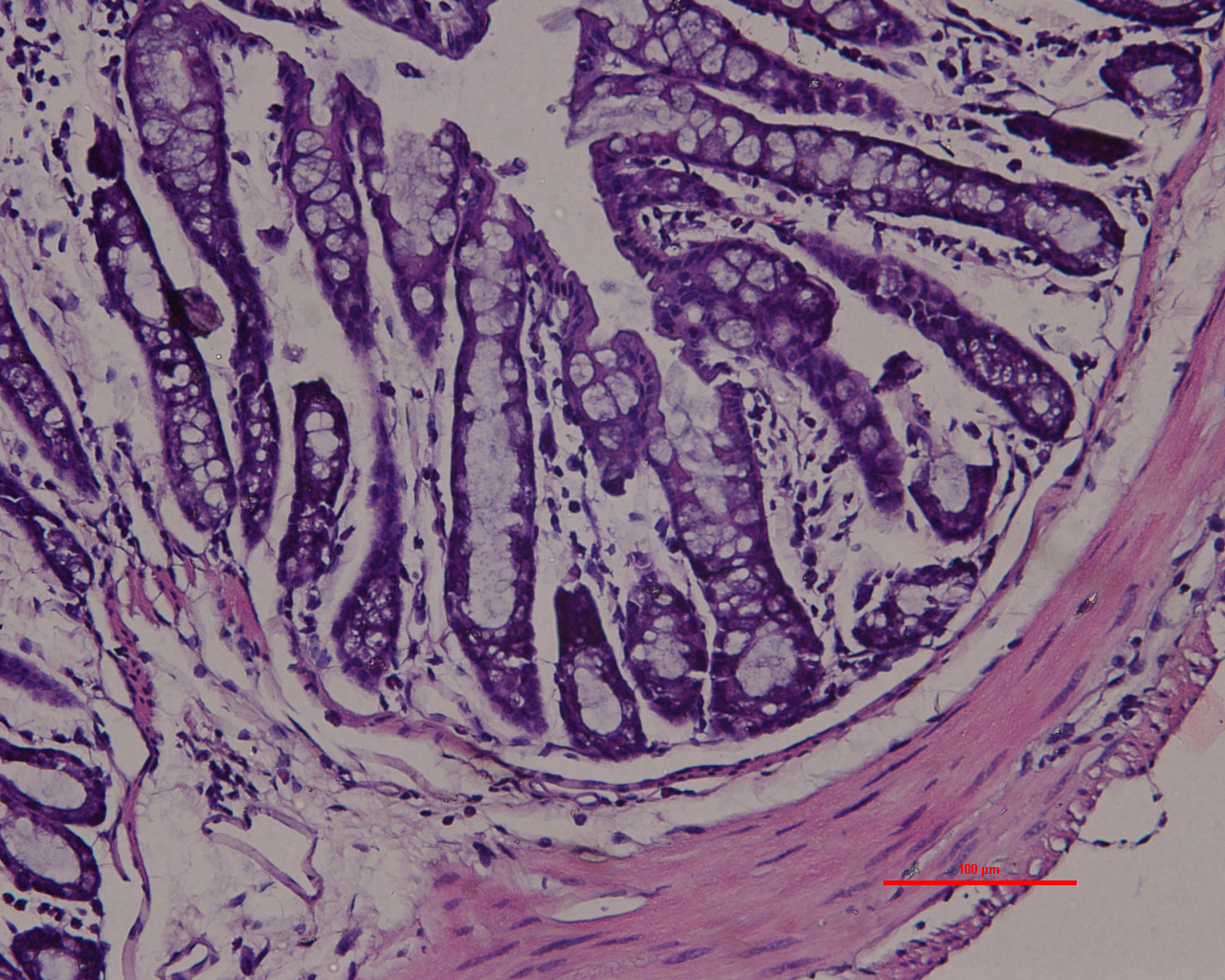

Supplement: Supplemental Information 5 [file peerj-12-16692-s005.zip › original data-figure 1/1A/Sham.tif]

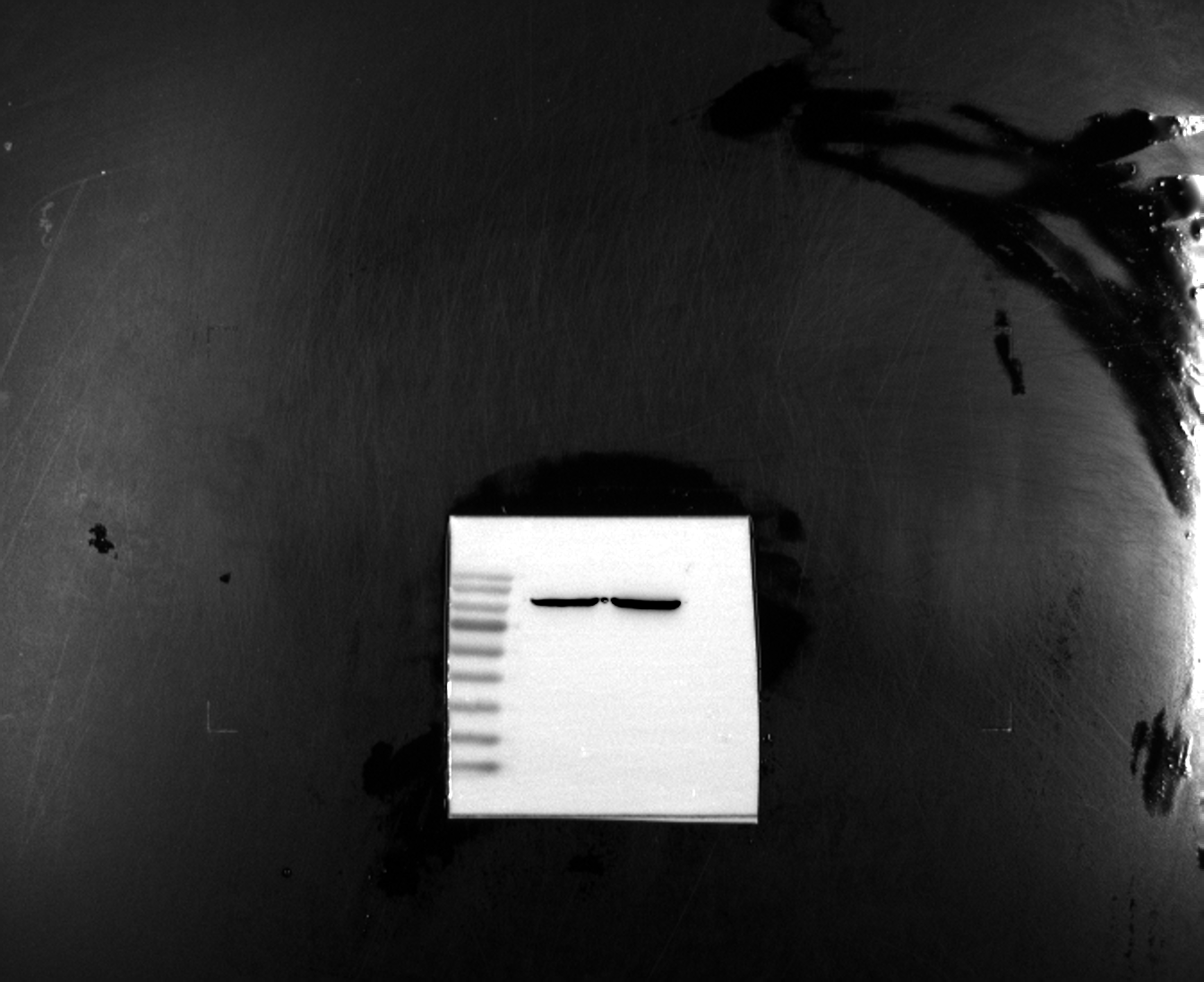

Supplement: Supplemental Information 5 [file peerj-12-16692-s005.zip › original data-figure 1/1B/1.NLRP3.tif]

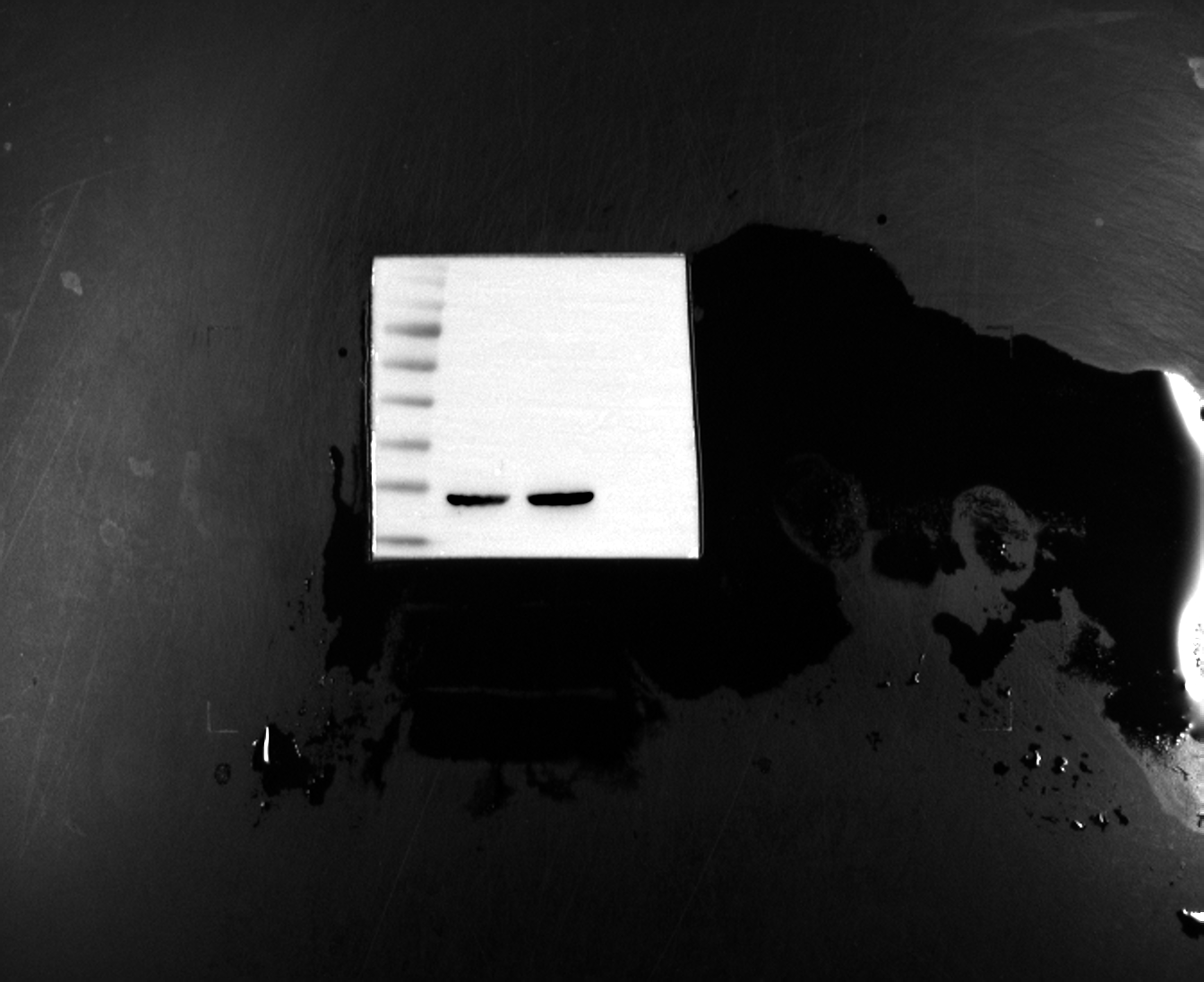

Supplement: Supplemental Information 5 [file peerj-12-16692-s005.zip › original data-figure 1/1B/2.ASC.tif]

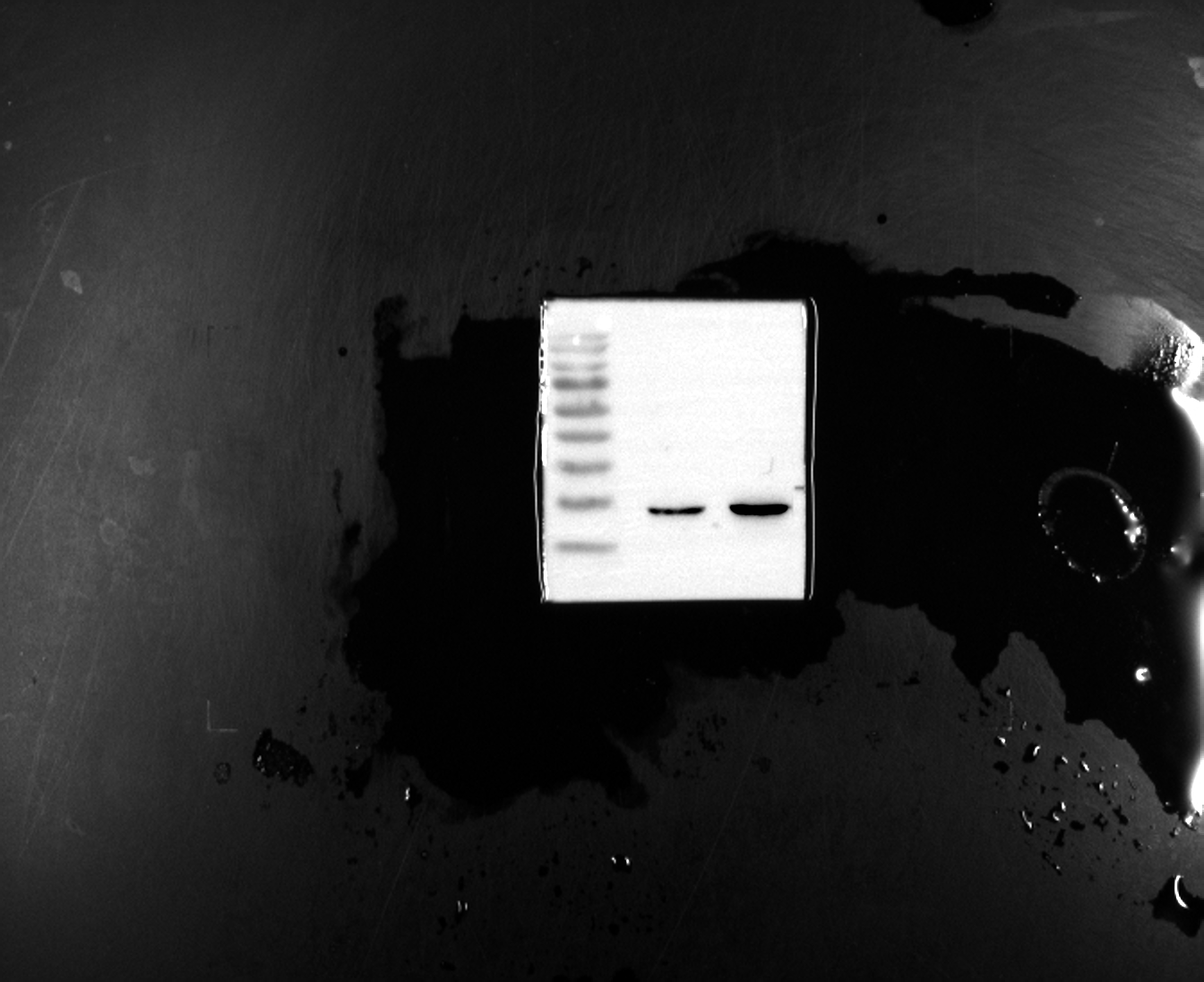

Supplement: Supplemental Information 5 [file peerj-12-16692-s005.zip › original data-figure 1/1B/3.Caspase 1.tif]

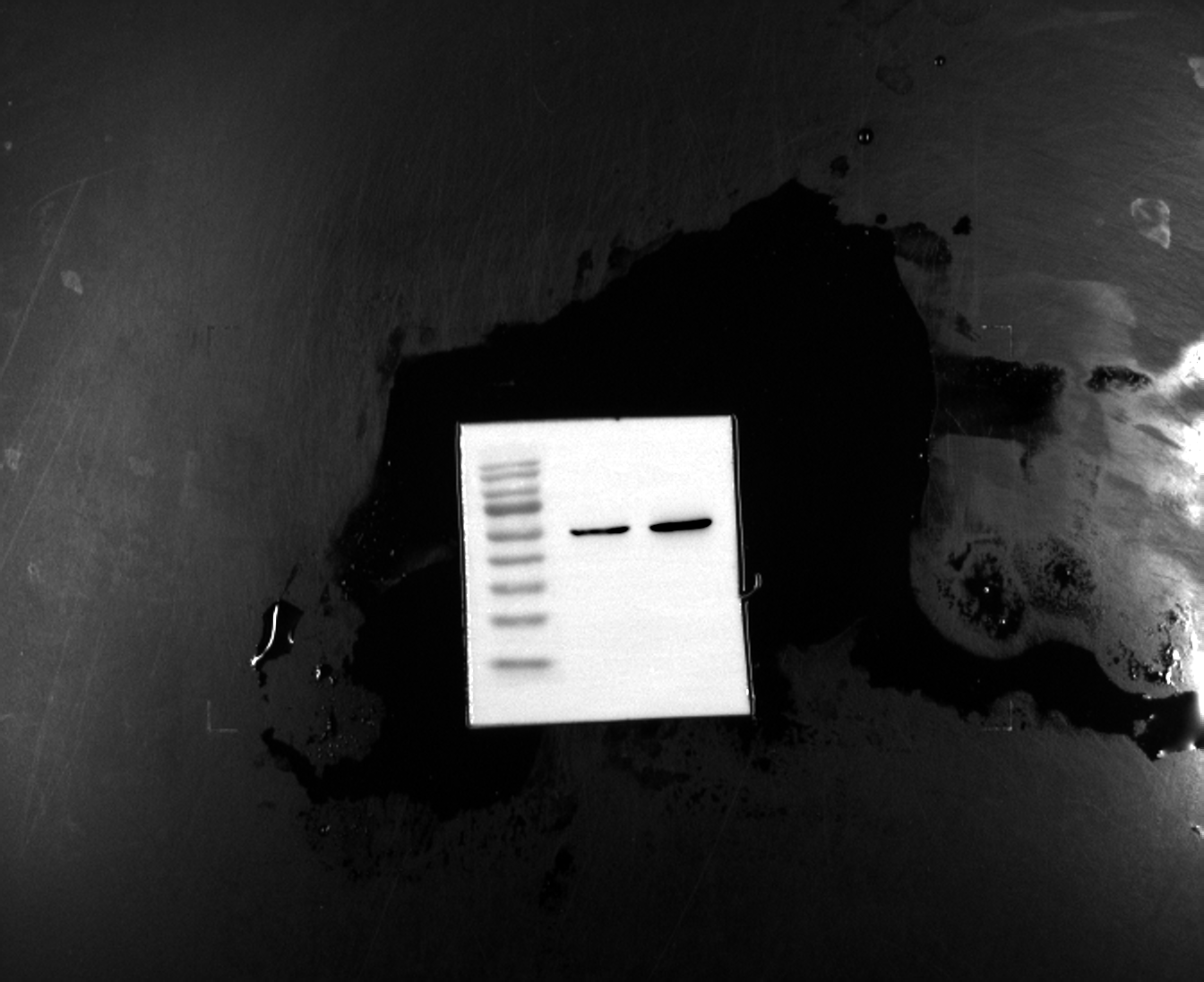

Supplement: Supplemental Information 5 [file peerj-12-16692-s005.zip › original data-figure 1/1B/4.GSDMD-N.tif]

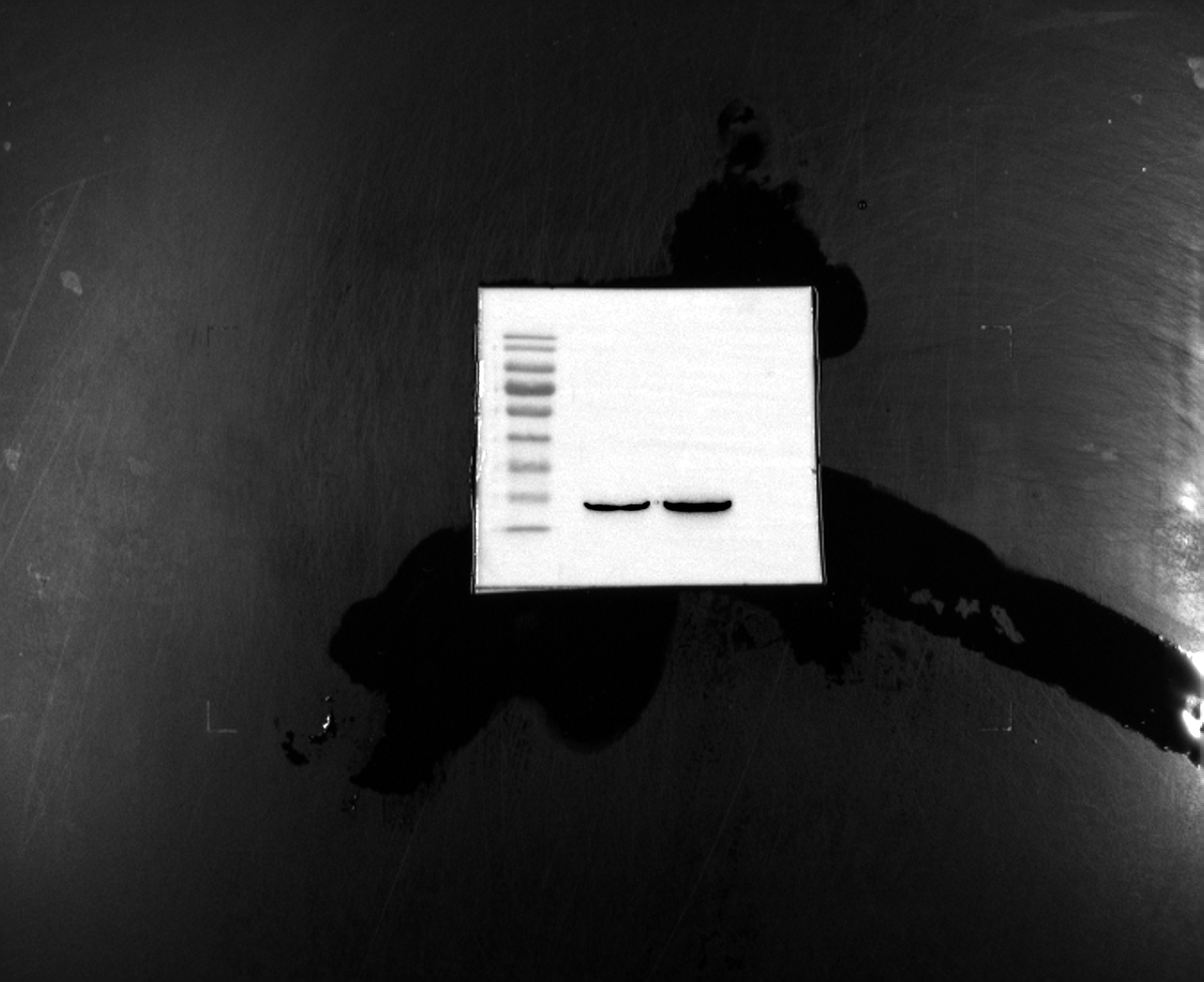

Supplement: Supplemental Information 5 [file peerj-12-16692-s005.zip › original data-figure 1/1B/5.IL-18.tif]

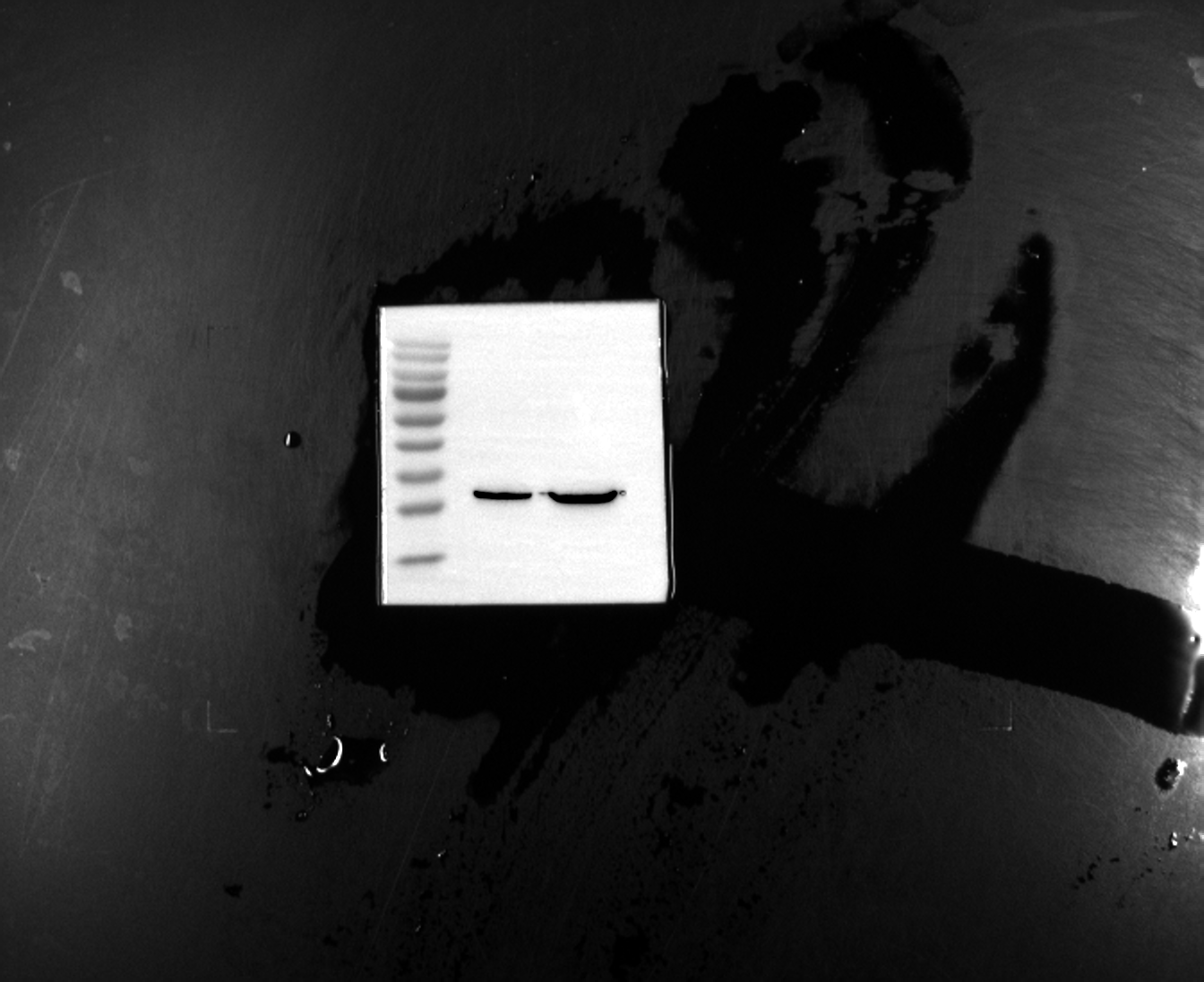

Supplement: Supplemental Information 5 [file peerj-12-16692-s005.zip › original data-figure 1/1B/6.IL-1β.tif]

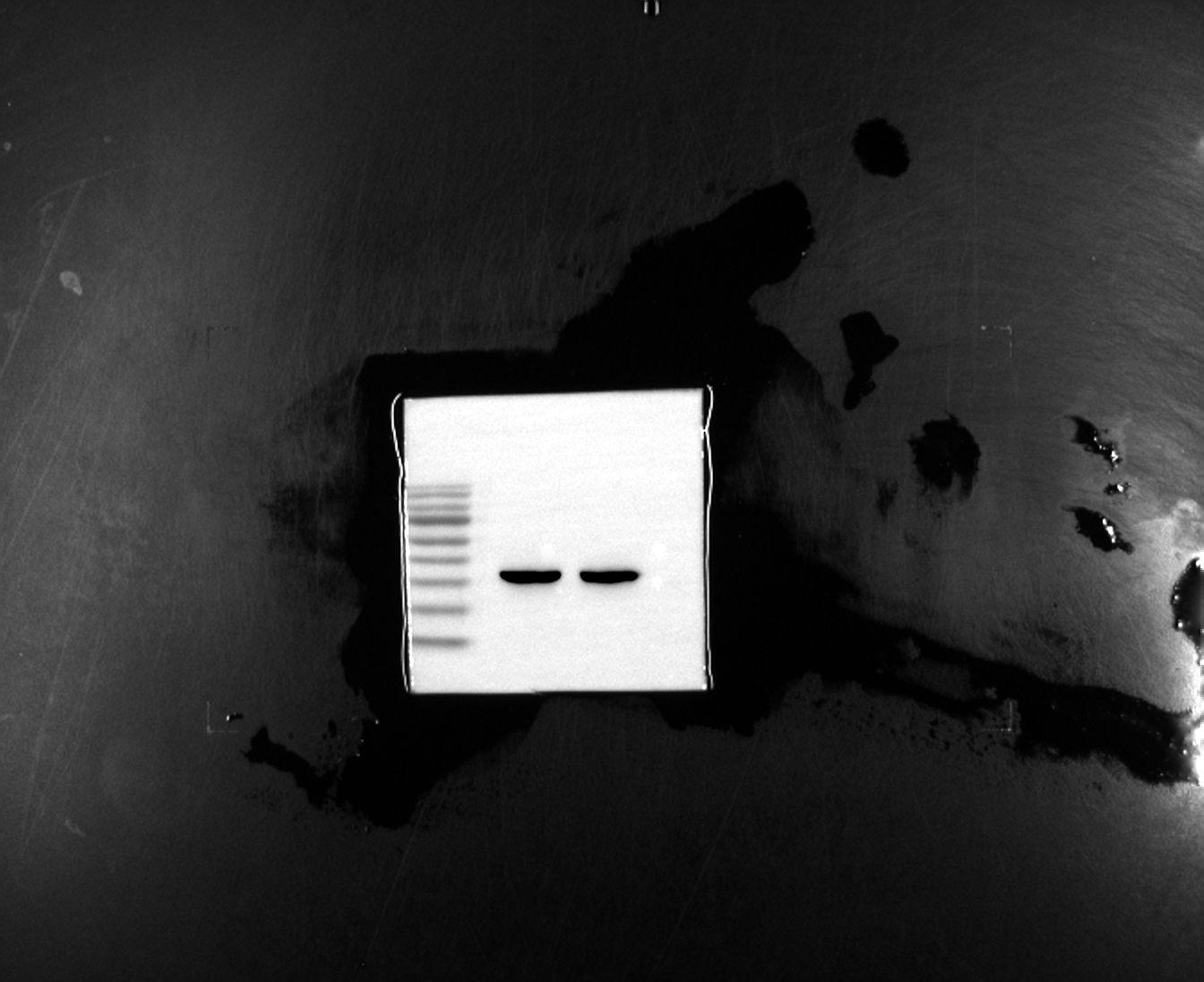

Supplement: Supplemental Information 5 [file peerj-12-16692-s005.zip › original data-figure 1/1B/7.GAPDH.tif]

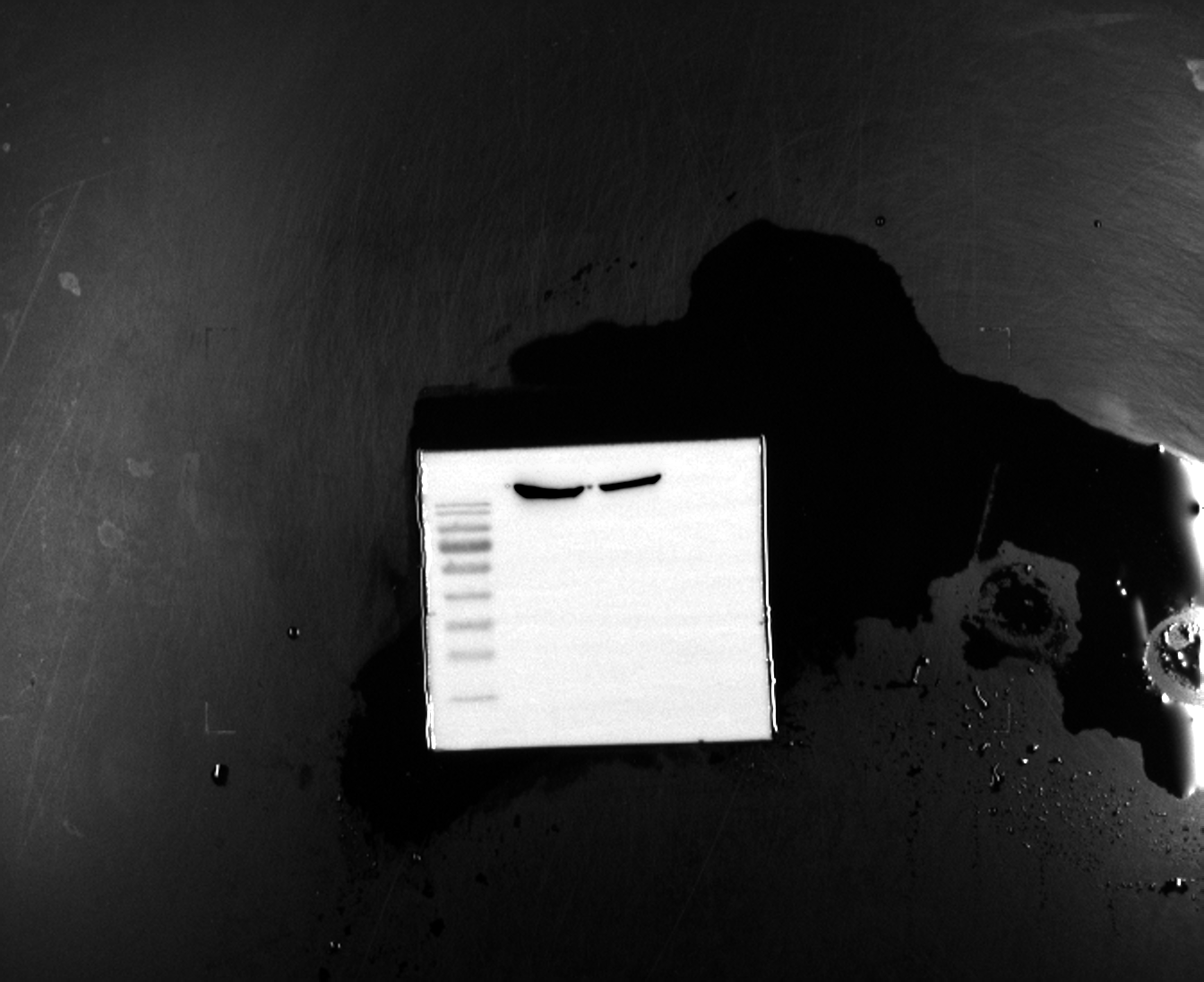

Supplement: Supplemental Information 5 [file peerj-12-16692-s005.zip › original data-figure 1/1C/1.ZO-1.tif]

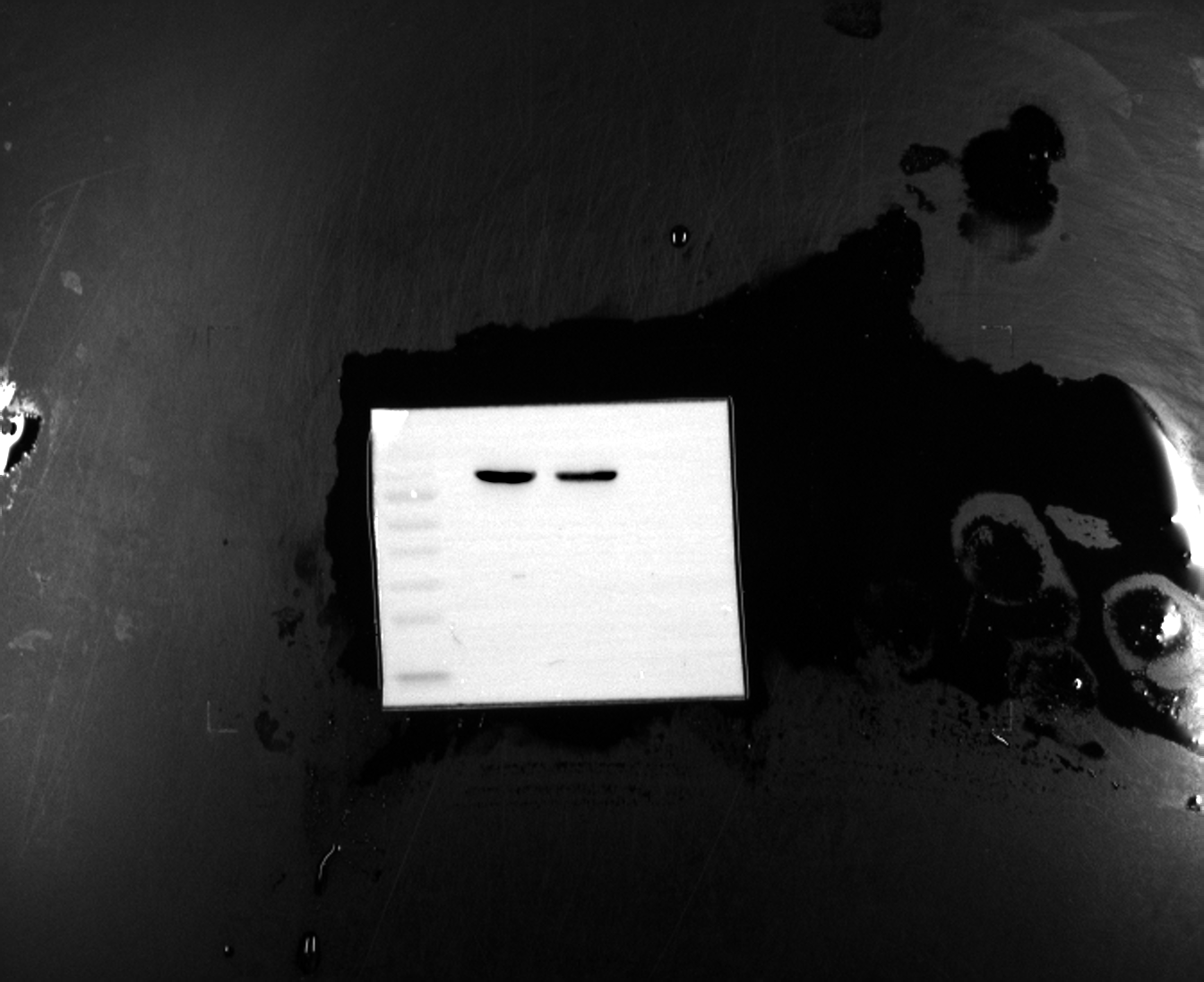

Supplement: Supplemental Information 5 [file peerj-12-16692-s005.zip › original data-figure 1/1C/2.Occludin.tif]

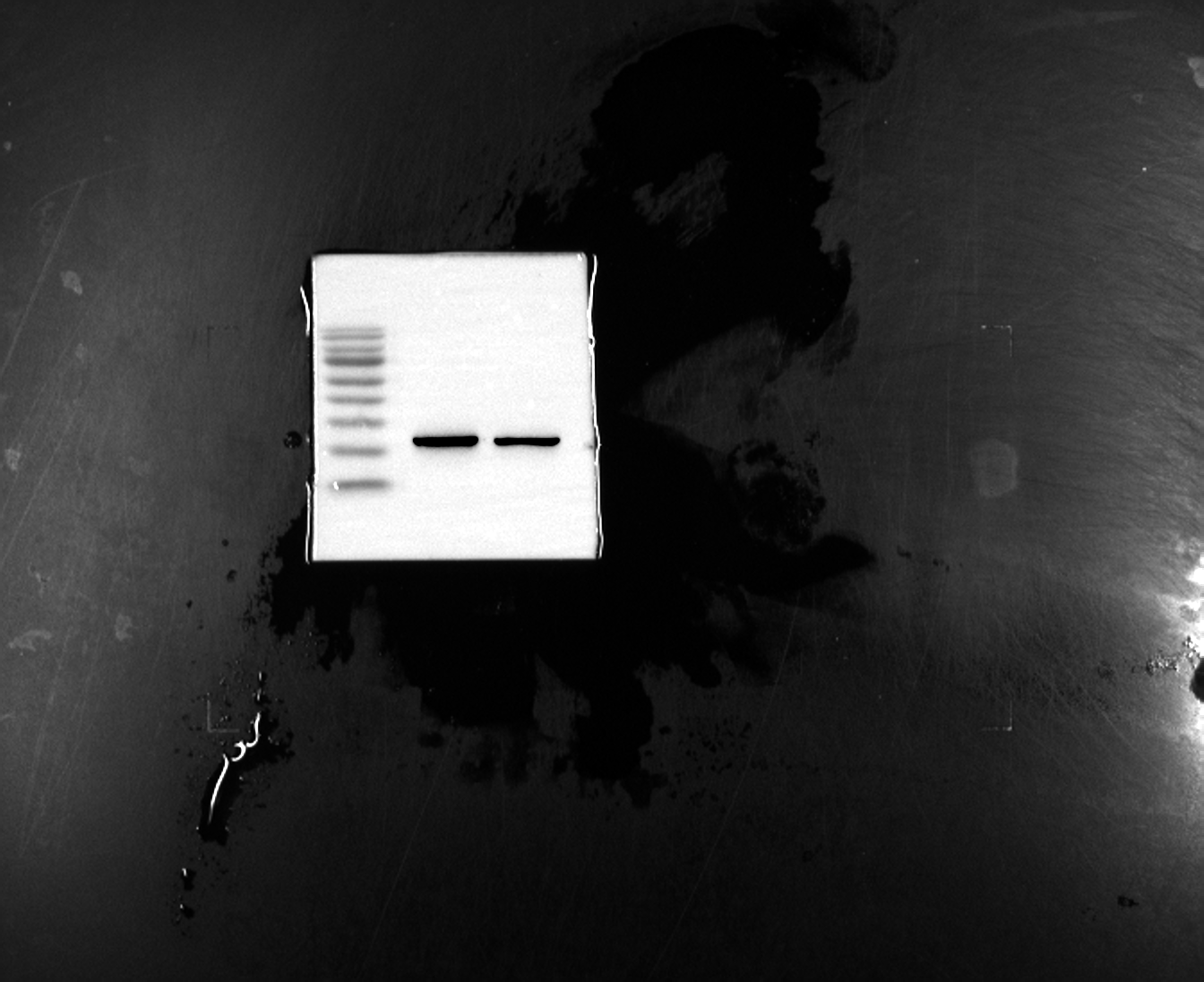

Supplement: Supplemental Information 5 [file peerj-12-16692-s005.zip › original data-figure 1/1C/3.Claudin-1.tif]

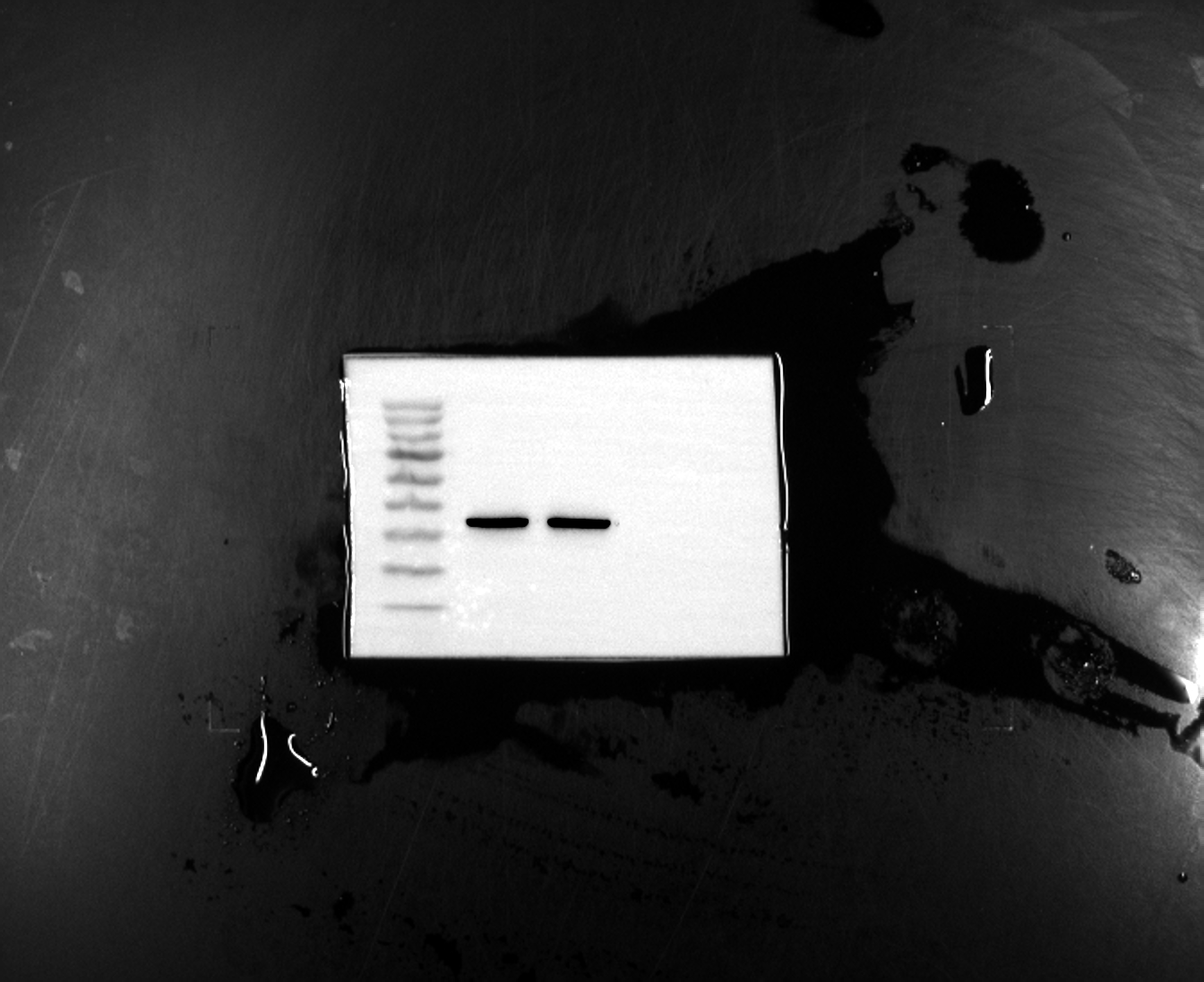

Supplement: Supplemental Information 5 [file peerj-12-16692-s005.zip › original data-figure 1/1C/4.GAPDH.tif]

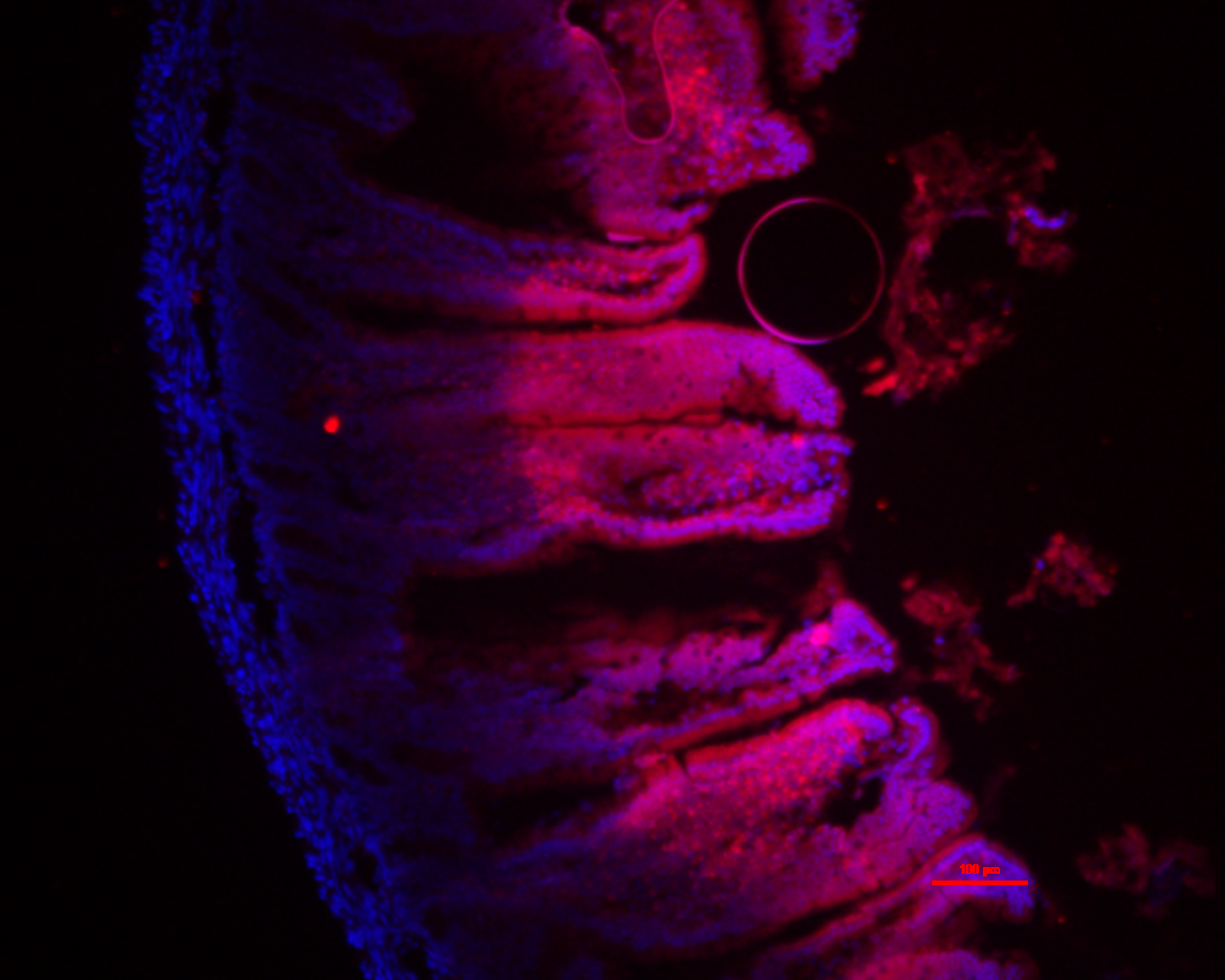

Supplement: Supplemental Information 5 [file peerj-12-16692-s005.zip › original data-figure 1/1D/1.Sham/1.ZO-1.tif]

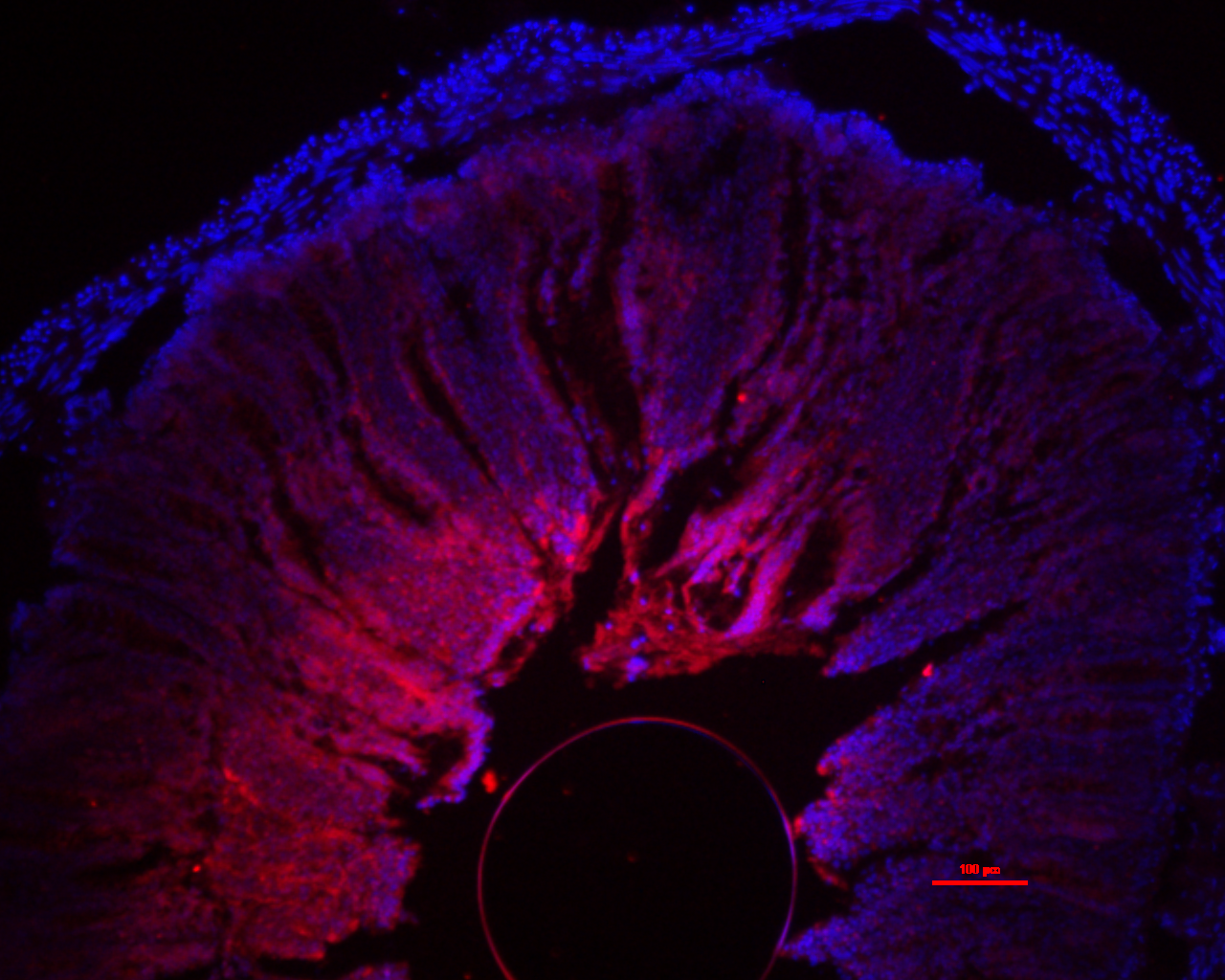

Supplement: Supplemental Information 5 [file peerj-12-16692-s005.zip › original data-figure 1/1D/1.Sham/2.Occludin.tif]

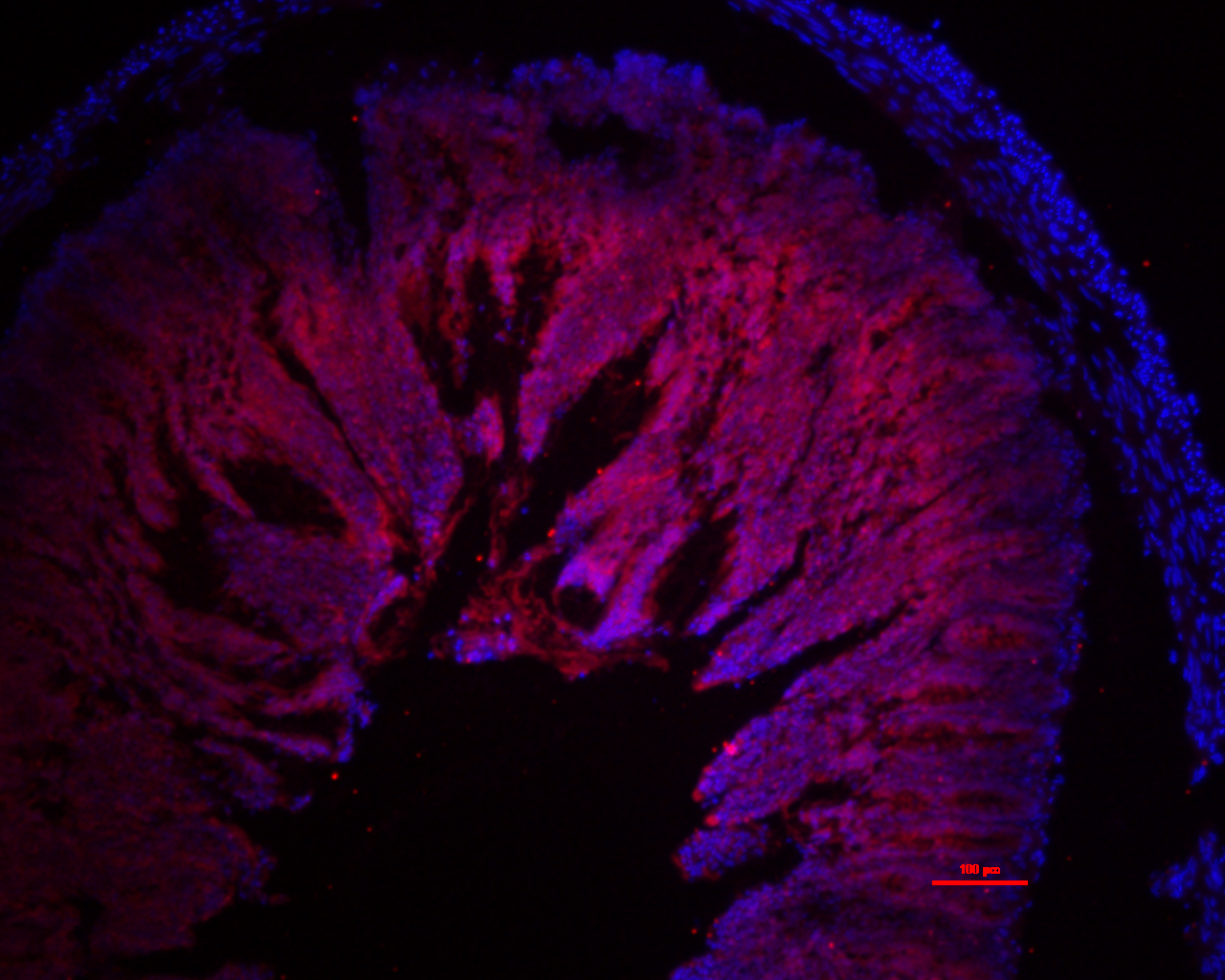

Supplement: Supplemental Information 5 [file peerj-12-16692-s005.zip › original data-figure 1/1D/1.Sham/3.Claudin-1.tif]

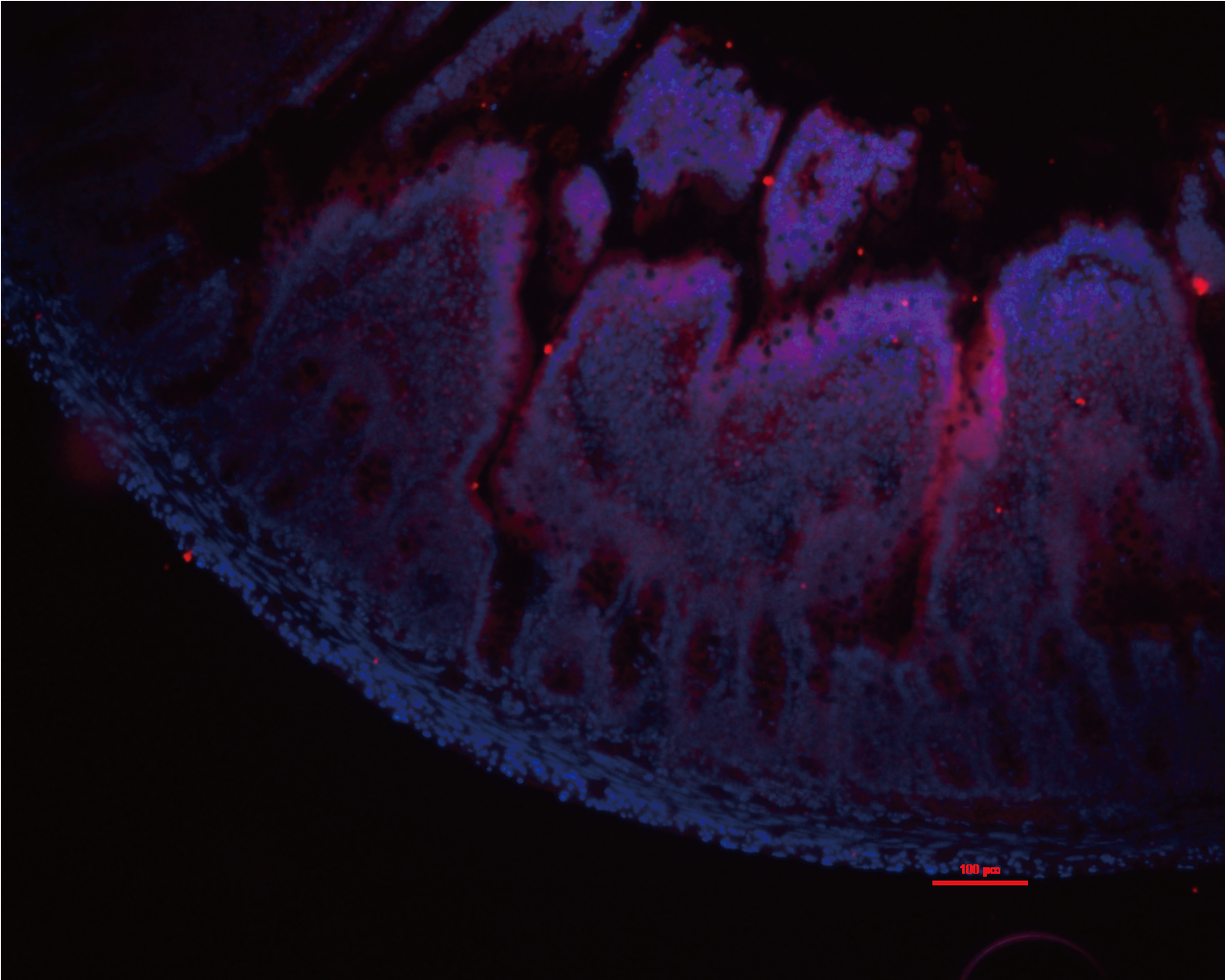

Supplement: Supplemental Information 5 [file peerj-12-16692-s005.zip › original data-figure 1/1D/2.ASMVT/1.ZO-1.png]

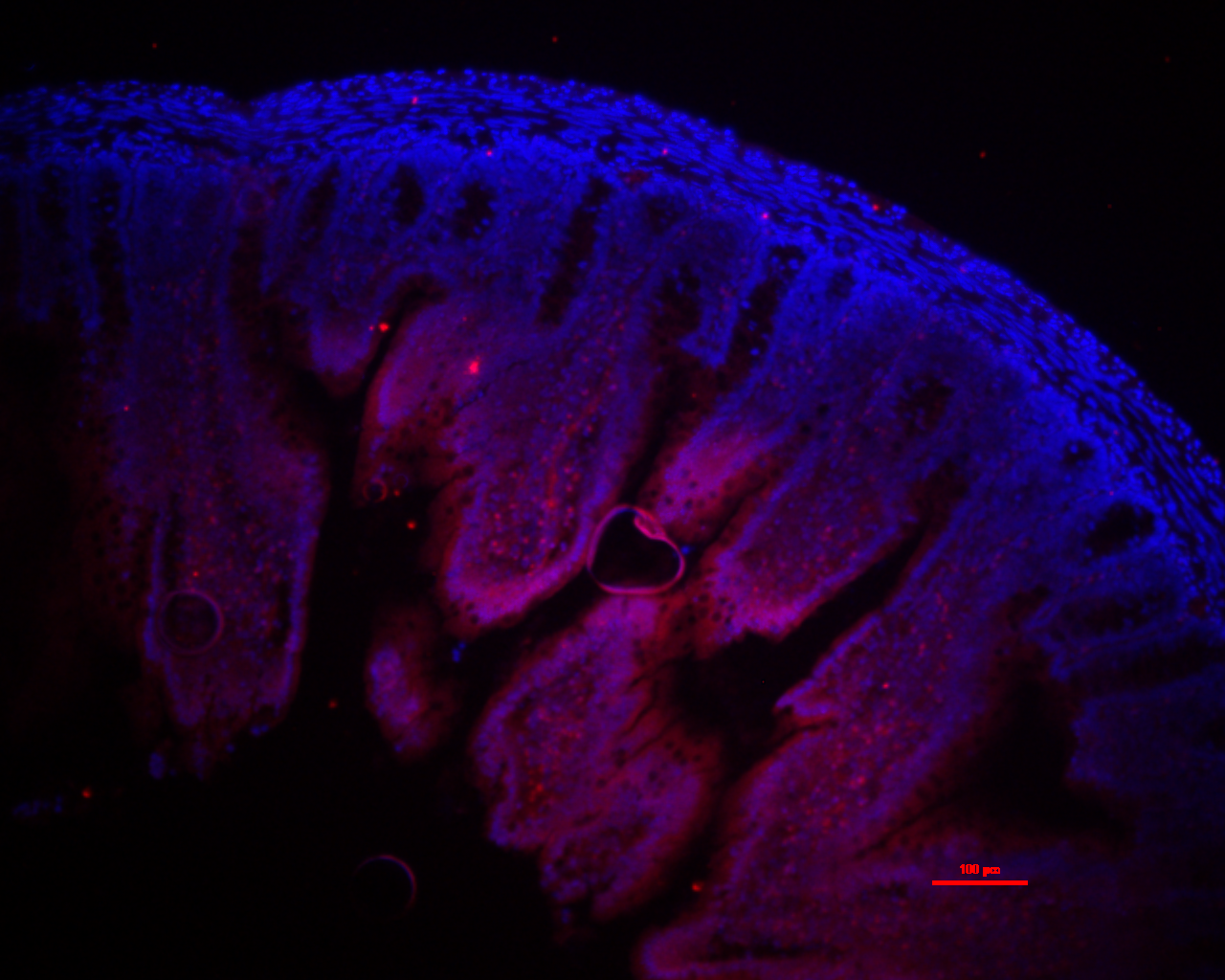

Supplement: Supplemental Information 5 [file peerj-12-16692-s005.zip › original data-figure 1/1D/2.ASMVT/2.Occludin.tif]

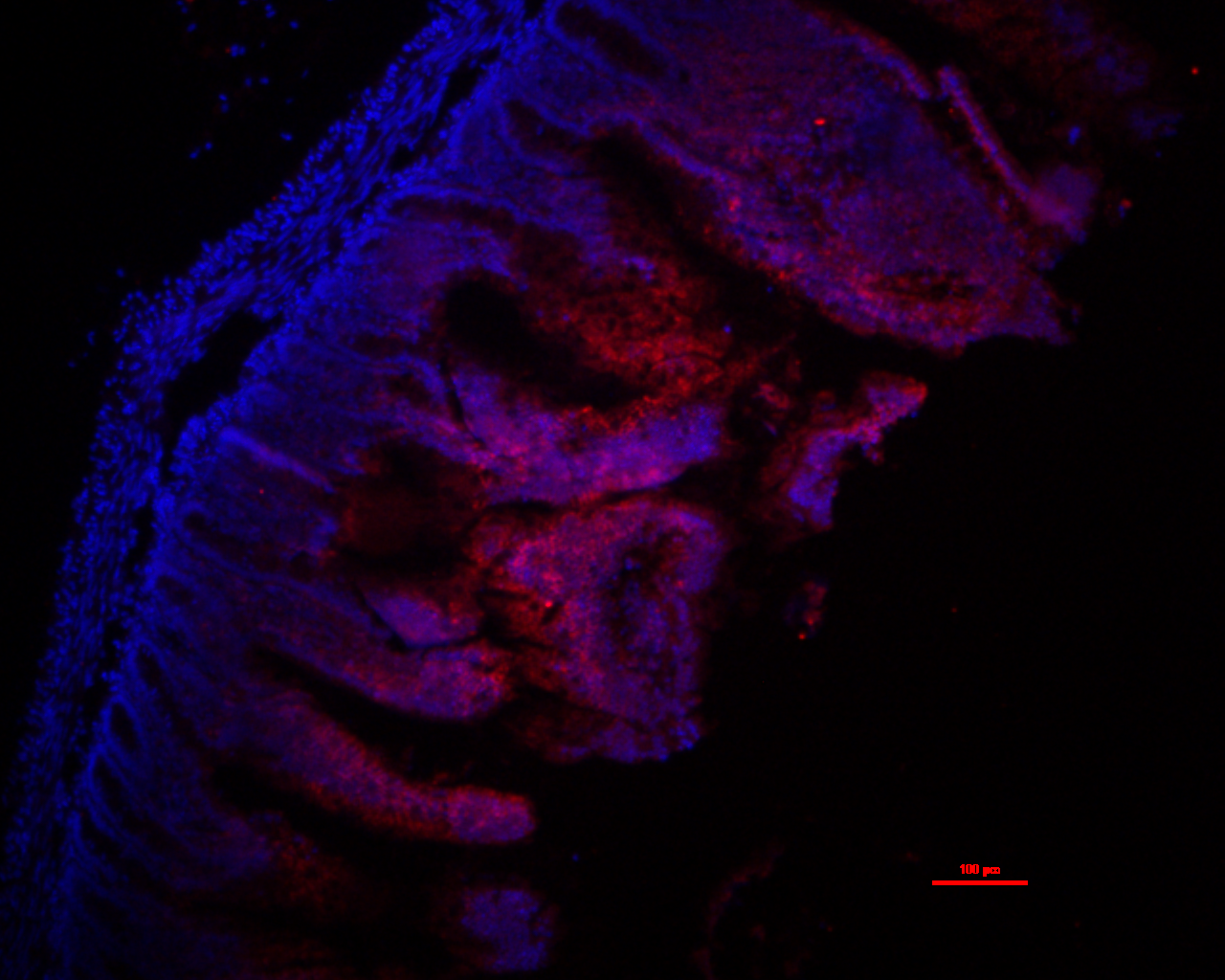

Supplement: Supplemental Information 5 [file peerj-12-16692-s005.zip › original data-figure 1/1D/2.ASMVT/3.Claudin-1.tif]

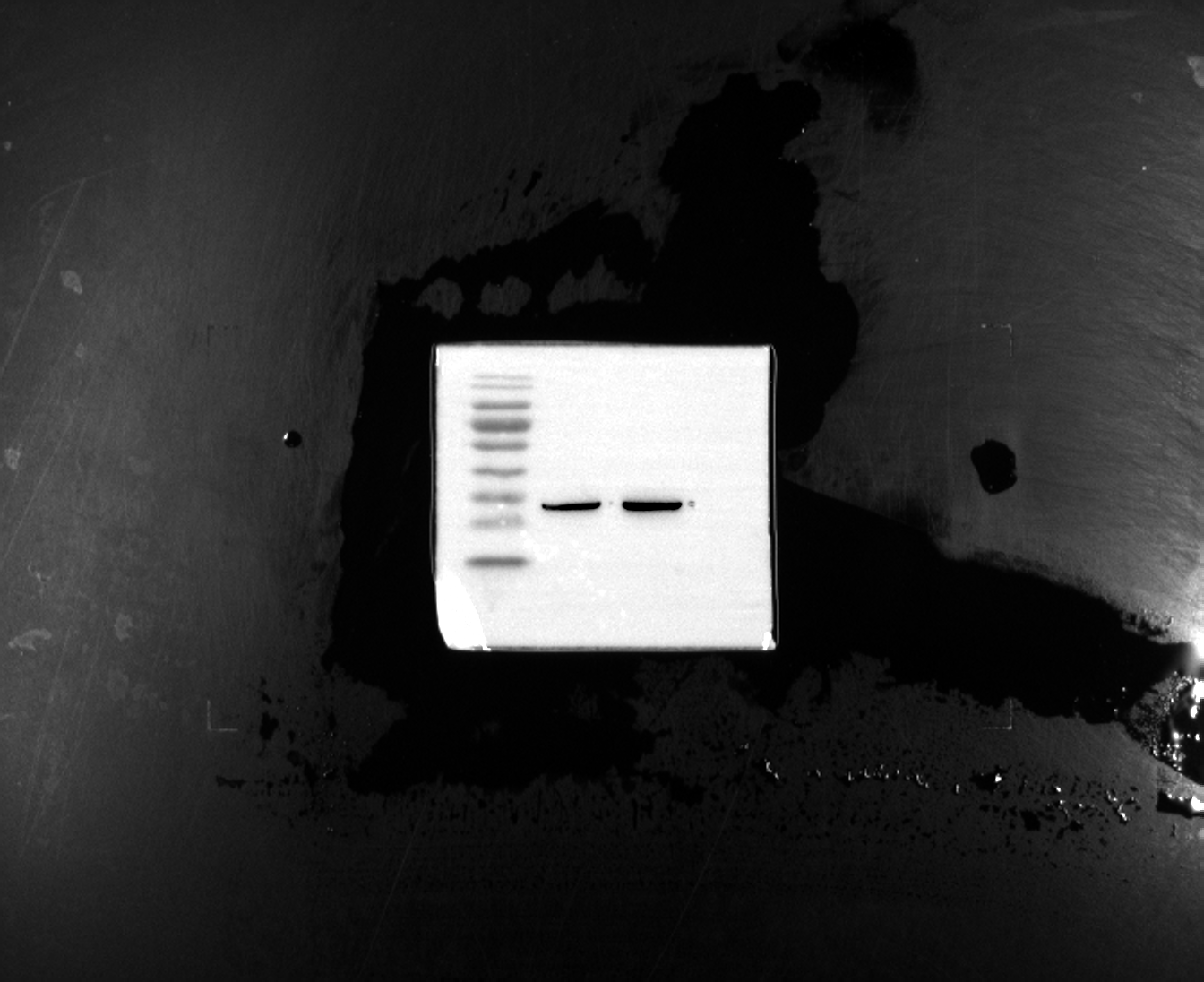

Supplement: Supplemental Information 5 [file peerj-12-16692-s005.zip › original data-figure 1/1E/1.HMGB1.tif]

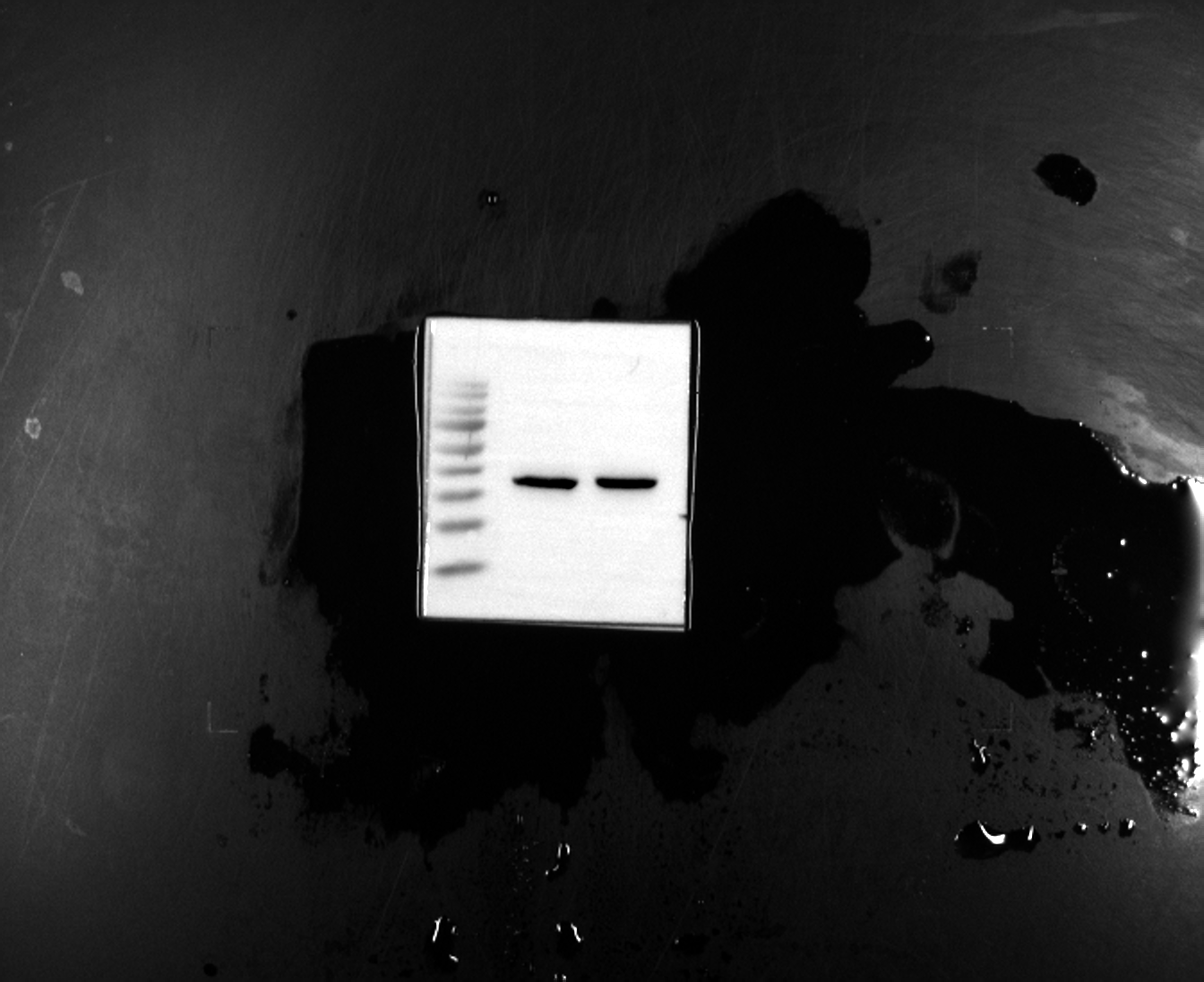

Supplement: Supplemental Information 5 [file peerj-12-16692-s005.zip › original data-figure 1/1E/2.GAPDH.tif]

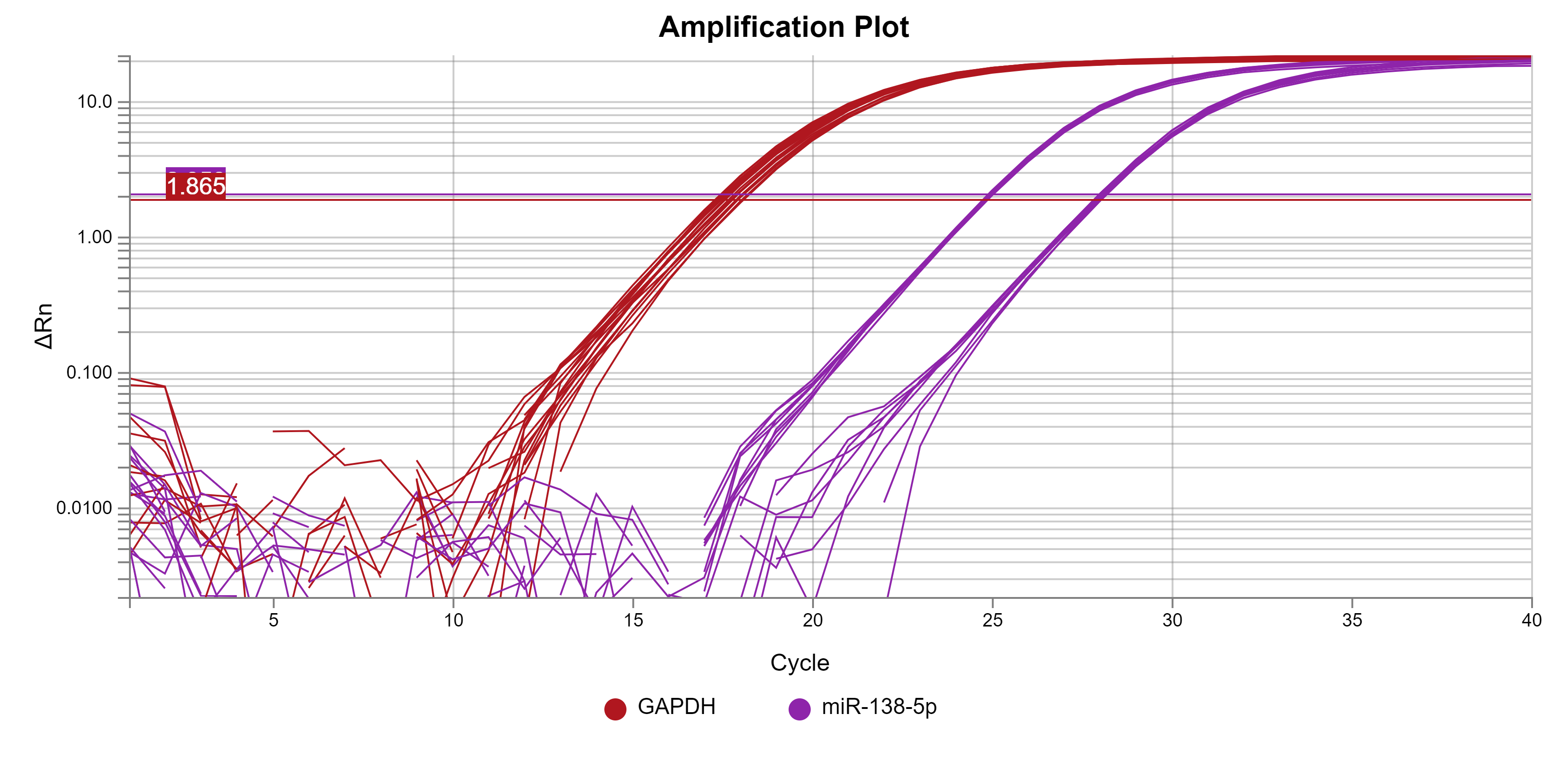

Supplement: Supplemental Information 6 [file peerj-12-16692-s006.zip › original data-figure 2/image/2B/Amplification Plot_2023-08-17-141942.png]

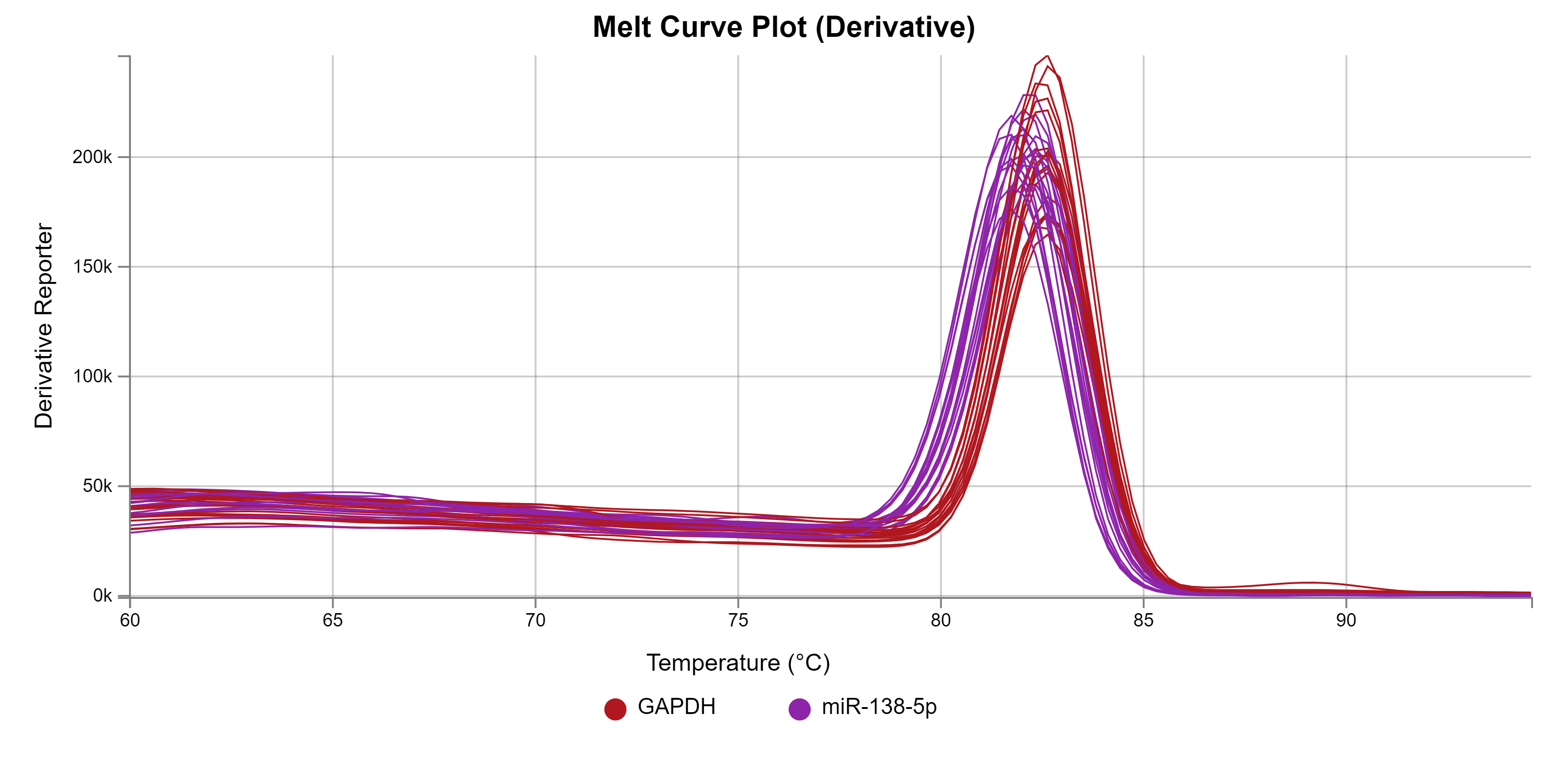

Supplement: Supplemental Information 6 [file peerj-12-16692-s006.zip › original data-figure 2/image/2B/Melt Curve Plot_2023-08-17-142010.png]

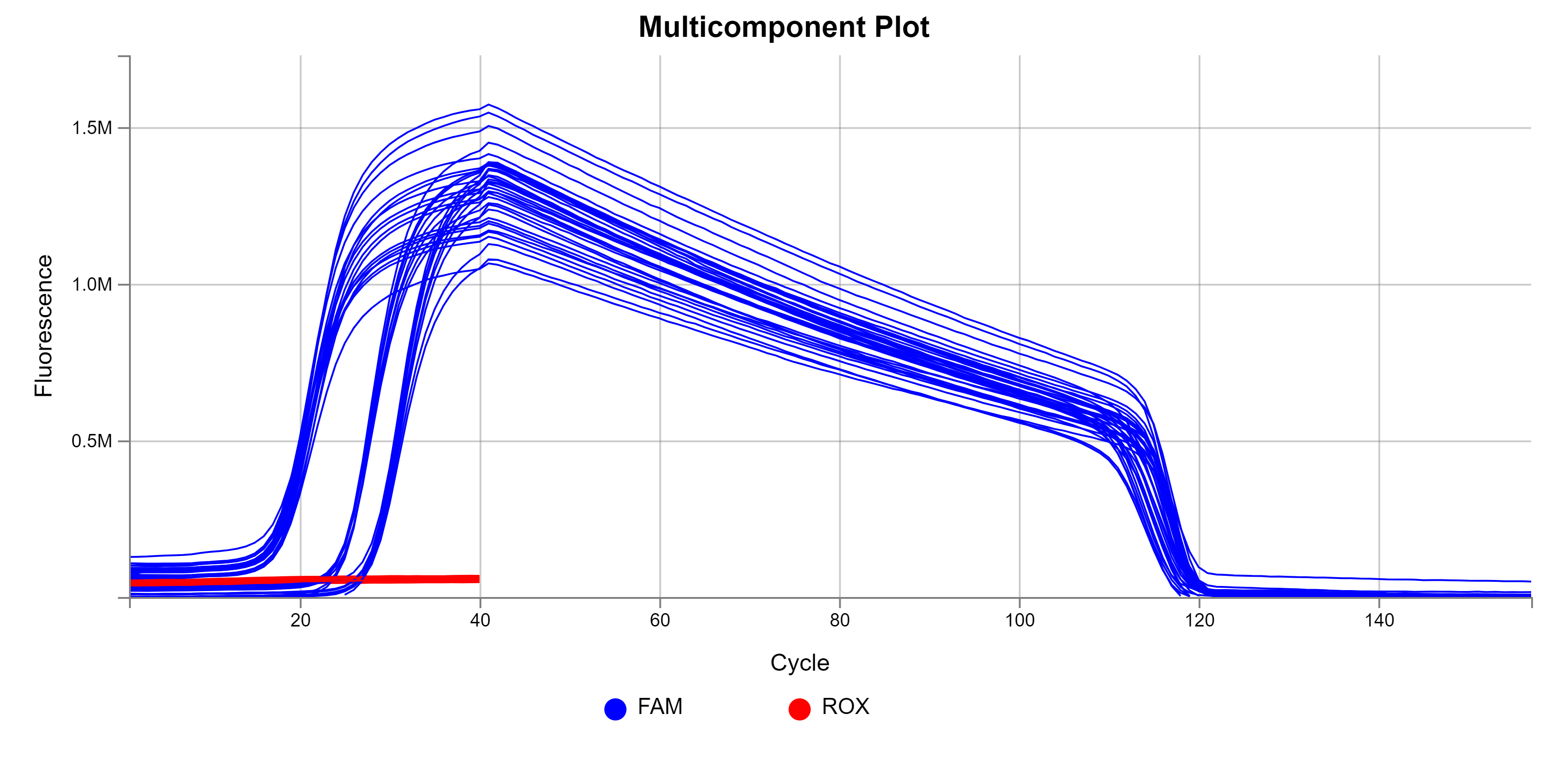

Supplement: Supplemental Information 6 [file peerj-12-16692-s006.zip › original data-figure 2/image/2B/Multicomponent Plot_2023-08-17-141956.png]

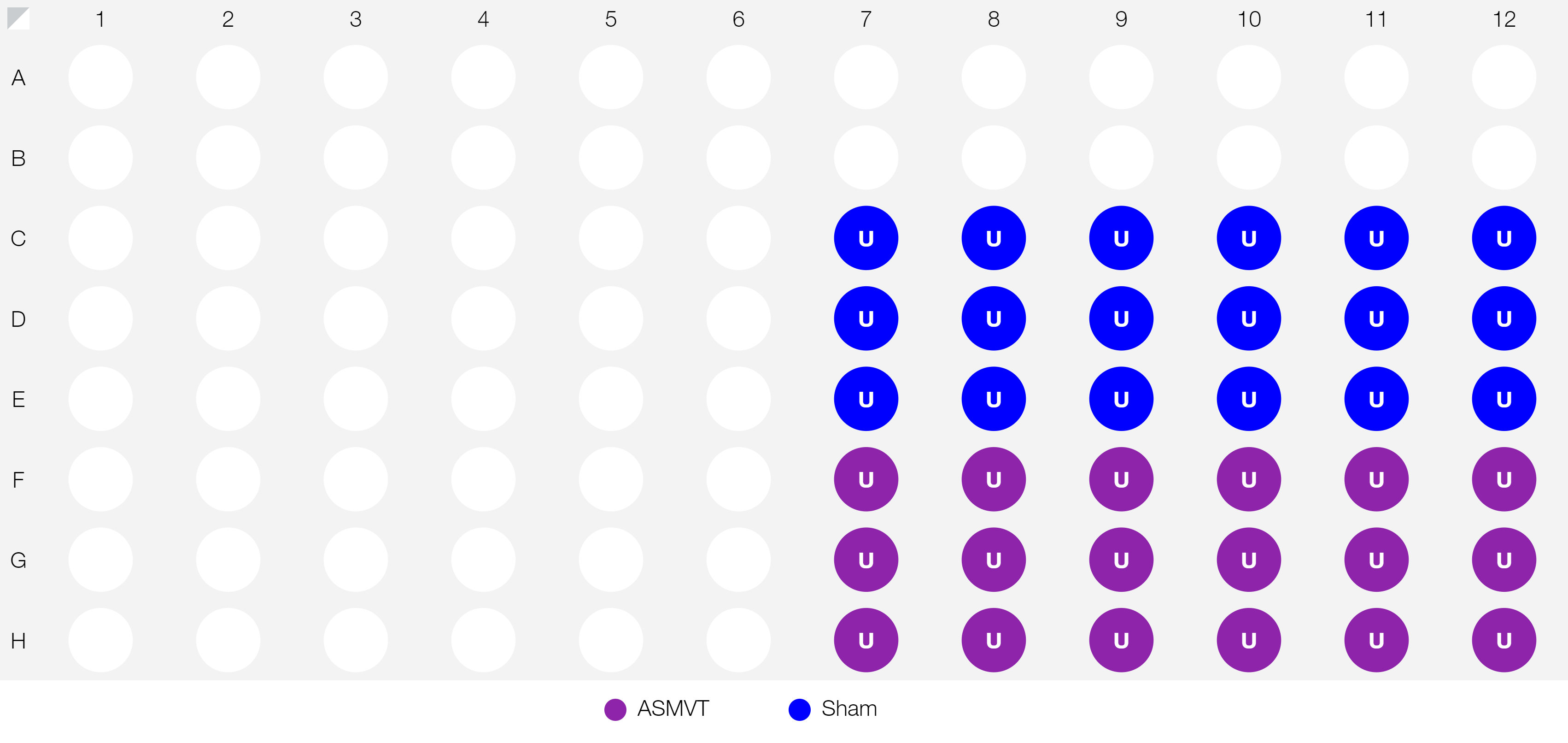

Supplement: Supplemental Information 6 [file peerj-12-16692-s006.zip › original data-figure 2/image/2B/Plate_2023-08-17-142018.png]

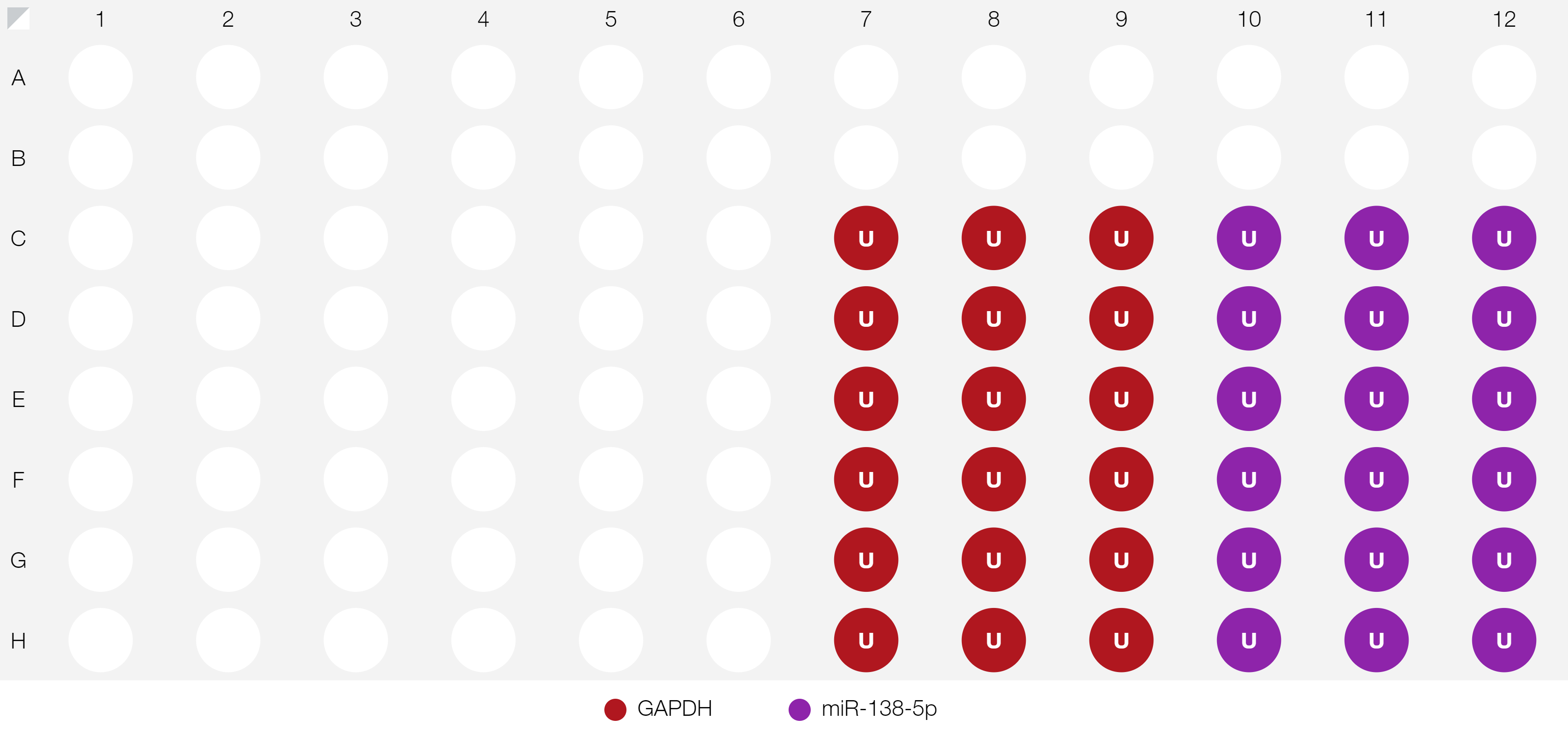

Supplement: Supplemental Information 6 [file peerj-12-16692-s006.zip › original data-figure 2/image/2B/Plate_2023-08-17-142035.png]

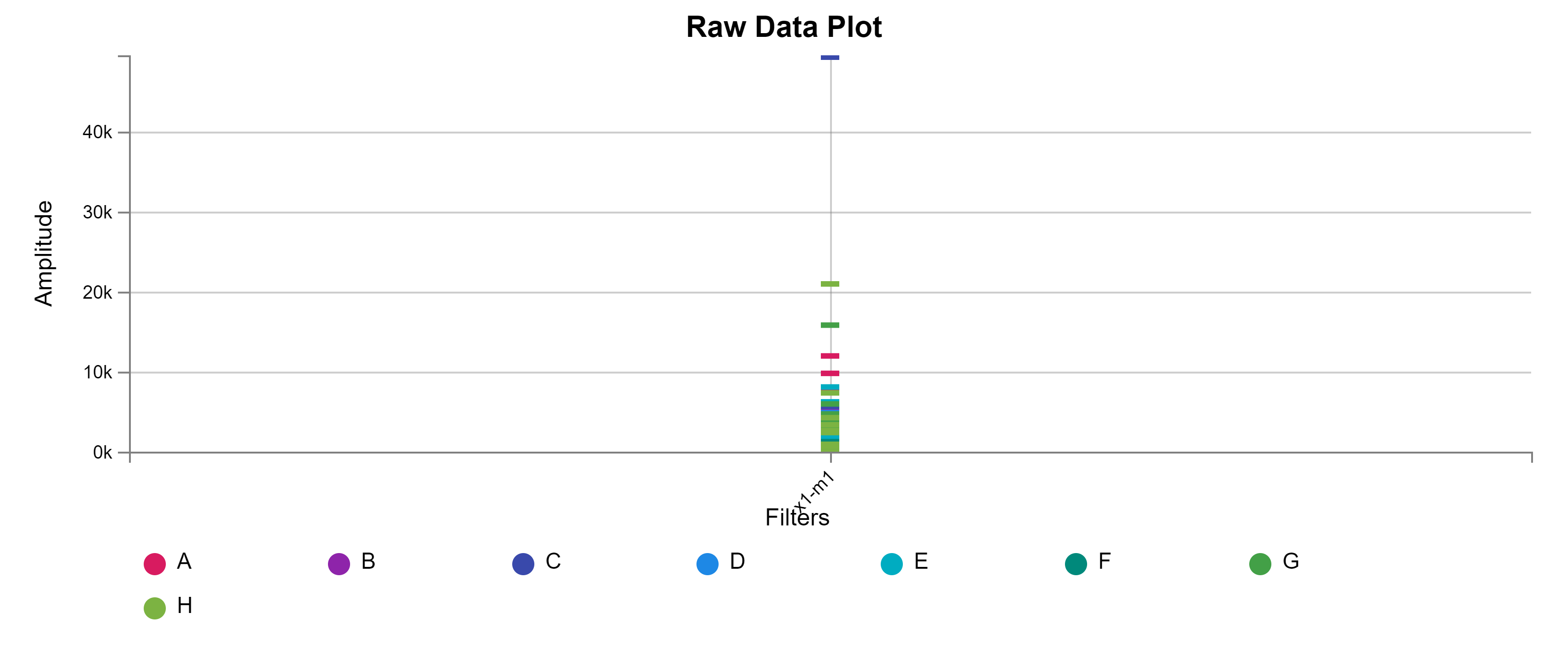

Supplement: Supplemental Information 6 [file peerj-12-16692-s006.zip › original data-figure 2/image/2B/Raw Data Plot_2023-08-17-14203.png]

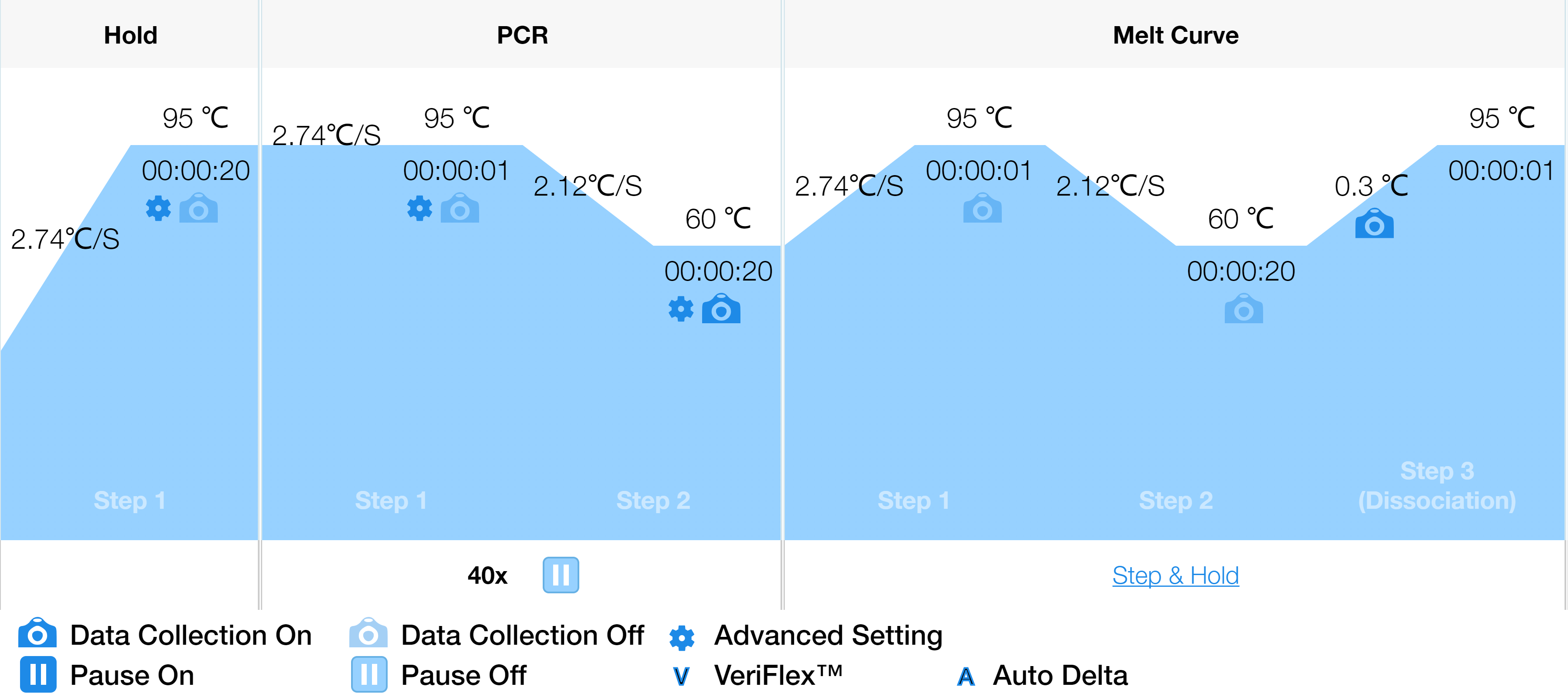

Supplement: Supplemental Information 6 [file peerj-12-16692-s006.zip › original data-figure 2/image/2B/Run_Protocol_2023-08-17-141852.png]

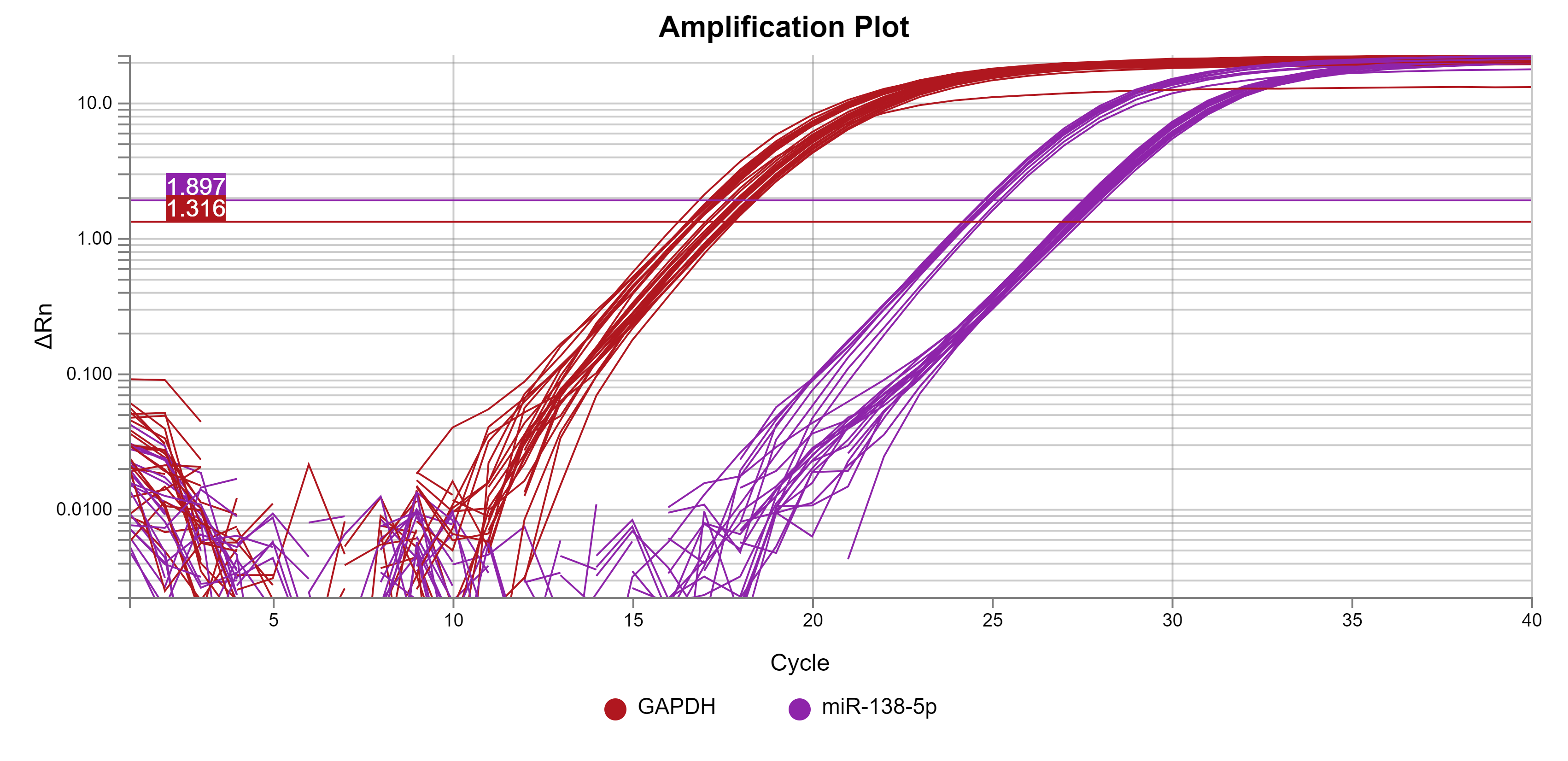

Supplement: Supplemental Information 6 [file peerj-12-16692-s006.zip › original data-figure 2/image/2C/Amplification Plot_2023-08-17-145116.png]

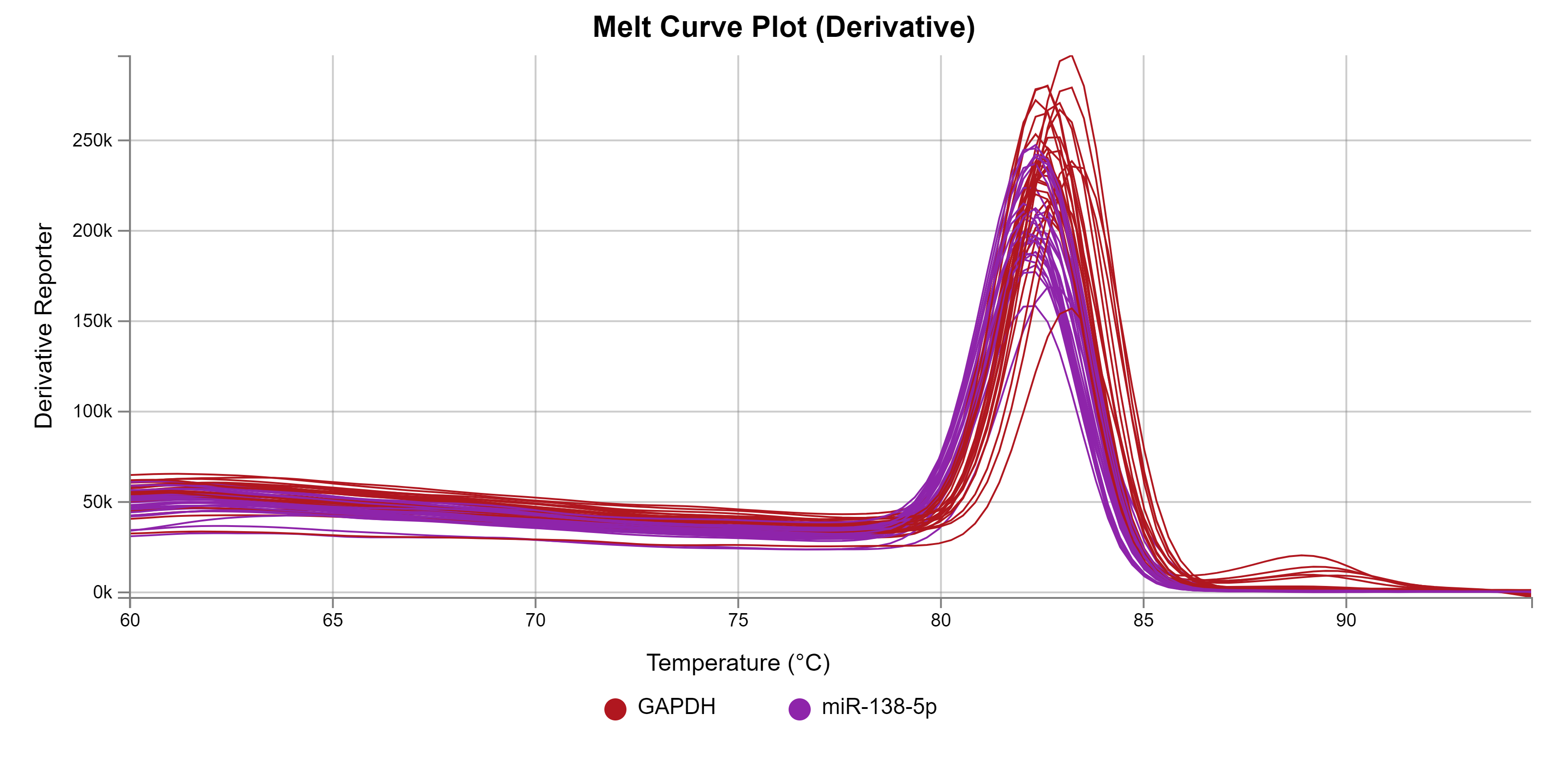

Supplement: Supplemental Information 6 [file peerj-12-16692-s006.zip › original data-figure 2/image/2C/Melt Curve Plot_2023-08-17-145030.png]

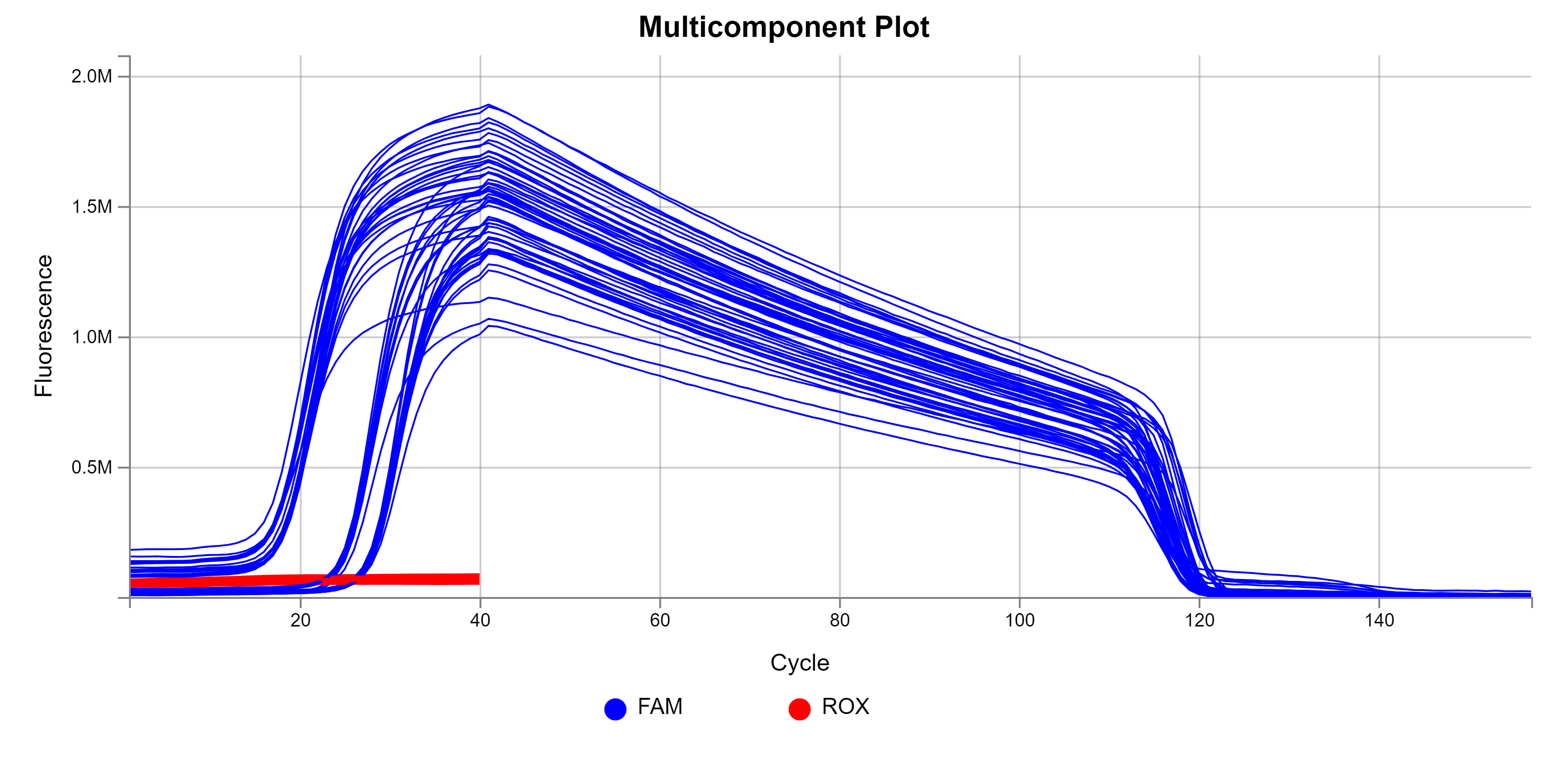

Supplement: Supplemental Information 6 [file peerj-12-16692-s006.zip › original data-figure 2/image/2C/Multicomponent Plot_2023-08-17-145058.png]

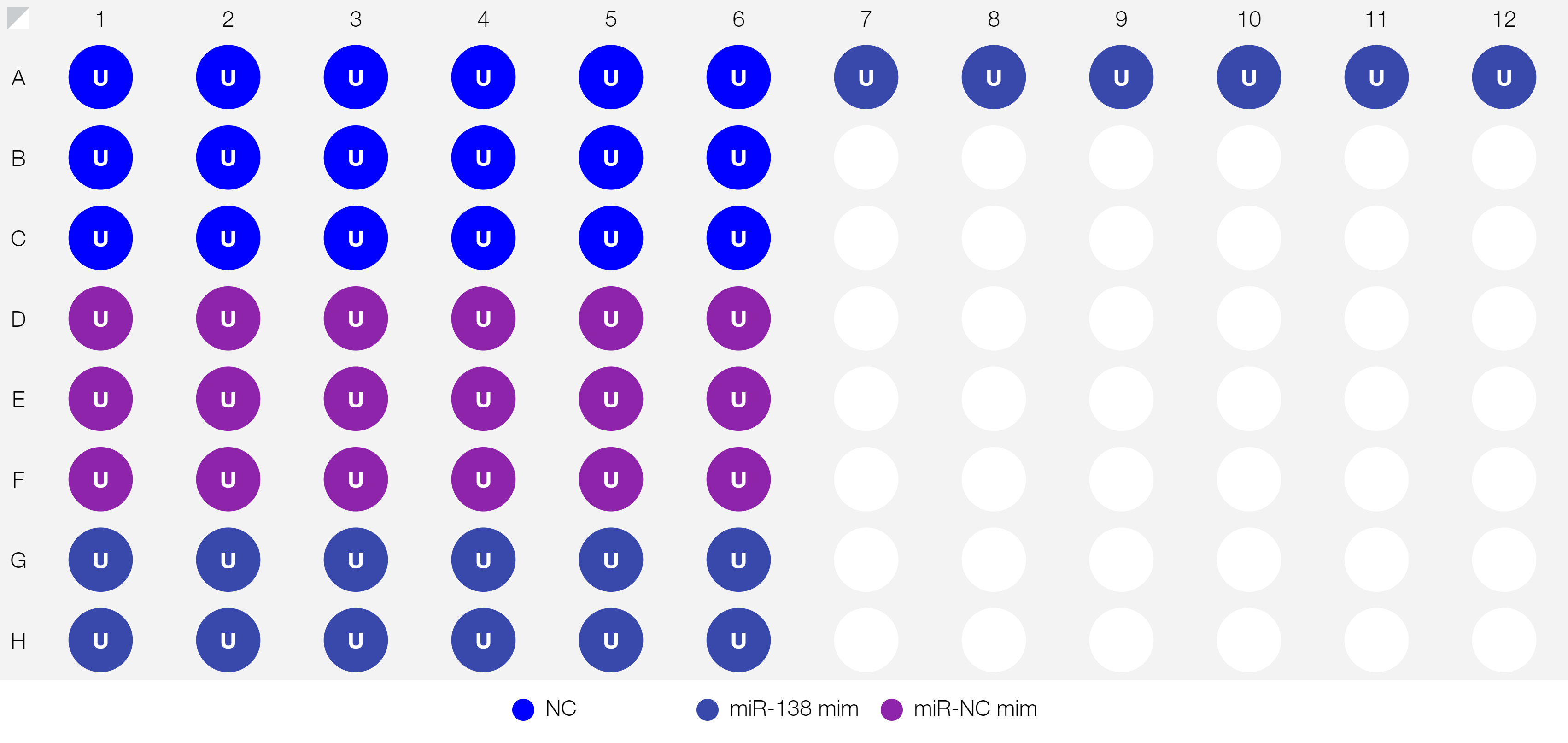

Supplement: Supplemental Information 6 [file peerj-12-16692-s006.zip › original data-figure 2/image/2C/Plate_2023-08-17-145135.png]

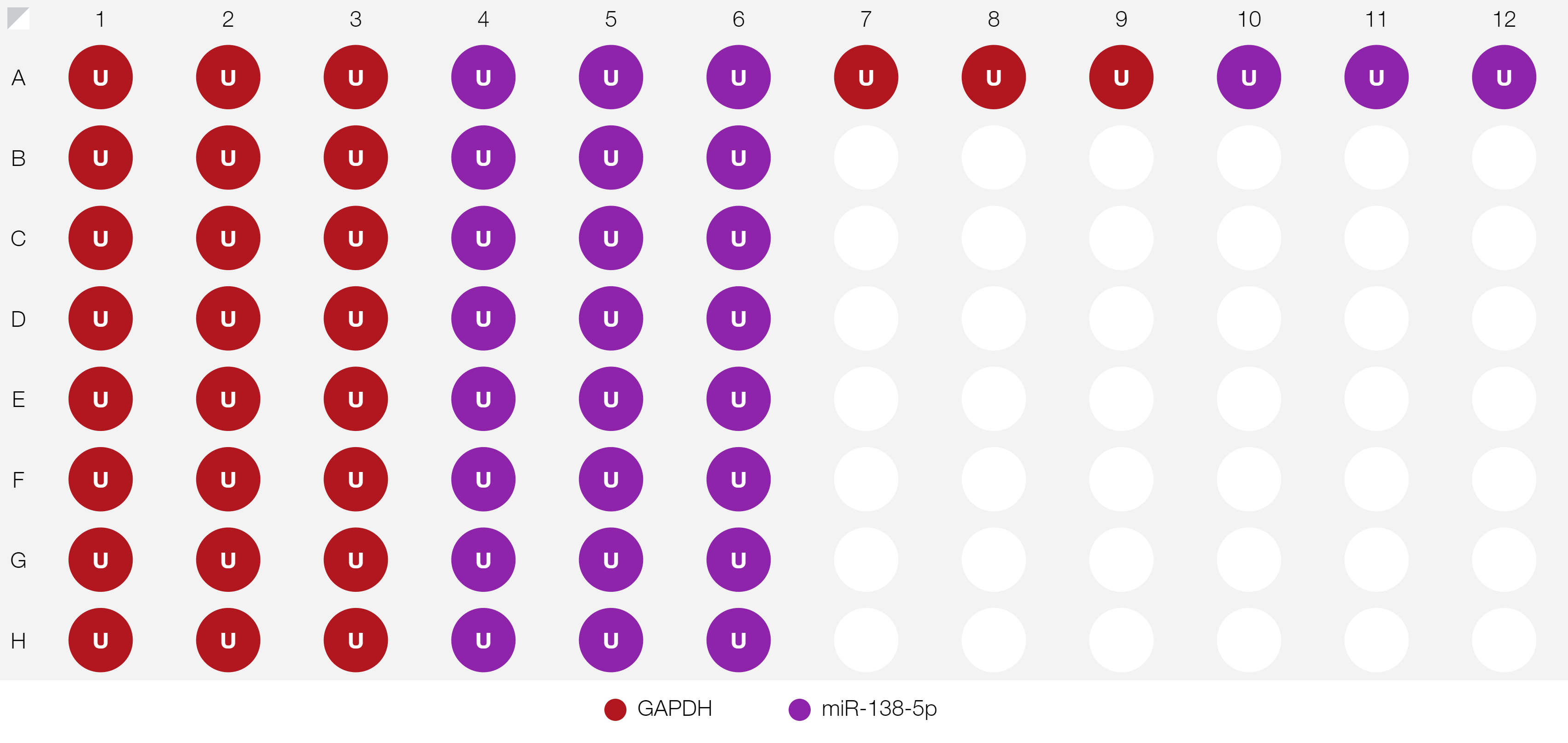

Supplement: Supplemental Information 6 [file peerj-12-16692-s006.zip › original data-figure 2/image/2C/Plate_2023-08-17-145148.png]

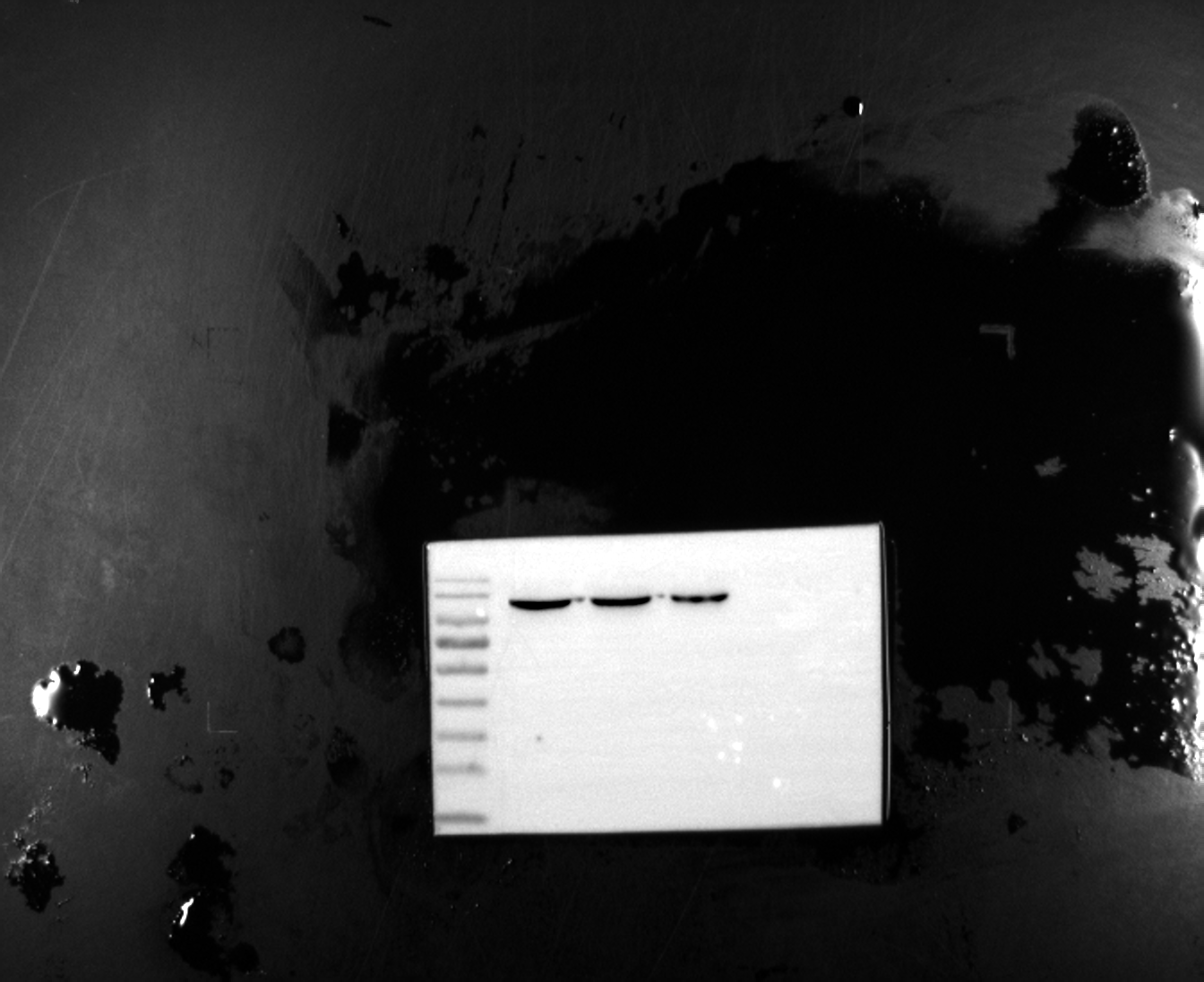

Supplement: Supplemental Information 6 [file peerj-12-16692-s006.zip › original data-figure 2/image/2E/1.NLRP3.tif]

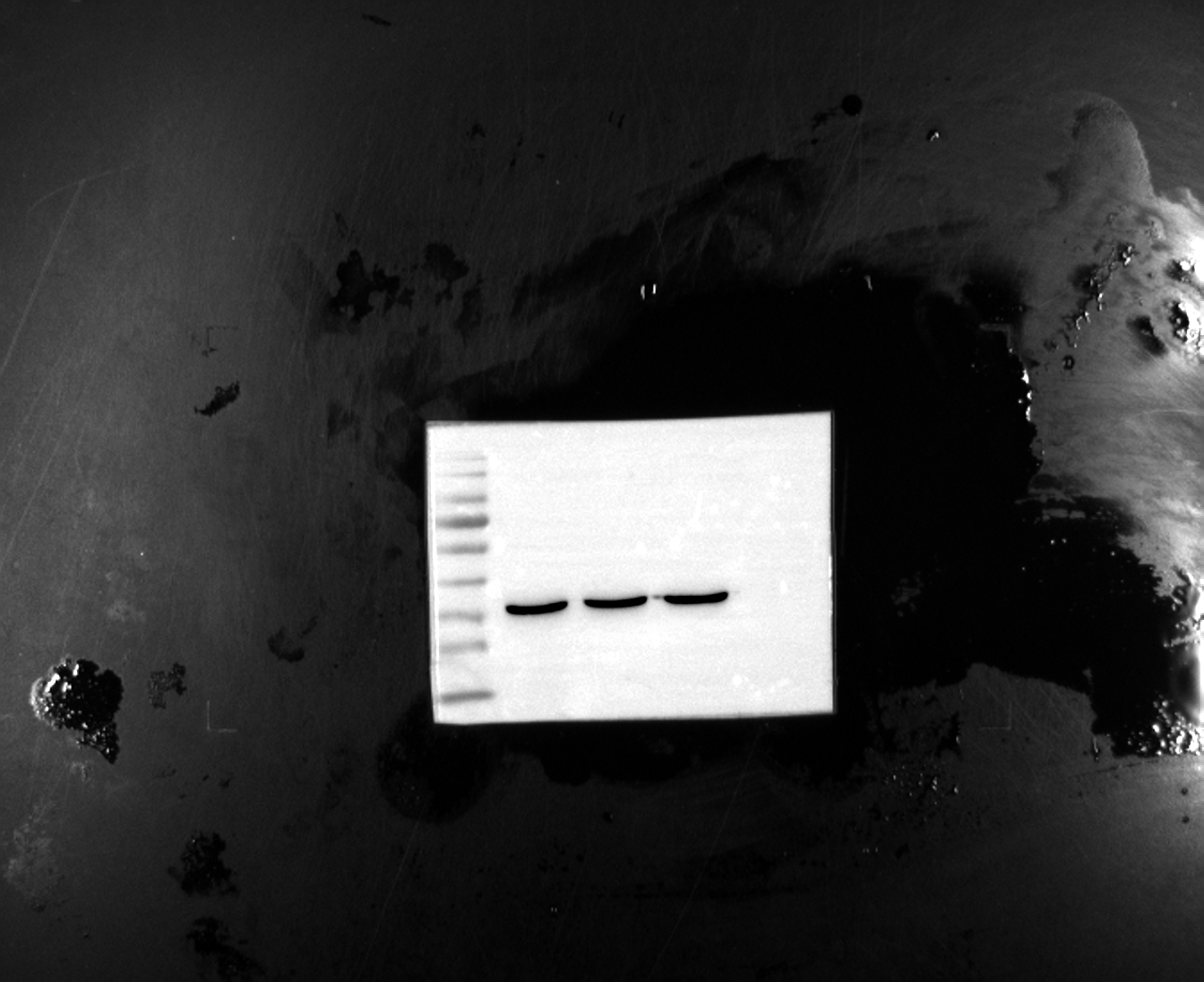

Supplement: Supplemental Information 6 [file peerj-12-16692-s006.zip › original data-figure 2/image/2E/2.GAPDH.tif]

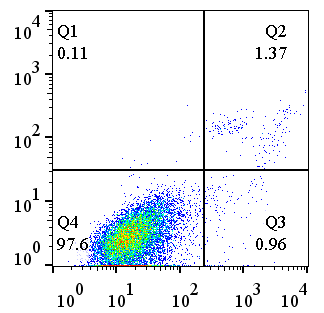

Supplement: Supplemental Information 7 [file peerj-12-16692-s007.zip › original data-figure 3/3C/1.NC.png]

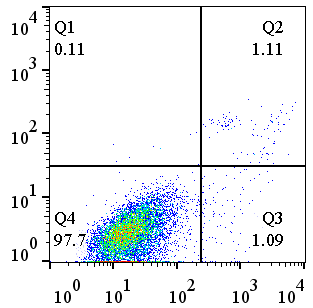

Supplement: Supplemental Information 7 [file peerj-12-16692-s007.zip › original data-figure 3/3C/2.miR-NC inh.png]

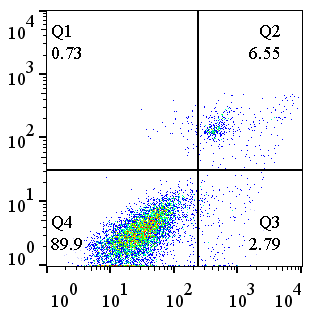

Supplement: Supplemental Information 7 [file peerj-12-16692-s007.zip › original data-figure 3/3C/3.miR-138 inh.png]

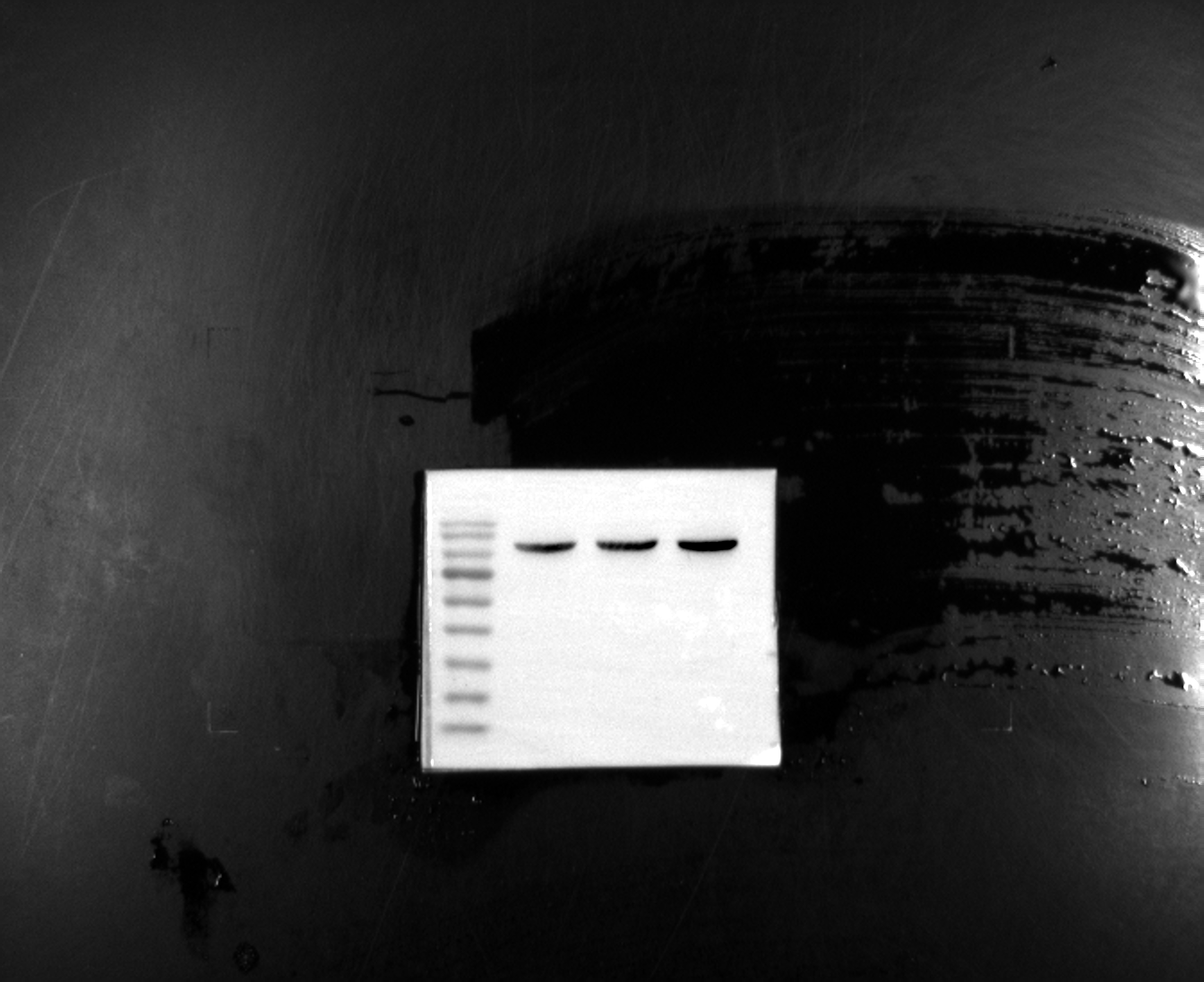

Supplement: Supplemental Information 7 [file peerj-12-16692-s007.zip › original data-figure 3/3D/1.NLRP3.tif]

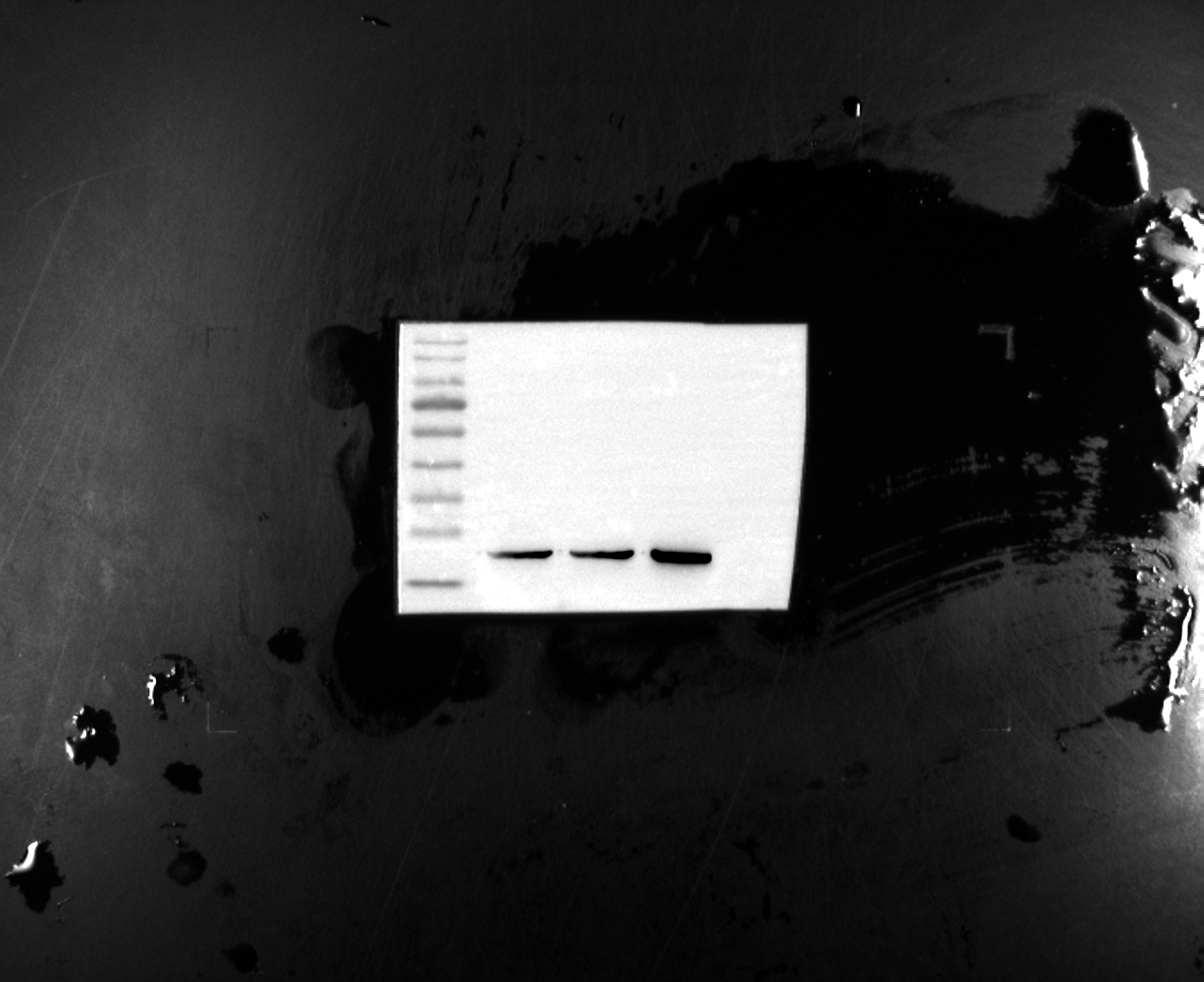

Supplement: Supplemental Information 7 [file peerj-12-16692-s007.zip › original data-figure 3/3D/2.ASC.tif]

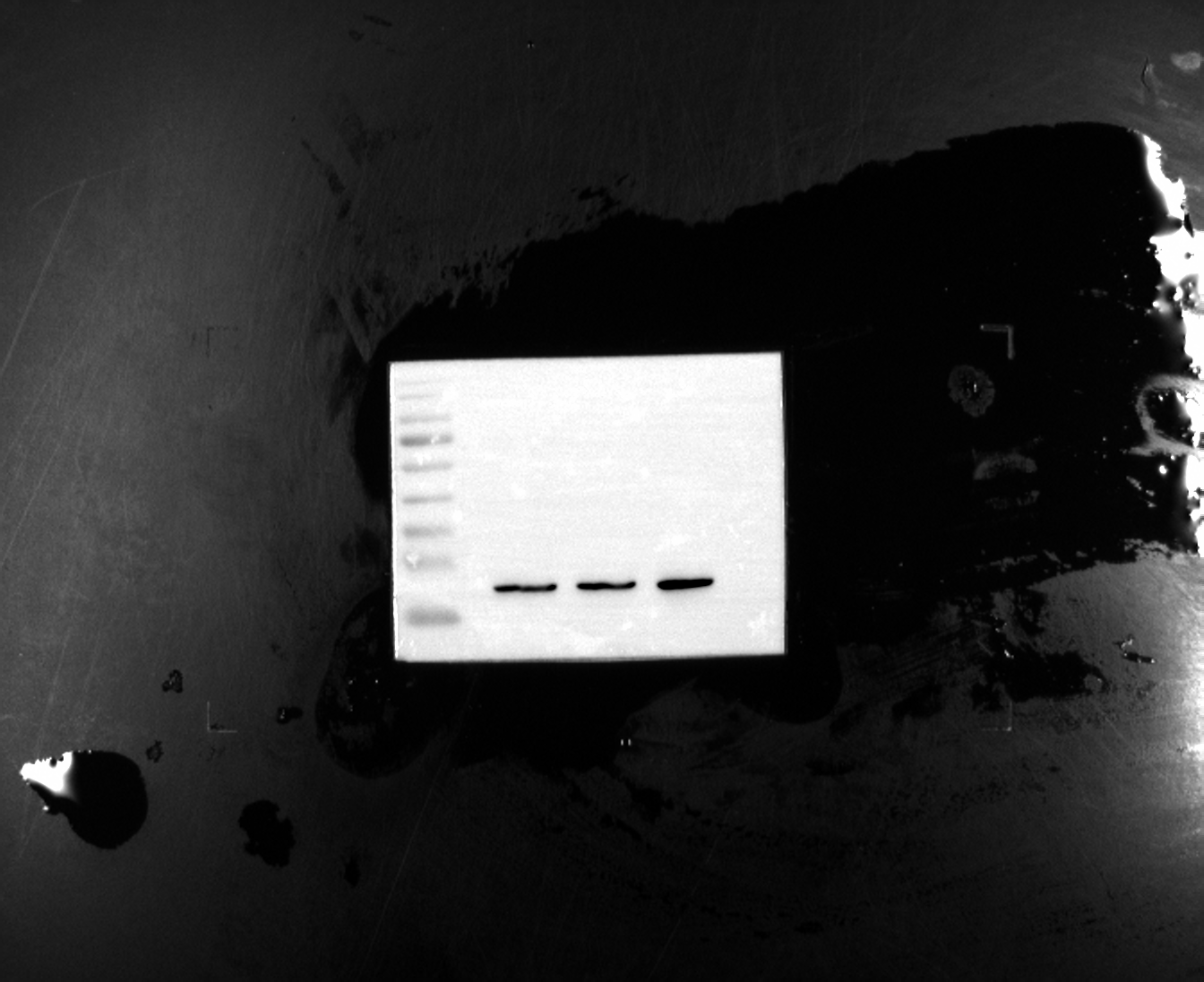

Supplement: Supplemental Information 7 [file peerj-12-16692-s007.zip › original data-figure 3/3D/3.Caspase 1.tif]

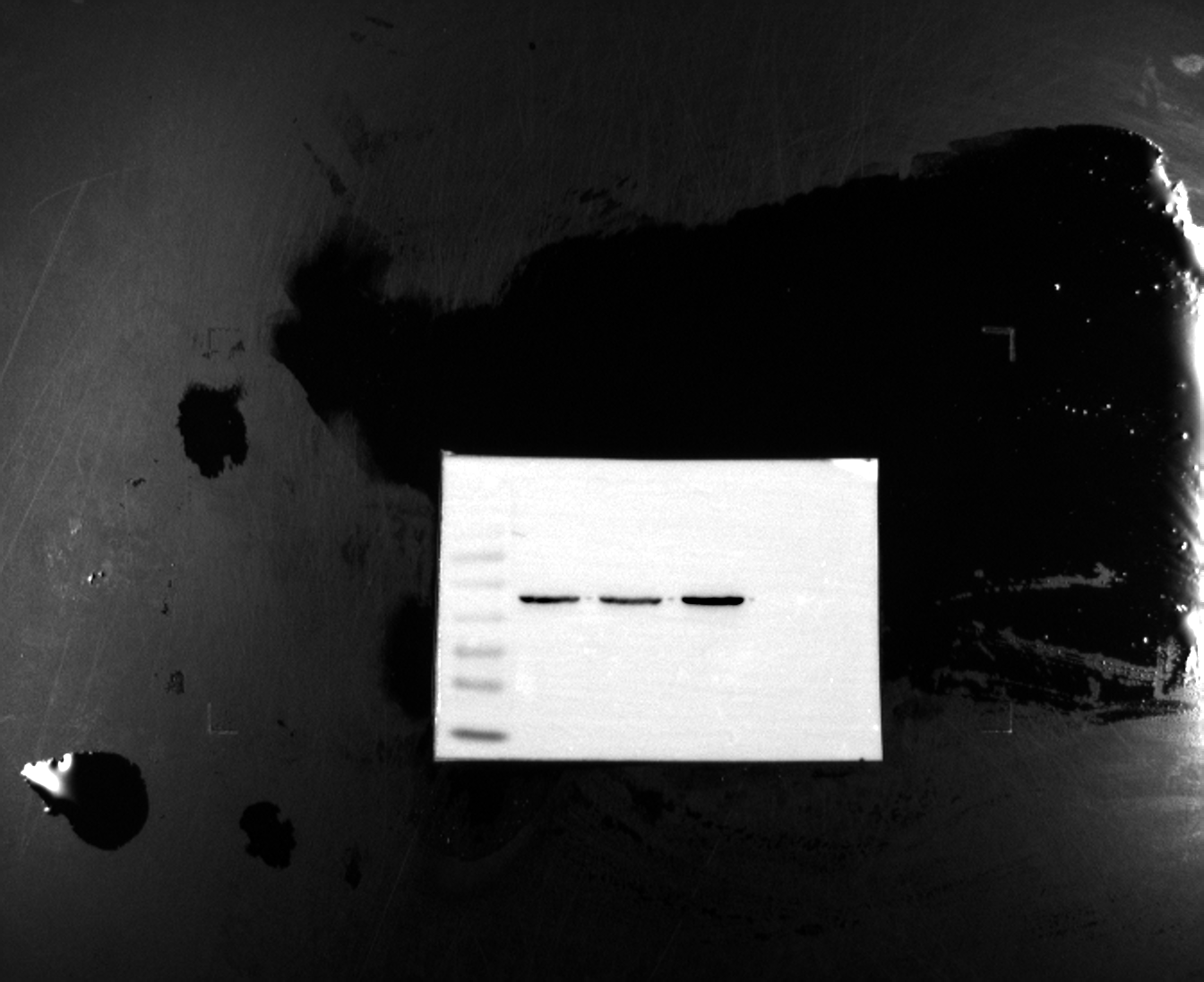

Supplement: Supplemental Information 7 [file peerj-12-16692-s007.zip › original data-figure 3/3D/4.GSDMD-N.tif]

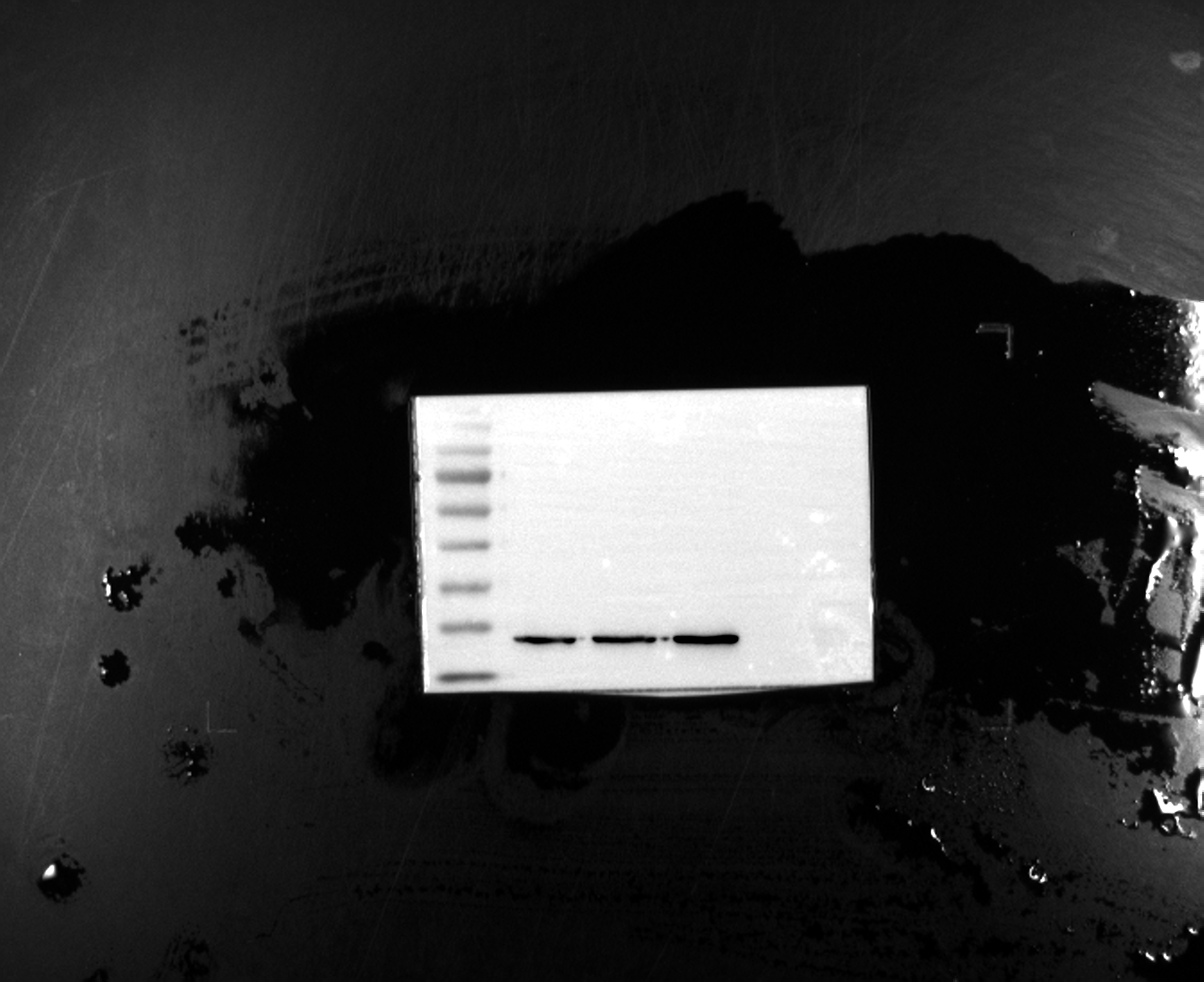

Supplement: Supplemental Information 7 [file peerj-12-16692-s007.zip › original data-figure 3/3D/5.IL-18.tif]

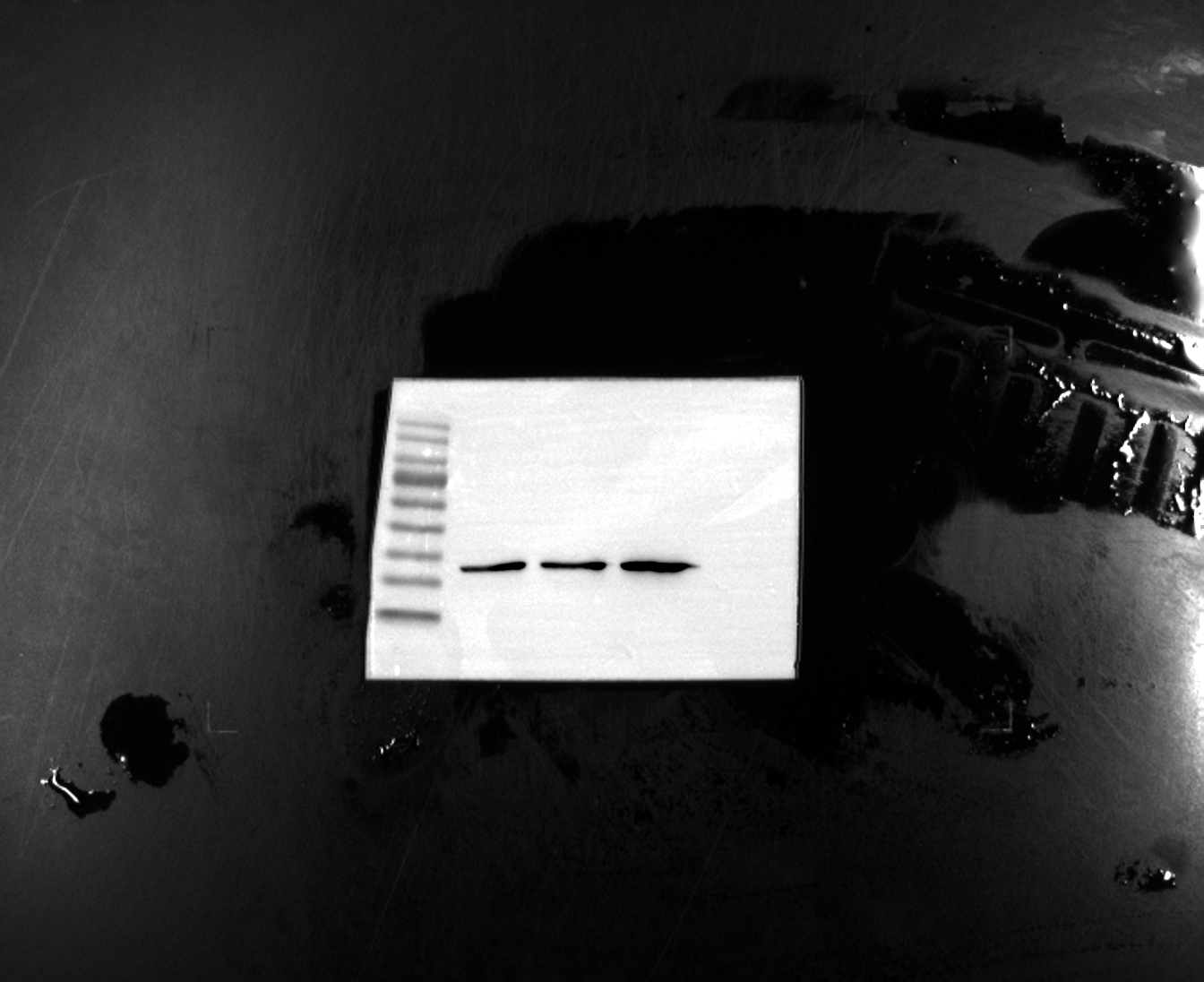

Supplement: Supplemental Information 7 [file peerj-12-16692-s007.zip › original data-figure 3/3D/6.IL-1β.tif]

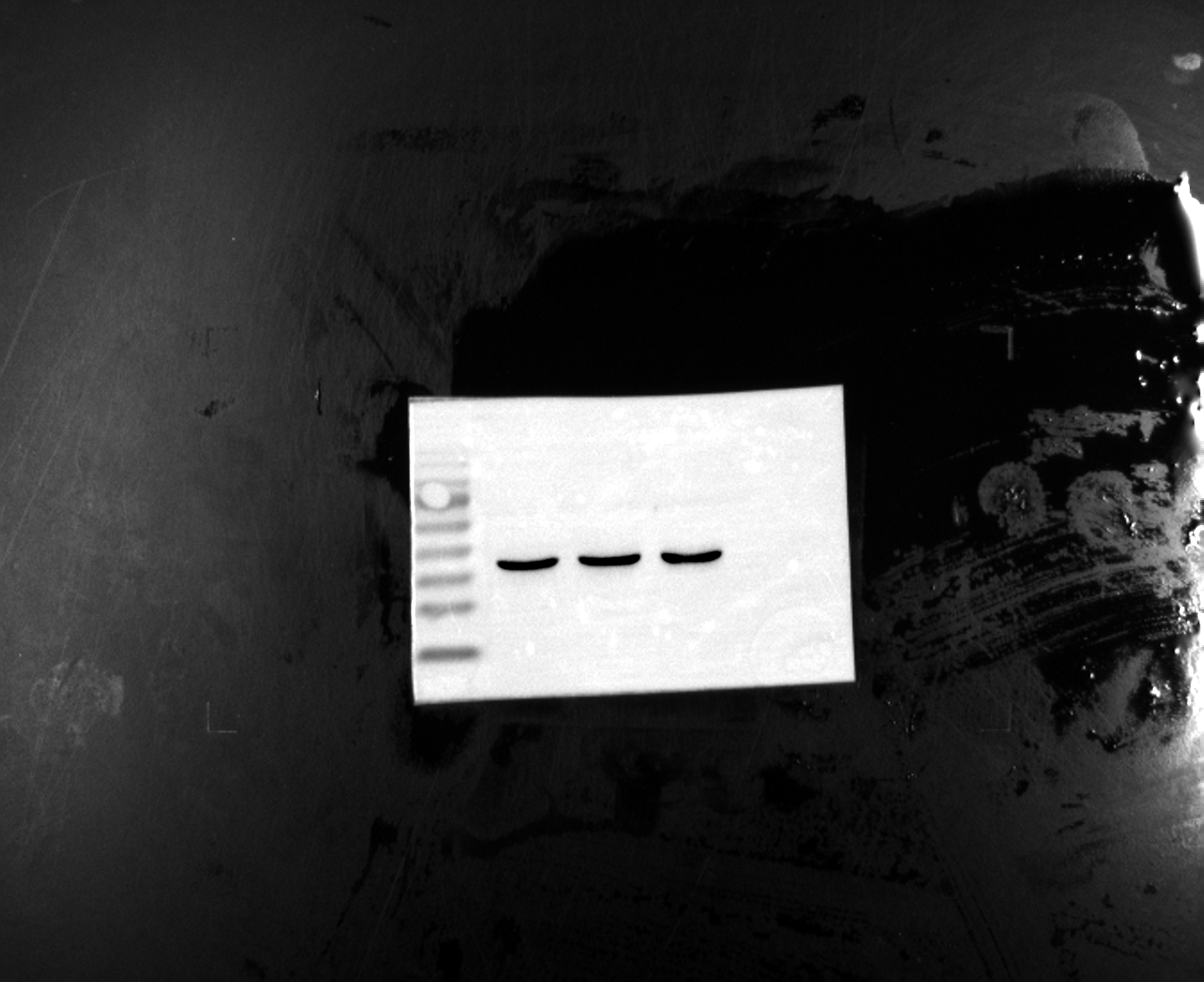

Supplement: Supplemental Information 7 [file peerj-12-16692-s007.zip › original data-figure 3/3D/7.GAPDH.tif]

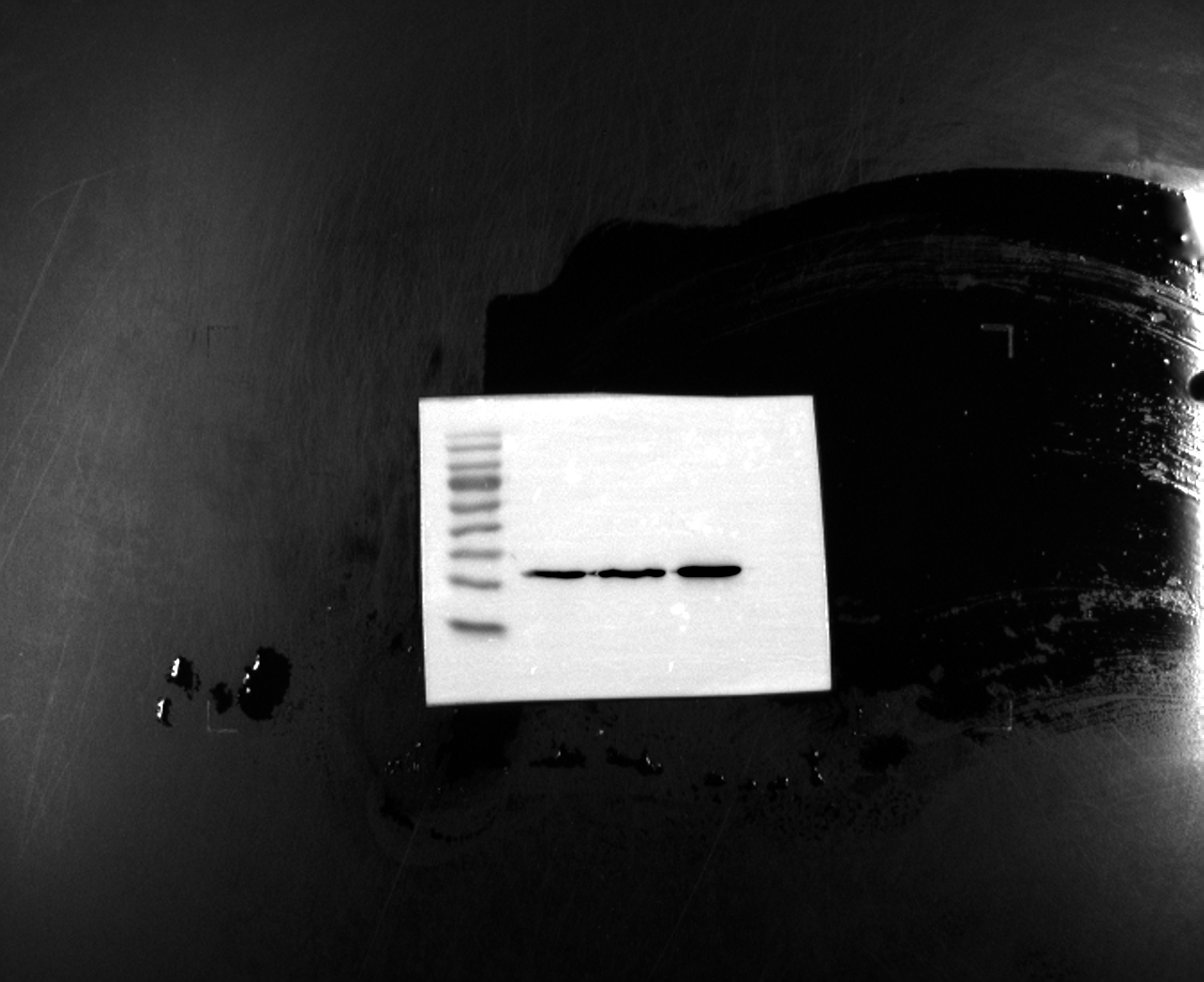

Supplement: Supplemental Information 7 [file peerj-12-16692-s007.zip › original data-figure 3/3E/1.HMGB1.tif]

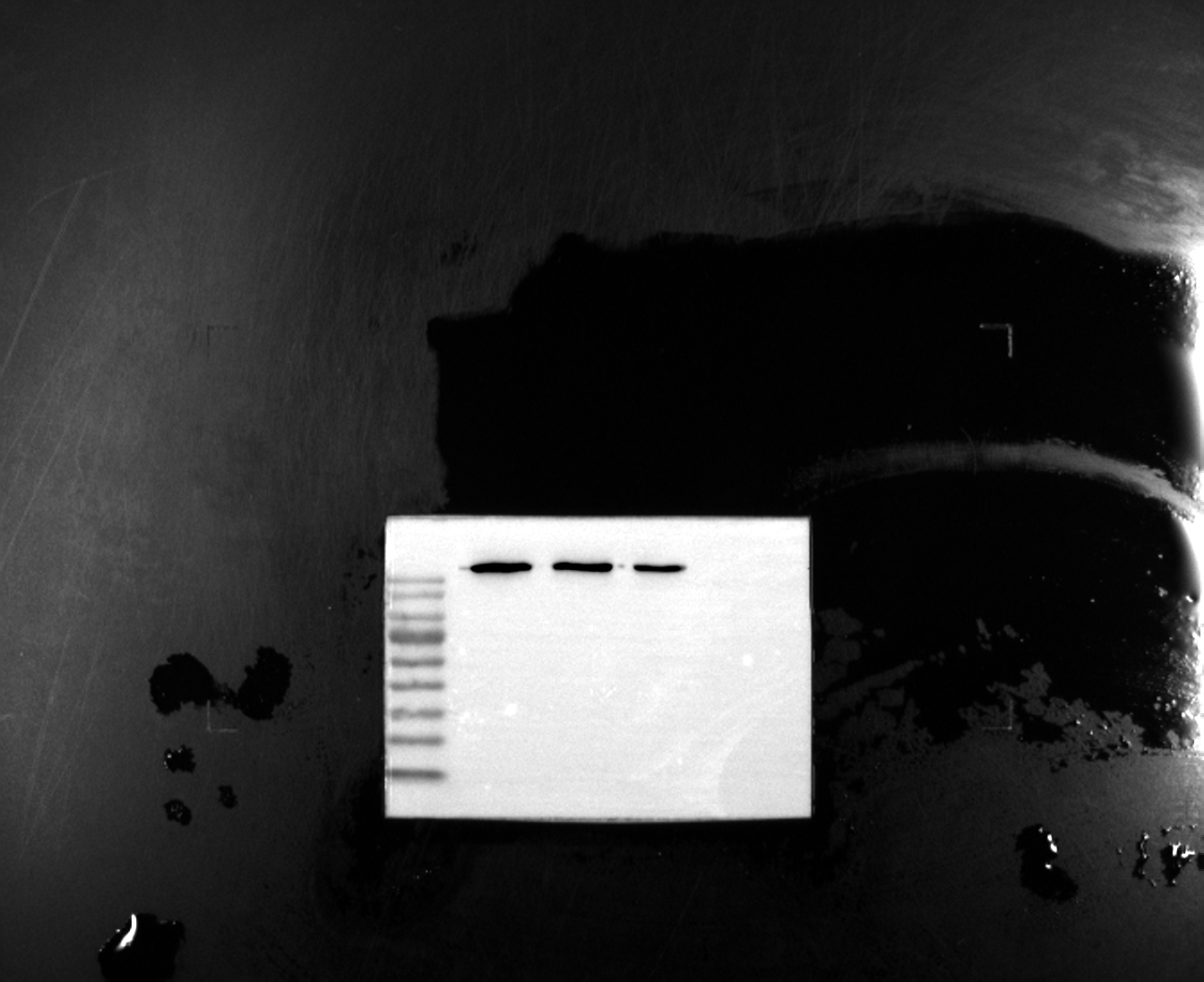

Supplement: Supplemental Information 7 [file peerj-12-16692-s007.zip › original data-figure 3/3E/2.ZO-1.tif]

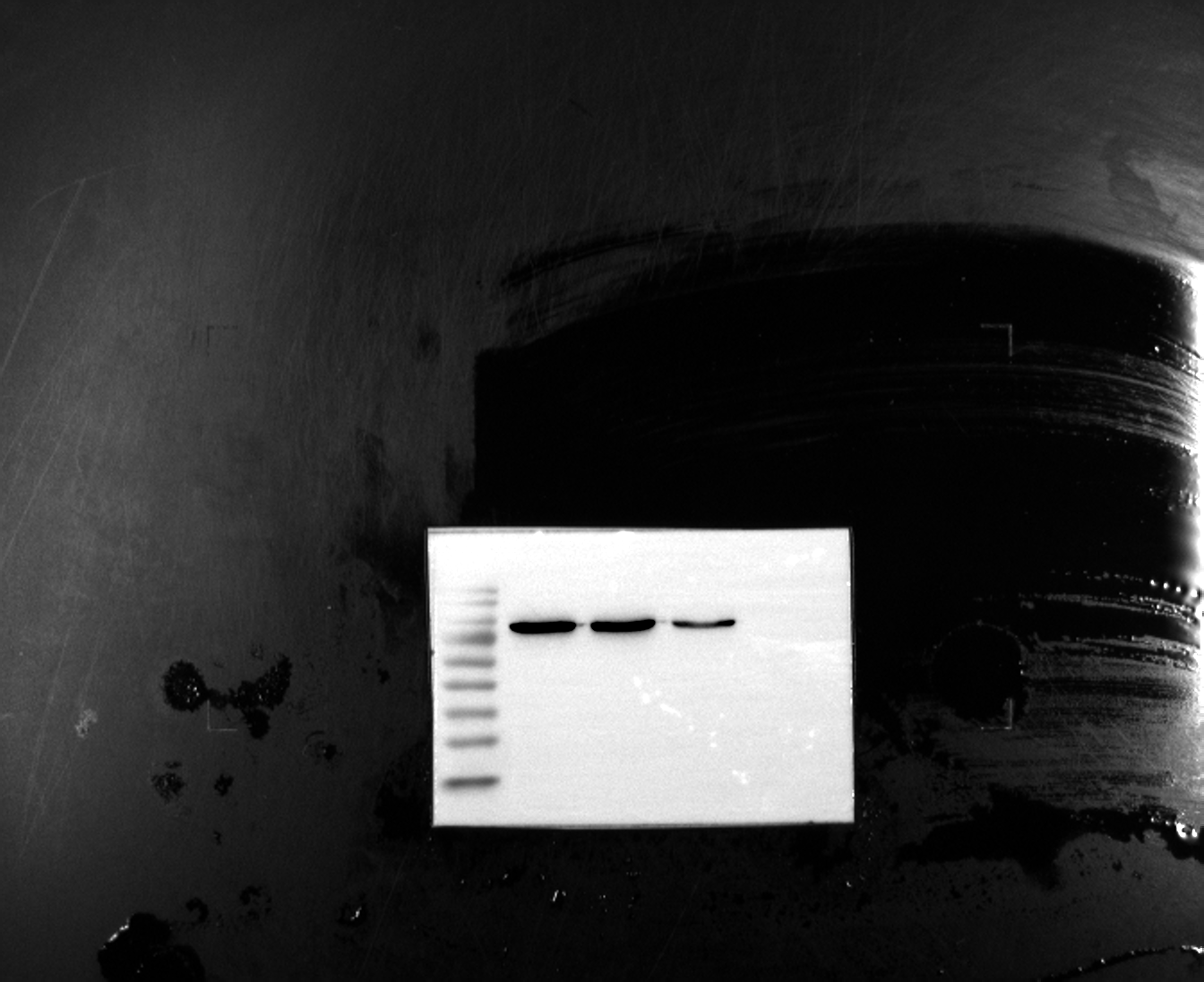

Supplement: Supplemental Information 7 [file peerj-12-16692-s007.zip › original data-figure 3/3E/3.Occludin.tif]

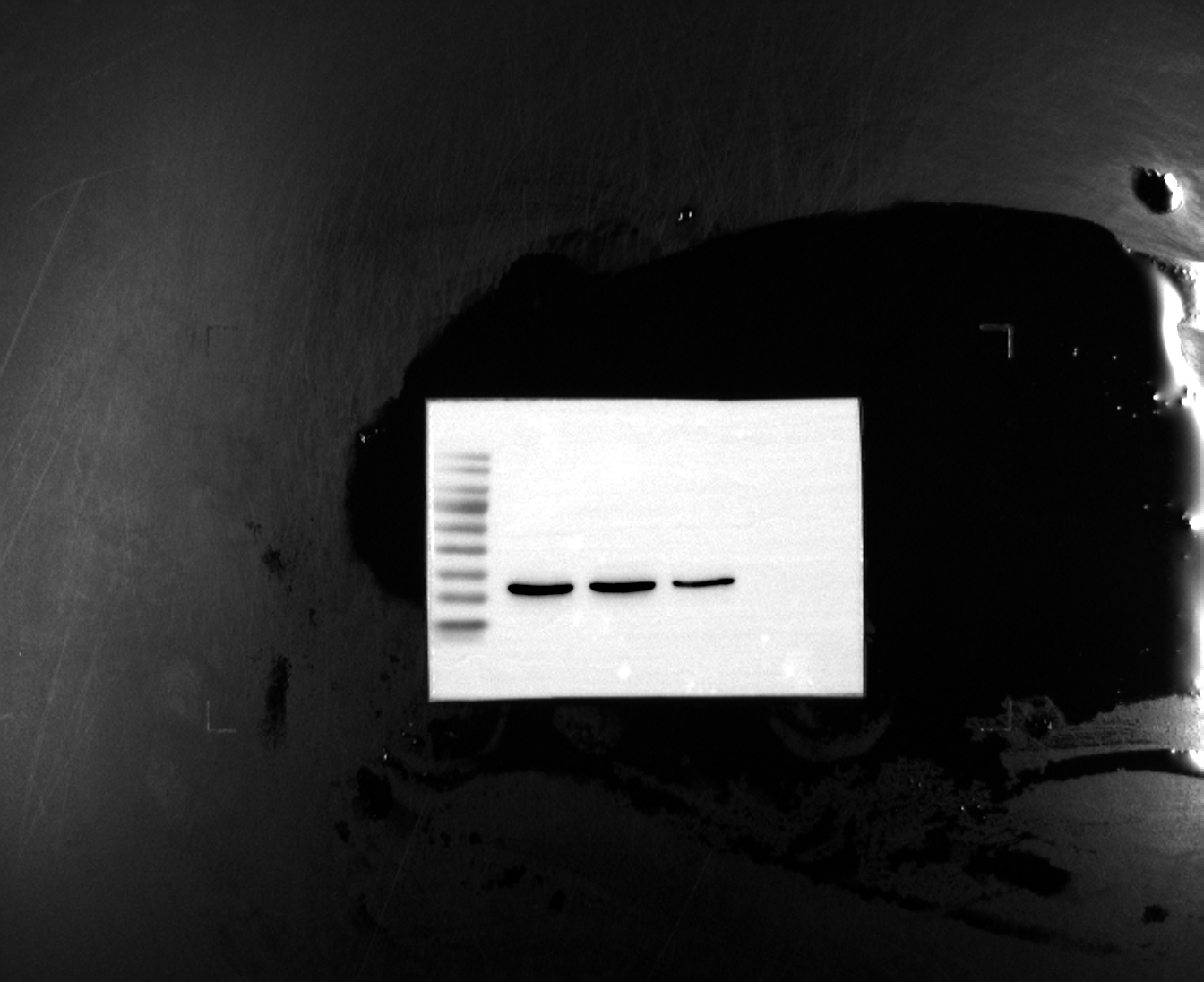

Supplement: Supplemental Information 7 [file peerj-12-16692-s007.zip › original data-figure 3/3E/4.Claudin-1.tif]

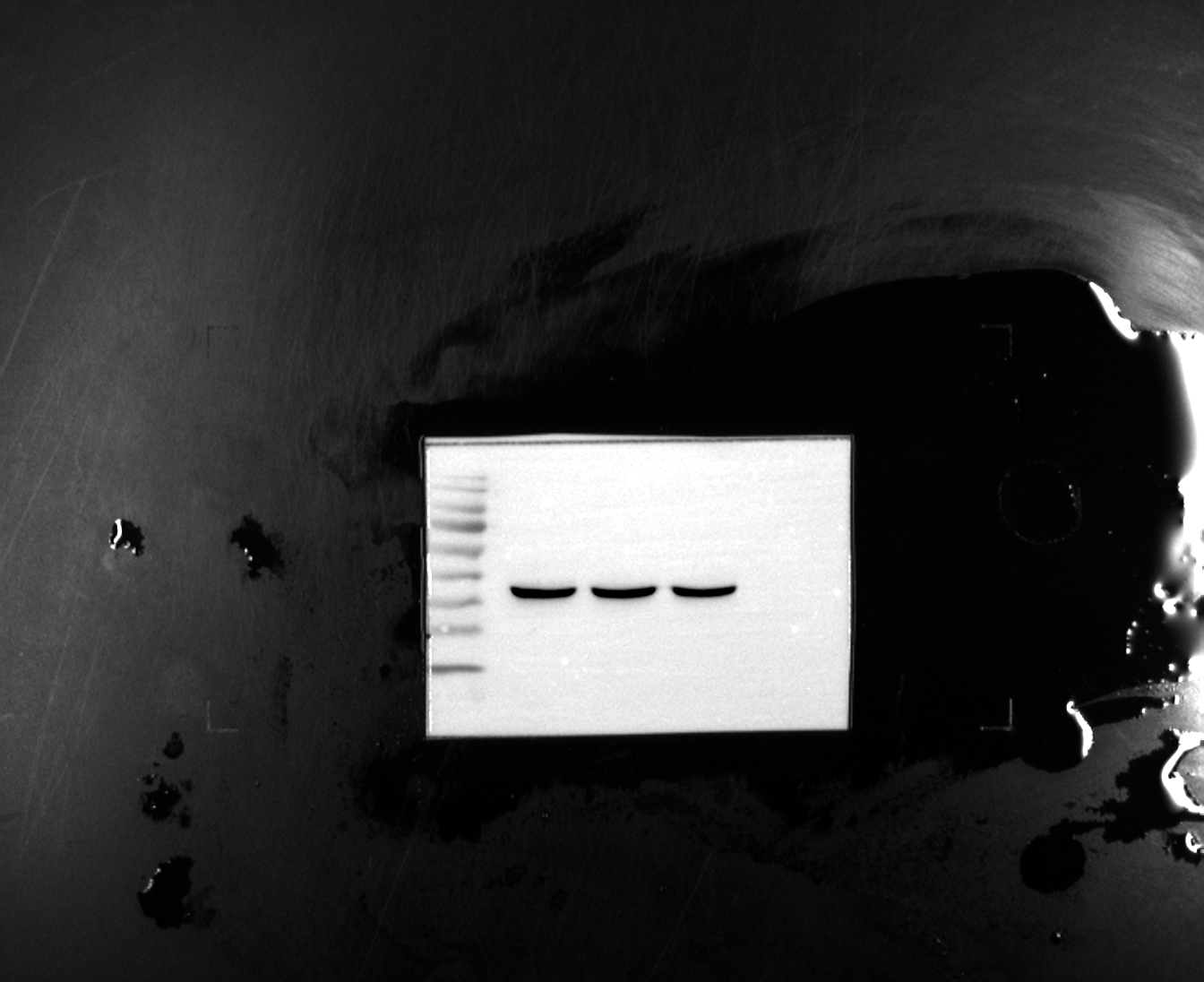

Supplement: Supplemental Information 7 [file peerj-12-16692-s007.zip › original data-figure 3/3E/5.GAPDH.tif]

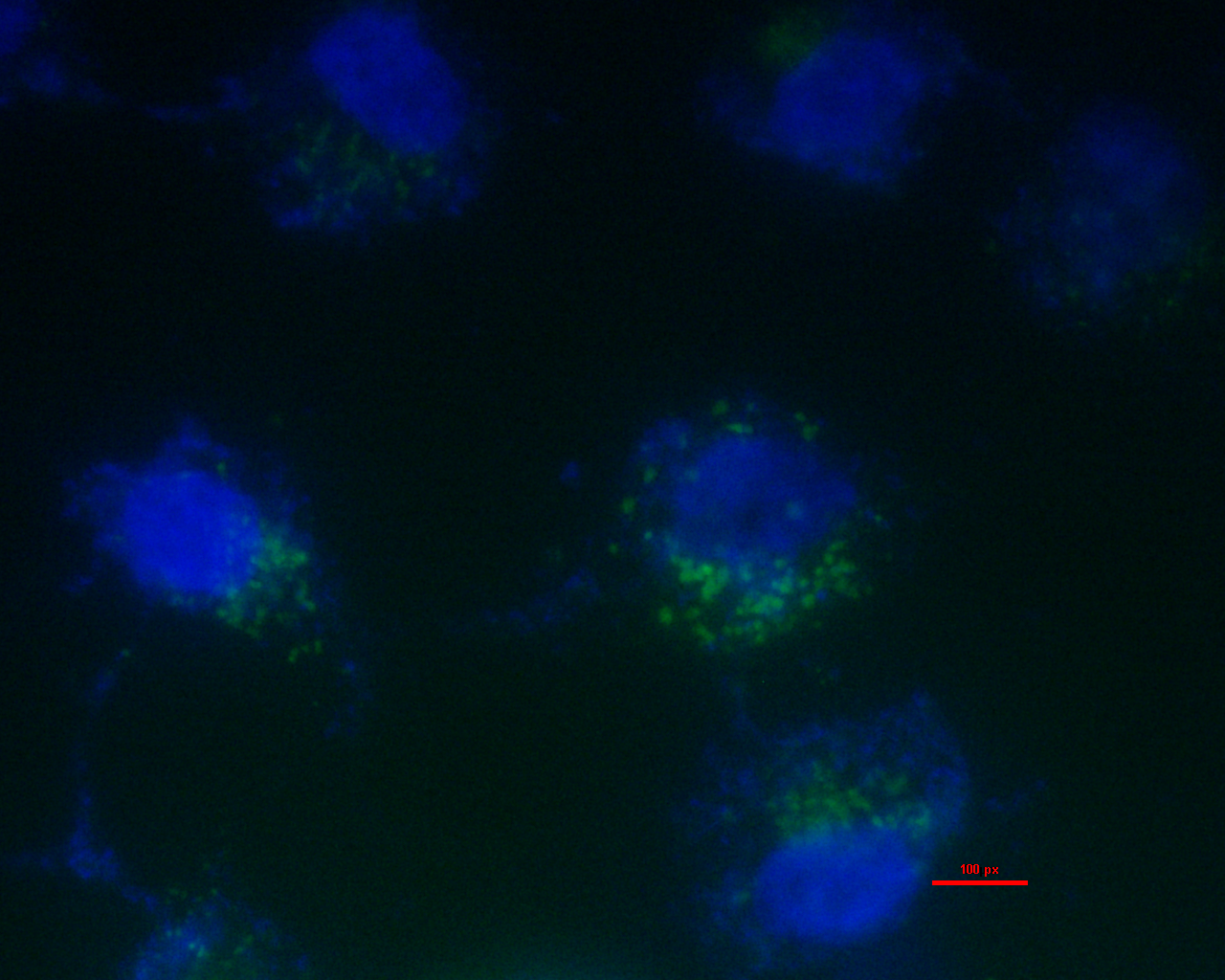

Supplement: Supplemental Information 7 [file peerj-12-16692-s007.zip › original data-figure 3/3F/NC/1.NLRP3.tif]

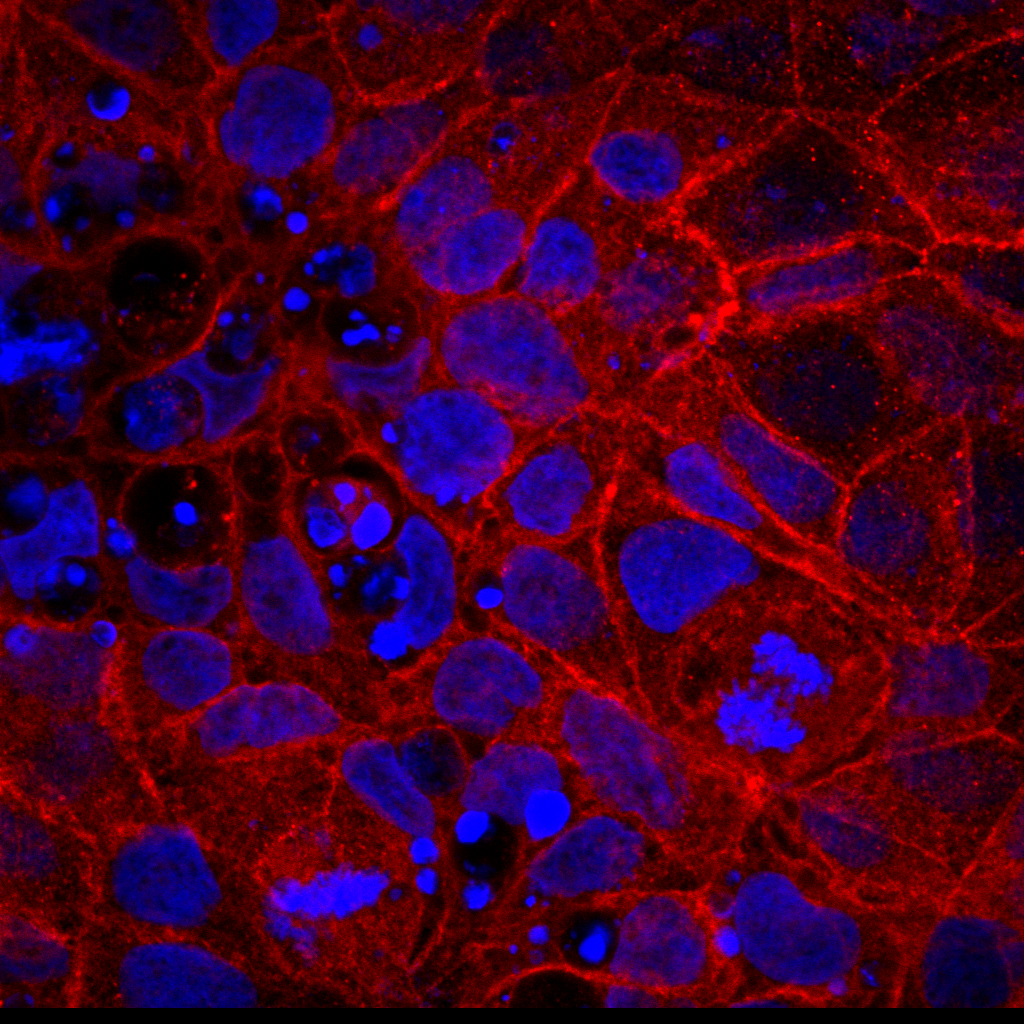

Supplement: Supplemental Information 7 [file peerj-12-16692-s007.zip › original data-figure 3/3F/NC/2.ZO-1.tif]

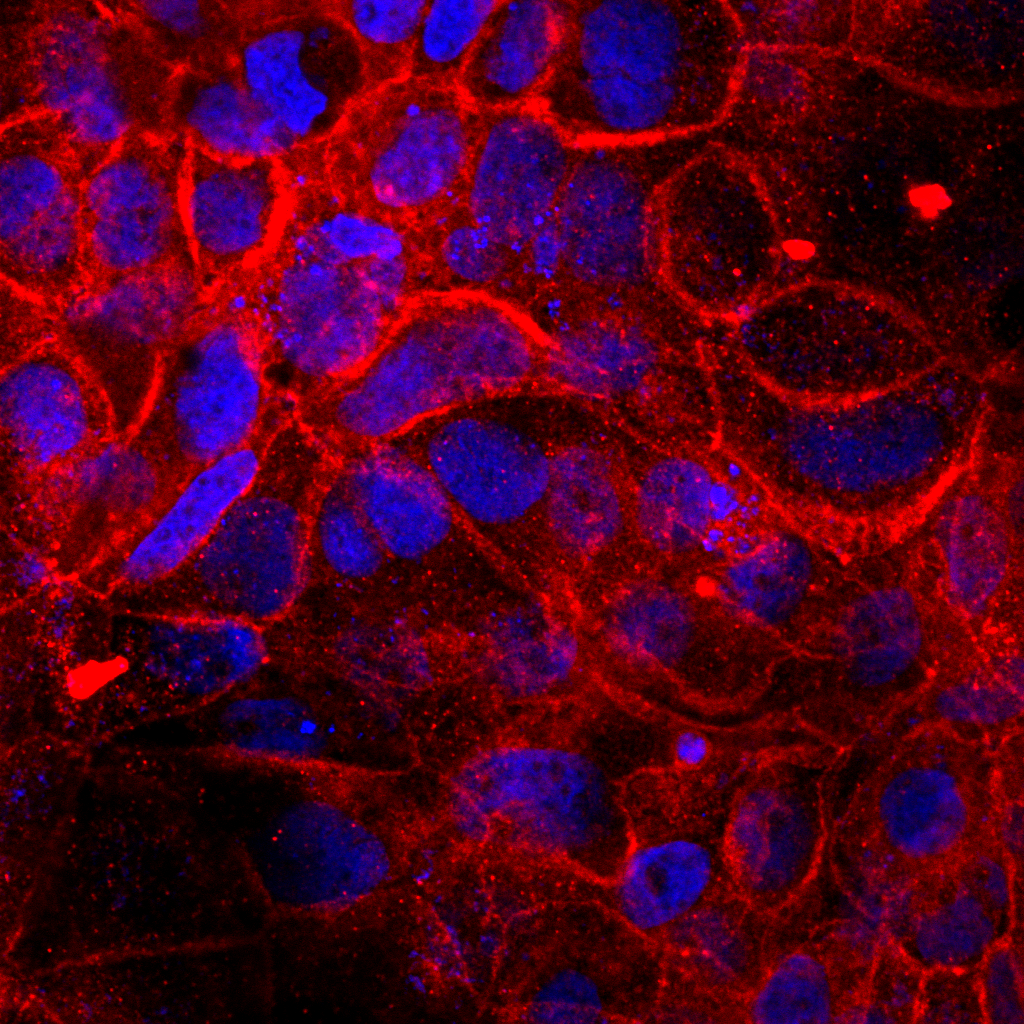

Supplement: Supplemental Information 7 [file peerj-12-16692-s007.zip › original data-figure 3/3F/NC/3.Occludin.tif]

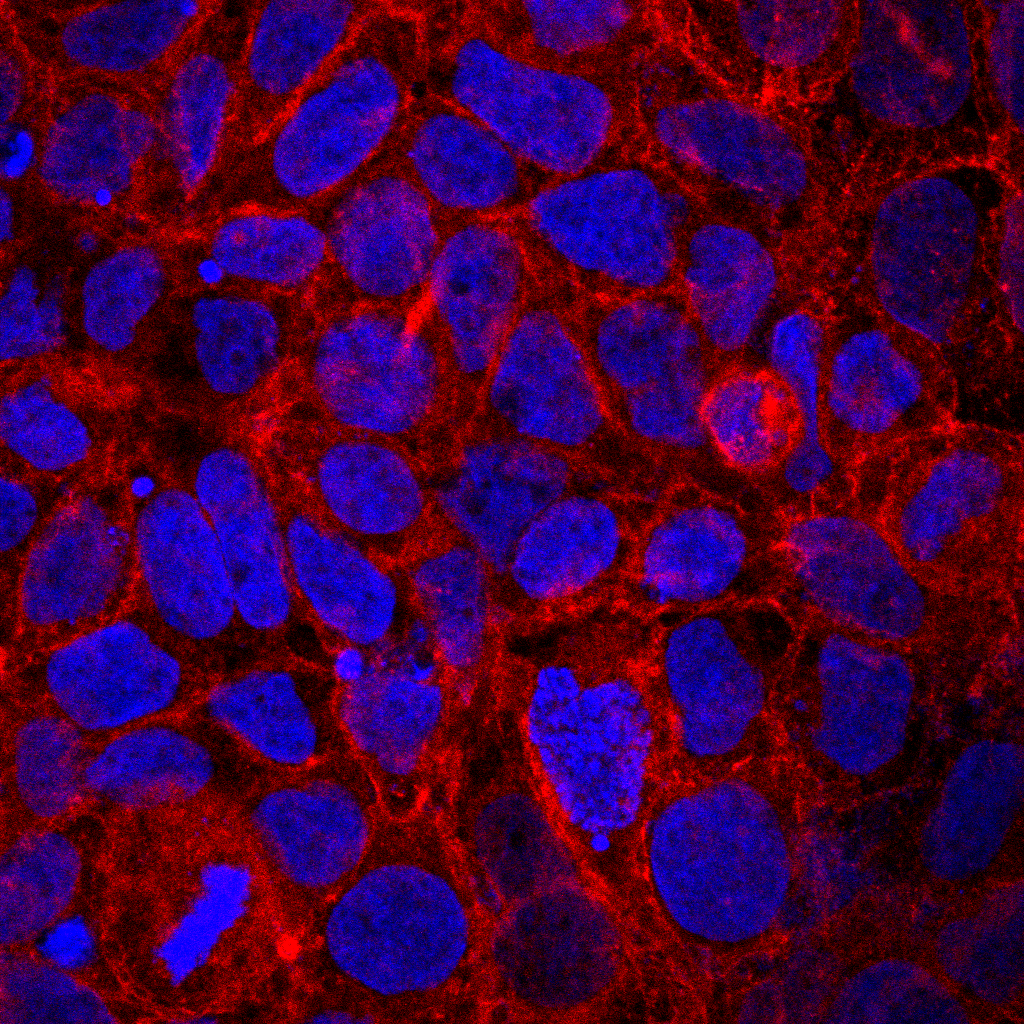

Supplement: Supplemental Information 7 [file peerj-12-16692-s007.zip › original data-figure 3/3F/NC/4.Claudin-1.tif]

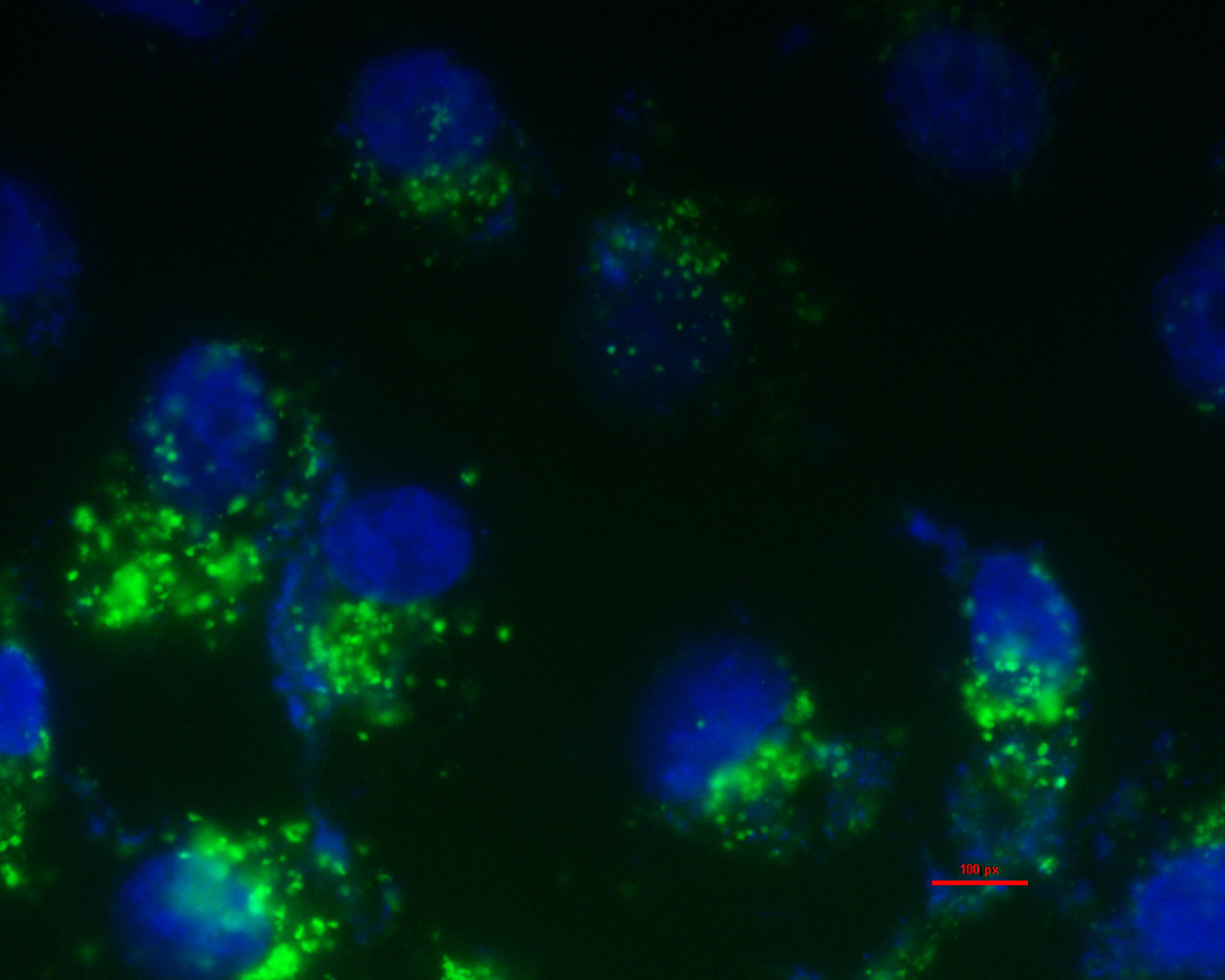

Supplement: Supplemental Information 7 [file peerj-12-16692-s007.zip › original data-figure 3/3F/miR-138 inhi/1.NLRP3.tif]

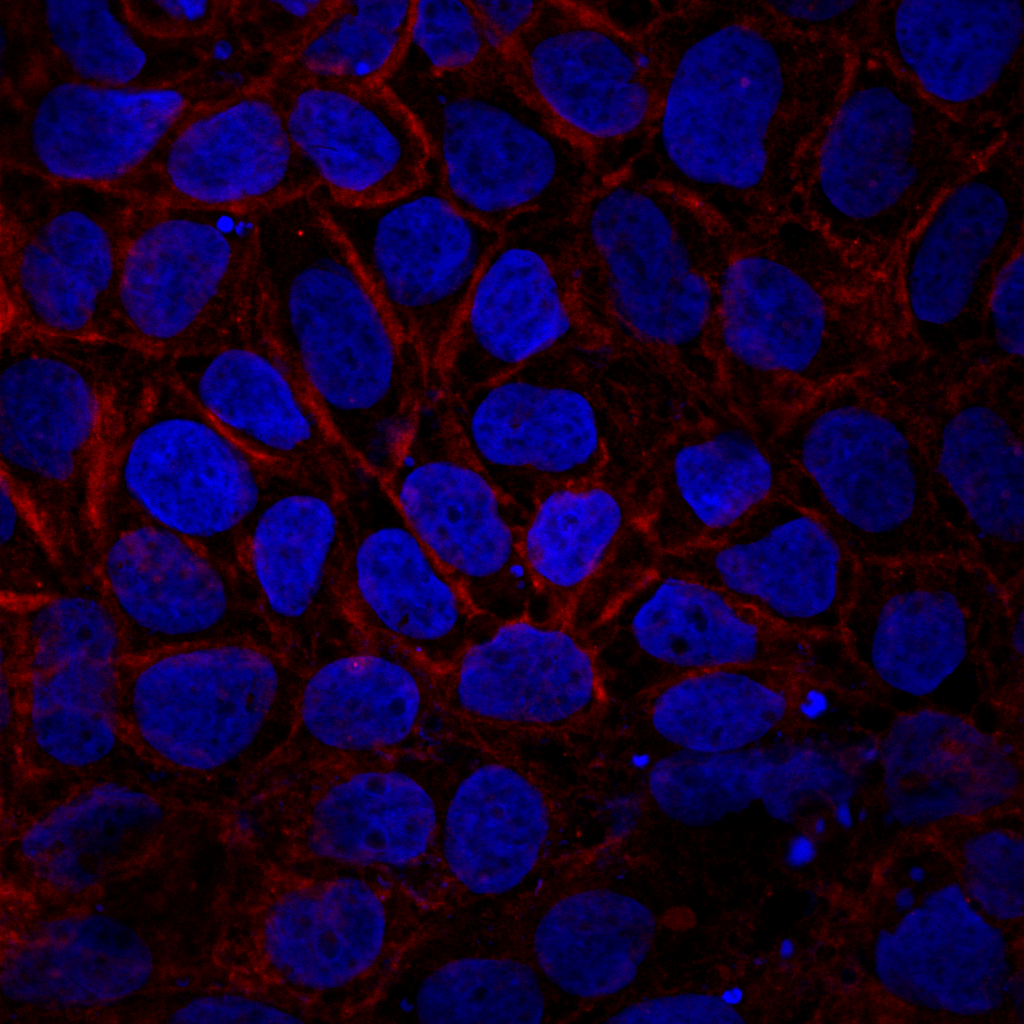

Supplement: Supplemental Information 7 [file peerj-12-16692-s007.zip › original data-figure 3/3F/miR-138 inhi/2.ZO-1.tif]

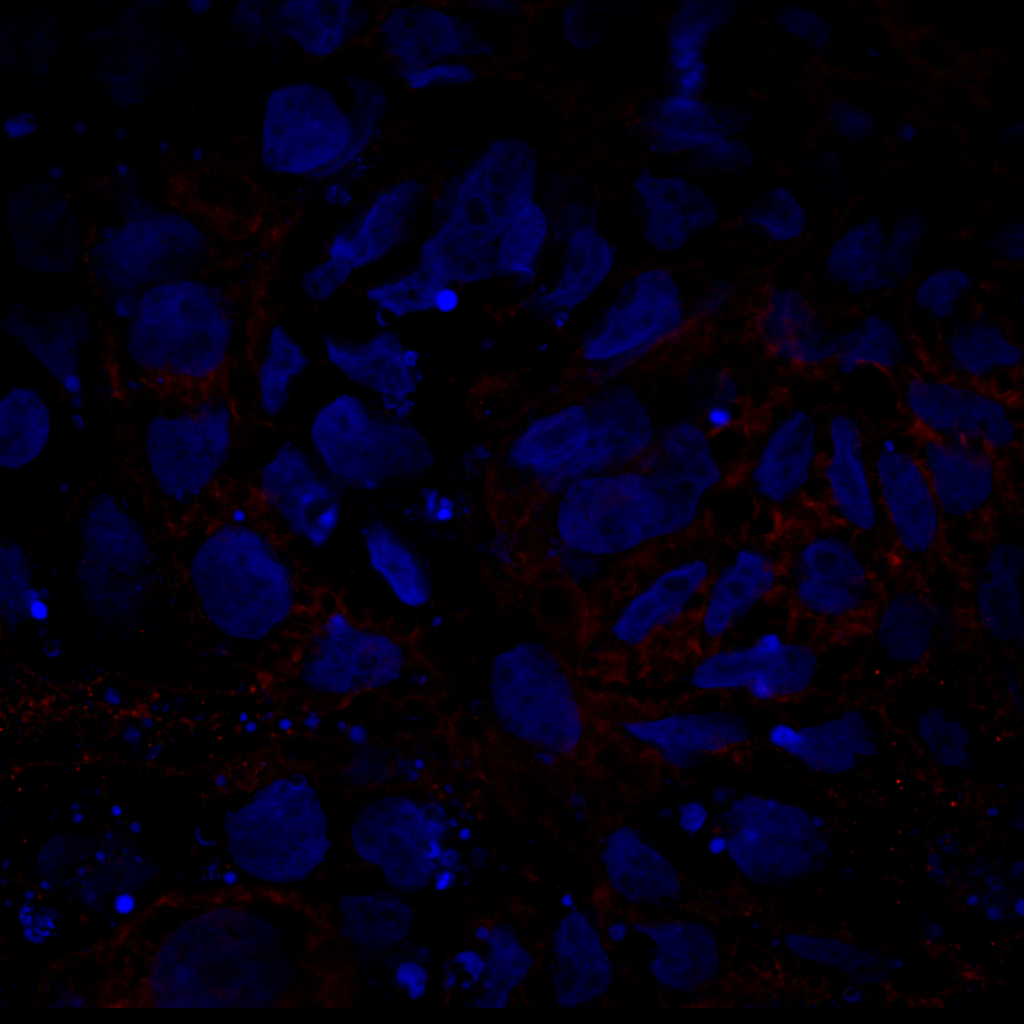

Supplement: Supplemental Information 7 [file peerj-12-16692-s007.zip › original data-figure 3/3F/miR-138 inhi/3.Occludin.tif]

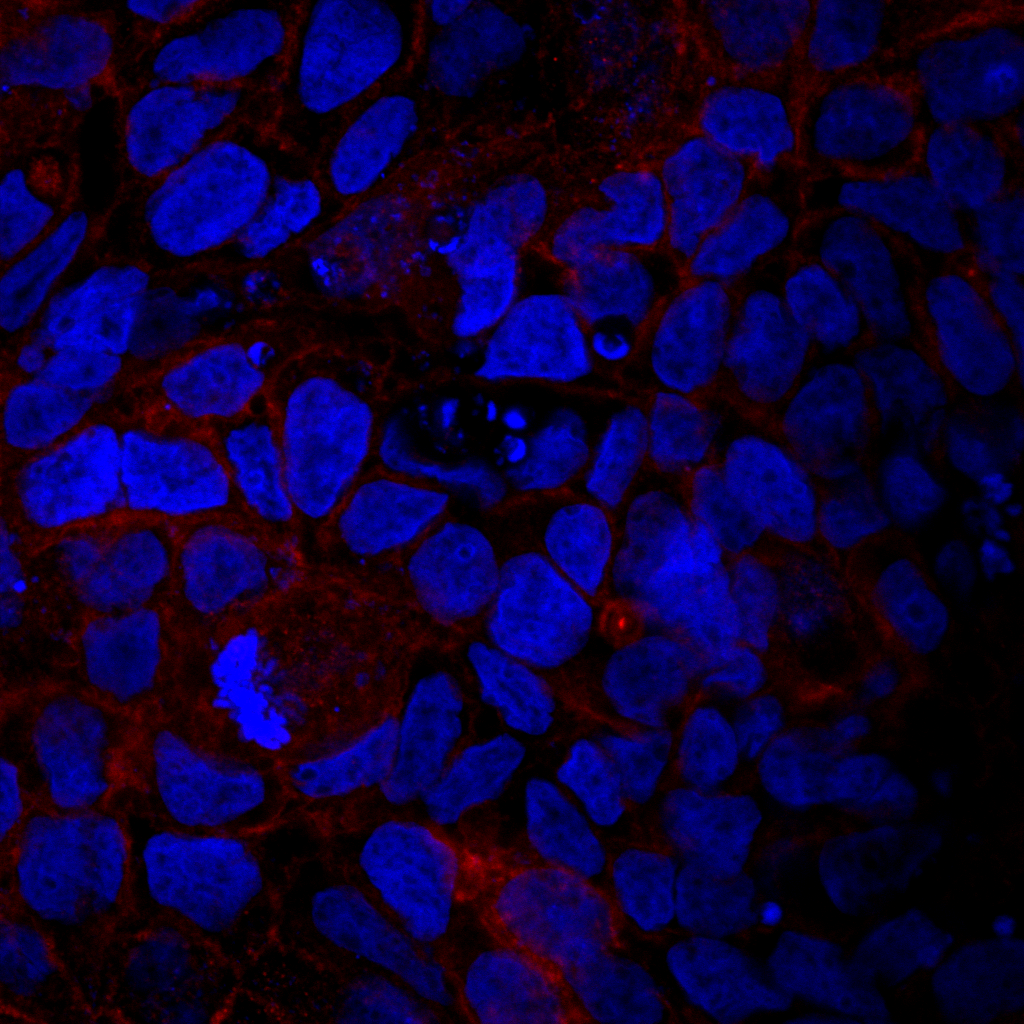

Supplement: Supplemental Information 7 [file peerj-12-16692-s007.zip › original data-figure 3/3F/miR-138 inhi/4.Claudin-1.tif]

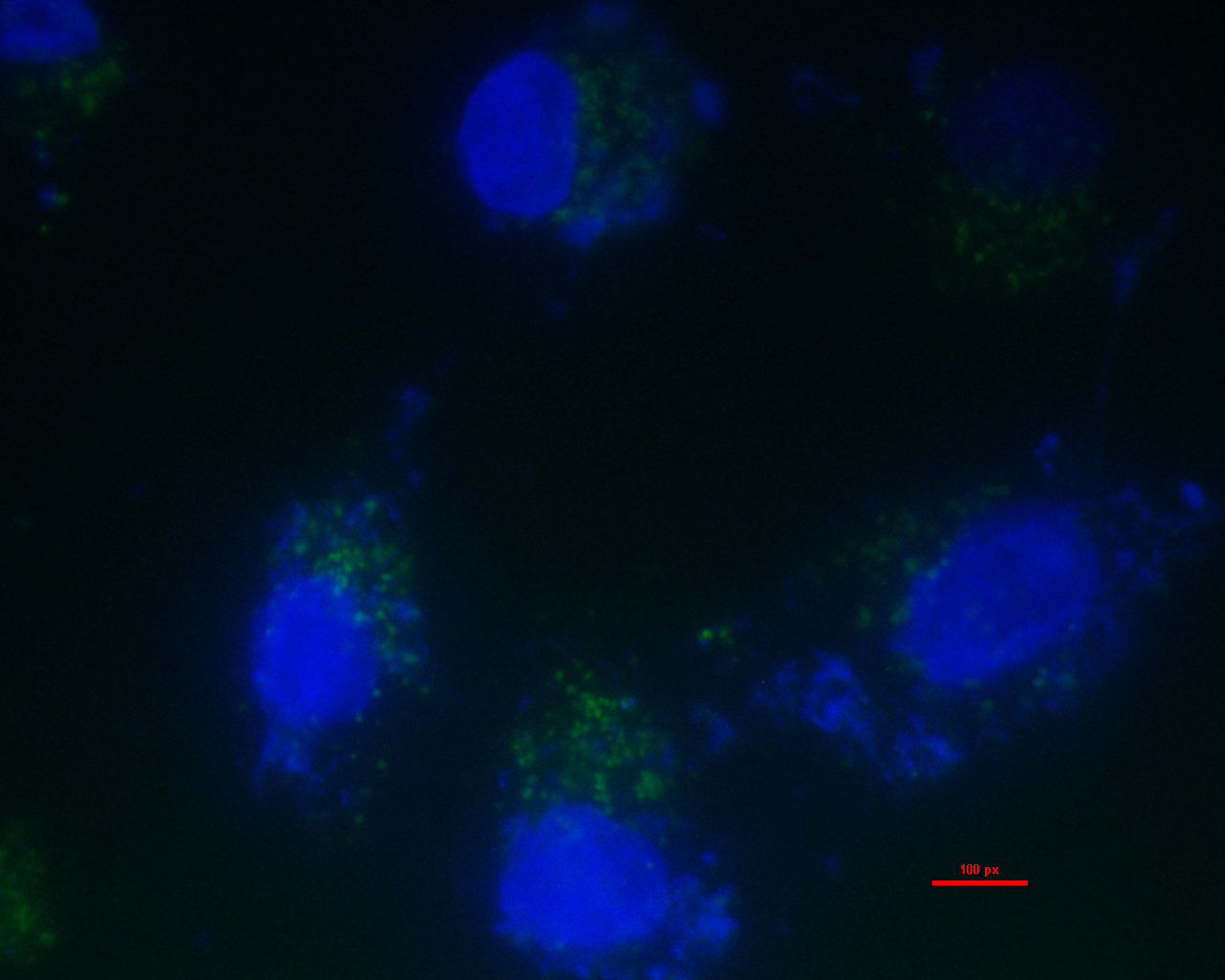

Supplement: Supplemental Information 7 [file peerj-12-16692-s007.zip › original data-figure 3/3F/miR-NC inhi/1.NLRP3.tif]

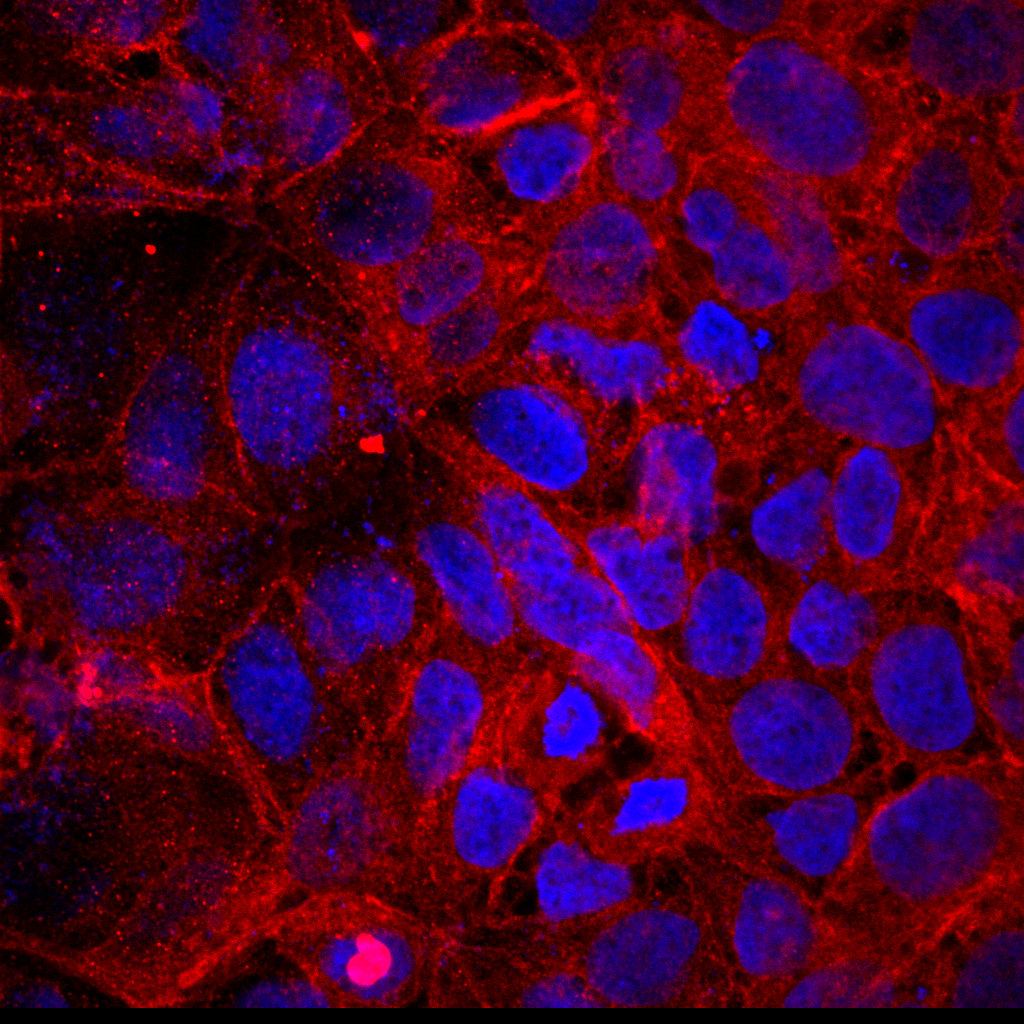

Supplement: Supplemental Information 7 [file peerj-12-16692-s007.zip › original data-figure 3/3F/miR-NC inhi/2.ZO-1.tif]

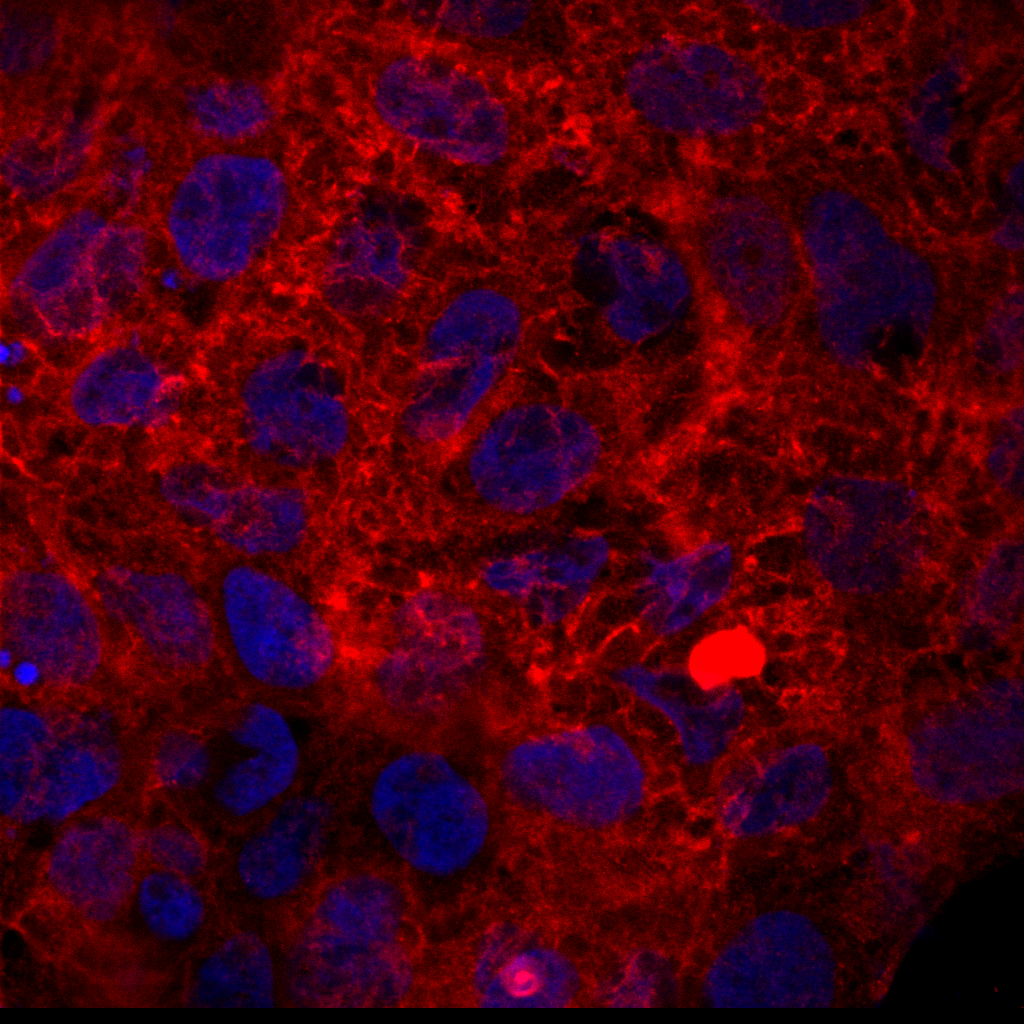

Supplement: Supplemental Information 7 [file peerj-12-16692-s007.zip › original data-figure 3/3F/miR-NC inhi/3.Occludin.tif]

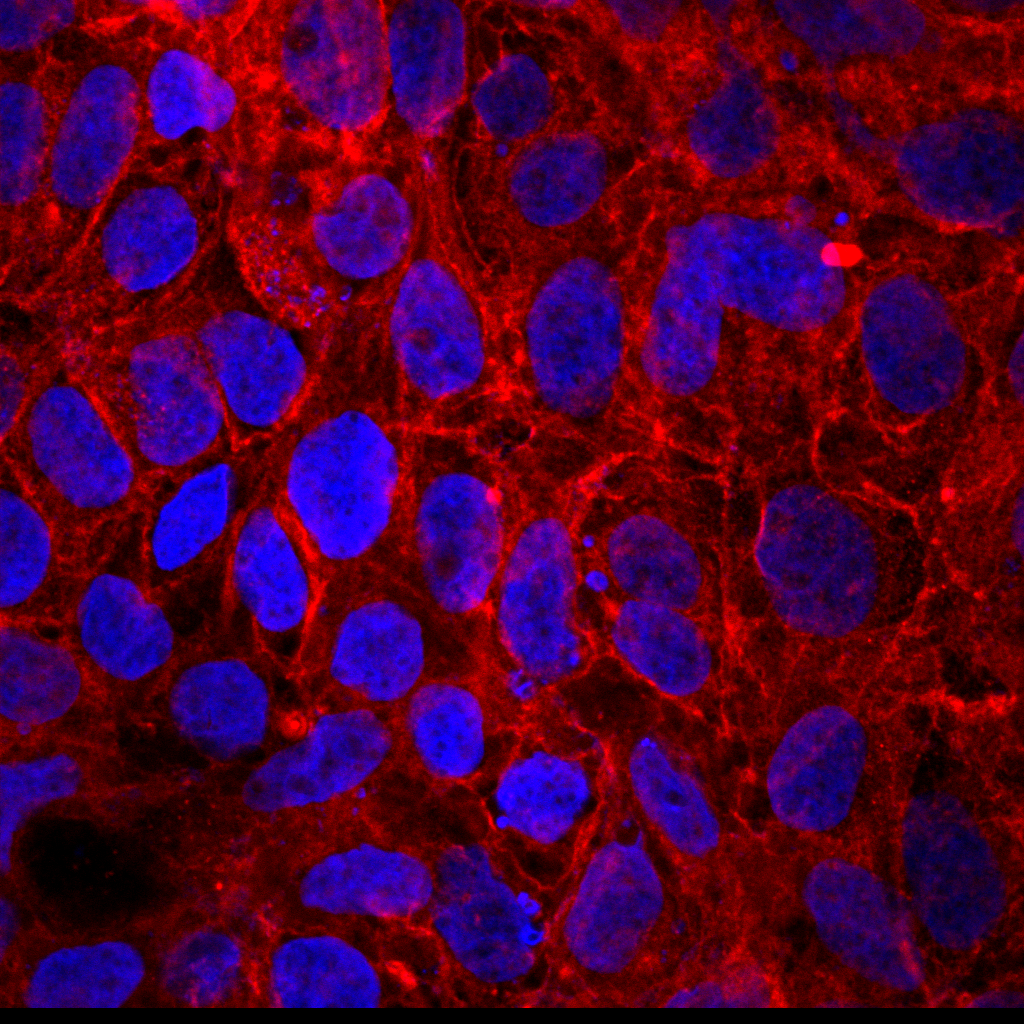

Supplement: Supplemental Information 7 [file peerj-12-16692-s007.zip › original data-figure 3/3F/miR-NC inhi/4.Claudin-1.tif]

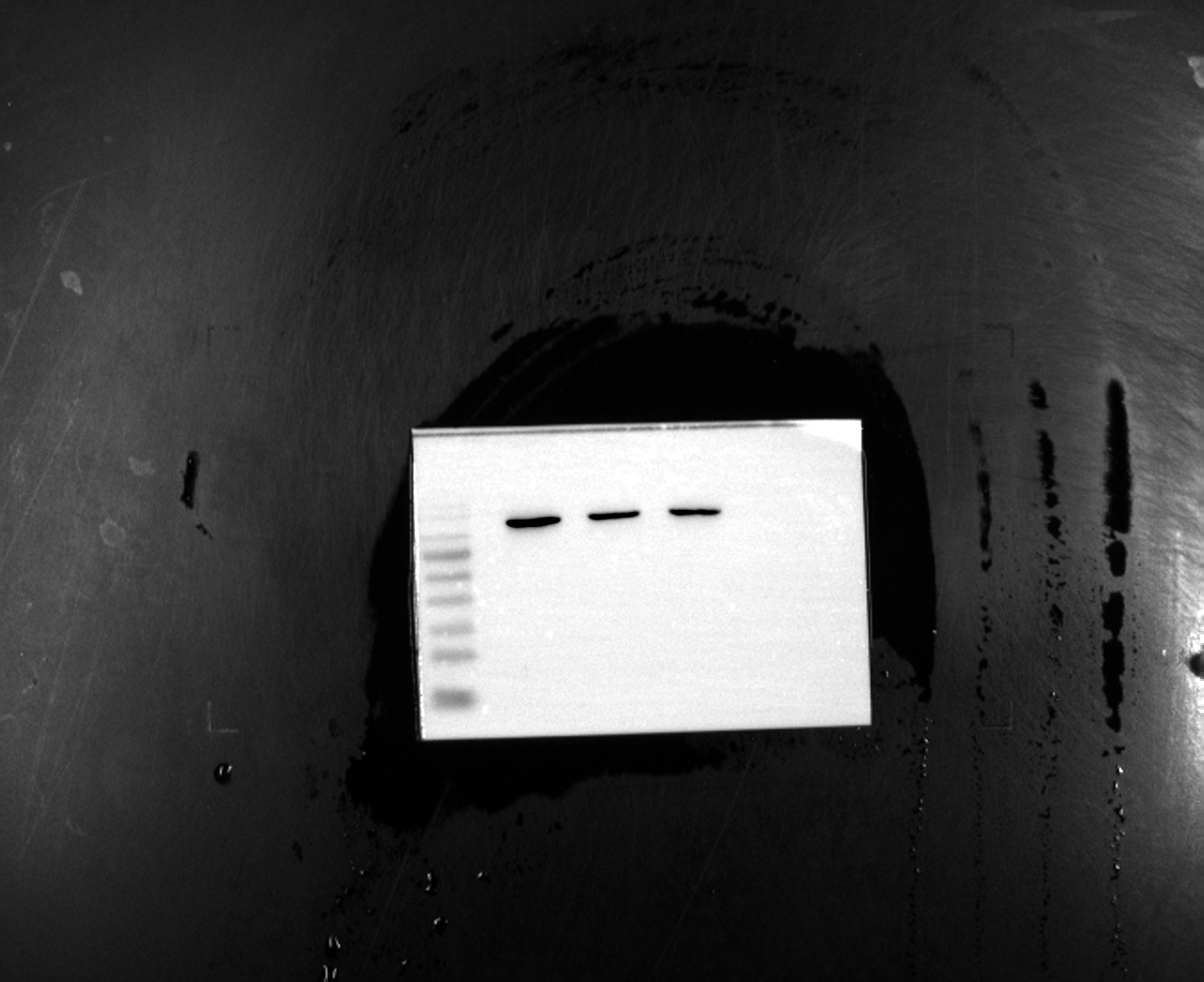

Supplement: Supplemental Information 8 [file peerj-12-16692-s008.zip › original data-figure 4-1/4A/1.NLRP3.tif]

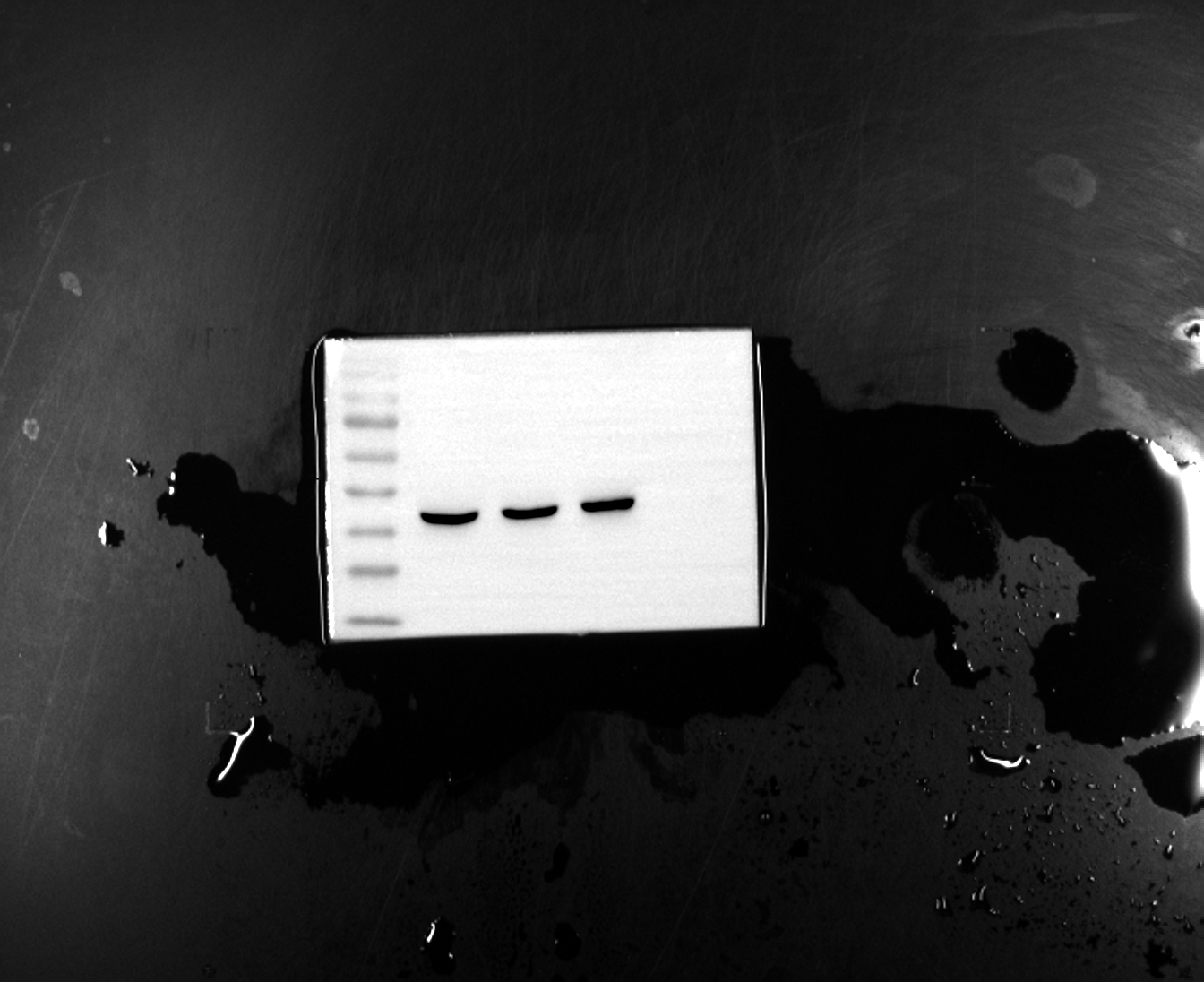

Supplement: Supplemental Information 8 [file peerj-12-16692-s008.zip › original data-figure 4-1/4A/2.GAPDH.tif]

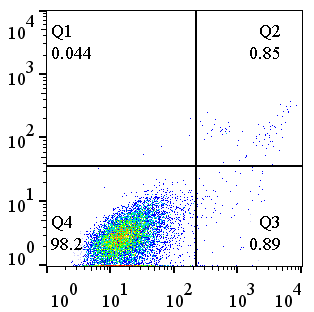

Supplement: Supplemental Information 8 [file peerj-12-16692-s008.zip › original data-figure 4-1/4C/NC.png]

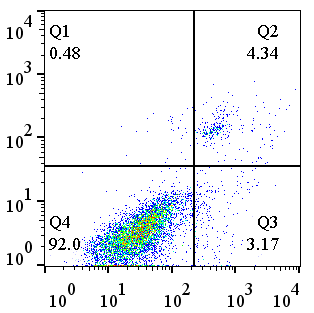

Supplement: Supplemental Information 8 [file peerj-12-16692-s008.zip › original data-figure 4-1/4C/miR-138 inh+si-NC.png]

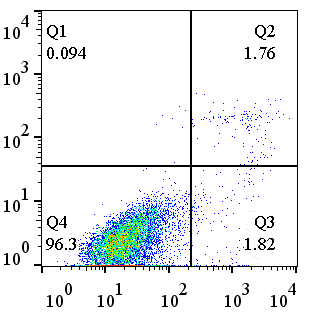

Supplement: Supplemental Information 8 [file peerj-12-16692-s008.zip › original data-figure 4-1/4C/miR-138 inh+si-NLRP3.png]

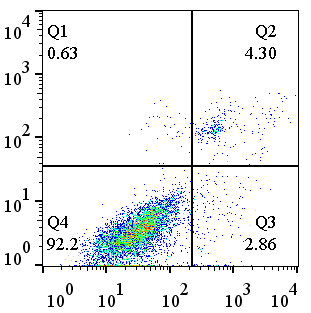

Supplement: Supplemental Information 8 [file peerj-12-16692-s008.zip › original data-figure 4-1/4C/miR-138 inh.png]

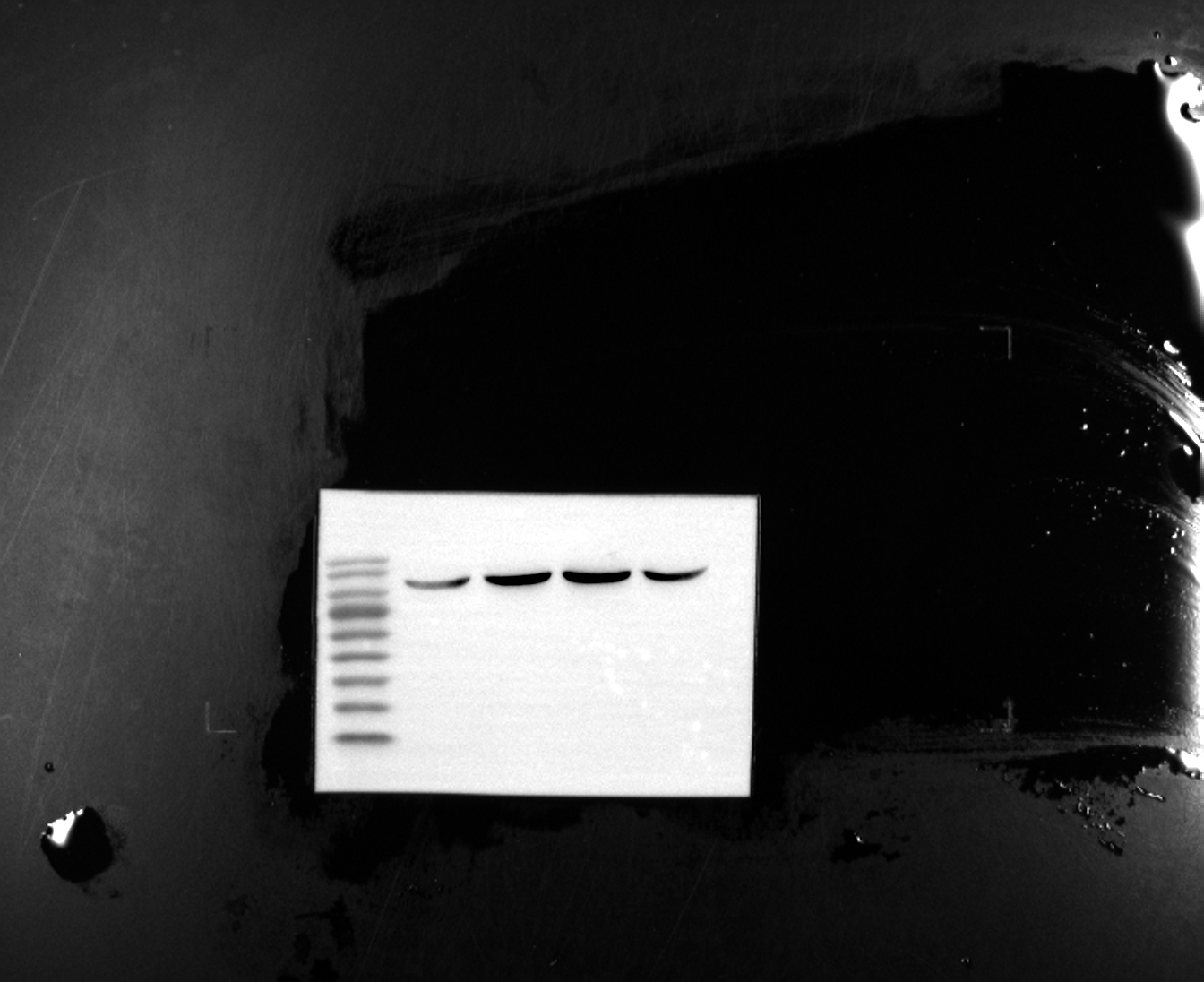

Supplement: Supplemental Information 8 [file peerj-12-16692-s008.zip › original data-figure 4-1/4D/1.NLRP3.tif]

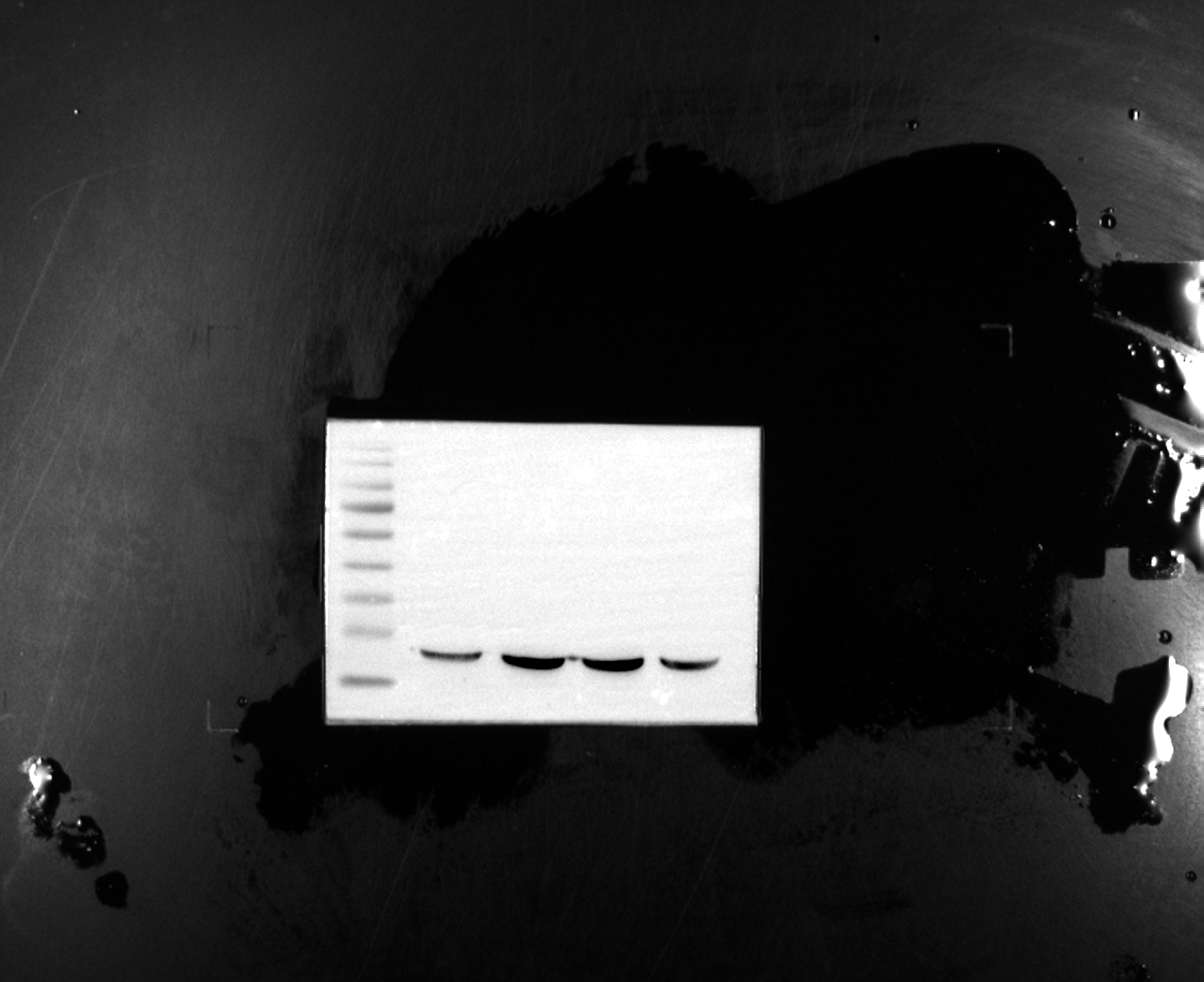

Supplement: Supplemental Information 8 [file peerj-12-16692-s008.zip › original data-figure 4-1/4D/2.ASC.tif]

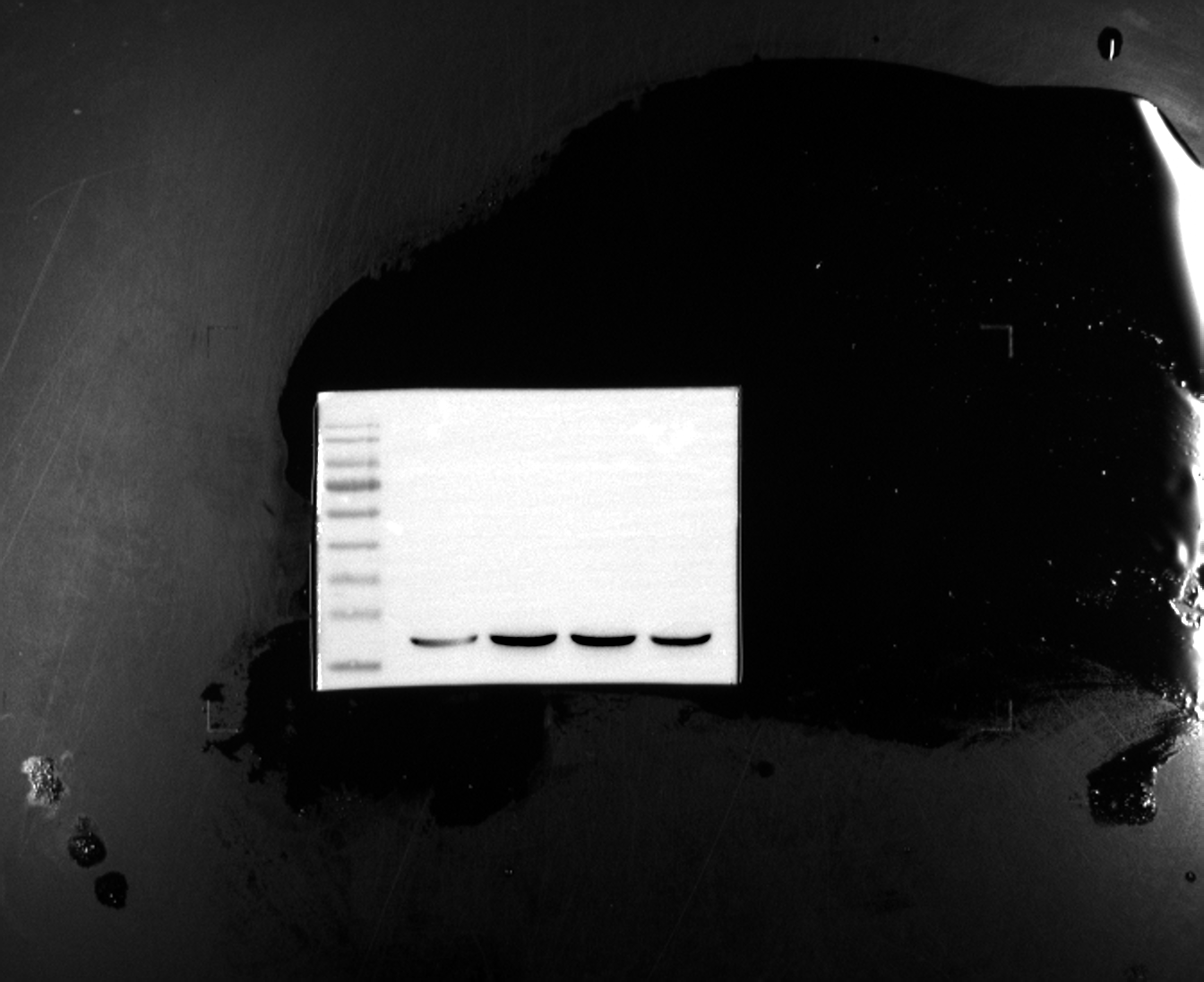

Supplement: Supplemental Information 8 [file peerj-12-16692-s008.zip › original data-figure 4-1/4D/3.Caspase 1.tif]

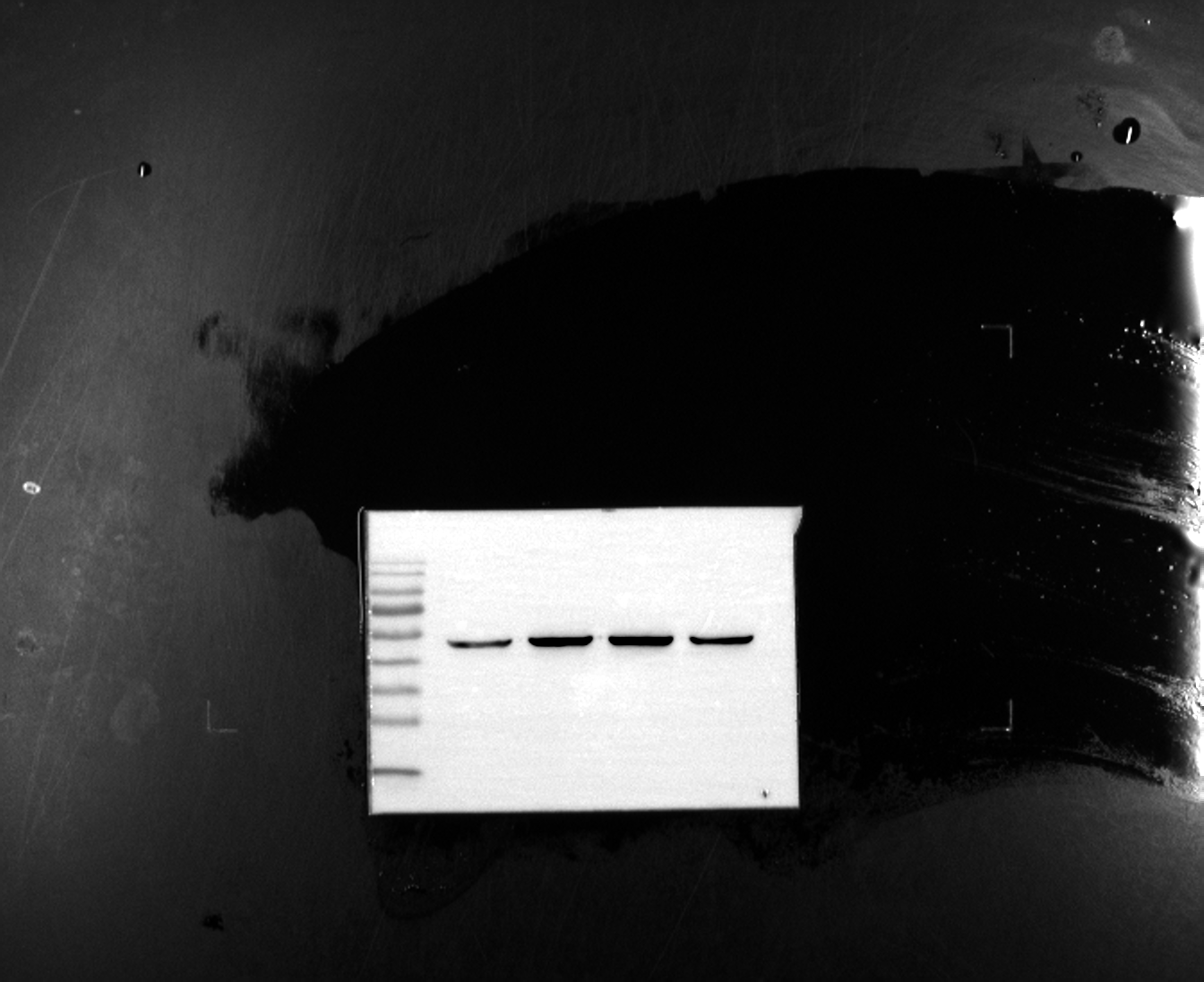

Supplement: Supplemental Information 8 [file peerj-12-16692-s008.zip › original data-figure 4-1/4D/4.GSDMD-N.tif]

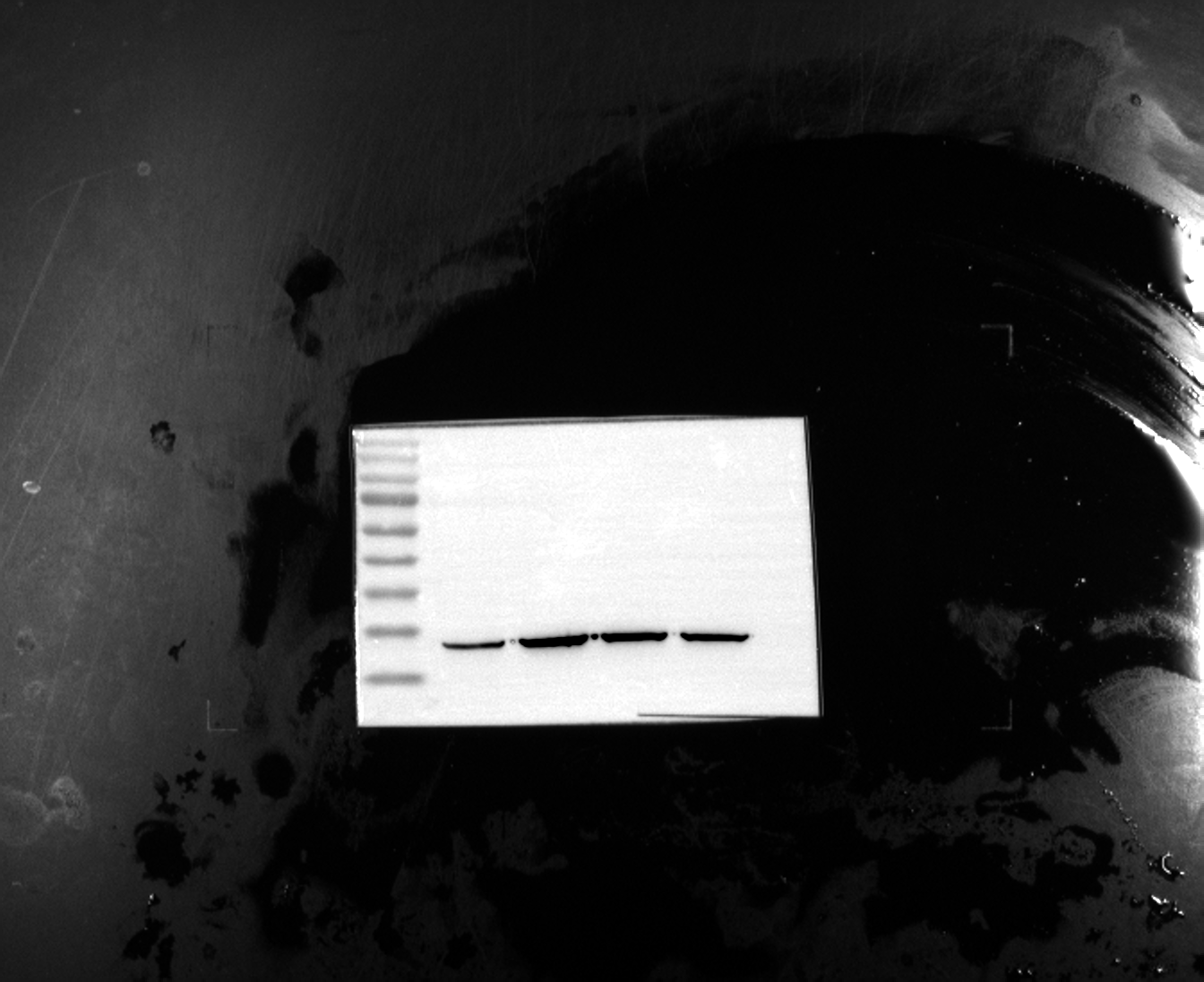

Supplement: Supplemental Information 8 [file peerj-12-16692-s008.zip › original data-figure 4-1/4D/5.IL-18.tif]

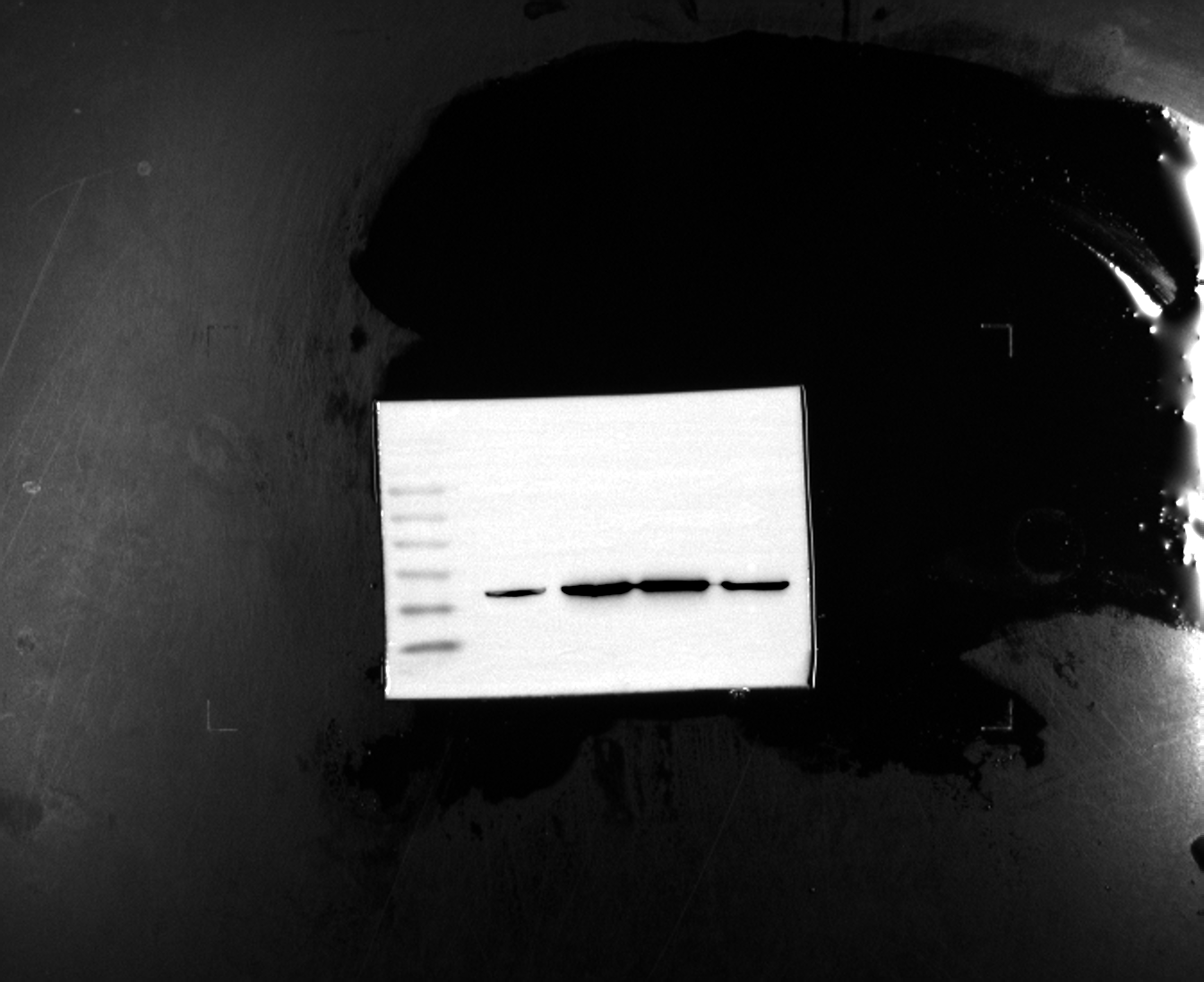

Supplement: Supplemental Information 8 [file peerj-12-16692-s008.zip › original data-figure 4-1/4D/6.IL-1β.tif]

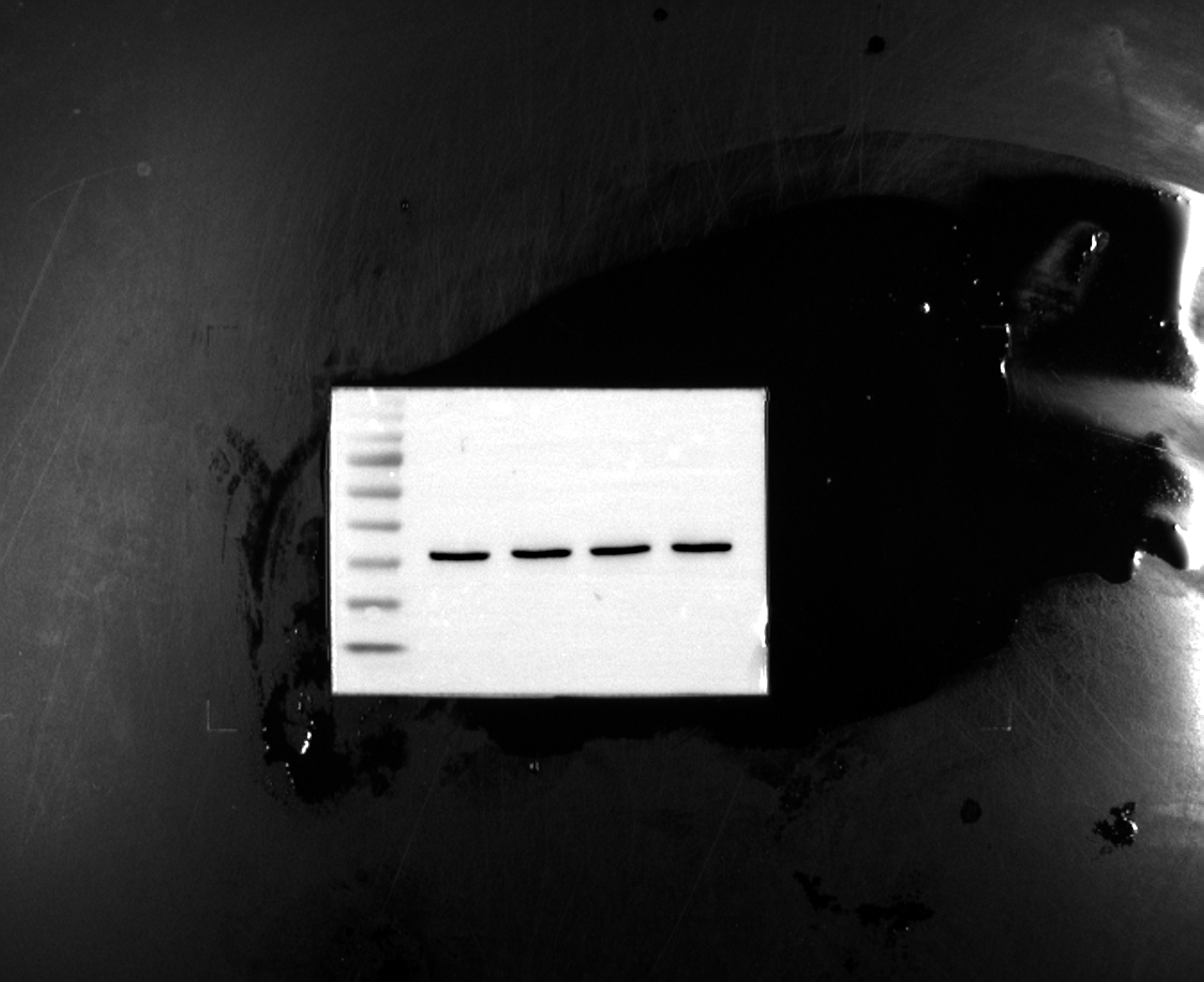

Supplement: Supplemental Information 8 [file peerj-12-16692-s008.zip › original data-figure 4-1/4D/7.GAPDH.tif]

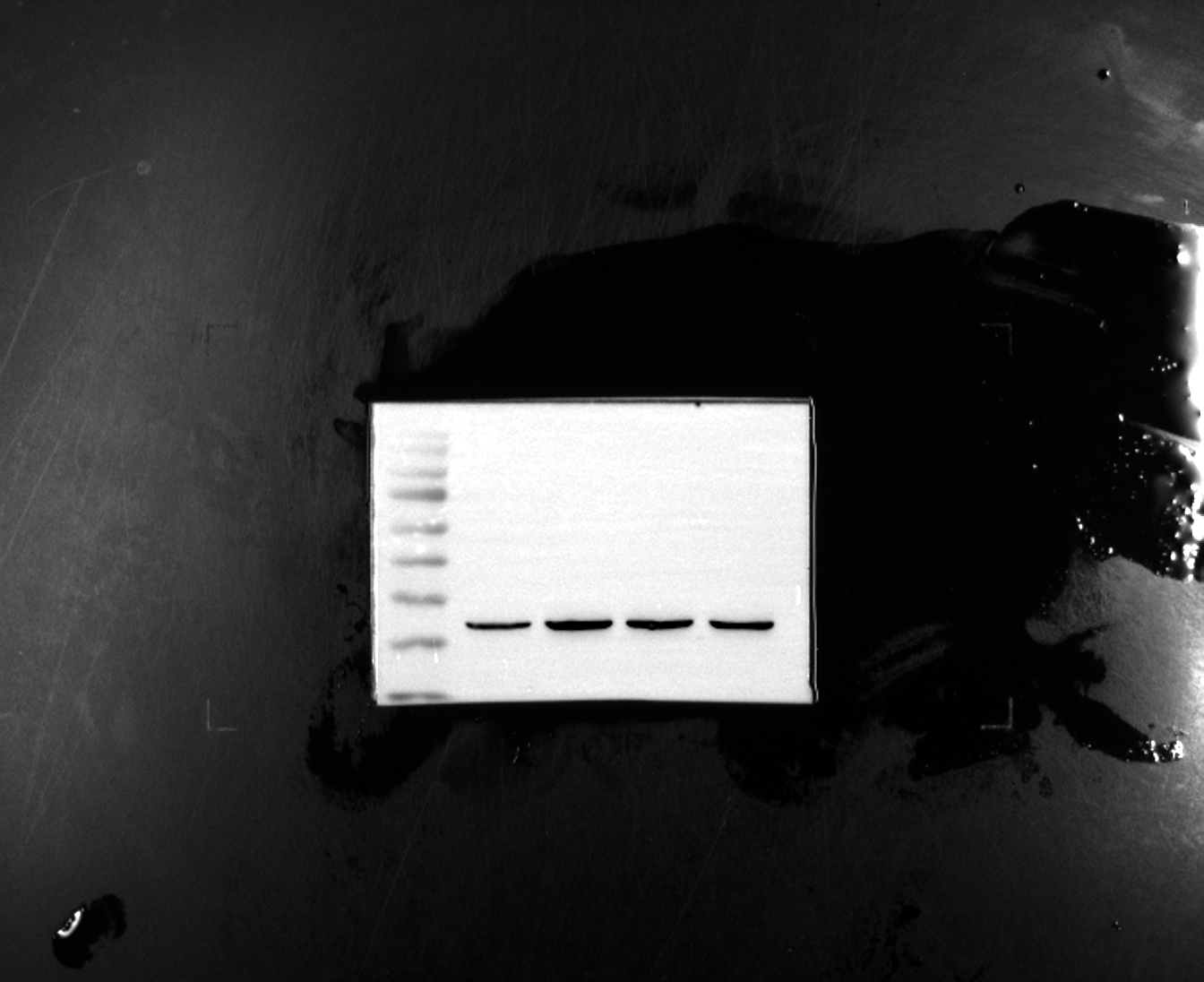

Supplement: Supplemental Information 9 [file peerj-12-16692-s009.zip › original data-figure 4-2/4E/1.HMGB1.tif]

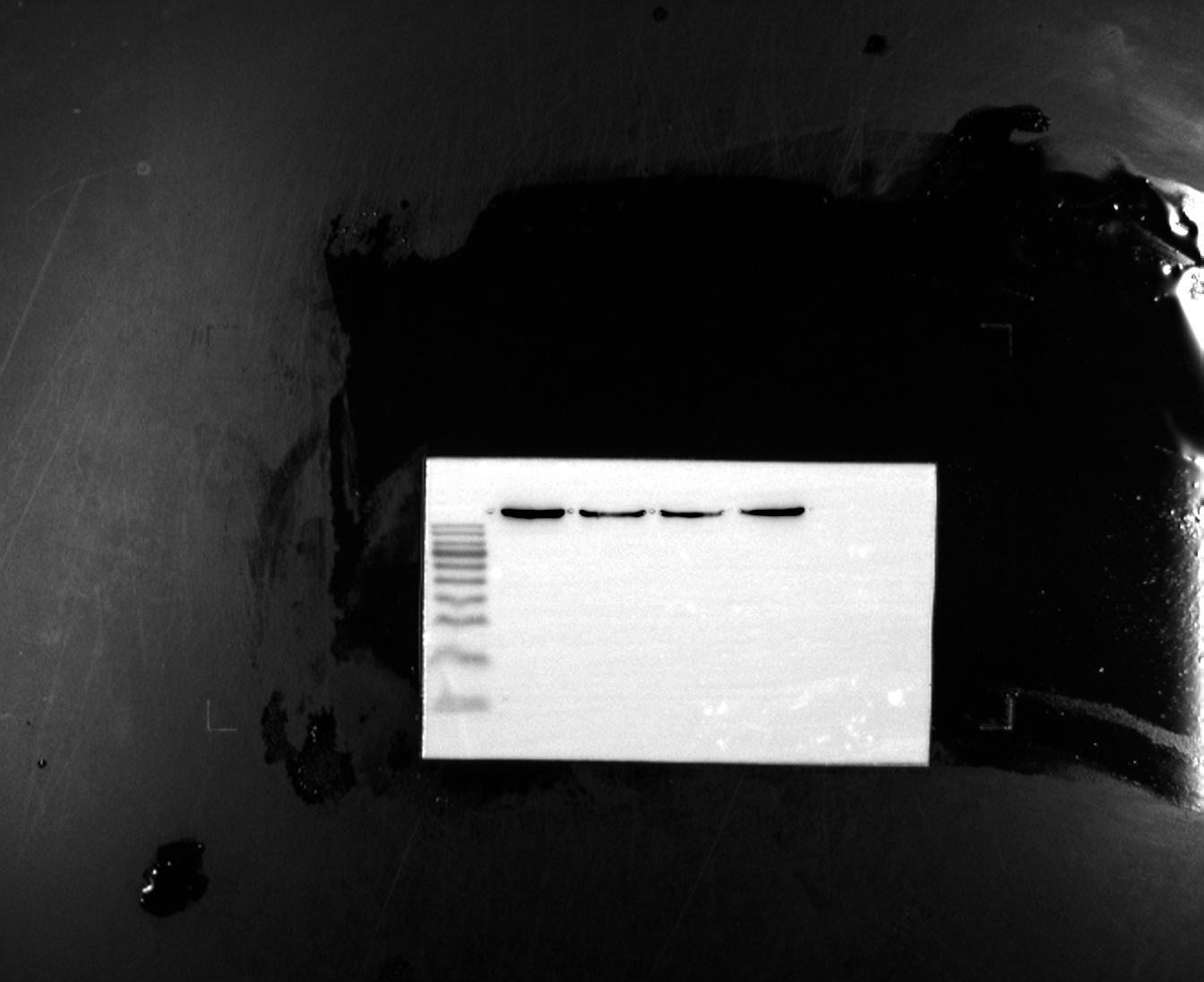

Supplement: Supplemental Information 9 [file peerj-12-16692-s009.zip › original data-figure 4-2/4E/2.ZO-1.tif]

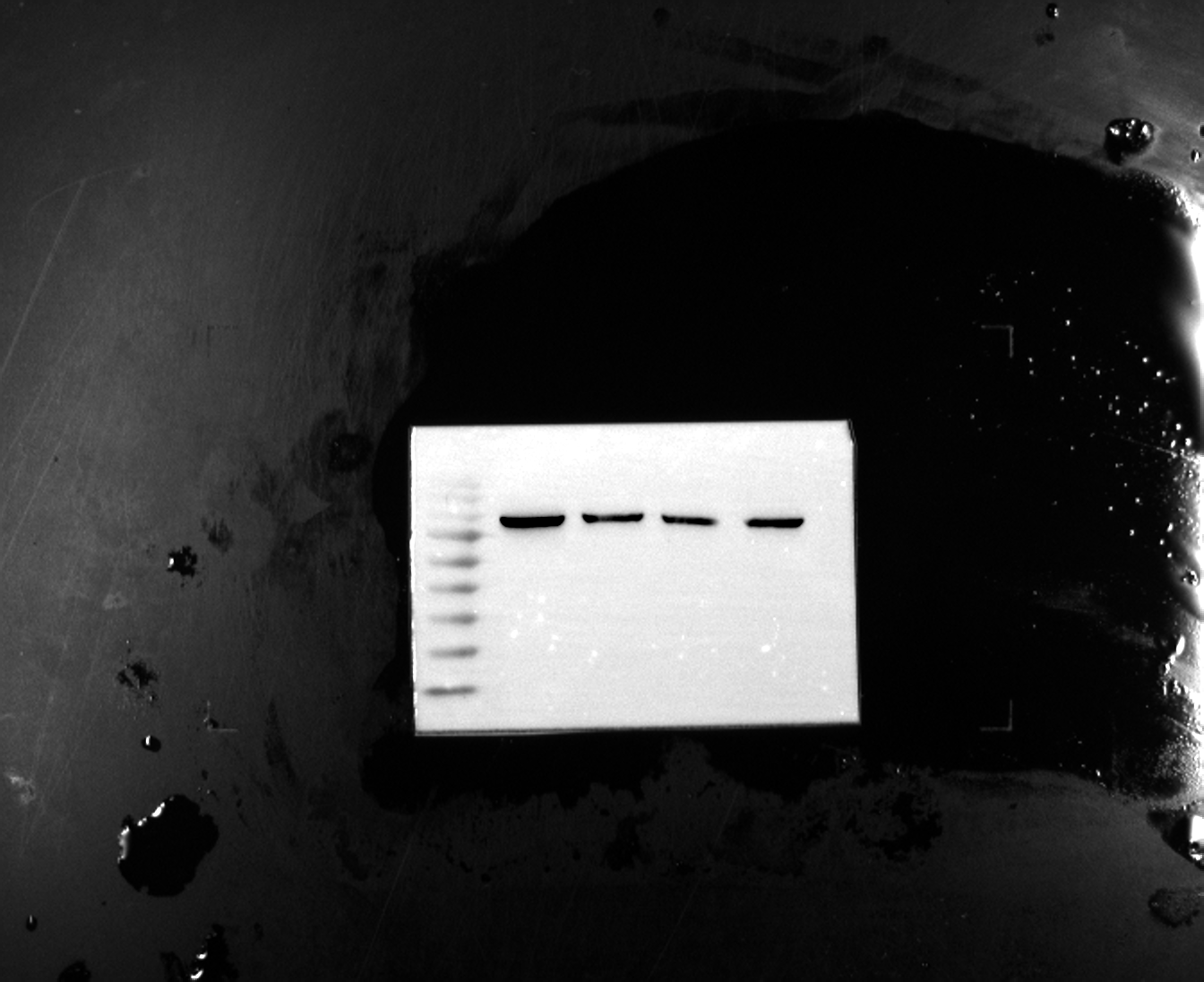

Supplement: Supplemental Information 9 [file peerj-12-16692-s009.zip › original data-figure 4-2/4E/3.Occludin.tif]

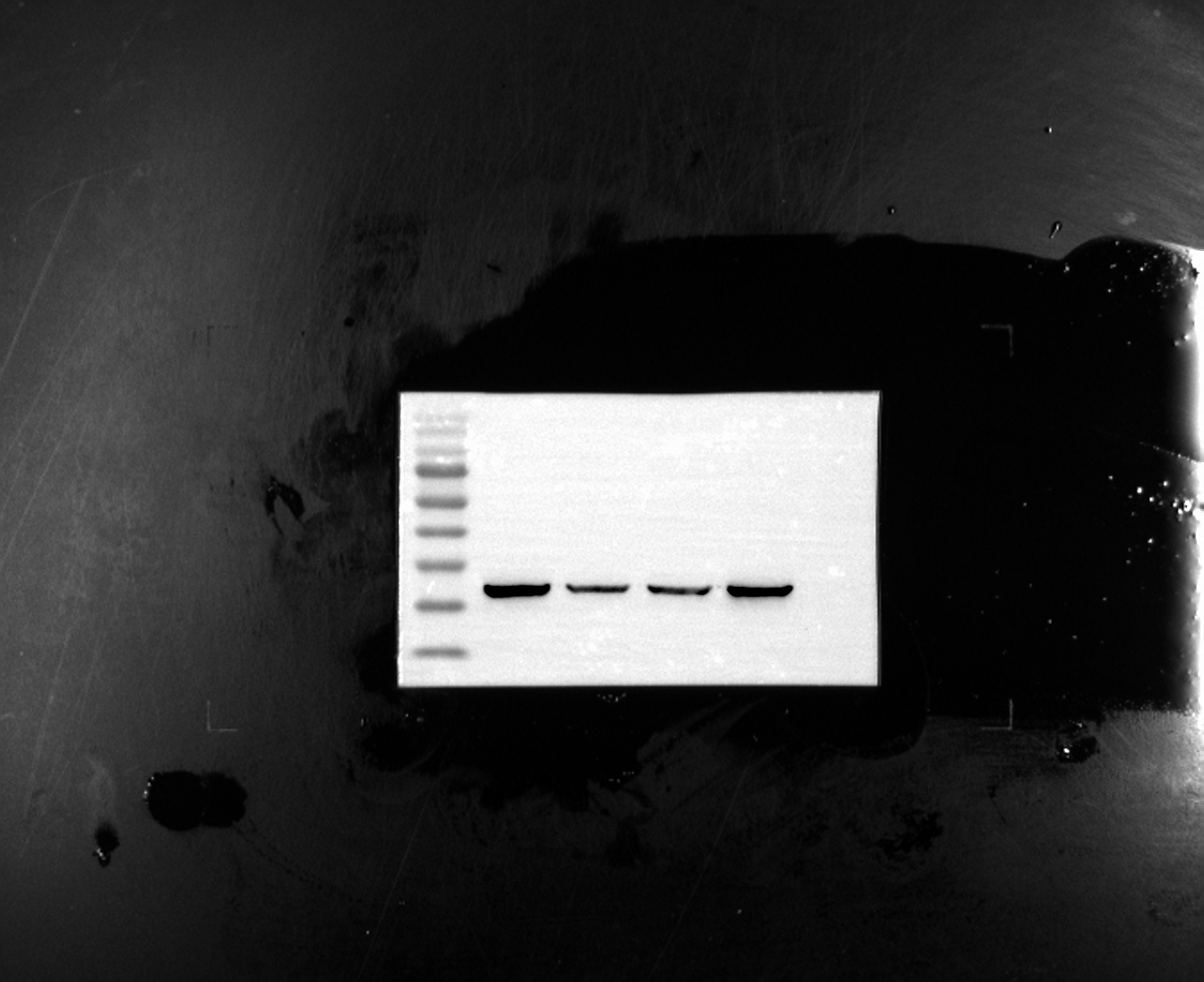

Supplement: Supplemental Information 9 [file peerj-12-16692-s009.zip › original data-figure 4-2/4E/4.Claudin-1.tif]

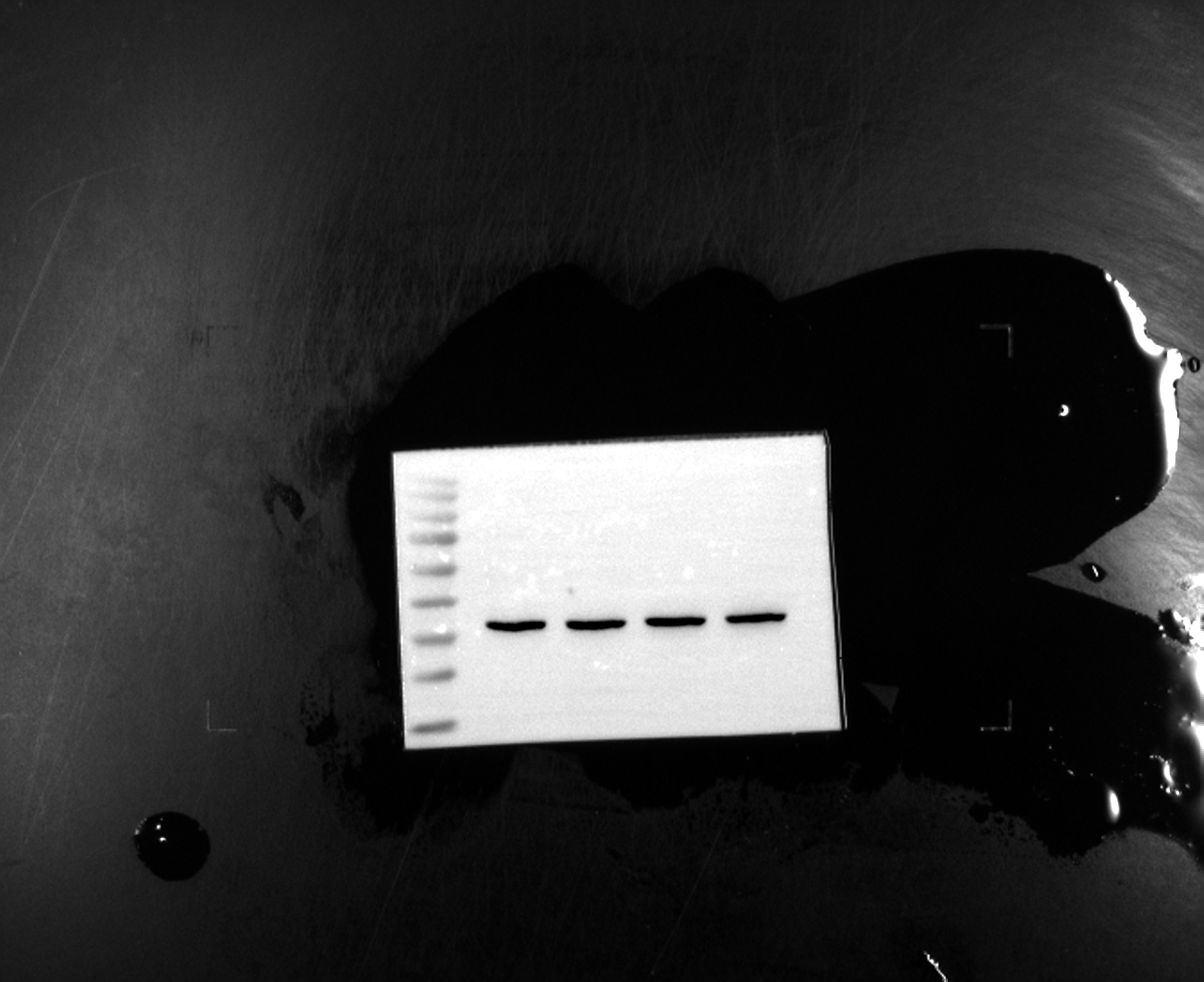

Supplement: Supplemental Information 9 [file peerj-12-16692-s009.zip › original data-figure 4-2/4E/5.GAPDH.tif]

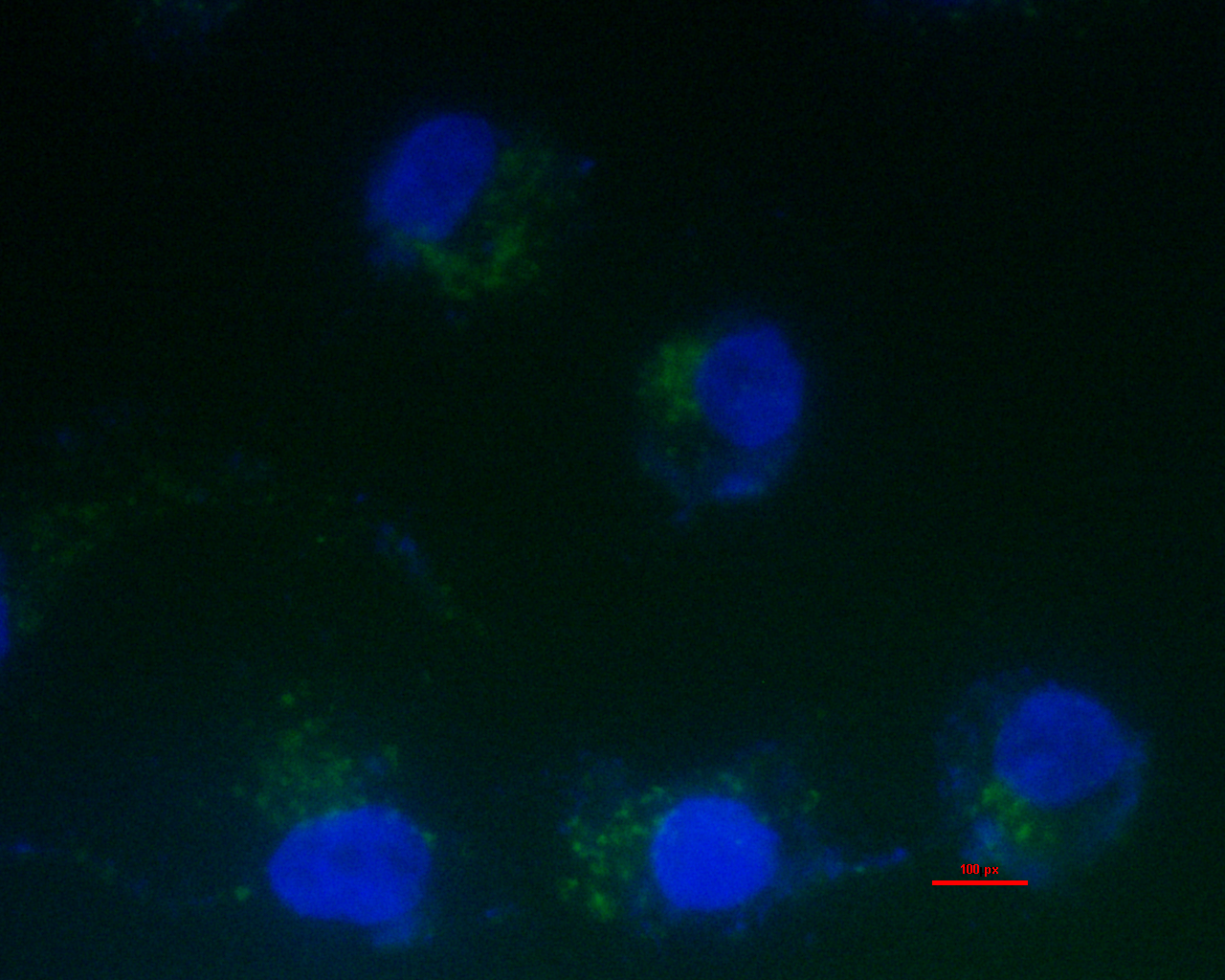

Supplement: Supplemental Information 9 [file peerj-12-16692-s009.zip › original data-figure 4-2/4F/NC/1.NLRP3.tif]

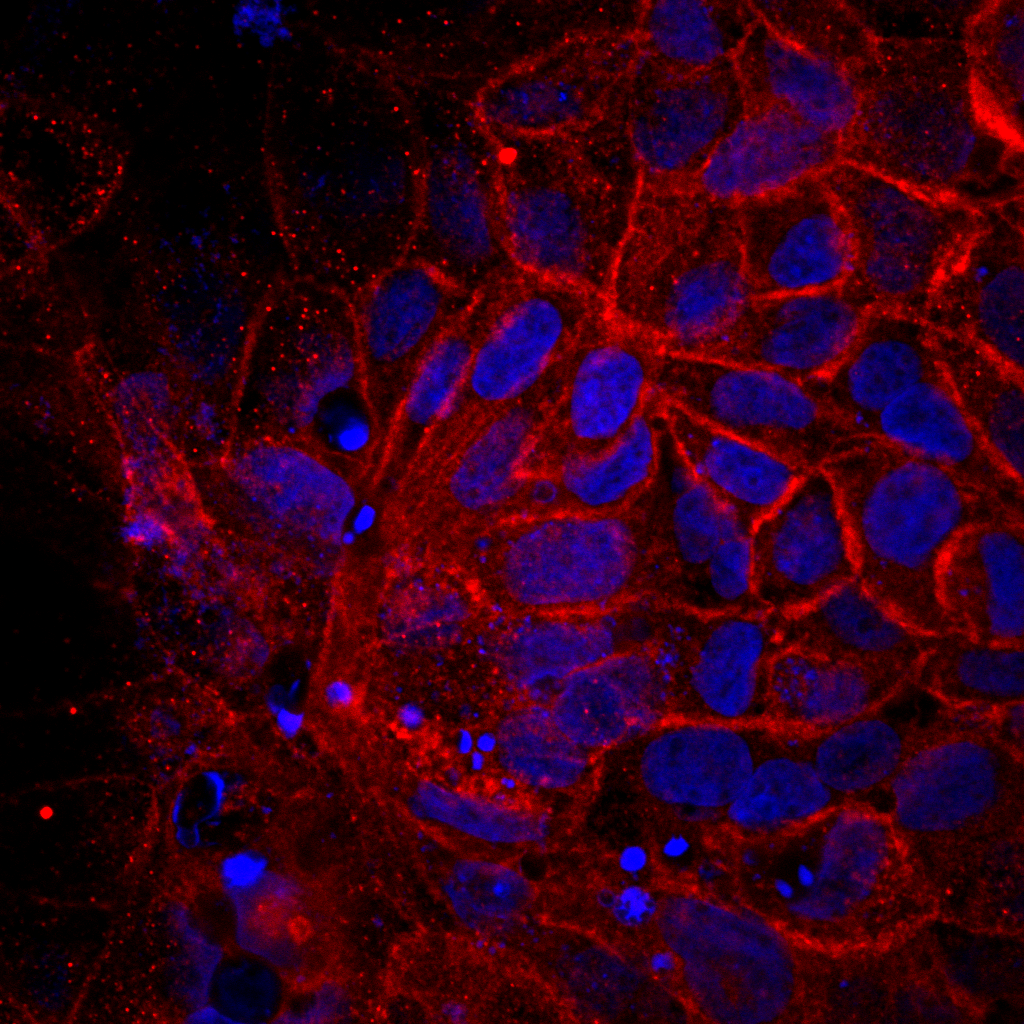

Supplement: Supplemental Information 9 [file peerj-12-16692-s009.zip › original data-figure 4-2/4F/NC/2.ZO-1.tif]

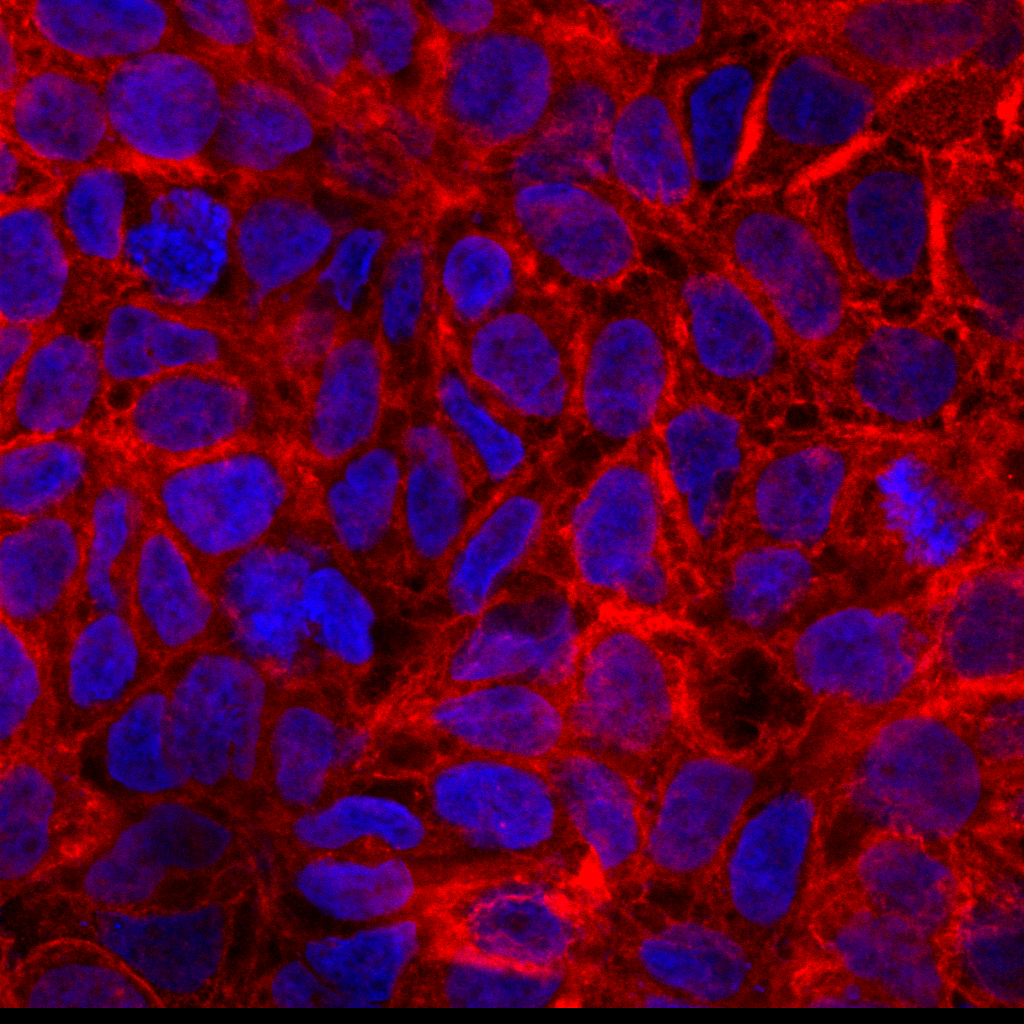

Supplement: Supplemental Information 9 [file peerj-12-16692-s009.zip › original data-figure 4-2/4F/NC/3.Occludin.tif]

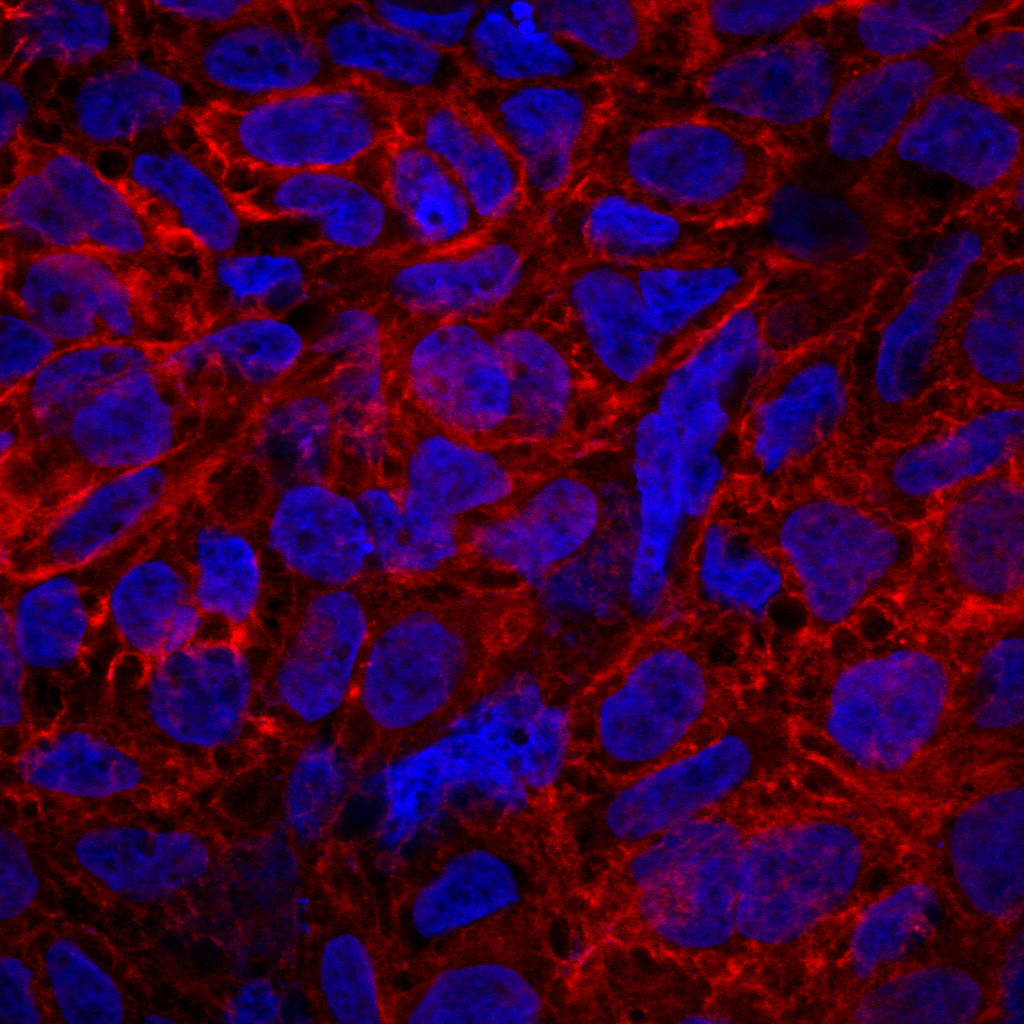

Supplement: Supplemental Information 9 [file peerj-12-16692-s009.zip › original data-figure 4-2/4F/NC/4.Claudin-1.tif]

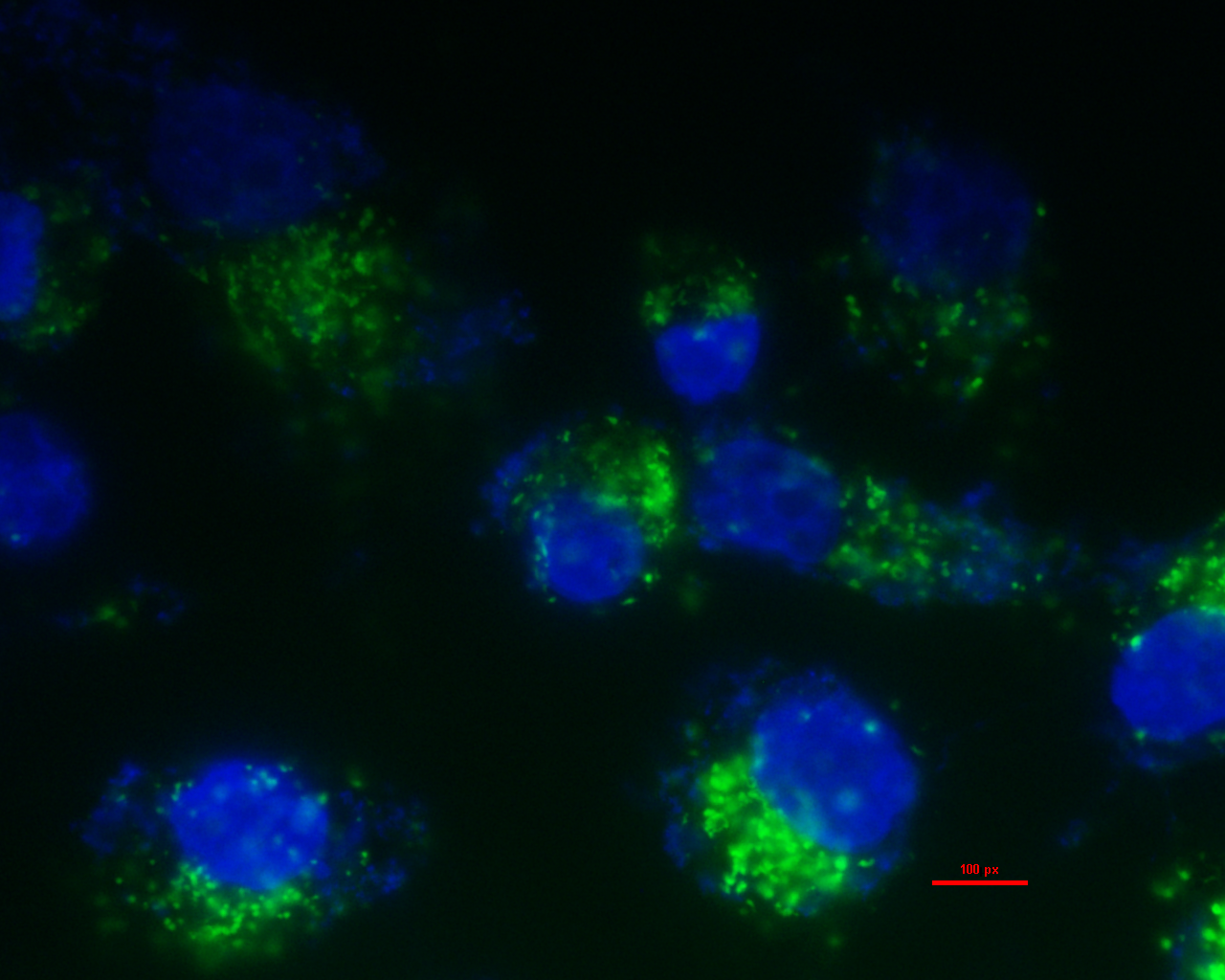

Supplement: Supplemental Information 9 [file peerj-12-16692-s009.zip › original data-figure 4-2/4F/miR-138 inhi/1.NLRP3.tif]

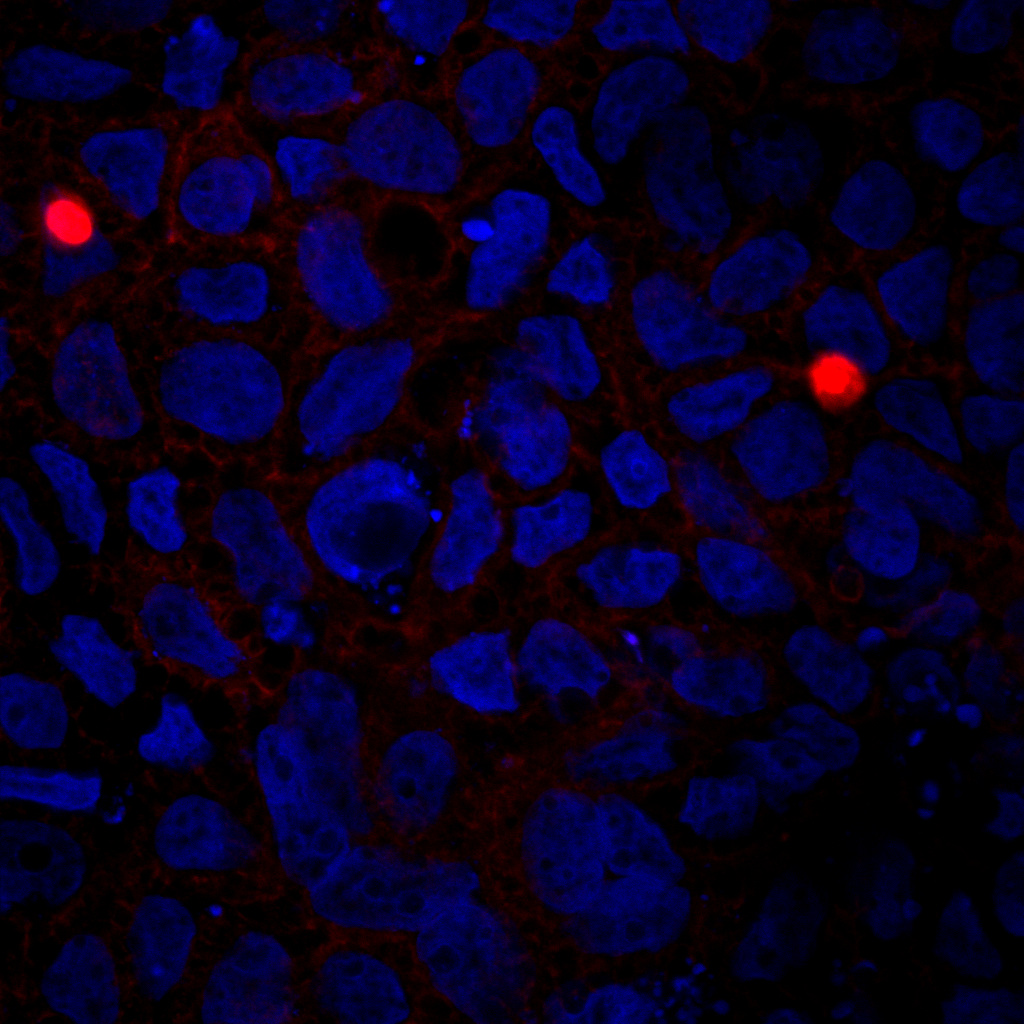

Supplement: Supplemental Information 9 [file peerj-12-16692-s009.zip › original data-figure 4-2/4F/miR-138 inhi/2.ZO-1.tif]

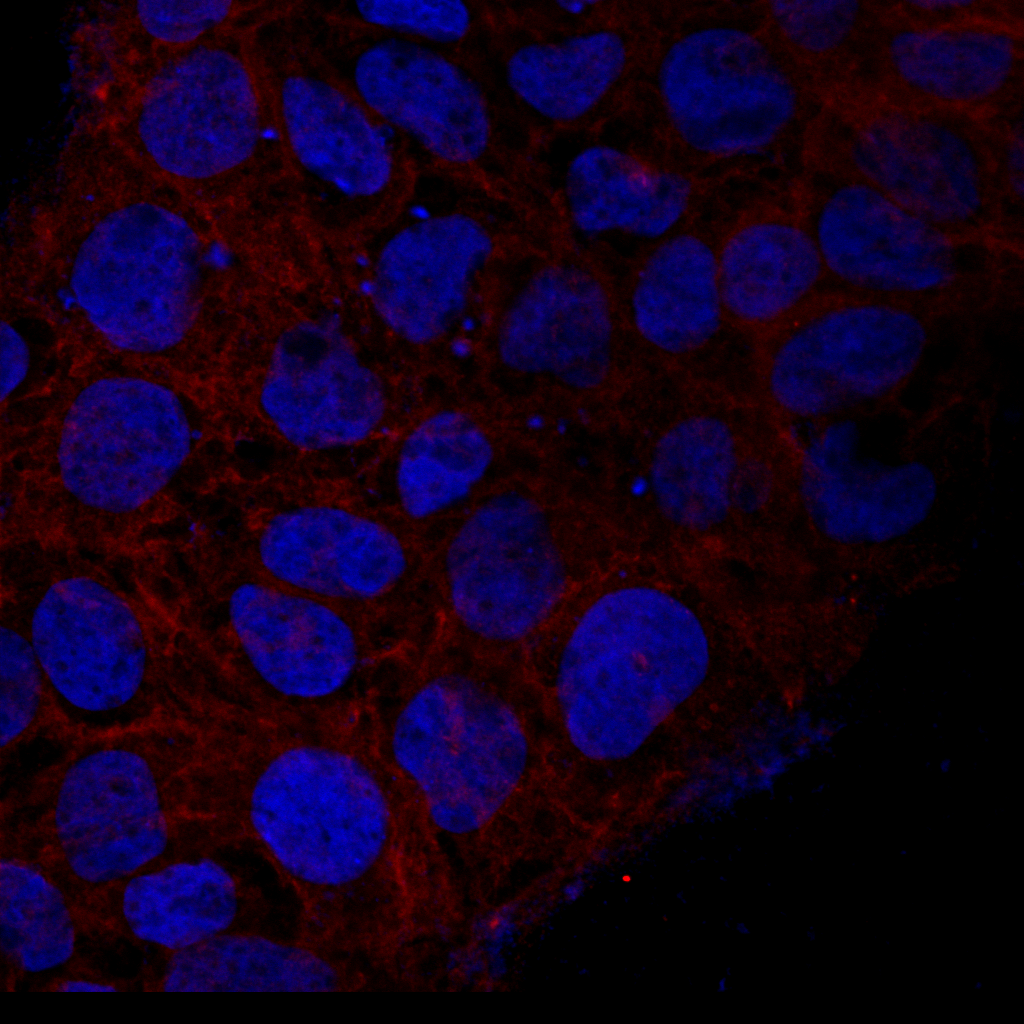

Supplement: Supplemental Information 9 [file peerj-12-16692-s009.zip › original data-figure 4-2/4F/miR-138 inhi/3.Occludin.tif]

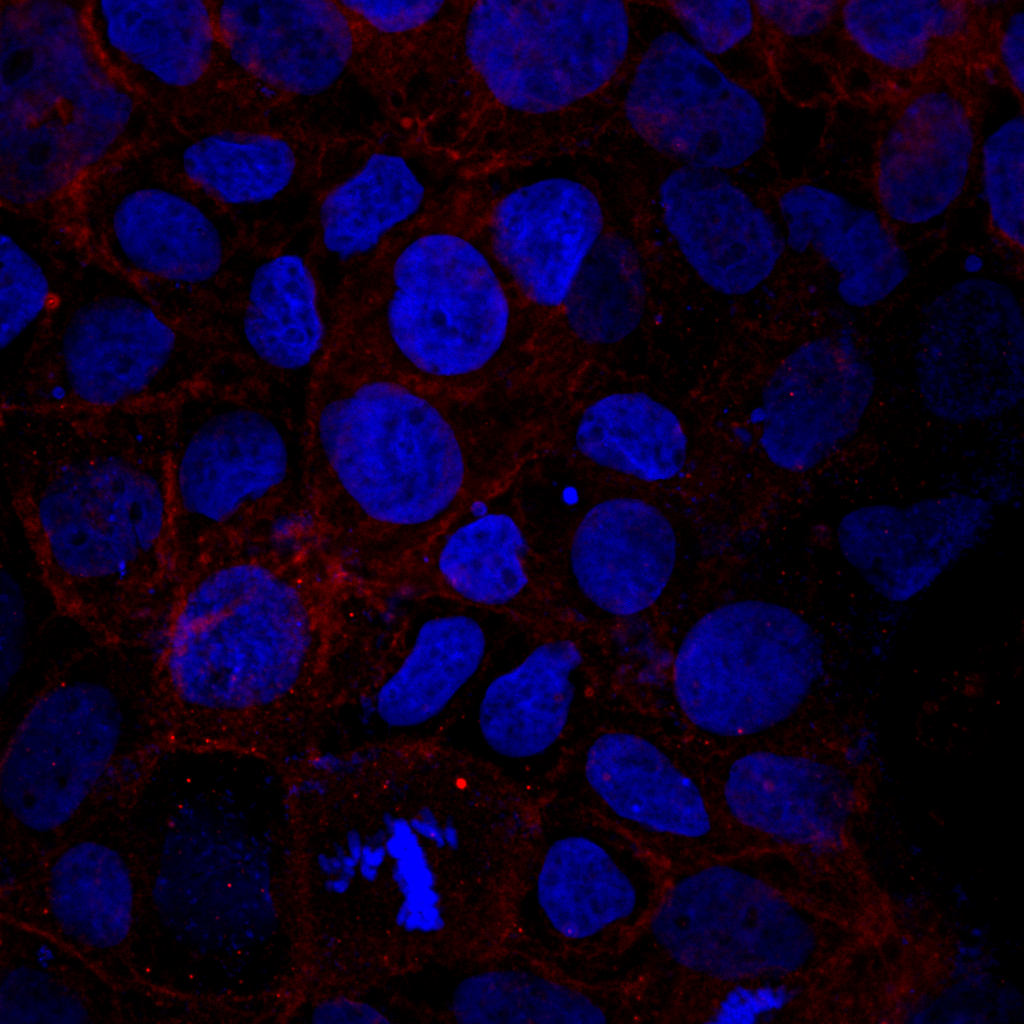

Supplement: Supplemental Information 9 [file peerj-12-16692-s009.zip › original data-figure 4-2/4F/miR-138 inhi/4.Claudin-1.tif]

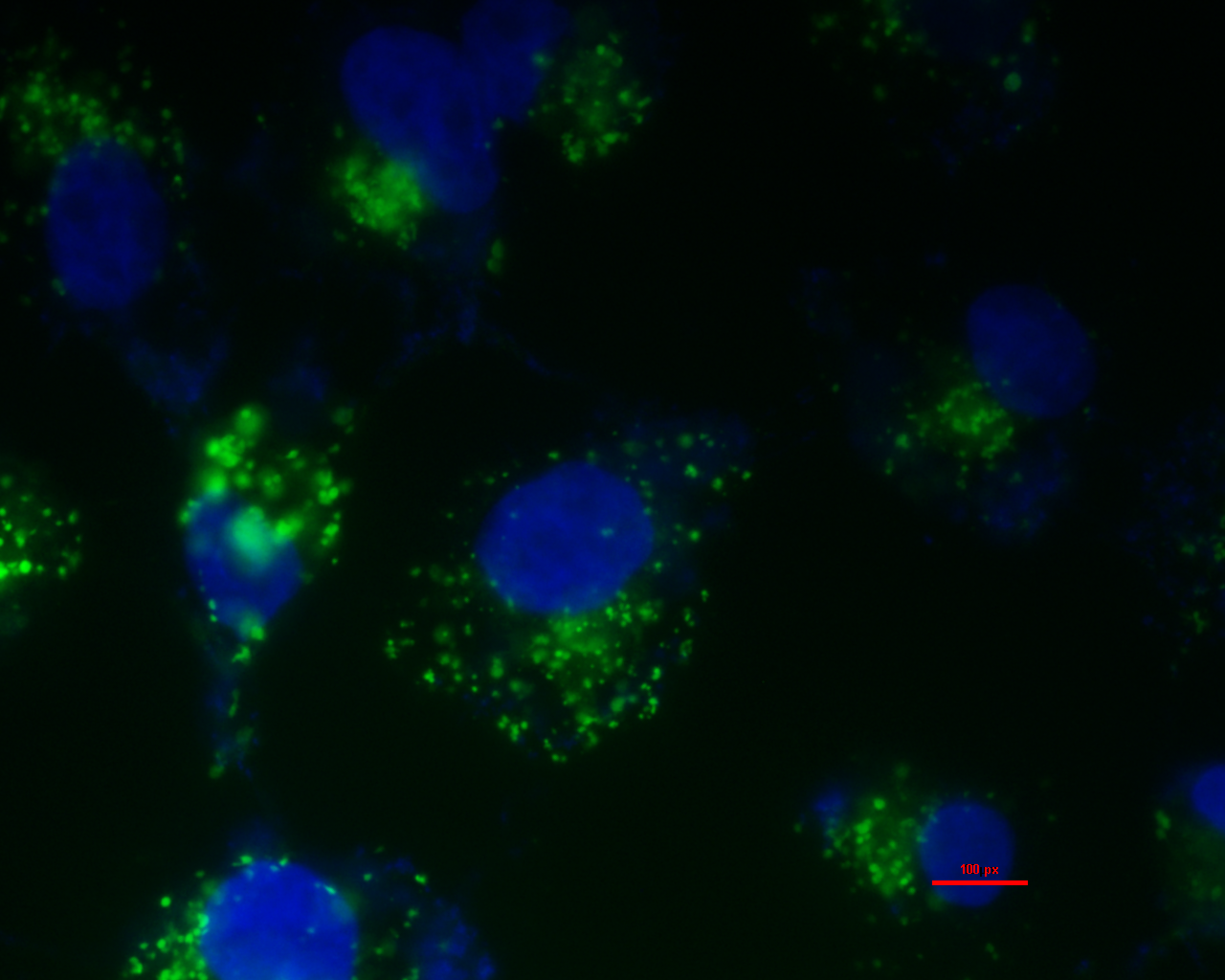

Supplement: Supplemental Information 9 [file peerj-12-16692-s009.zip › original data-figure 4-2/4F/miR-138 inhi+si-NC/1.NLRP3.tif]

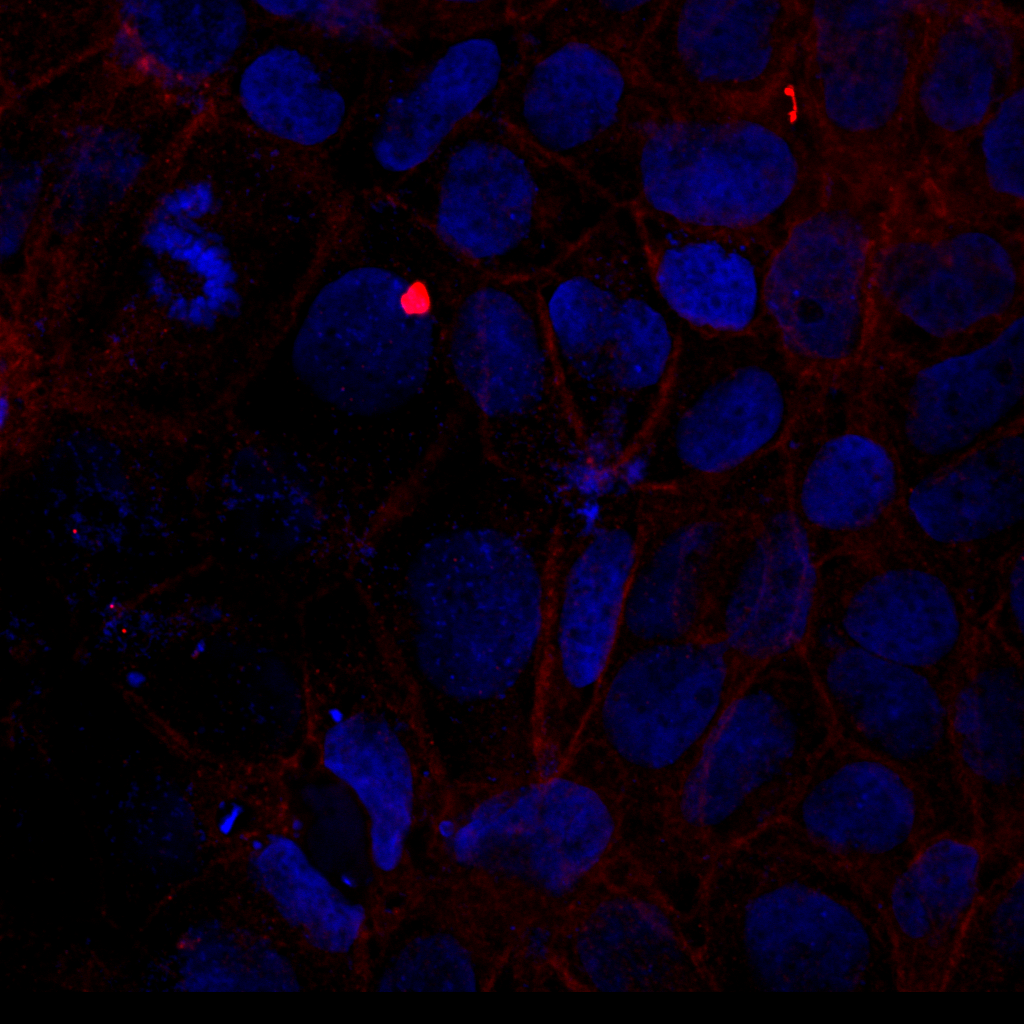

Supplement: Supplemental Information 9 [file peerj-12-16692-s009.zip › original data-figure 4-2/4F/miR-138 inhi+si-NC/2.ZO-1.tif]

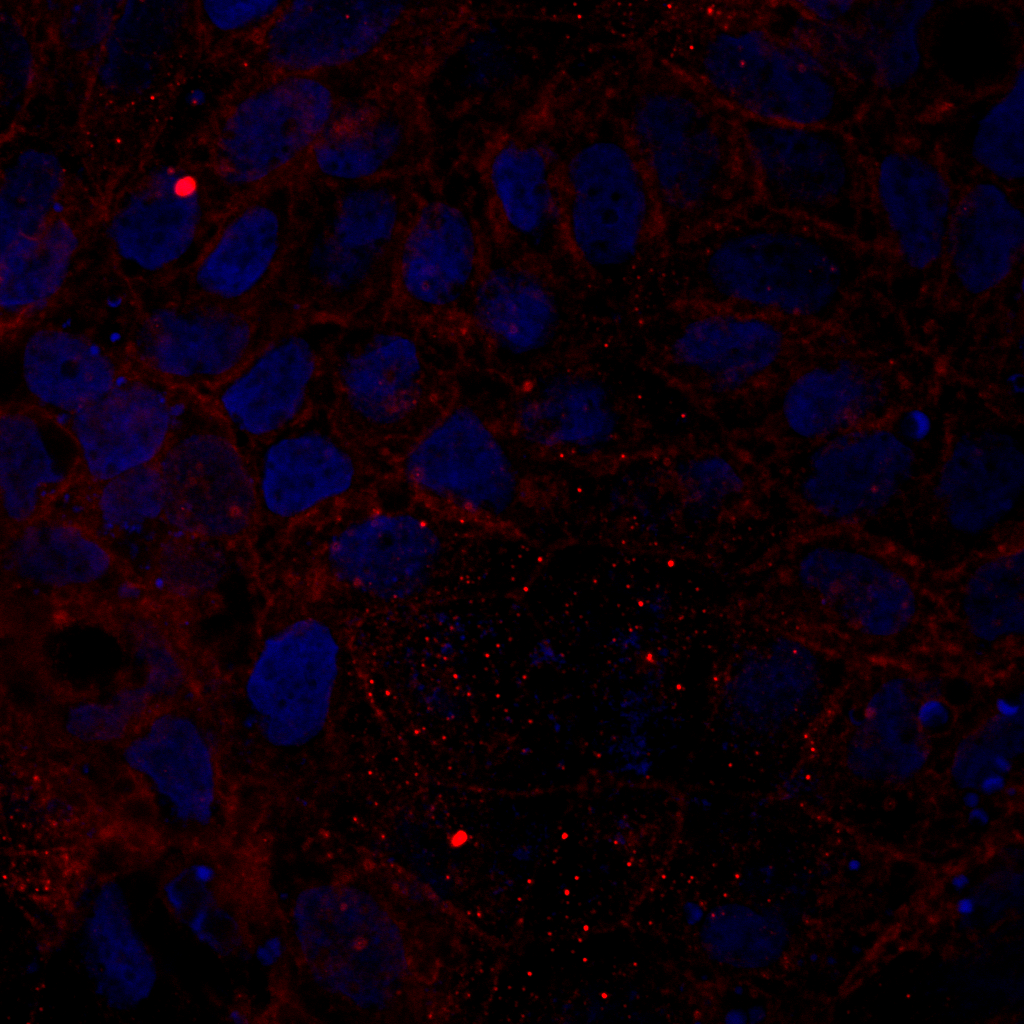

Supplement: Supplemental Information 9 [file peerj-12-16692-s009.zip › original data-figure 4-2/4F/miR-138 inhi+si-NC/3.Occludin.tif]

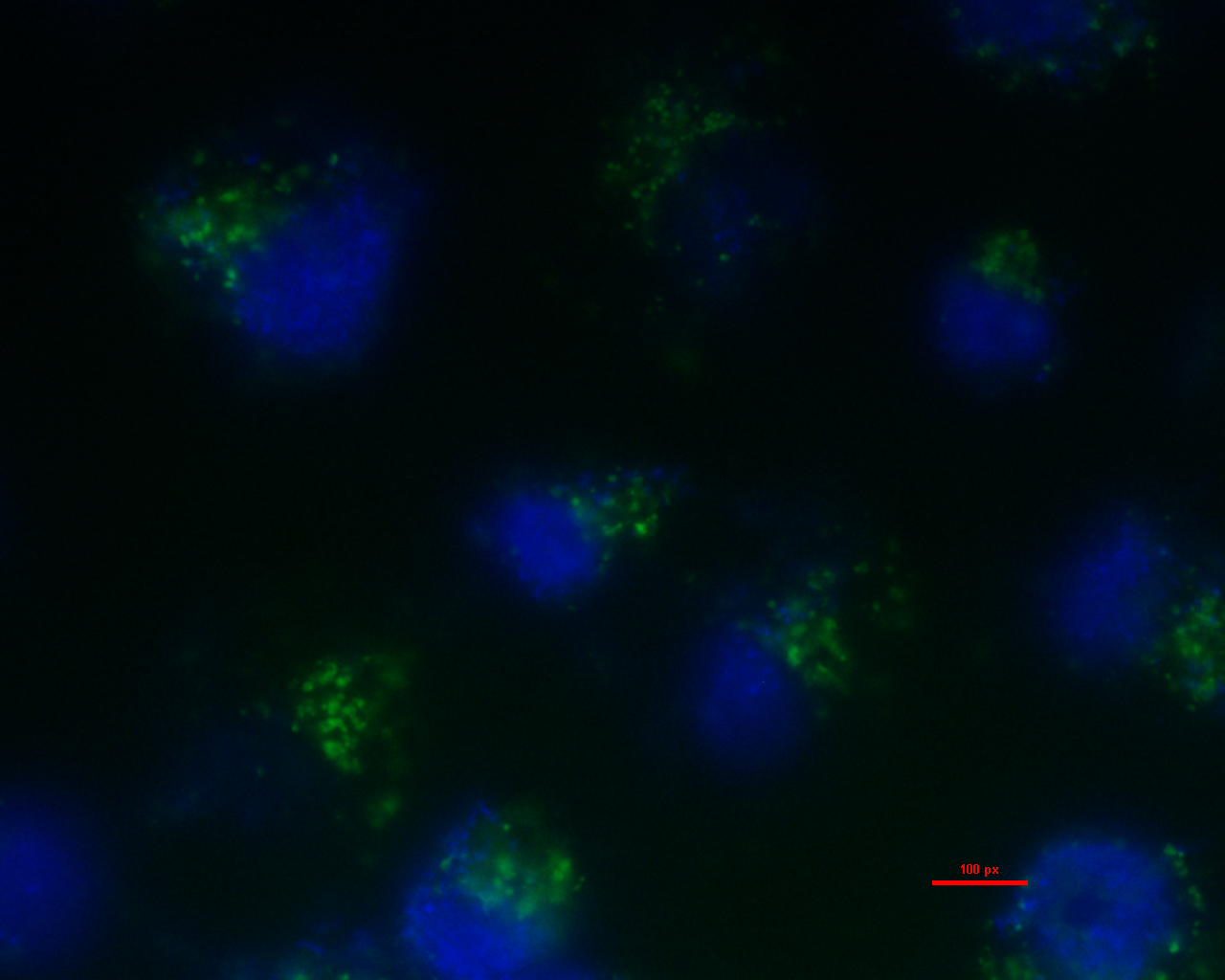

Supplement: Supplemental Information 9 [file peerj-12-16692-s009.zip › original data-figure 4-2/4F/miR-138 inhi+si-NLRP3/1.NLRP3.tif]

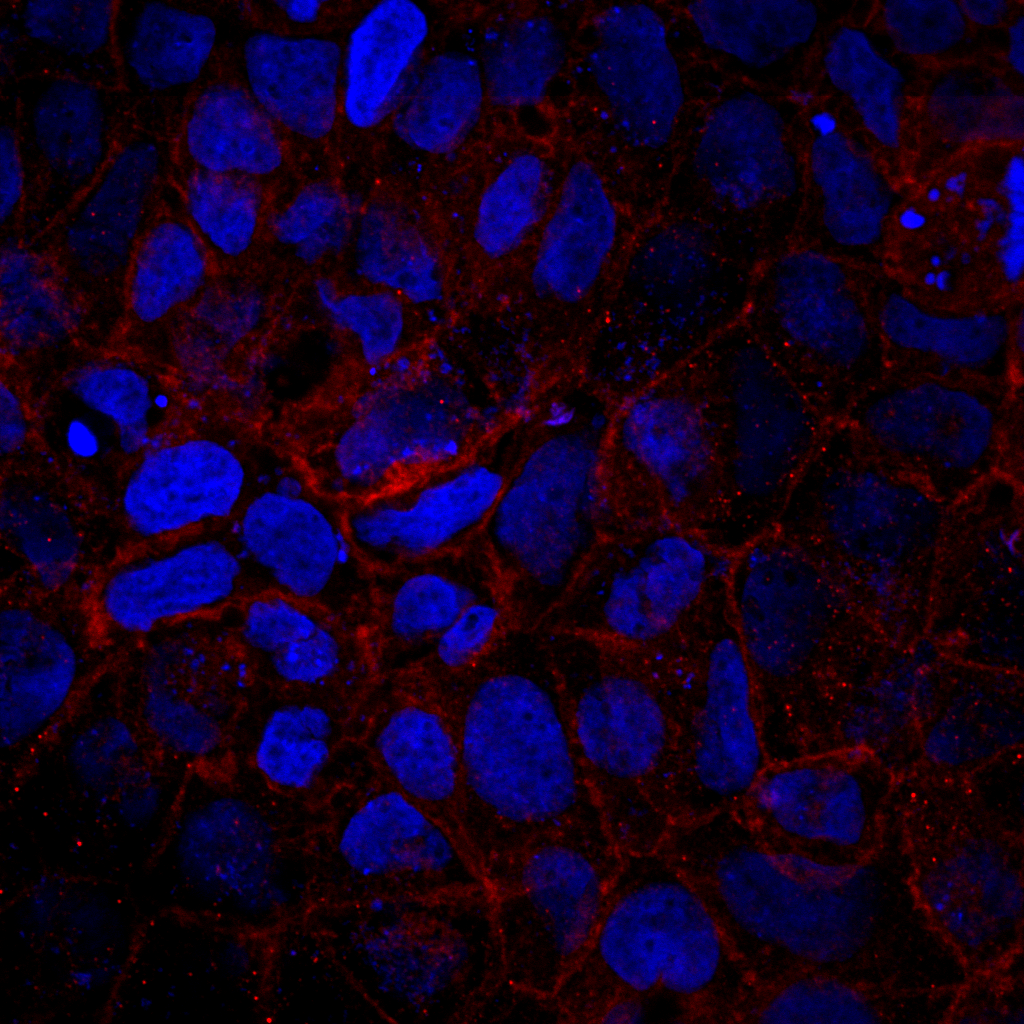

Supplement: Supplemental Information 9 [file peerj-12-16692-s009.zip › original data-figure 4-2/4F/miR-138 inhi+si-NLRP3/2.ZO-1.tif]

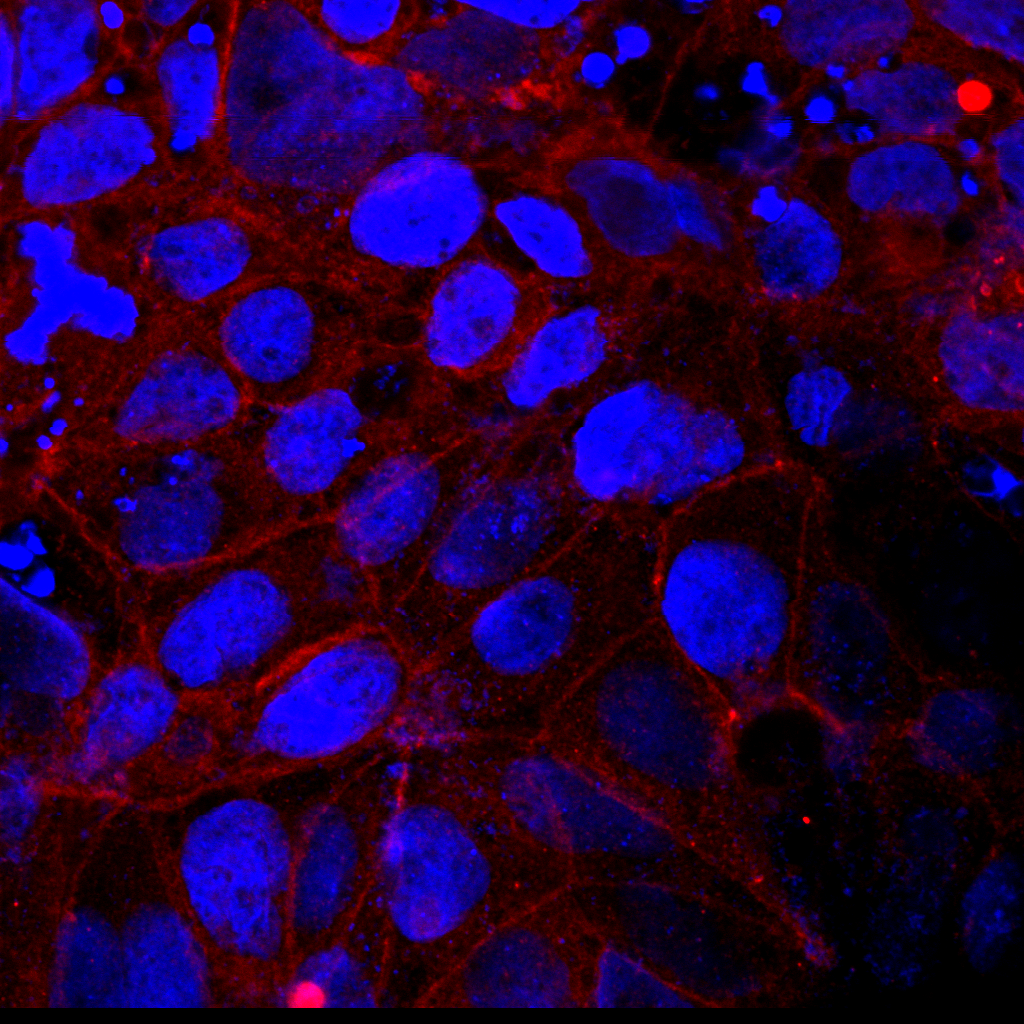

Supplement: Supplemental Information 9 [file peerj-12-16692-s009.zip › original data-figure 4-2/4F/miR-138 inhi+si-NLRP3/3.Occludin.tif]

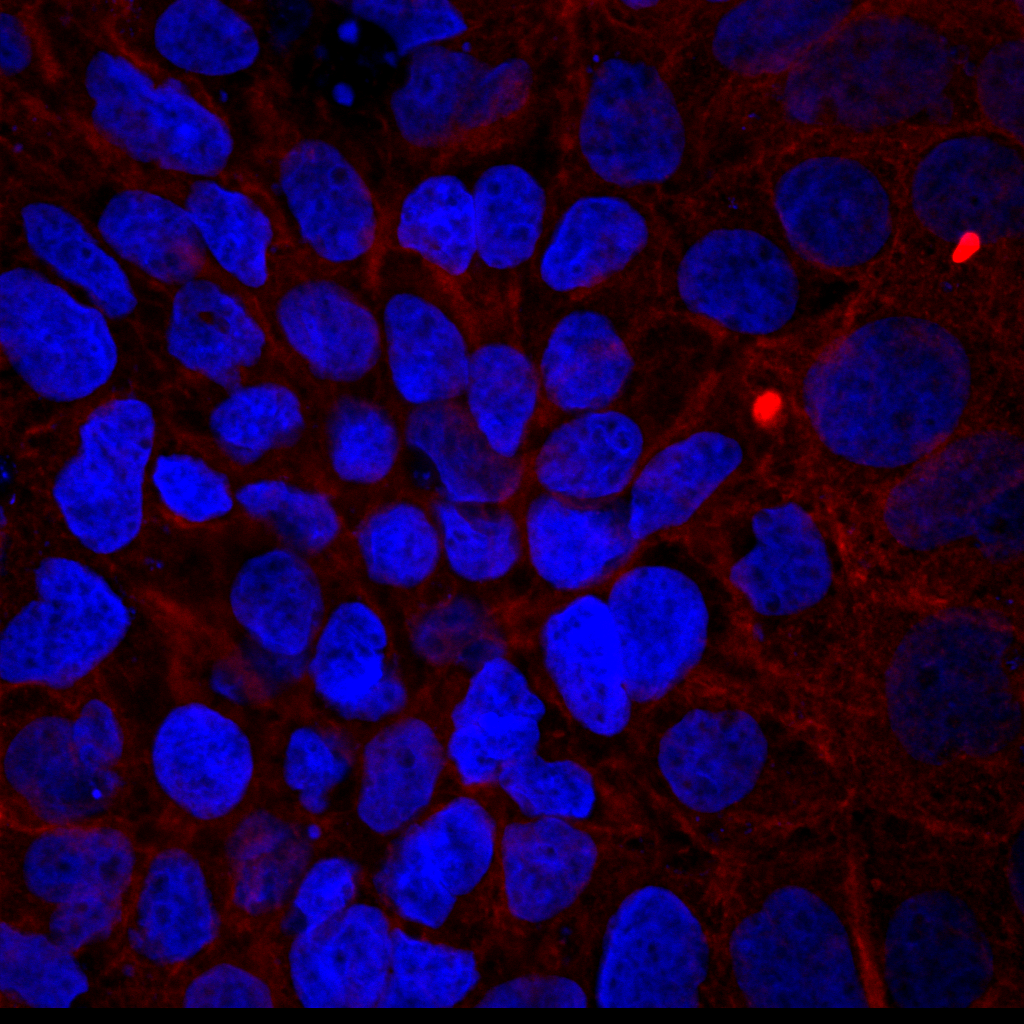

Supplement: Supplemental Information 9 [file peerj-12-16692-s009.zip › original data-figure 4-2/4F/miR-138 inhi+si-NLRP3/4.Claudin-1.tif]

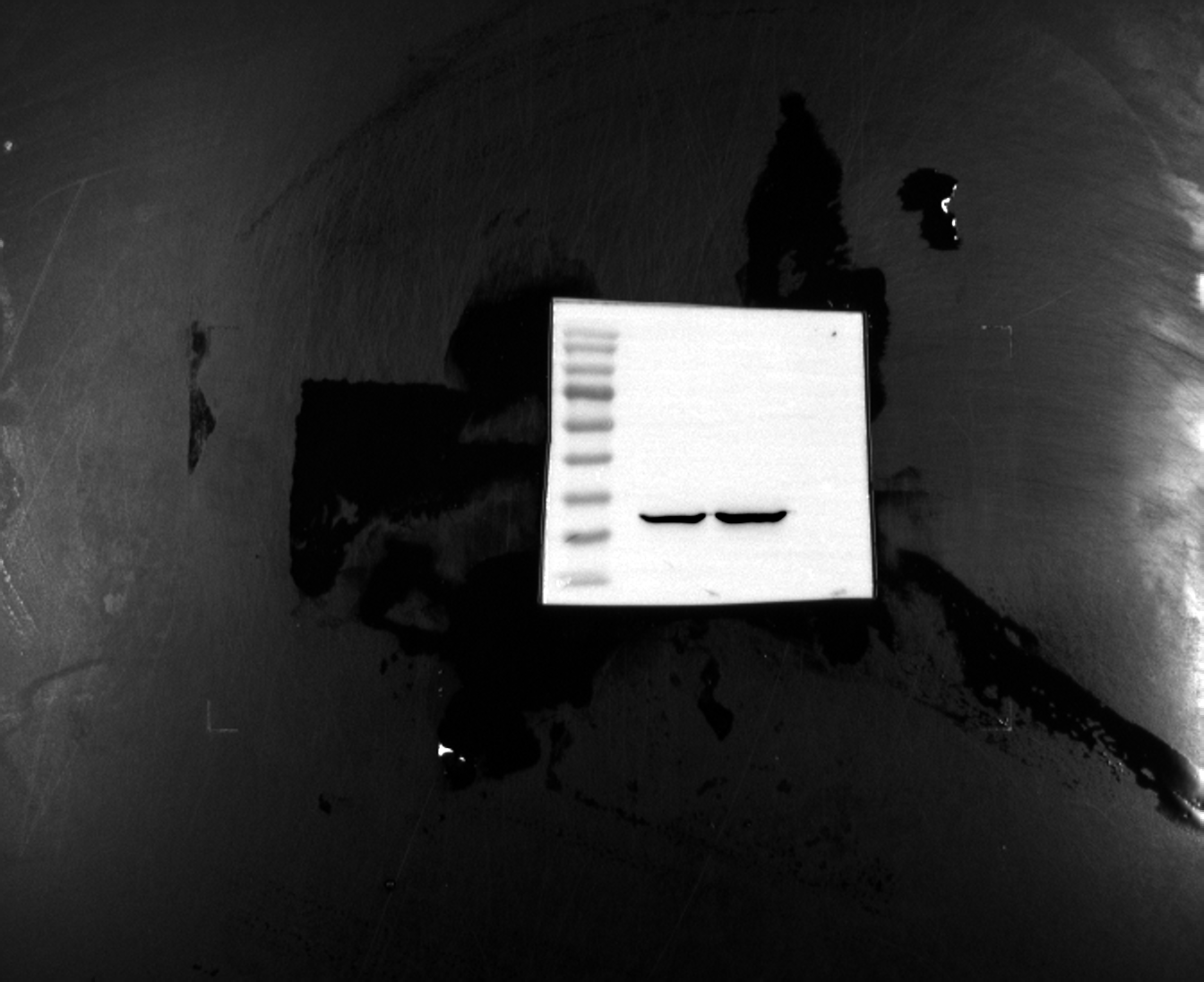

Supplement: Supplemental Information 10 [file peerj-12-16692-s010.zip › original data-figure 5/5A/1.HMGB1.tif]

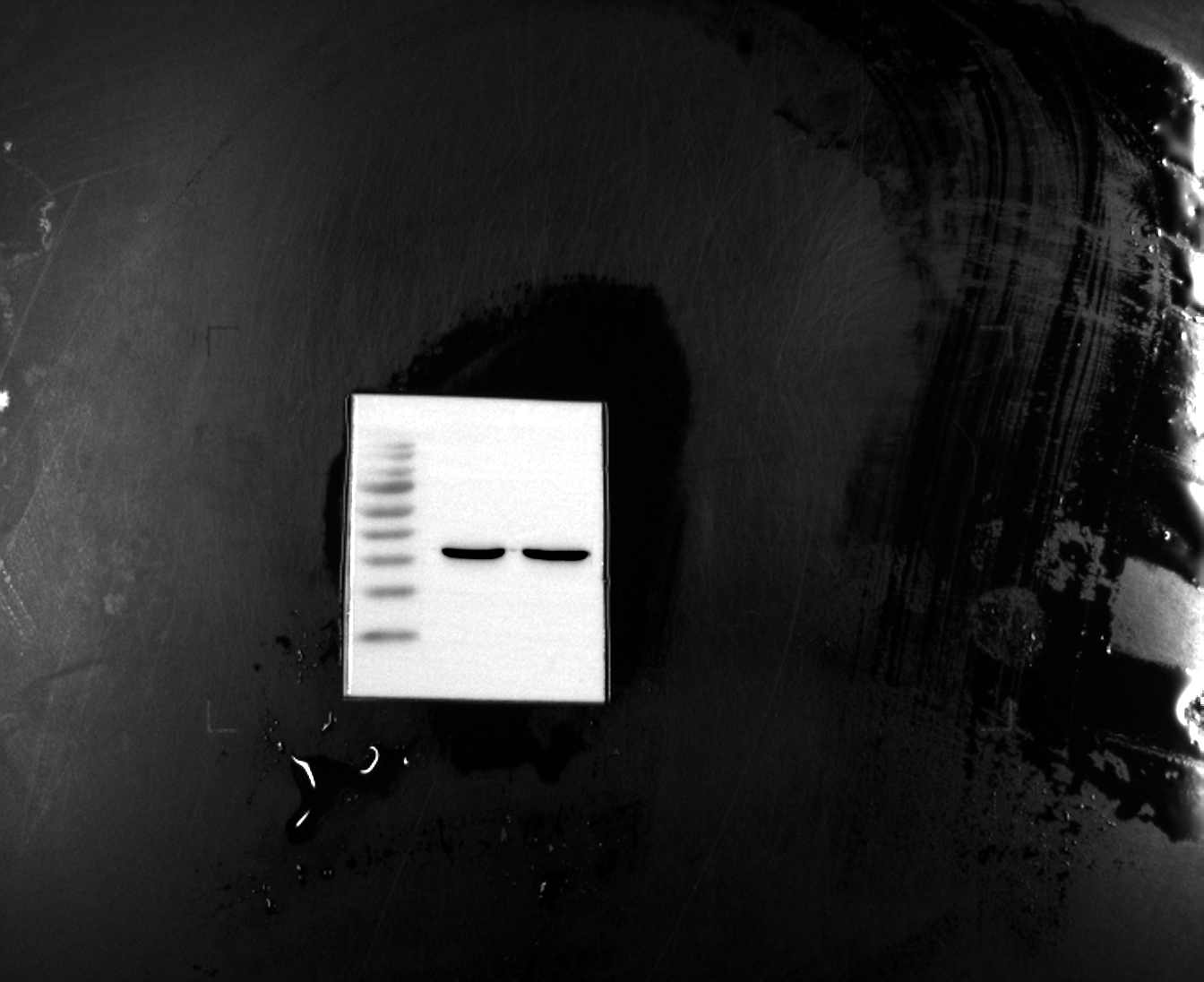

Supplement: Supplemental Information 10 [file peerj-12-16692-s010.zip › original data-figure 5/5A/2.GAPDH.tif]

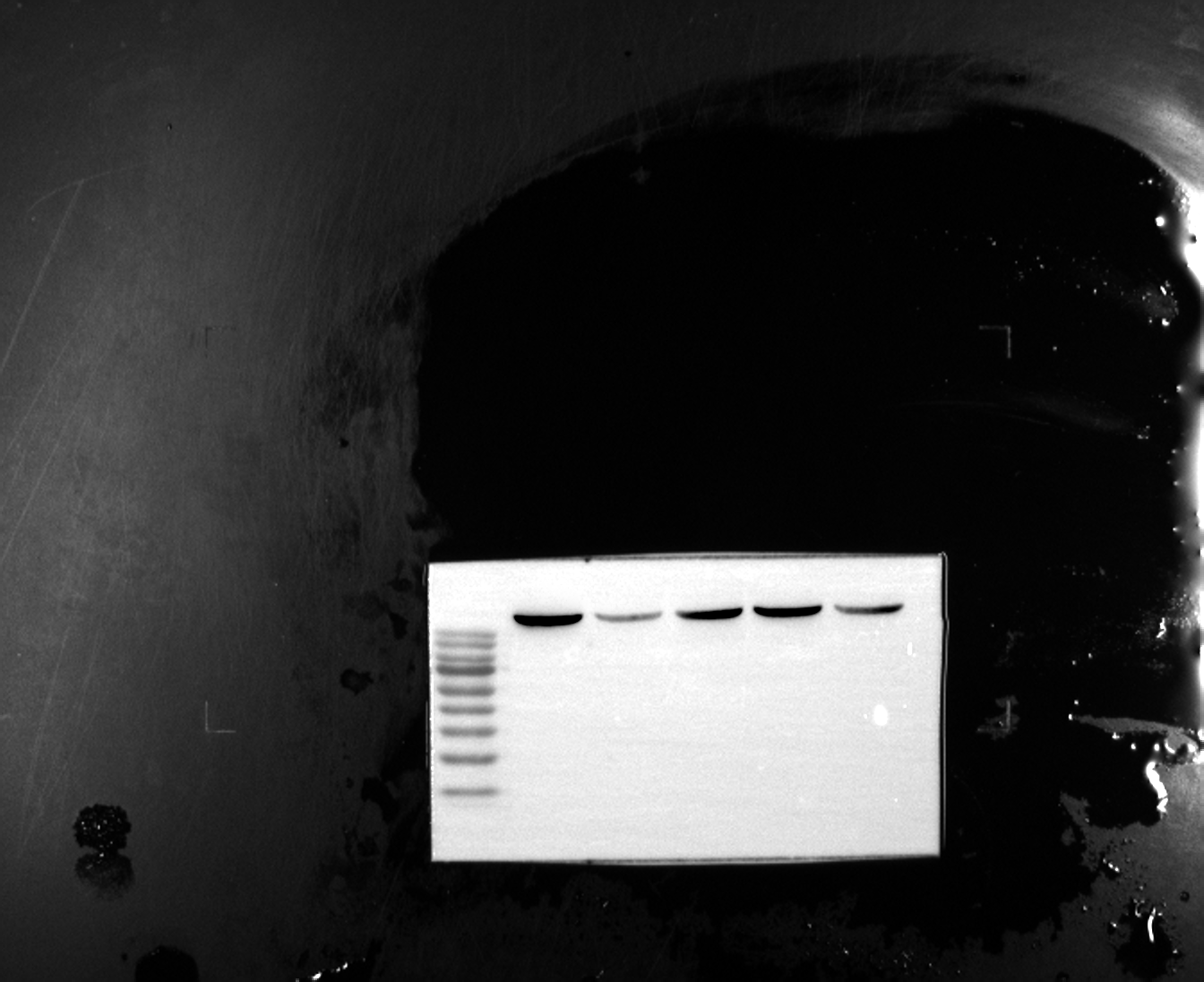

Supplement: Supplemental Information 10 [file peerj-12-16692-s010.zip › original data-figure 5/5B/1.ZO-1.tif]
