# Supplementary material for: miR-138-5p ameliorates intestinal barrier disruption caused by acute superior mesenteric vein thrombosis injury by inhibiting the NLRP3/HMGB1 axis
Source: PeerJ. 2024 Feb 21;12:e16692. doi: 10.7717/peerj.16692 (PMC10893868; doi:10.7717/peerj.16692)
Supplement: Supplemental Information 4 [file peerj-12-16692-s004.docx]

1B


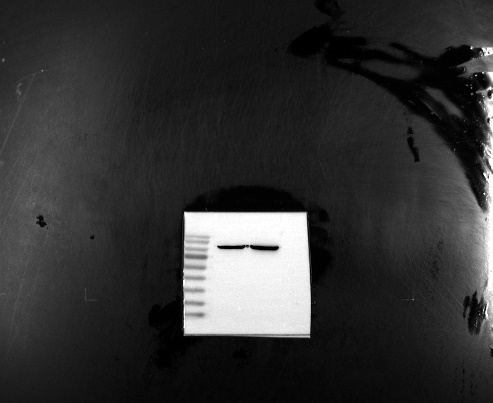

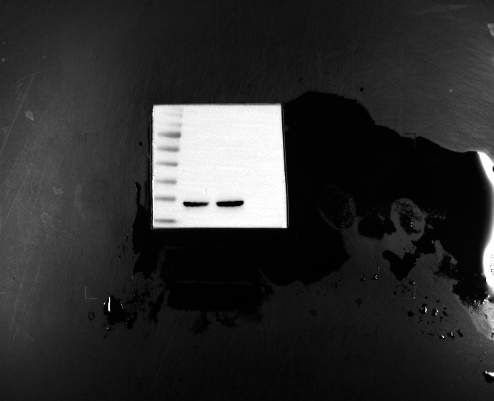

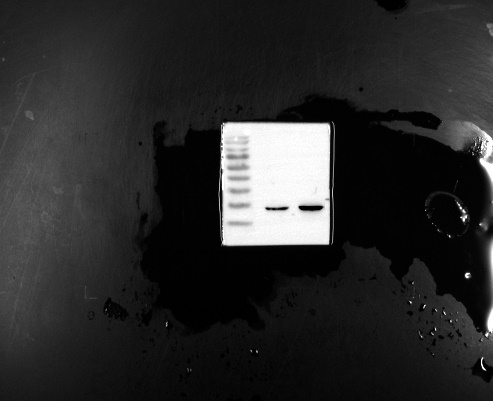

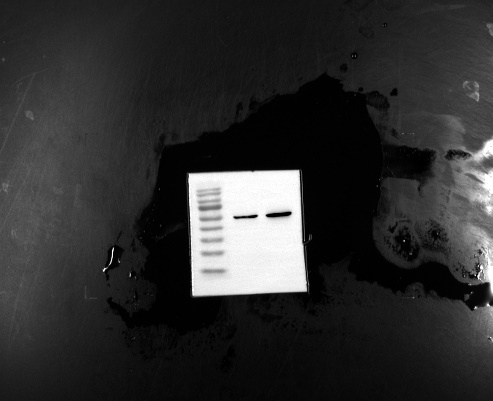

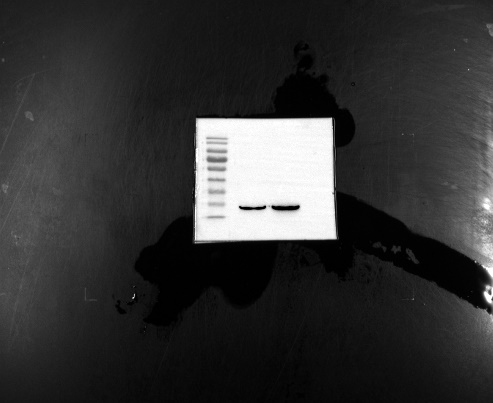

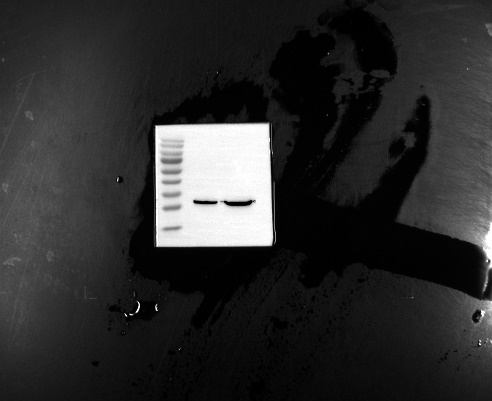

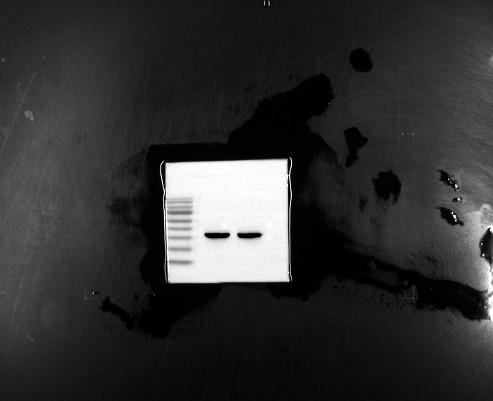


NLRP3 ASC Caspase 1 GSDMD-N IL-18 IL-1β GAPDH

1C


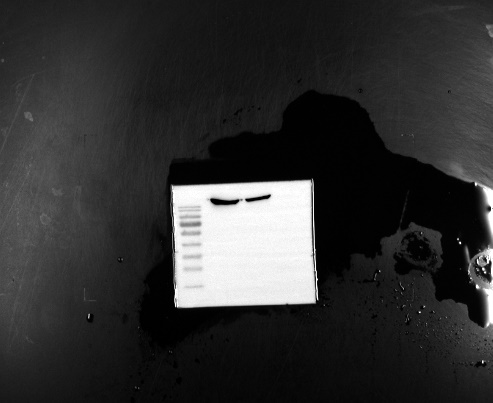

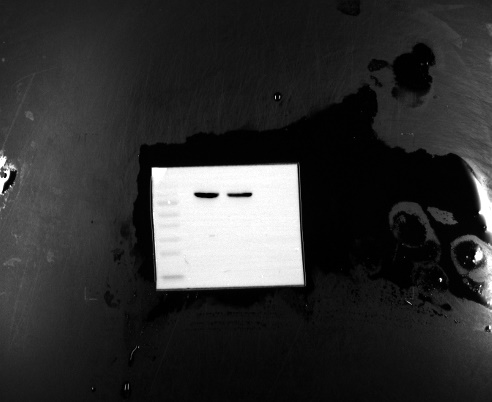

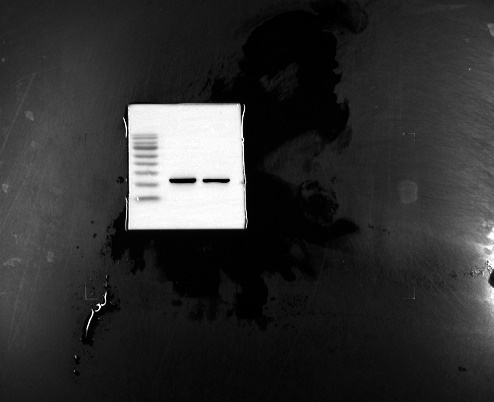

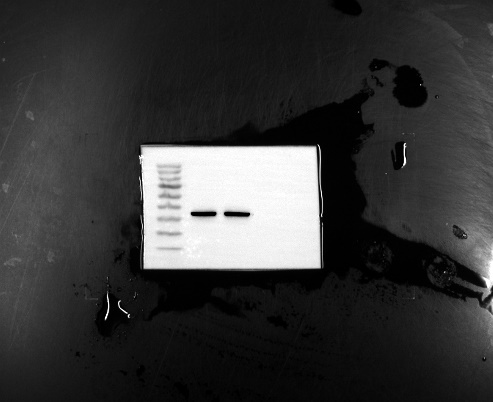


ZO-1 Occludin Claudin-1 GAPDH

1E


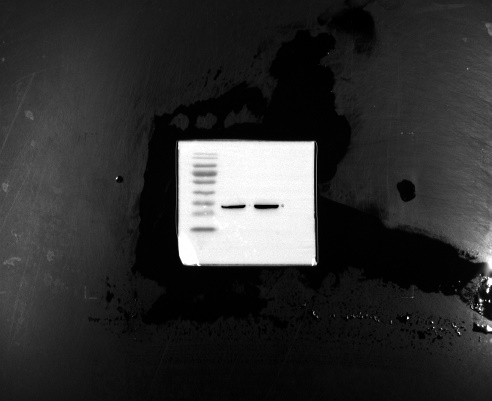

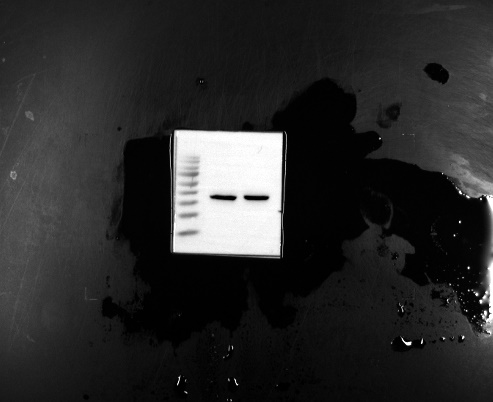


HMGB1 GAPDH

2E


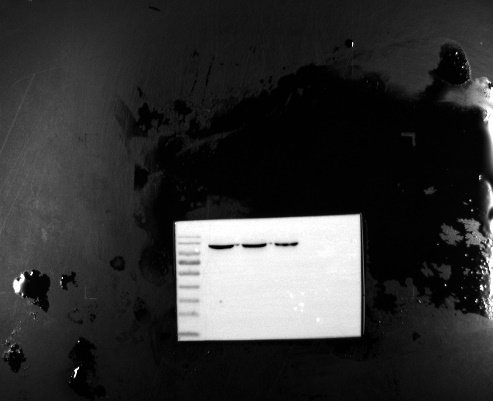

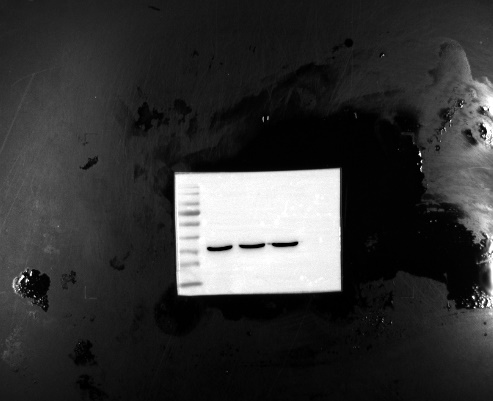


NLRP3 GAPDH

3D


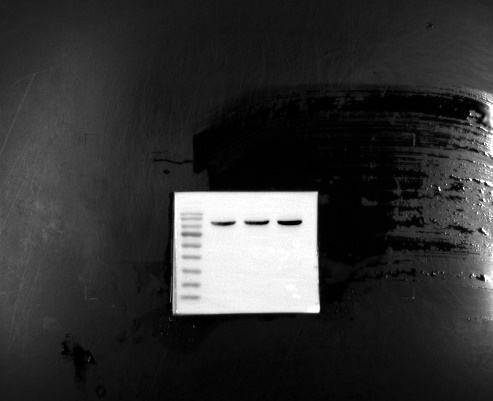

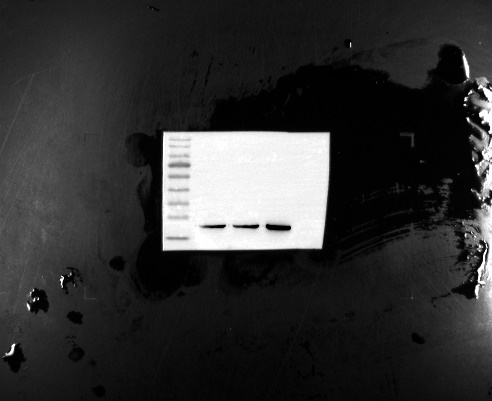

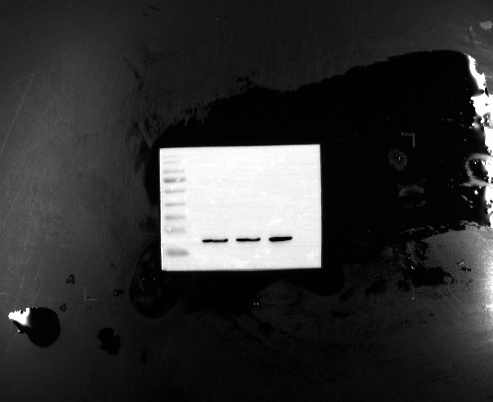

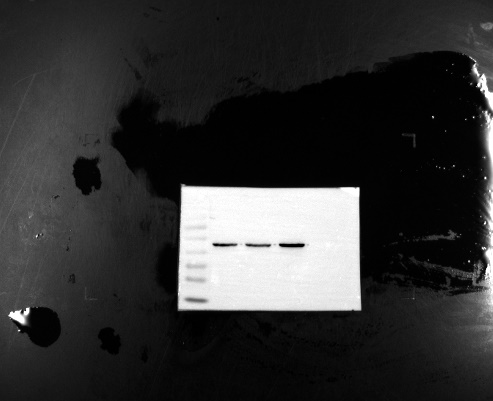

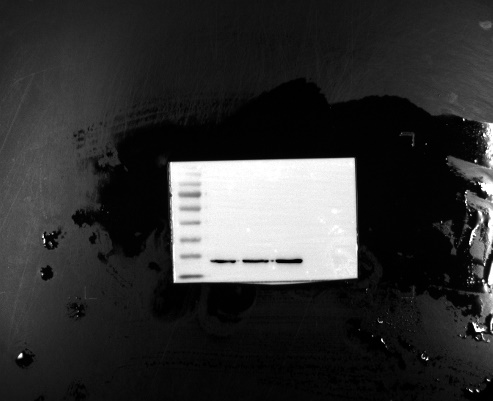

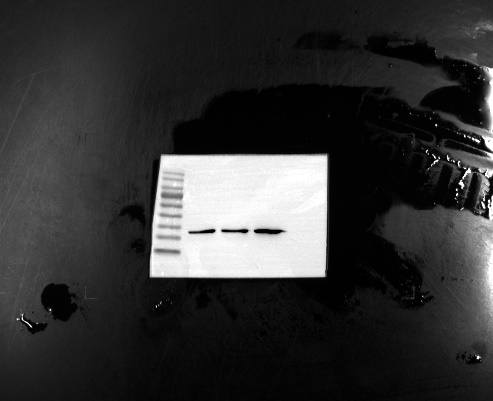

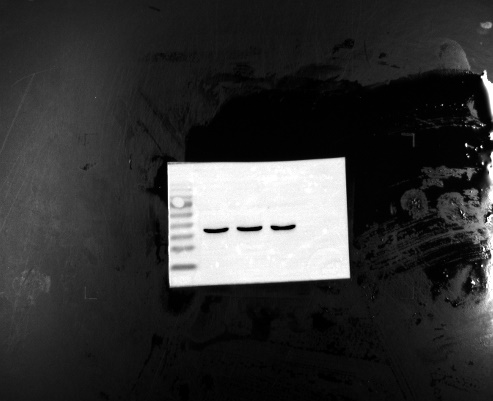


NLRP3 ASC Caspase 1 GSDMD-N IL-18 IL-1β GAPDH

3E


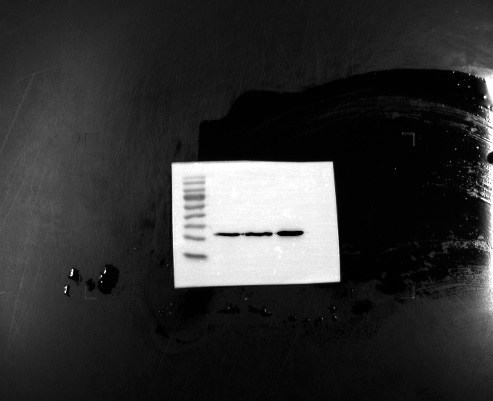

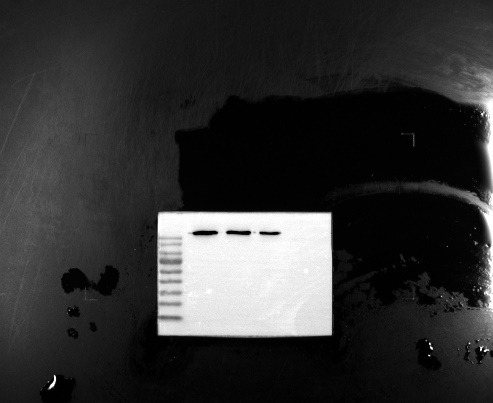

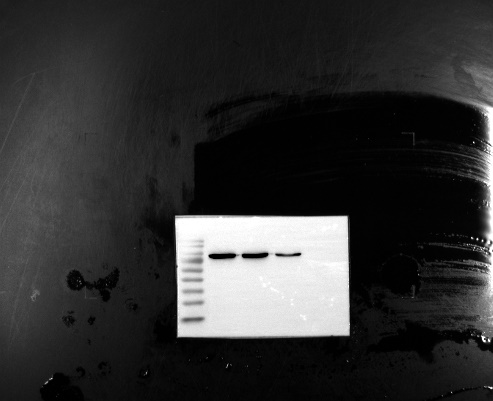

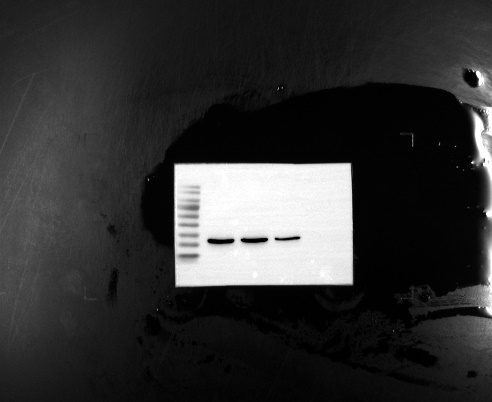

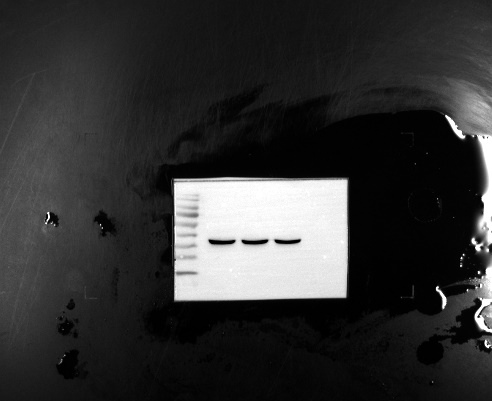


HMGB1 ZO-1 Occludin Claudin-1 GAPDH

4D


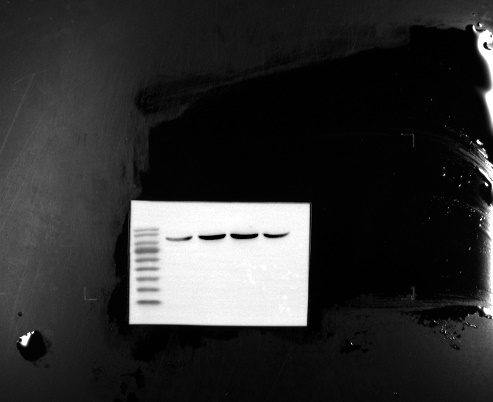

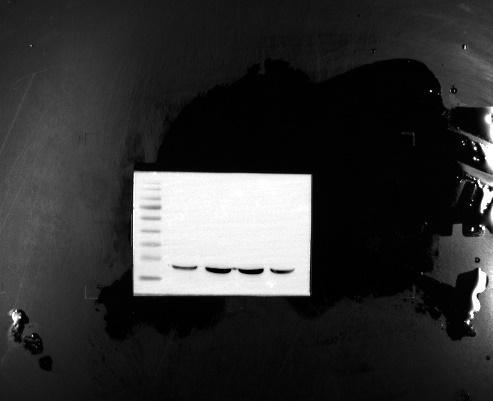

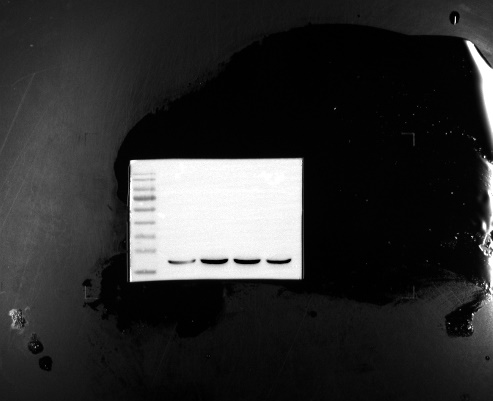

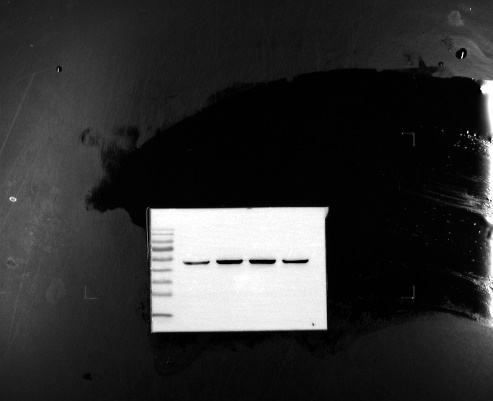

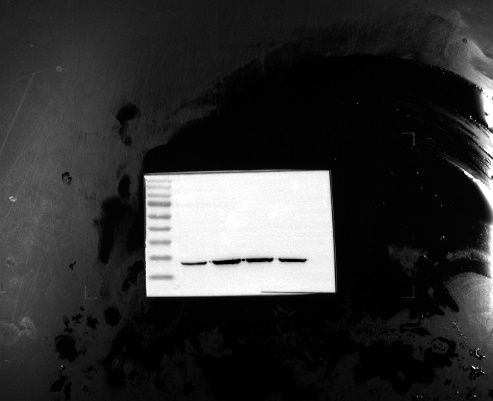

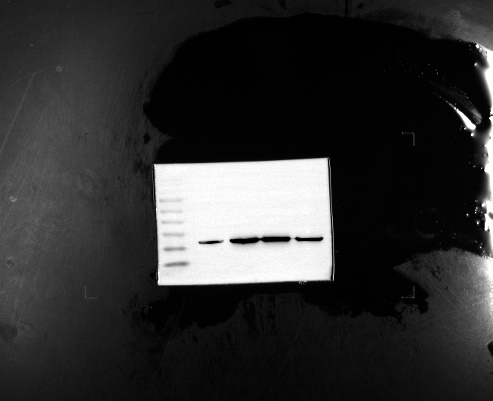


NLRP3 ASC Caspase 1 GSDMD-N IL-18 IL-1β


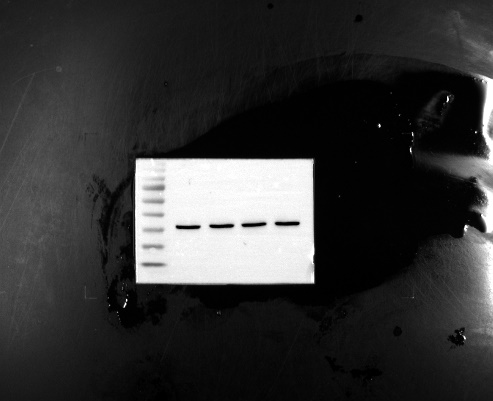


GAPDH

4E


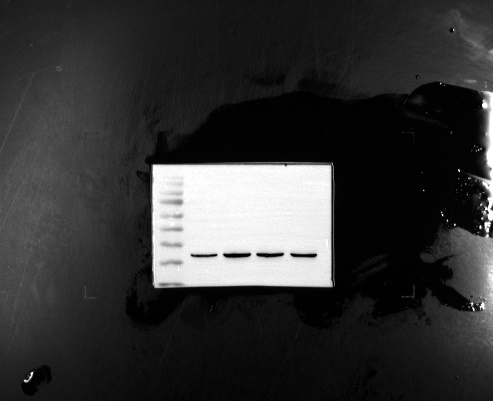

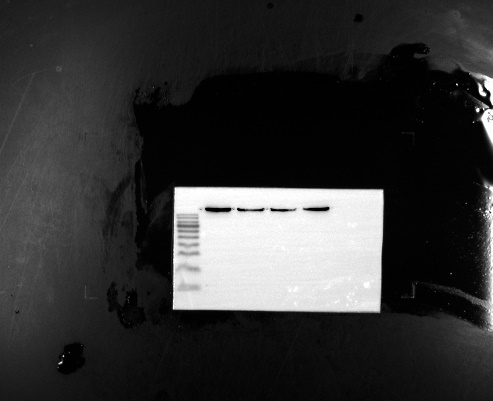

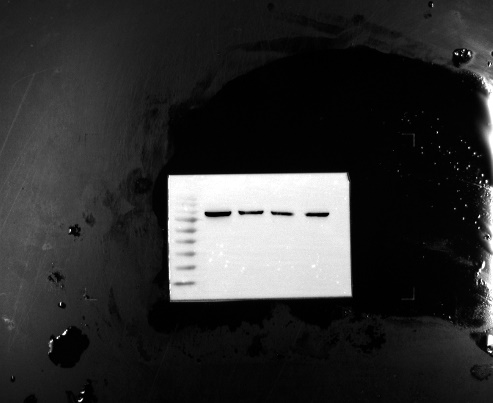

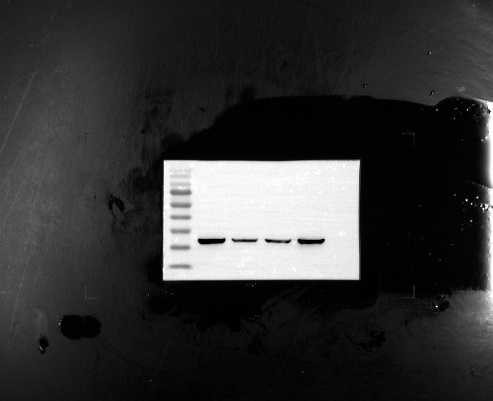

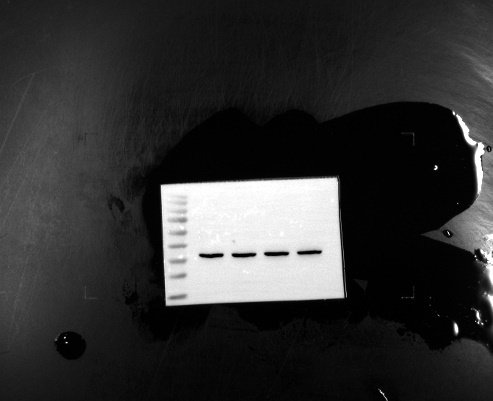


HMGB1 ZO-1 Occludin Claudin-1 GAPDH

5A


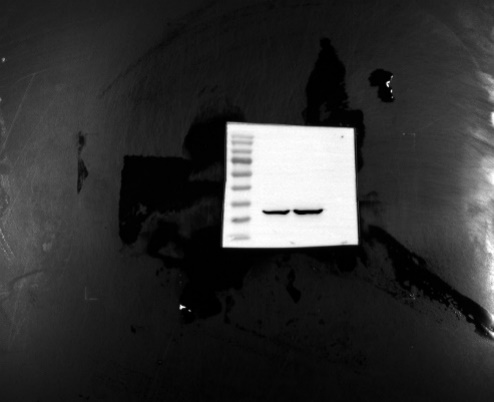

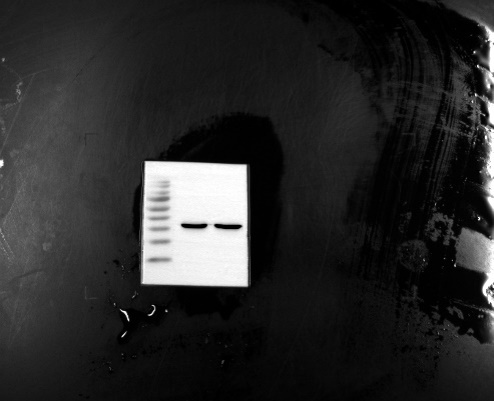


HMGB1 GAPDH

5B


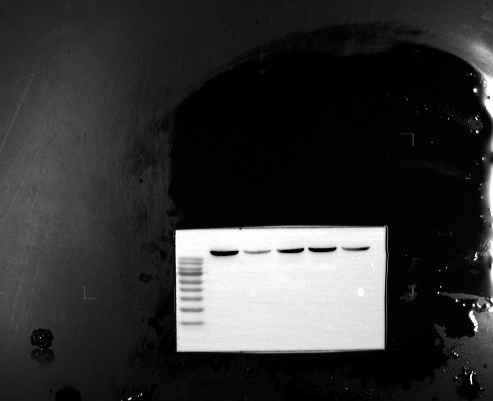

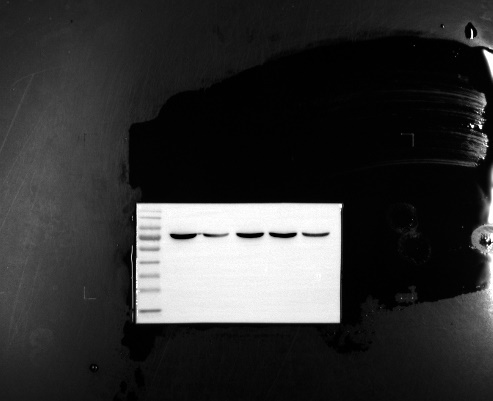

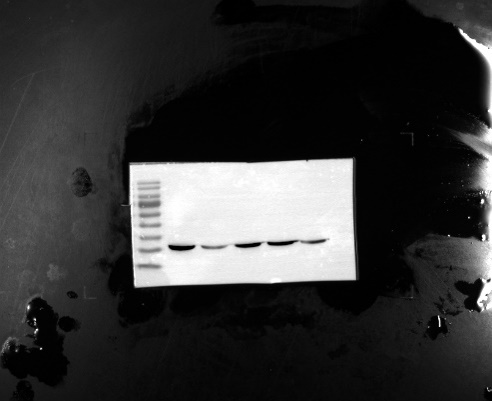

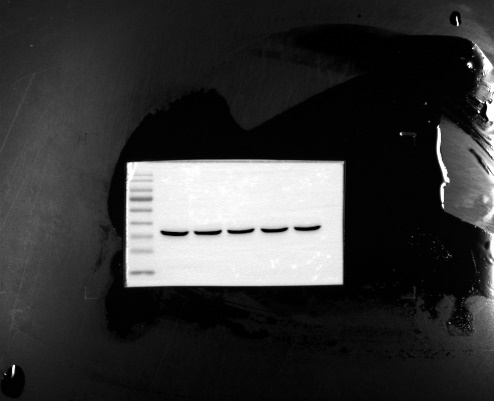


ZO-1 Occludin Claudin-1 GAPDH

6C


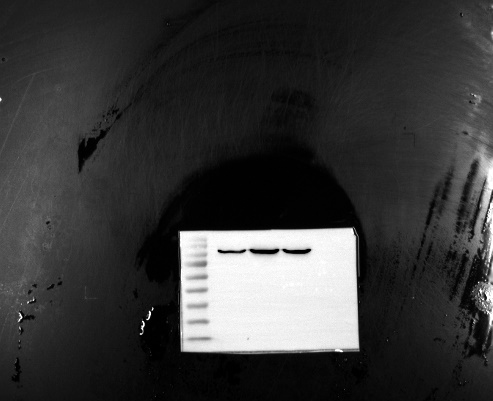

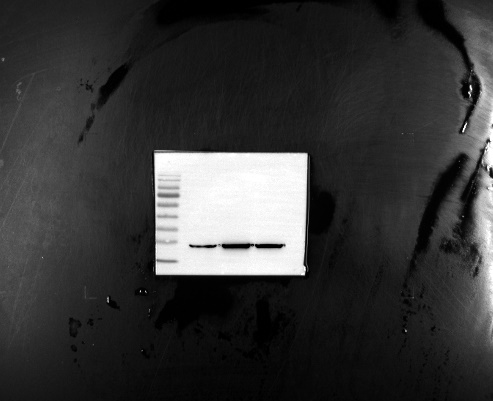

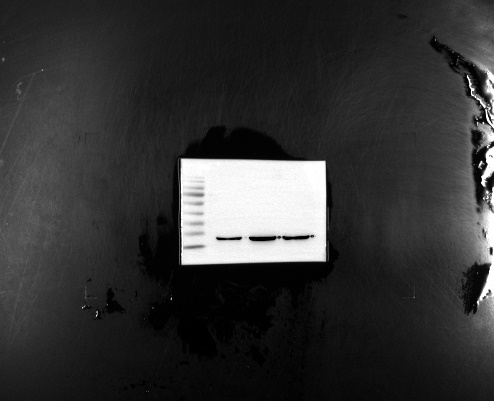

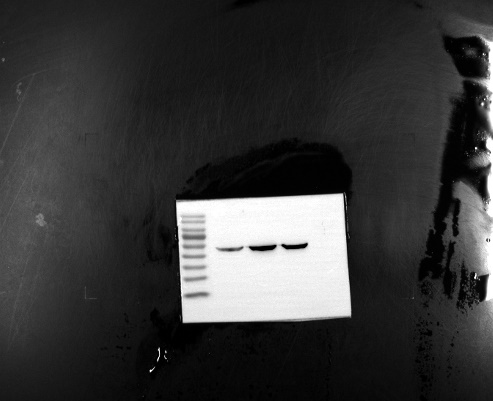

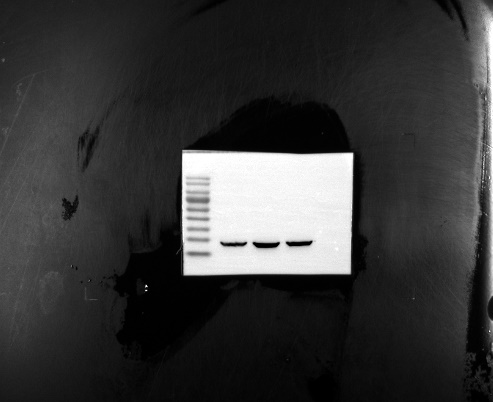

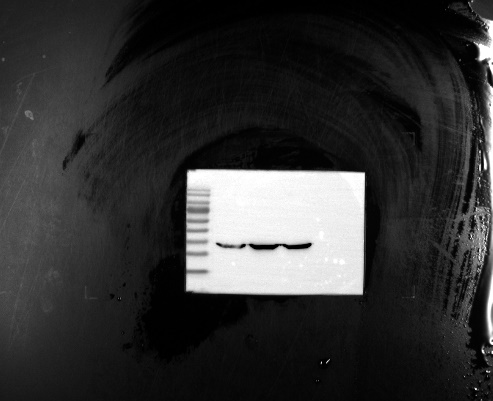


NLRP3 ASC Caspase 1 GSDMD-N IL-18 IL-1β


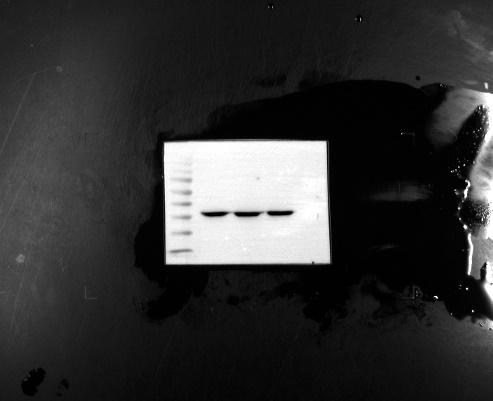


GAPDH

6D


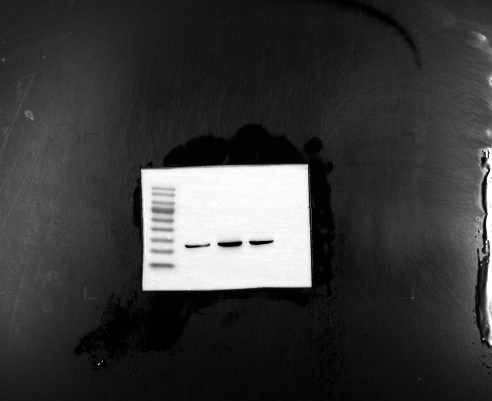

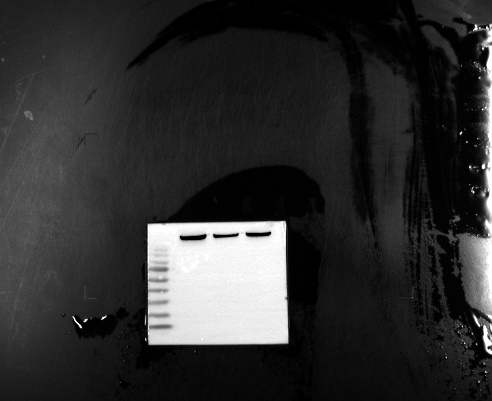

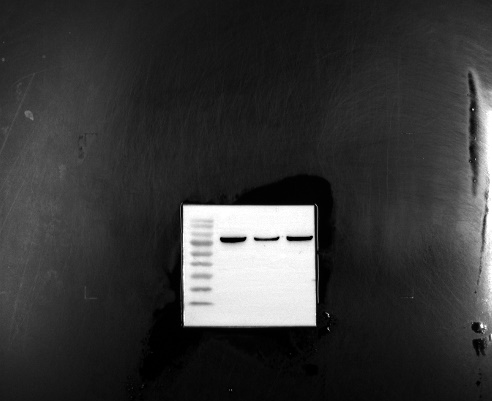

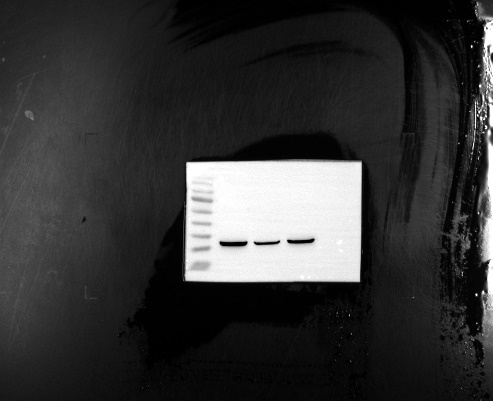

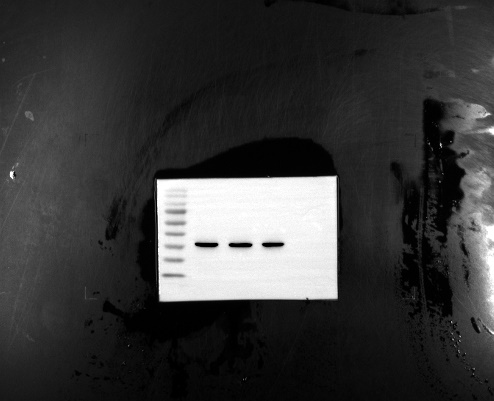


HMGB1 ZO-1 Occludin Claudin-1 GAPDH
