# Supplementary material for: miR-138-5p ameliorates intestinal barrier disruption caused by acute superior mesenteric vein thrombosis injury by inhibiting the NLRP3/HMGB1 axis
Source: PeerJ. 2024 Feb 21;12:e16692. doi: 10.7717/peerj.16692 (PMC10893868; doi:10.7717/peerj.16692)
Supplement: Supplemental Information 6 [file peerj-12-16692-s006.zip › original data-figure 2/image/2B/Results_Report_2023-08-17-141754.pdf]

Plate Results Report

A22-1_Copy.eds

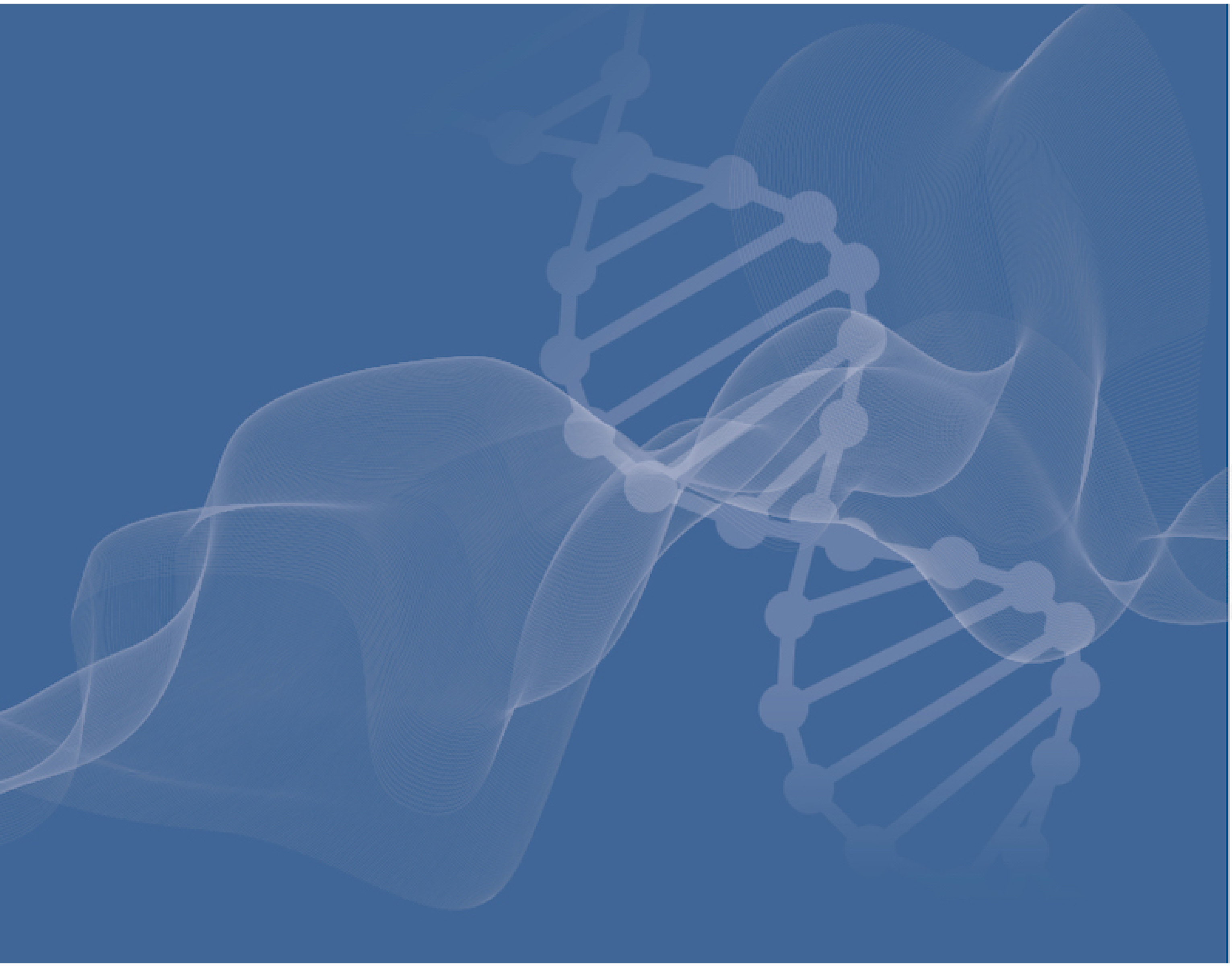

Summary

Property	Details
Bar Code	-
File Name	A22-1_Copy.eds
Run Start Date/Time	Dec 1, 2021 10:16:57 AM
Run End Date/Time	Dec 1, 2021 11:13:01 AM
Run Duration	56 minutes, and 4 seconds
Operator	DEFAULT
Instrument Name	SVT004
Instrument Type	QuantStudio™ 3 System
Instrument Serial Number	SVT004
Block Type	96-Well 0.2-mL
Block Serial Number	41145627
Heated Cover Serial Number	N/A
PCR Stage/Step Number	Stage 2, Step 2
Melt Stage Number	Stage 3
Quantification Cycle Method	Baseline Threshold
Comment	-
Software Name and Version	Design & Analysis Software v2.6.0
Plugin Name and Version	Primary Analysis v1.7.0, Relative Quantification v1.5.0
Analysis Date/Time	Jul 17, 2023 2:44:28 PM

Well Table

Well	Sample	Target	Task	Cq	Cq Confidence	Amp Score	Amp Status	Cq Threshold	Baseline Start/End	Melt Temp
C7	Sham	GAPDH	Unknown	17.76	0.989	1.834	AMP	1.865	3-11	82.653
C8	Sham	GAPDH	Unknown	17.28	0.96	1.827	AMP	1.865	3-11	82.653
C9	Sham	GAPDH	Unknown	17.347	0.985	1.838	AMP	1.865	3-10	82.655
C10	Sham	miR-138-5p	Unknown	24.989	0.991	1.861	AMP	2.05	3-16	82.356
C11	Sham	miR-138-5p	Unknown	24.956	0.99	1.85	AMP	2.05	3-16	82.208
C12	Sham	miR-138-5p	Unknown	24.981	0.99	1.85	AMP	2.05	3-16	82.208
D7	Sham	GAPDH	Unknown	17.651	0.985	1.844	AMP	1.865	3-10	82.653
D8	Sham	GAPDH	Unknown	17.254	0.986	1.844	AMP	1.865	3-10	82.653
D9	Sham	GAPDH	Unknown	17.372	0.99	1.845	AMP	1.865	3-10	82.655
D10	Sham	miR-138-5p	Unknown	24.937	0.987	1.862	AMP	2.05	3-16	82.356
D11	Sham	miR-138-5p	Unknown	24.938	0.99	1.855	AMP	2.05	3-16	82.059
D12	Sham	miR-138-5p	Unknown	24.961	0.987	1.861	AMP	2.05	3-16	82.059
E7	Sham	GAPDH	Unknown	18.008	0.945	1.838	AMP	1.865	3-12	82.504
E8	Sham	GAPDH	Unknown	18.045	0.987	1.851	AMP	1.865	3-11	82.504
E9	Sham	GAPDH	Unknown	17.389	0.988	1.837	AMP	1.865	3-10	82.655
E10	Sham	miR-138-5p	Unknown	24.989	0.991	1.853	AMP	2.05	3-16	82.356
E11	Sham	miR-138-5p	Unknown	24.984	0.992	1.841	AMP	2.05	3-16	81.91
E12	Sham	miR-138-5p	Unknown	24.885	0.985	1.861	AMP	2.05	3-16	82.059
F7	ASMVT	GAPDH	Unknown	17.922	0.993	1.834	AMP	1.865	3-10	82.504
F8	ASMVT	GAPDH	Unknown	17.506	0.989	1.841	AMP	1.865	3-10	82.653
F9	ASMVT	GAPDH	Unknown	17.53	0.989	1.839	AMP	1.865	3-10	82.655
F10	ASMVT	miR-138-5p	Unknown	28.066	0.984	1.868	AMP	2.05	3-19	82.058
F11	ASMVT	miR-138-5p	Unknown	28.09	0.983	1.85	AMP	2.05	3-20	81.612
F12	ASMVT	miR-138-5p	Unknown	28.029	0.983	1.852	AMP	2.05	3-20	81.761
G7	ASMVT	GAPDH	Unknown	17.363	0.99	1.847	AMP	1.865	3-10	82.653
G8	ASMVT	GAPDH	Unknown	17.918	0.988	1.847	AMP	1.865	3-11	82.504
G9	ASMVT	GAPDH	Unknown	17.876	0.99	1.845	AMP	1.865	3-10	82.505
G10	ASMVT	miR-138-5p	Unknown	28.076	0.985	1.86	AMP	2.05	3-18	82.207
G11	ASMVT	miR-138-5p	Unknown	27.984	0.989	1.839	AMP	2.05	3-19	81.761
G12	ASMVT	miR-138-5p	Unknown	28.085	0.969	1.857	AMP	2.05	3-20	81.761

Well	Sample	Target	Task	Cq	Cq Confi dence	Amp Score	Amp Status	Cq Thres hold	Baseline Start/End	Melt Temp
H7	ASMVT	GAPDH	Unknown	17.725	0.986	1.849	AMP	1.865	3-10	82.653
H8	ASMVT	GAPDH	Unknown	17.528	0.992	1.842	AMP	1.865	3-10	82.504
H9	ASMVT	GAPDH	Unknown	17.43	0.989	1.836	AMP	1.865	3-9	82.655
H10	ASMVT	miR-138-5p	Unknown	27.978	0.992	1.859	AMP	2.05	3-20	82.356
H11	ASMVT	miR-138-5p	Unknown	27.979	0.991	1.858	AMP	2.05	3-19	81.761
H12	ASMVT	miR-138-5p	Unknown	28.139	0.984	1.836	AMP	2.05	3-20	81.761

Replicate Group Table

Sample	Target	No. of Replicates	Cq Mean	Cq SD
ASMVT	GAPDH	9	17.644	0.219
ASMVT	miR-138-5p	9	28.047	0.058
Sham	GAPDH	9	17.567	0.309
Sham	miR-138-5p	9	24.958	0.034

Plate Layout

	1	2	3	4	5	6	7	8	9	10	11	12
A												
B												
C							Sham<br>GAPDH (17.76)	Sham<br>GAPDH (17.28)	Sham<br>GAPDH (17.347)	Sham<br>miR-138-5p (24.989)	Sham<br>miR-138-5p (24.956)	Sham<br>miR-138-5p (24.981)
D							Sham<br>GAPDH (17.651)	Sham<br>GAPDH (17.254)	Sham<br>GAPDH (17.372)	Sham<br>miR-138-5p (24.937)	Sham<br>miR-138-5p (24.938)	Sham<br>miR-138-5p (24.961)
E							Sham<br>GAPDH (18.008)	Sham<br>GAPDH (18.045)	Sham<br>GAPDH (17.389)	Sham<br>miR-138-5p (24.989)	Sham<br>miR-138-5p (24.984)	Sham<br>miR-138-5p (24.885)
F							ASMVT<br>GAPDH (17.922)	ASMVT<br>GAPDH (17.506)	ASMVT<br>GAPDH (17.53)	ASMVT<br>miR-138-5p (28.066)	ASMVT<br>miR-138-5p (28.09)	ASMVT<br>miR-138-5p (28.029)
G							ASMVT<br>GAPDH (17.363)	ASMVT<br>GAPDH (17.918)	ASMVT<br>GAPDH (17.876)	ASMVT<br>miR-138-5p (28.076)	ASMVT<br>miR-138-5p (27.984)	ASMVT<br>miR-138-5p (28.085)
H							ASMVT<br>GAPDH (17.725)	ASMVT<br>GAPDH (17.528)	ASMVT<br>GAPDH (17.43)	ASMVT<br>miR-138-5p (27.978)	ASMVT<br>miR-138-5p (27.979)	ASMVT<br>miR-138-5p (28.139)

Amplification Plot (dRn)

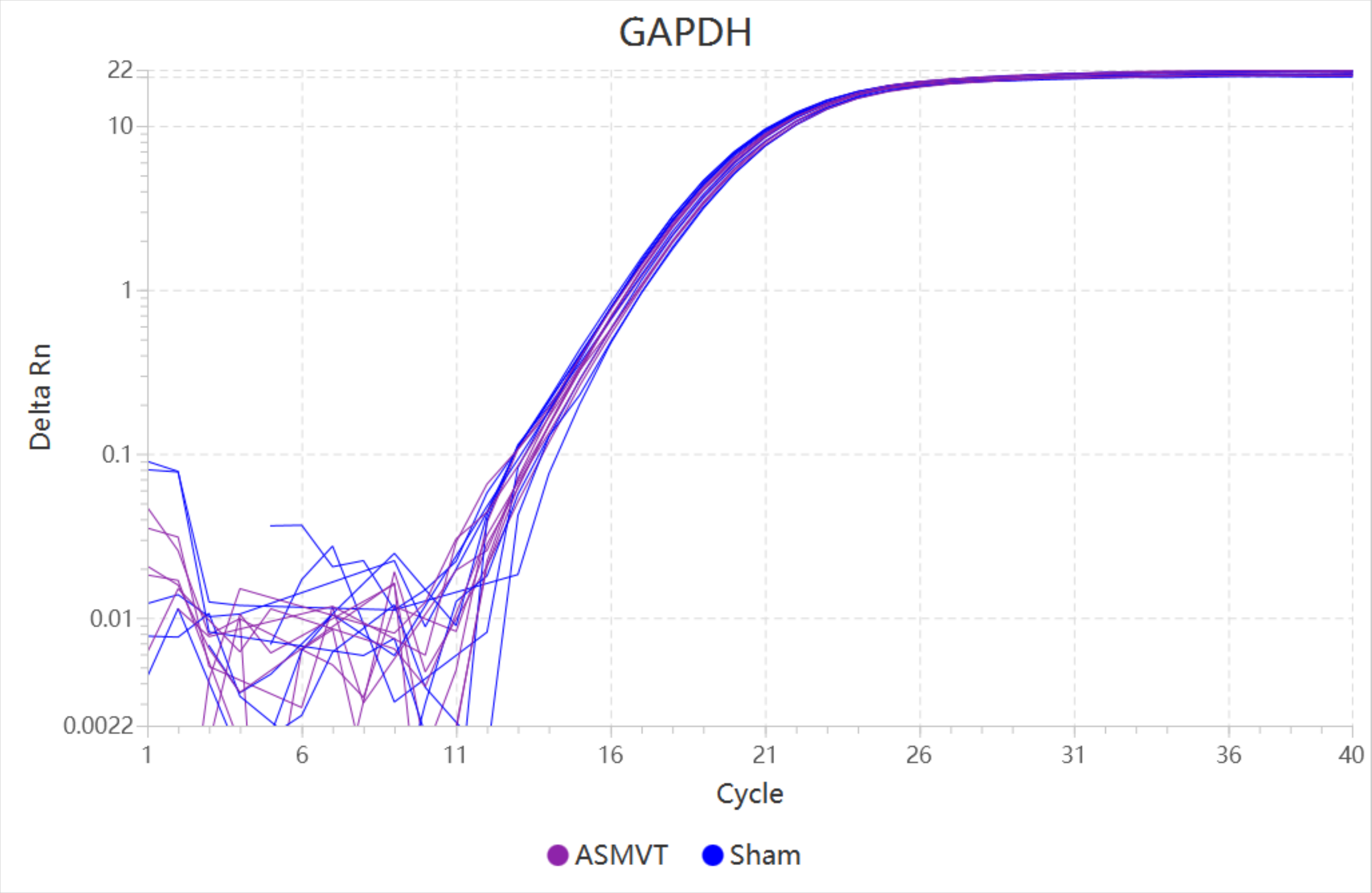

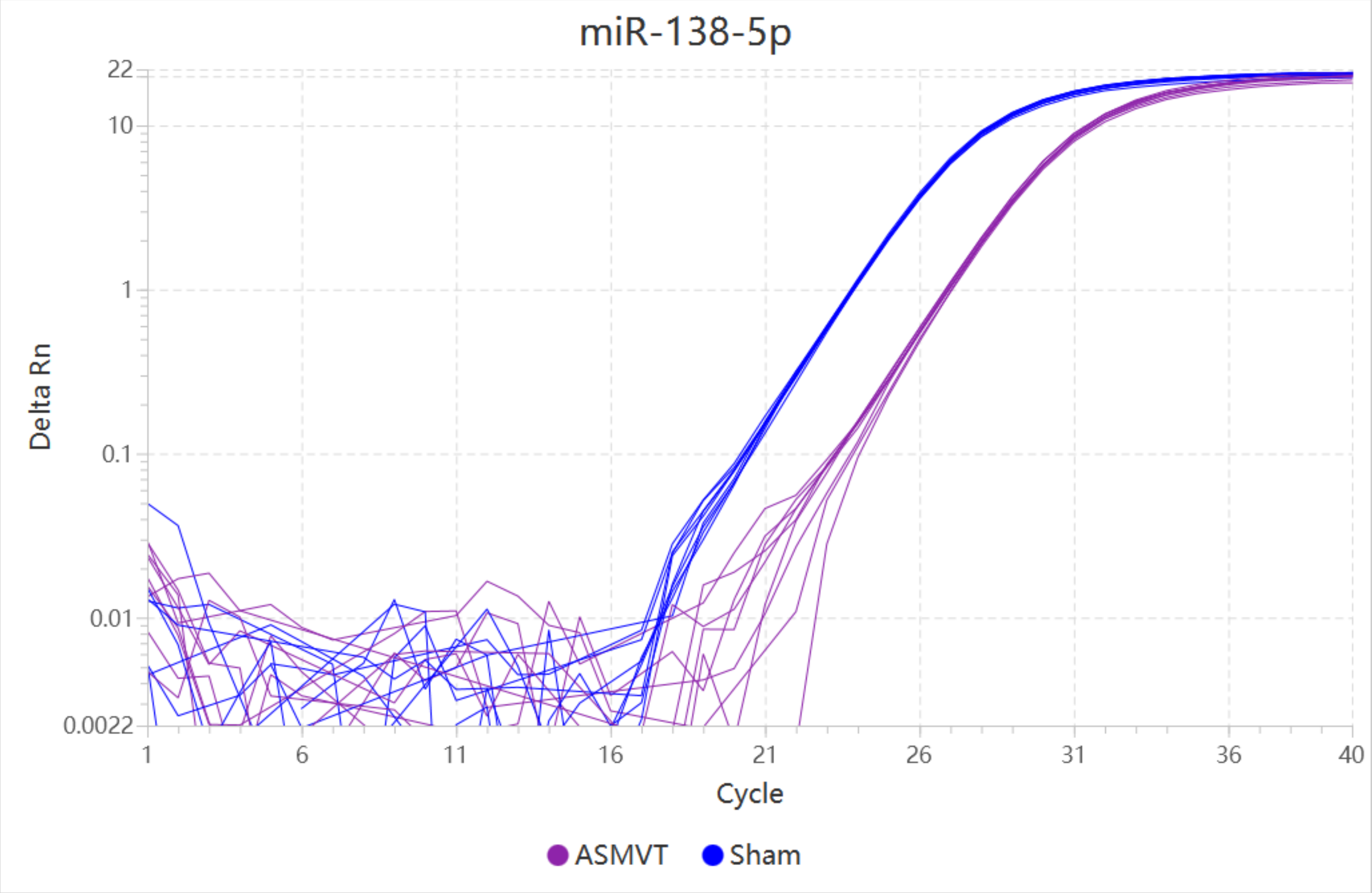

Amplification Plot (Rn)

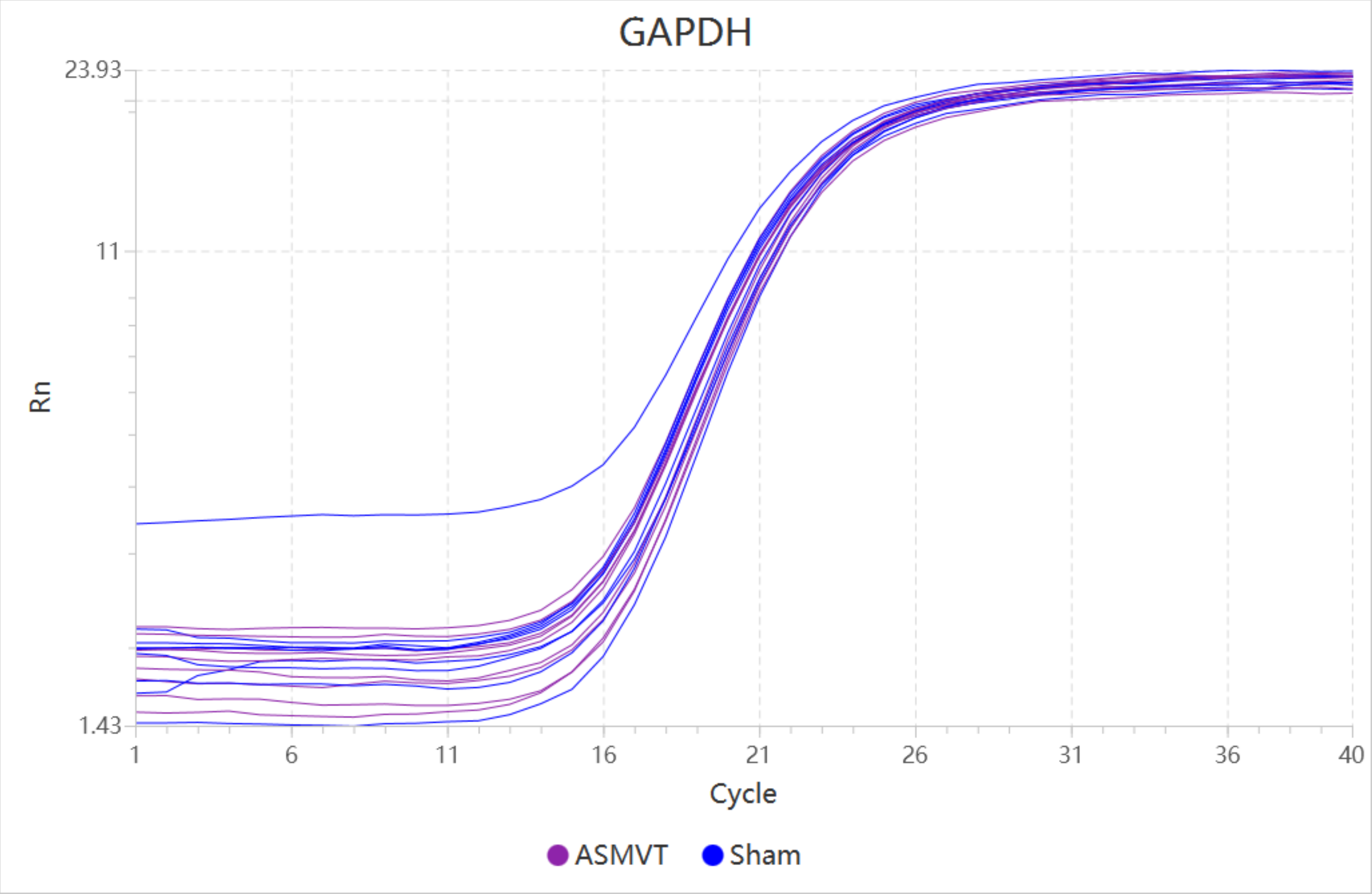

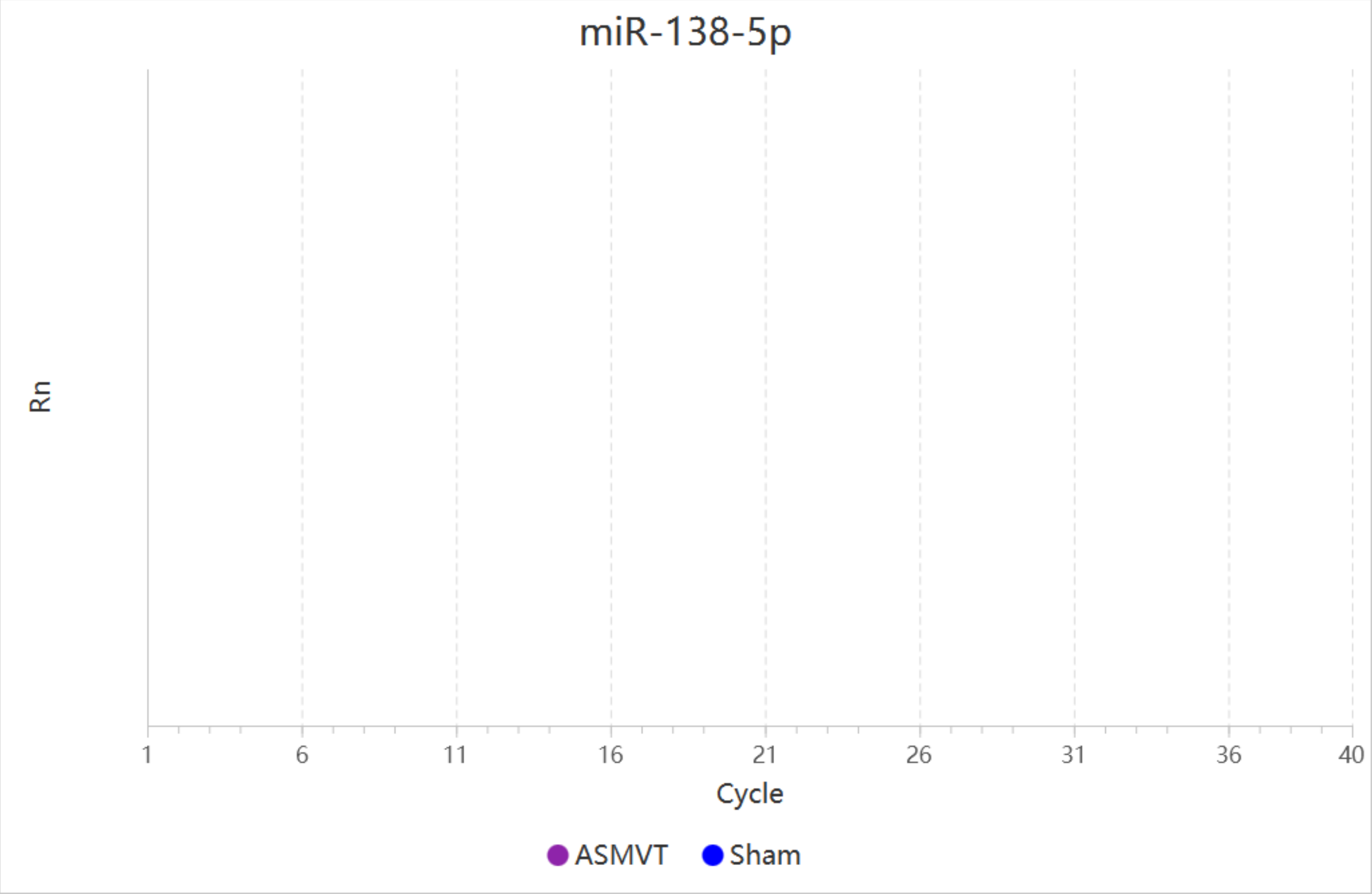

Melt Curve Plot

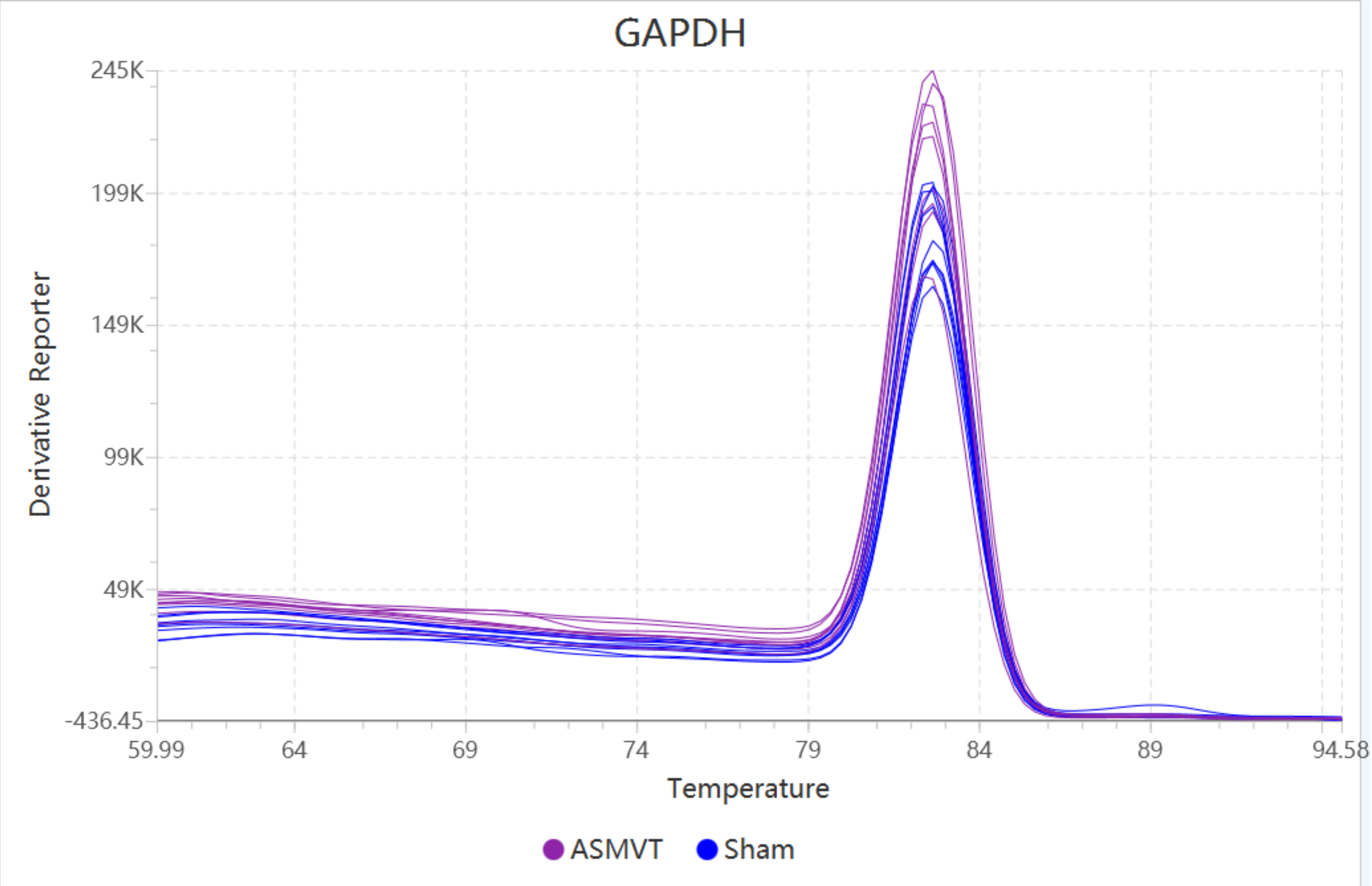

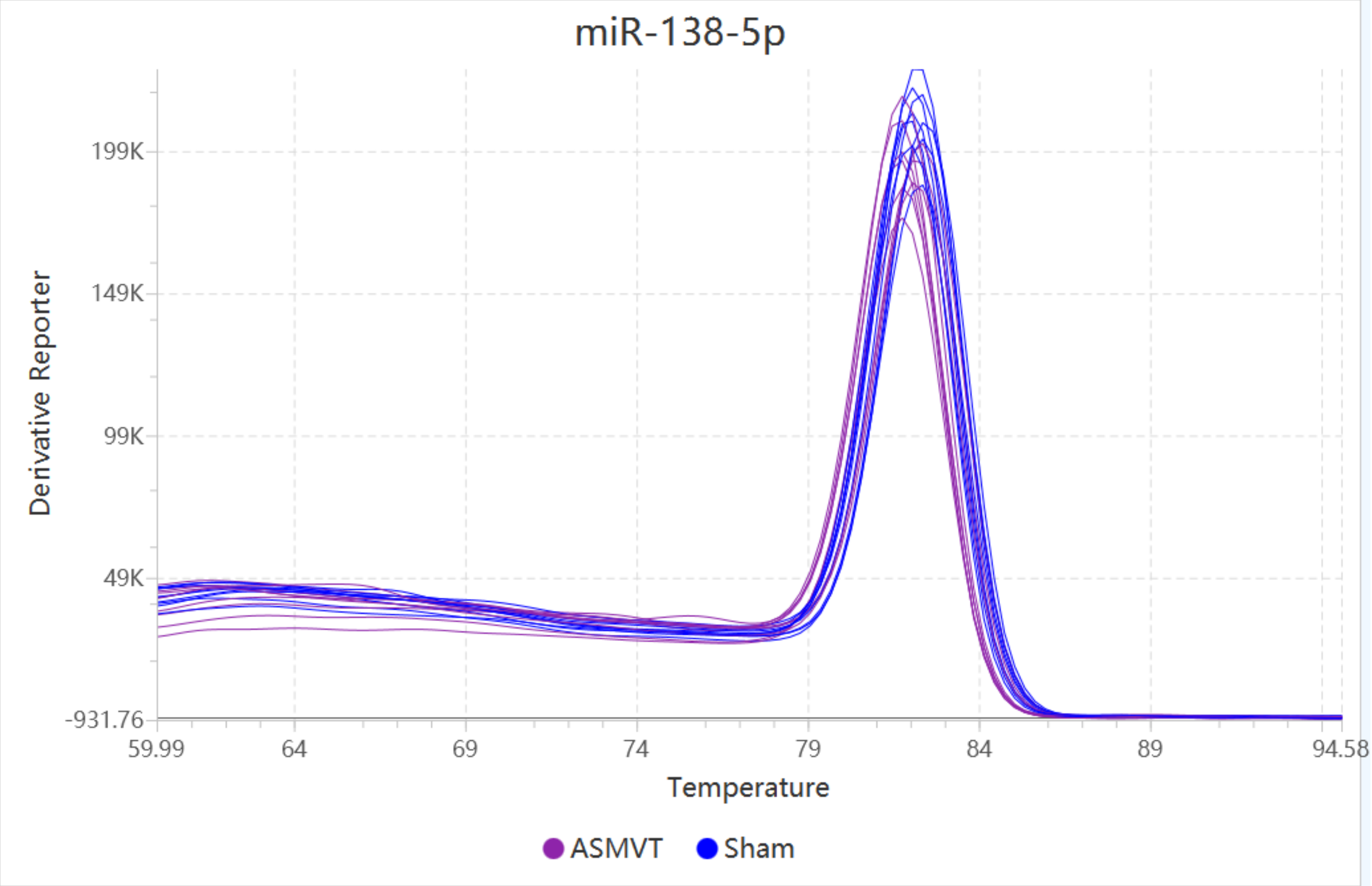

Run Method

Block Type	96-Well 0.2-mL Block
Sample Volume	20.0
Cover Temperature	105.0
Run mode	FAST

Stage	Collection Flag	Ramp Rate	Temperature	Hold Time	Starting Cycle	Auto Delta Temperature	Auto Delta Hold Time
Hold Stage	false	2.74°C/sec	95.0°C	20	-	-	-
PCR Stage (40 cycles)	false	2.74°C/sec	95.0°C	1	-	-	-
	true	2.12°C/sec	60.0°C	20	-	-	-
Melt Stage	false	2.74°C/sec	95.0°C	1	-	-	-
	false	2.12°C/sec	60.0°C	20	-	-	-
	true	0.15°C/sec	95.0°C	1	-	-	-

Primary Analysis Settings

General

PCR Stage/Step	Stage 2, Step 2
Quantification Cycle Method	Baseline Threshold

Target	Auto Threshold	Threshold	Auto Baseline	Baseline Start	Baseline End
DEFAULT	Yes	AUTO	Yes	AUTO	AUTO

Melt

Melt Stage/Step	Stage 3, Step 3

Target	Multi Peak	Threshold Type	Peak Level (%)	Peak Height
DEFAULT	Yes	Percentage	10	-
GAPDH	Yes	Percentage	10	-

QC Alerts

Curve Quality Alert Enabled	No
Results Quality Alert Enabled	Yes

Advanced

Set the Delta-Rn below which curves will be considered Non-Amplified	No
Primary Analysis Variant	N/A

Relative Quantification Settings

General

RQ Min/Max Calculations	Confidence Level (95.0)
Max Allowed EqCq Mean	40
Include Adjusted EqCq Mean	No
Analysis Type	Singleplex

Endo Controls

Normalization Type	Specific endogenous control

Target	Endogenous Control	Auto	Efficiency(%)
GAPDH	Yes	Yes	AUTO

References

Reference Sample	Sham

Relative Quantification Results (Sample)

Sample	Target	EqCq Mean	Adjusted EqCq Mean	ΔEqCq Mean	ΔEqCq SD	ΔEqCq SE	ΔΔEqCq	RQ	RQ Min	RQ Max
Sham	GAPDH	17.567	17.567	-	-	-	-	-	-	-
Sham	miR-138-5p	24.958	24.958	7.39	0.311	0.104	-	1	0.859	1.165
ASMVT	GAPDH	17.644	17.644	-	-	-	-	-	-	-
ASMVT	miR-138-5p	28.047	28.047	10.403	0.226	0.075	3.013	0.124	0.111	0.138

Relative Quantification Plot

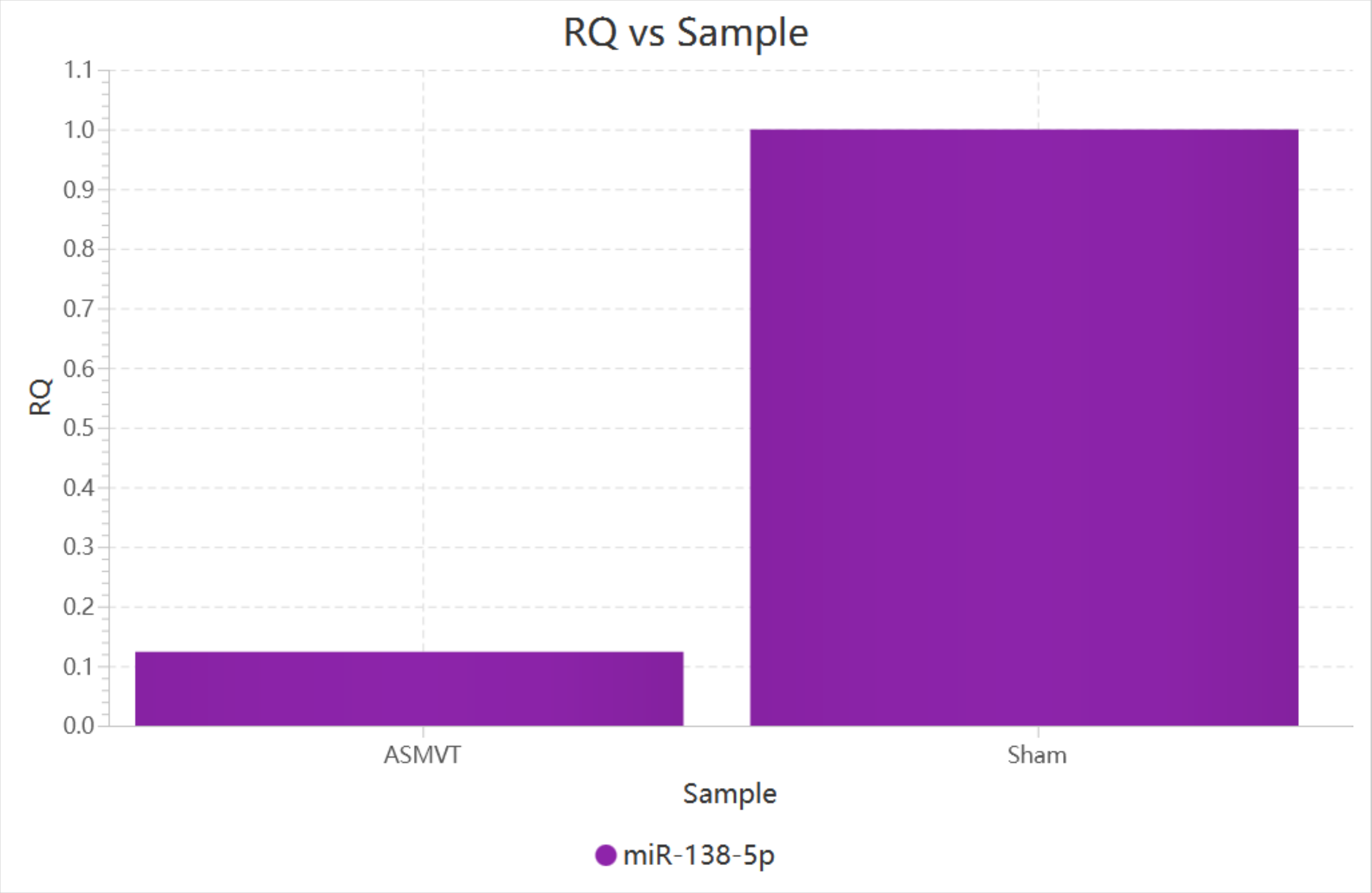

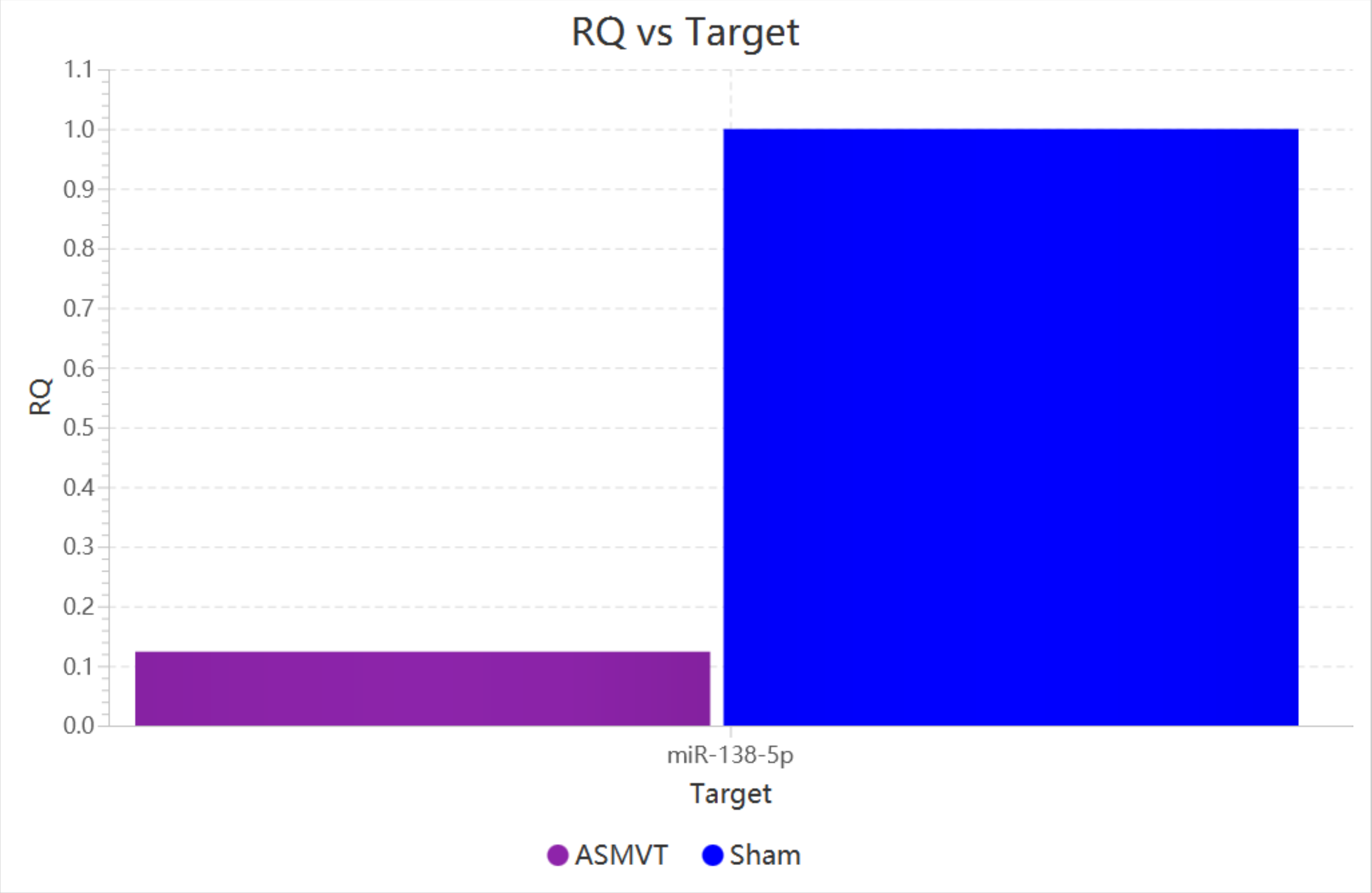

- End of Report -
