# Supplementary material for: miR-138-5p ameliorates intestinal barrier disruption caused by acute superior mesenteric vein thrombosis injury by inhibiting the NLRP3/HMGB1 axis
Source: PeerJ. 2024 Feb 21;12:e16692. doi: 10.7717/peerj.16692 (PMC10893868; doi:10.7717/peerj.16692)
Supplement: Supplemental Information 6 [file peerj-12-16692-s006.zip › original data-figure 2/image/2C/Results_Report_2023-08-17-144946.pdf]

Plate Results Report

A22-2_Copy.eds

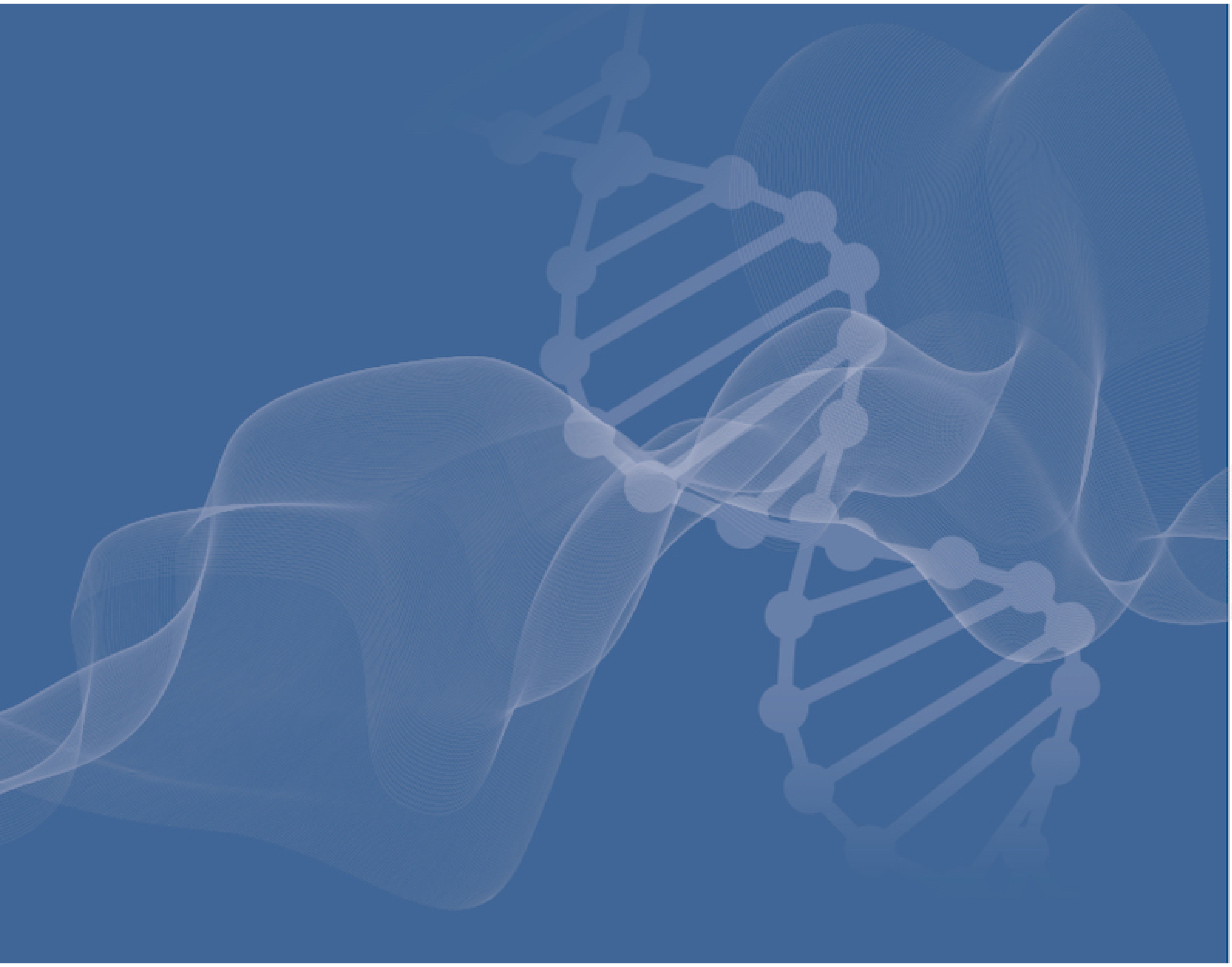

Summary

Property	Details
Bar Code	-
File Name	A22-2_Copy.eds
Run Start Date/Time	Dec 1, 2021 10:16:57 AM
Run End Date/Time	Dec 1, 2021 11:13:01 AM
Run Duration	56 minutes, and 4 seconds
Operator	DEFAULT
Instrument Name	SVT004
Instrument Type	QuantStudio™ 3 System
Instrument Serial Number	SVT004
Block Type	96-Well 0.2-mL
Block Serial Number	41145627
Heated Cover Serial Number	N/A
PCR Stage/Step Number	Stage 2, Step 2
Melt Stage Number	Stage 3
Quantification Cycle Method	Baseline Threshold
Comment	-
Software Name and Version	Design & Analysis Software v2.6.0
Plugin Name and Version	Primary Analysis v1.7.0, Relative Quantification v1.5.0
Analysis Date/Time	Jul 17, 2023 2:48:40 PM

Well Table

Well	Sample	Target	Task	Cq	Cq Confi dence	Amp Score	Amp Status	Cq Thres hold	Baseline Start/End	Melt Temp
A1	NC	GAPDH	Unknown	17.76	0.988	1.819	AMP	1.316	3-11	82.507
A2	NC	GAPDH	Unknown	17.815	0.992	1.832	AMP	1.316	3-12	82.357
A3	NC	GAPDH	Unknown	17.576	0.991	1.835	AMP	1.316	3-10	82.504
A4	NC	miR-138-5p	Unknown	27.879	0.988	1.862	AMP	1.897	3-20	82.355
A5	NC	miR-138-5p	Unknown	27.786	0.993	1.871	AMP	1.897	3-19	82.505
A6	NC	miR-138-5p	Unknown	27.521	0.988	1.87	AMP	1.897	3-18	82.505
A7	miR-138 mim	GAPDH	Unknown	17.241	0.991	1.84	AMP	1.316	3-10	82.653
A8	miR-138 mim	GAPDH	Unknown	17.105	0.989	1.834	AMP	1.316	3-10	82.653
A9	miR-138 mim	GAPDH	Unknown	17.38	0.986	1.84	AMP	1.316	3-11	82.655
A10	miR-138 mim	miR-138-5p	Unknown	25.157	0.985	1.846	AMP	1.897	3-18	82.505
A11	miR-138 mim	miR-138-5p	Unknown	24.957	0.99	1.849	AMP	1.897	3-16	82.506
A12	miR-138 mim	miR-138-5p	Unknown	25.257	0.982	1.822	AMP	1.897	3-18	82.804
B1	NC	GAPDH	Unknown	17.666	0.992	1.832	AMP	1.316	3-11	82.507
B2	NC	GAPDH	Unknown	17.611	0.993	1.841	AMP	1.316	3-10	82.357
B3	NC	GAPDH	Unknown	17.625	0.993	1.837	AMP	1.316	3-9	82.504
B4	NC	miR-138-5p	Unknown	27.699	0.992	1.872	AMP	1.897	3-20	82.355
B5	NC	miR-138-5p	Unknown	27.595	0.989	1.867	AMP	1.897	3-17	82.356
B6	NC	miR-138-5p	Unknown	27.546	0.988	1.871	AMP	1.897	3-17	82.356
C1	NC	GAPDH	Unknown	17.704	0.993	1.84	AMP	1.316	3-11	82.507
C2	NC	GAPDH	Unknown	16.242	0.989	1.825	AMP	1.316	3-10	82.805
C3	NC	GAPDH	Unknown	17.022	0.986	1.838	AMP	1.316	3-11	82.504
C4	NC	miR-138-5p	Unknown	27.632	0.989	1.869	AMP	1.897	3-20	82.206
C5	NC	miR-138-5p	Unknown	27.689	0.99	1.862	AMP	1.897	3-17	82.207
C6	NC	miR-138-5p	Unknown	27.943	0.988	1.852	AMP	1.897	3-18	82.356
D1	miR-NC mim	GAPDH	Unknown	16.531	0.986	1.82	AMP	1.316	3-10	82.954
D2	miR-NC mim	GAPDH	Unknown	17.412	0.991	1.835	AMP	1.316	3-10	82.507
D3	miR-NC mim	GAPDH	Unknown	17.308	0.991	1.845	AMP	1.316	3-11	82.504
D4	miR-NC mim	miR-138-5p	Unknown	27.822	0.988	1.858	AMP	1.897	3-19	82.057
D5	miR-NC mim	miR-138-5p	Unknown	27.747	0.99	1.867	AMP	1.897	3-18	82.207
D6	miR-NC mim	miR-138-5p	Unknown	27.861	0.984	1.849	AMP	1.897	3-18	82.207

Well	Sample	Target	Task	Cq	Cq Confidence	Amp Score	Amp Status	Cq Threshold	Baseline Start/End	Melt Temp
E1	miR-NC mim	GAPDH	Unknown	17.442	0.991	1.829	AMP	1.316	3-10	82.805
E2	miR-NC mim	GAPDH	Unknown	16.745	0.988	1.847	AMP	1.316	3-11	82.805
E3	miR-NC mim	GAPDH	Unknown	16.508	0.992	1.833	AMP	1.316	3-10	82.654
E4	miR-NC mim	miR-138-5p	Unknown	27.92	0.991	1.861	AMP	1.897	3-18	82.057
E5	miR-NC mim	miR-138-5p	Unknown	27.934	0.988	1.859	AMP	1.897	3-19	82.207
E6	miR-NC mim	miR-138-5p	Unknown	27.921	0.988	1.854	AMP	1.897	3-18	82.207
F1	miR-NC mim	GAPDH	Unknown	16.779	0.989	1.83	AMP	1.316	3-10	83.252
F2	miR-NC mim	GAPDH	Unknown	16.604	0.987	1.842	AMP	1.316	3-10	82.954
F3	miR-NC mim	GAPDH	Unknown	17.324	0.992	1.848	AMP	1.316	3-10	82.504
F4	miR-NC mim	miR-138-5p	Unknown	27.91	0.992	1.863	AMP	1.897	3-16	82.057
F5	miR-NC mim	miR-138-5p	Unknown	28.063	0.986	1.86	AMP	1.897	3-18	82.207
F6	miR-NC mim	miR-138-5p	Unknown	27.993	0.986	1.861	AMP	1.897	3-18	82.207
G1	miR-138 mim	GAPDH	Unknown	16.704	0.99	1.832	AMP	1.316	3-10	83.401
G2	miR-138 mim	GAPDH	Unknown	16.746	0.993	1.839	AMP	1.316	3-9	83.103
G3	miR-138 mim	GAPDH	Unknown	16.587	0.988	1.843	AMP	1.316	3-8	82.654
G4	miR-138 mim	miR-138-5p	Unknown	24.801	0.991	1.865	AMP	1.897	3-17	82.206
G5	miR-138 mim	miR-138-5p	Unknown	24.998	0.992	1.859	AMP	1.897	3-16	82.207
G6	miR-138 mim	miR-138-5p	Unknown	24.896	0.992	1.866	AMP	1.897	3-17	82.356
H1	miR-138 mim	GAPDH	Unknown	16.774	0.98	1.729	AMP	1.316	3-10	83.252
H2	miR-138 mim	GAPDH	Unknown	16.794	0.989	1.844	AMP	1.316	3-10	83.103
H3	miR-138 mim	GAPDH	Unknown	17.388	0.991	1.848	AMP	1.316	3-11	82.504
H4	miR-138 mim	miR-138-5p	Unknown	24.763	0.992	1.871	AMP	1.897	3-17	82.355
H5	miR-138 mim	miR-138-5p	Unknown	24.901	0.993	1.87	AMP	1.897	3-17	82.356
H6	miR-138 mim	miR-138-5p	Unknown	24.938	0.993	1.862	AMP	1.897	3-16	82.505

Replicate Group Table

Sample	Target	No. of Replicates	Cq Mean	Cq SD
NC	GAPDH	9	17.447	0.507
NC	miR-138-5p	9	27.699	0.146
miR-138 mim	GAPDH	9	16.969	0.31
miR-138 mim	miR-138-5p	9	24.963	0.159
miR-NC mim	GAPDH	9	16.962	0.401
miR-NC mim	miR-138-5p	9	27.908	0.092

Plate Layout

	1	2	3	4	5	6	7	8	9	10	11	12
A	NC<br>GAPDH (17.76)	NC<br>GAPDH (17.815)	NC<br>GAPDH (17.576)	NC<br>miR-138-5p (27.879)	NC<br>miR-138-5p (27.786)	NC<br>miR-138-5p (27.521)	miR-138 mim<br>GAPDH (17.241)	miR-138 mim<br>GAPDH (17.105)	miR-138 mim<br>GAPDH (17.38)	miR-138 mim<br>miR-138-5p (25.157)	miR-138 mim<br>miR-138-5p (24.957)	miR-138 mim<br>miR-138-5p (25.257)
B	NC<br>GAPDH (17.666)	NC<br>GAPDH (17.611)	NC<br>GAPDH (17.625)	NC<br>miR-138-5p (27.699)	NC<br>miR-138-5p (27.595)	NC<br>miR-138-5p (27.546)						
C	NC<br>GAPDH (17.704)	NC<br>GAPDH (16.242)	NC<br>GAPDH (17.022)	NC<br>miR-138-5p (27.632)	NC<br>miR-138-5p (27.689)	NC<br>miR-138-5p (27.943)						
D	miR-NC mim<br>GAPDH (16.531)	miR-NC mim<br>GAPDH (17.412)	miR-NC mim<br>GAPDH (17.308)	miR-NC mim<br>miR-138-5p (27.822)	miR-NC mim<br>miR-138-5p (27.747)	miR-NC mim<br>miR-138-5p (27.861)						
E	miR-NC mim<br>GAPDH (17.442)	miR-NC mim<br>GAPDH (16.745)	miR-NC mim<br>GAPDH (16.508)	miR-NC mim<br>miR-138-5p (27.92)	miR-NC mim<br>miR-138-5p (27.934)	miR-NC mim<br>miR-138-5p (27.921)						
F	miR-NC mim<br>GAPDH (16.779)	miR-NC mim<br>GAPDH (16.604)	miR-NC mim<br>GAPDH (17.324)	miR-NC mim<br>miR-138-5p (27.91)	miR-NC mim<br>miR-138-5p (28.063)	miR-NC mim<br>miR-138-5p (27.993)						
G	miR-138 mim<br>GAPDH (16.704)	miR-138 mim<br>GAPDH (16.746)	miR-138 mim<br>GAPDH (16.587)	miR-138 mim<br>miR-138-5p (24.801)	miR-138 mim<br>miR-138-5p (24.998)	miR-138 mim<br>miR-138-5p (24.896)						
H	miR-138 mim<br>GAPDH (16.774)	miR-138 mim<br>GAPDH (16.794)	miR-138 mim<br>GAPDH (17.388)	miR-138 mim<br>miR-138-5p (24.763)	miR-138 mim<br>miR-138-5p (24.901)	miR-138 mim<br>miR-138-5p (24.938)						

Amplification Plot (dRn)

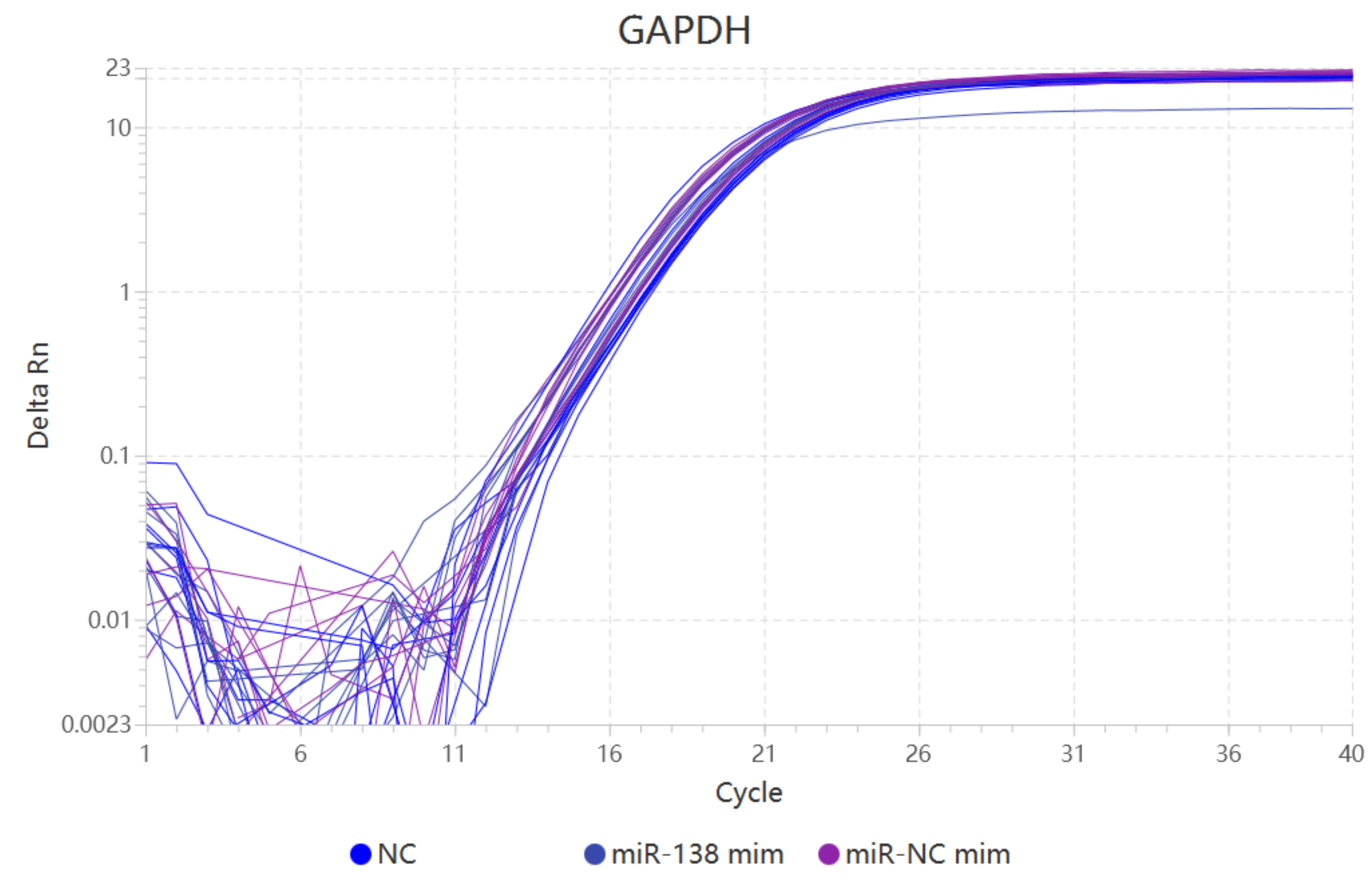

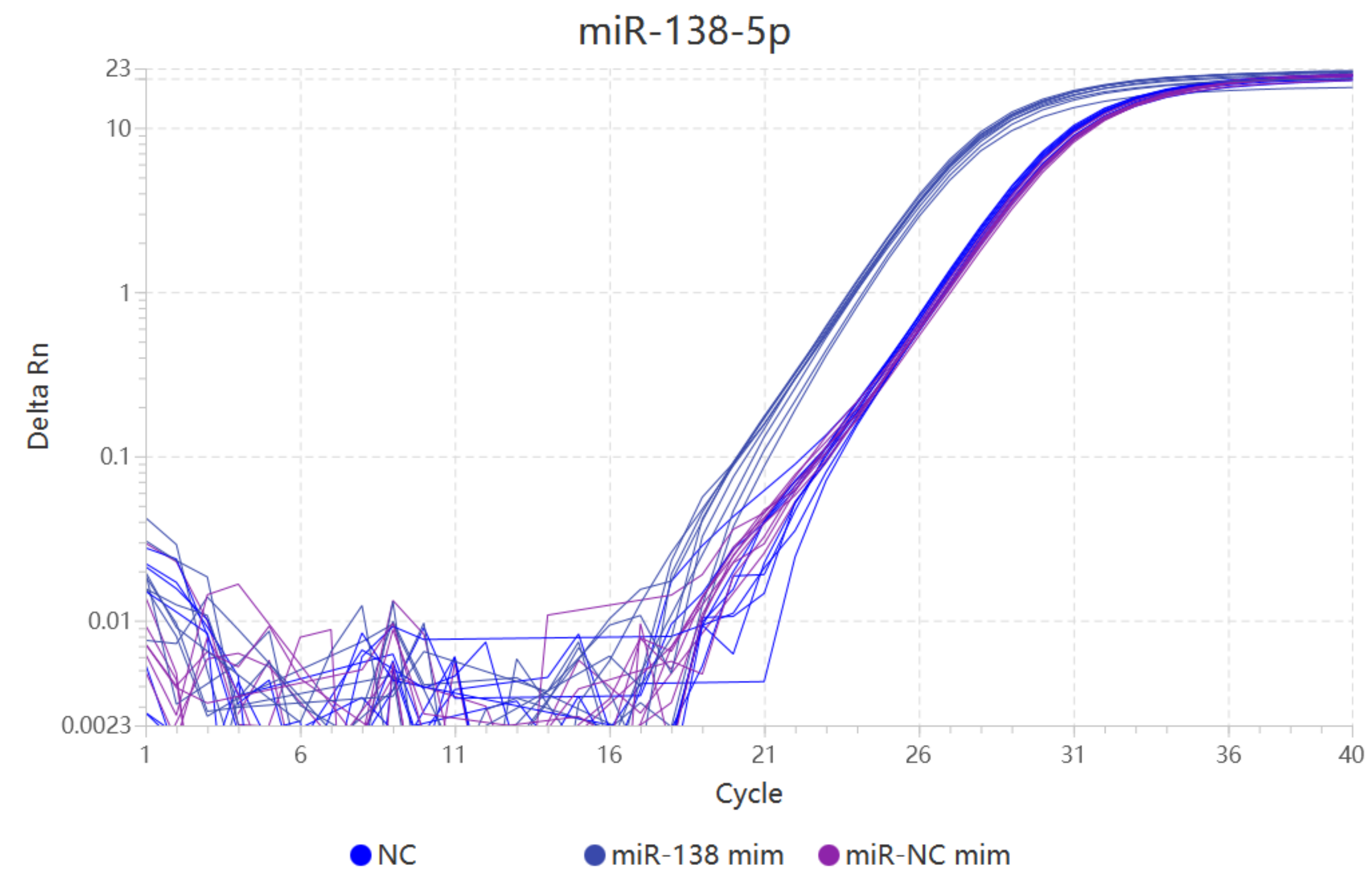

Amplification Plot (Rn)

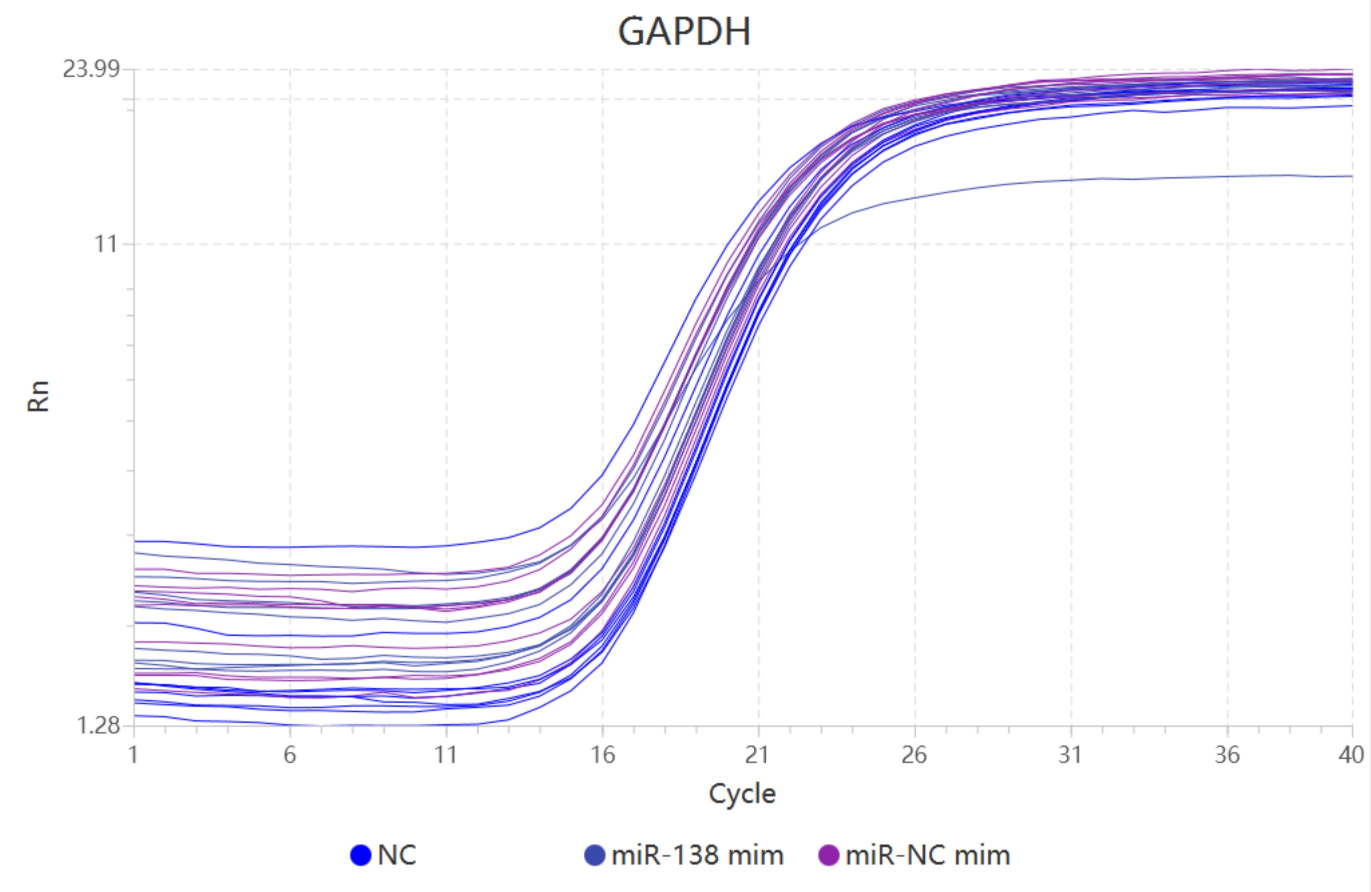

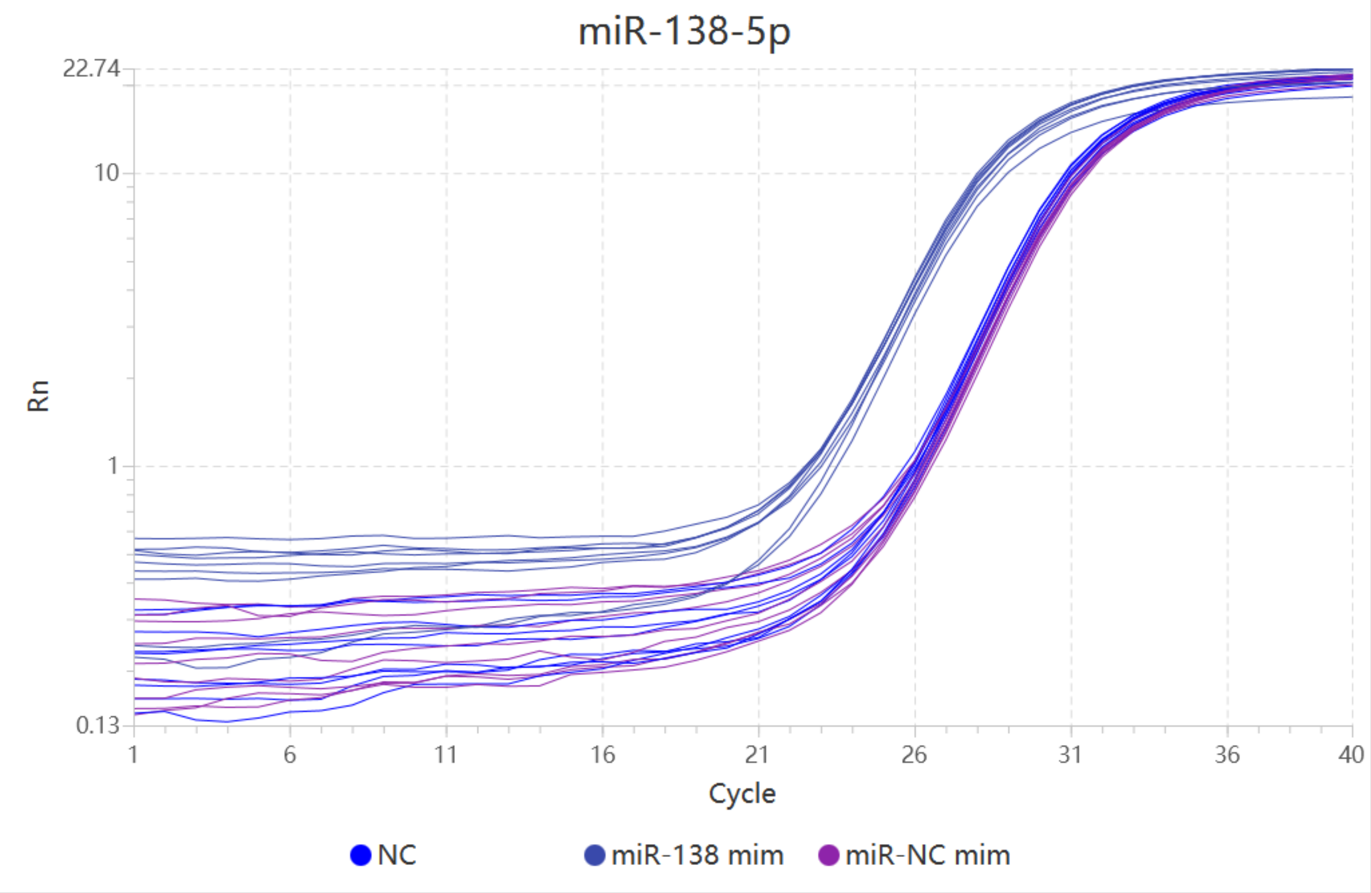

Melt Curve Plot

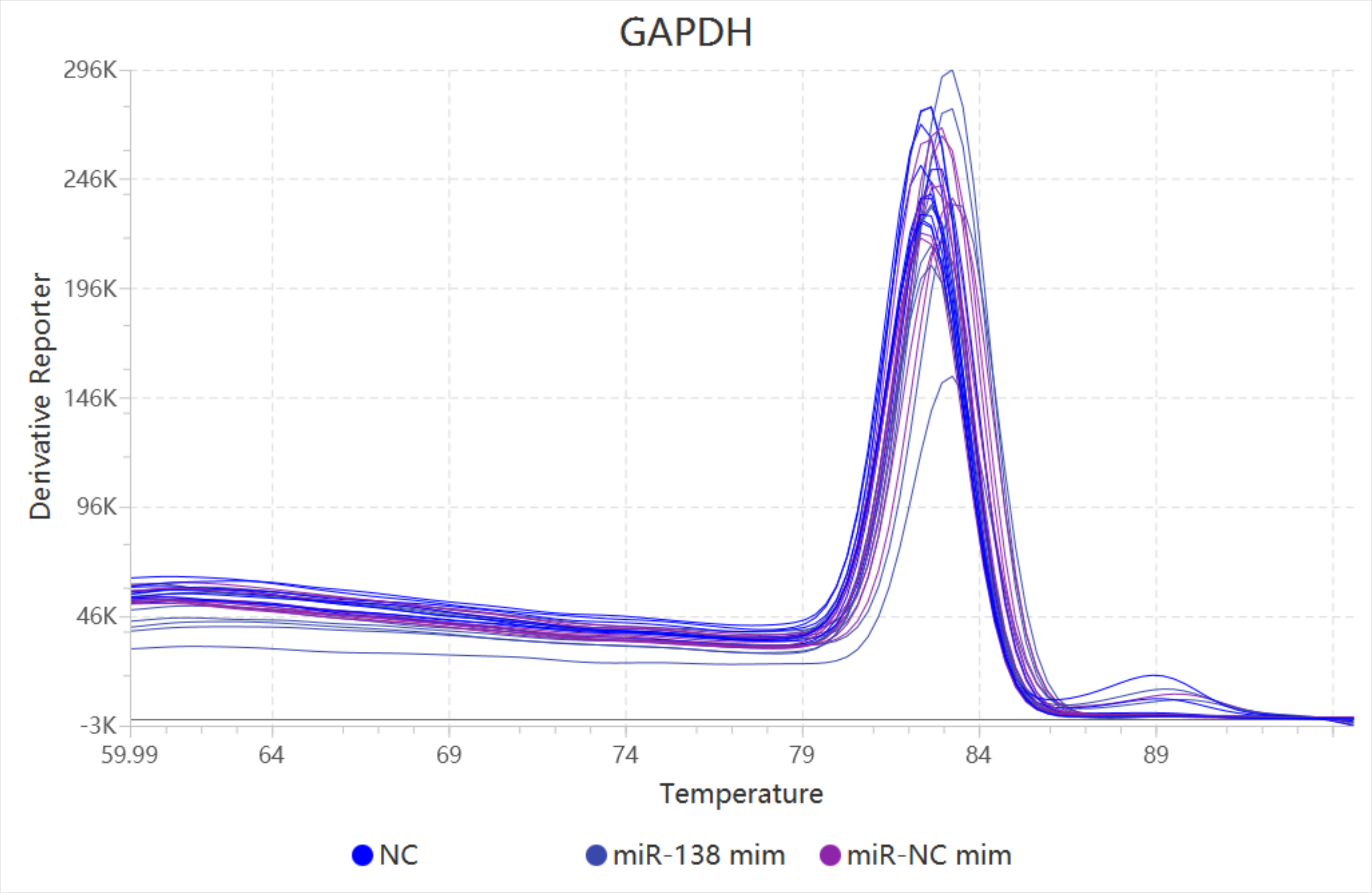

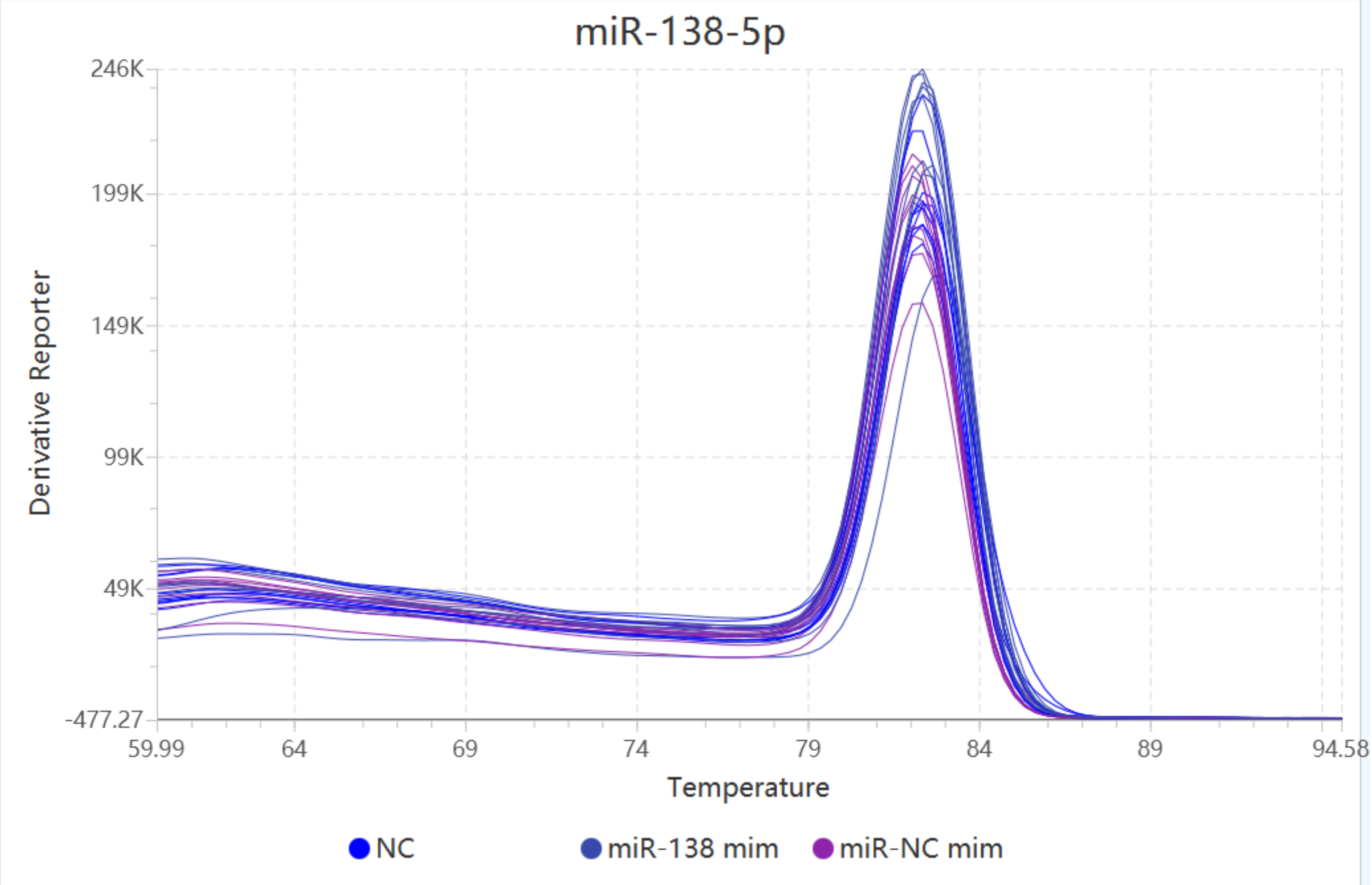

Run Method

Block Type	96-Well 0.2-mL Block
Sample Volume	20.0
Cover Temperature	105.0
Run mode	FAST

Stage	Collection Flag	Ramp Rate	Temperature	Hold Time	Starting Cycle	Auto Delta Temperature	Auto Delta Hold Time
Hold Stage	false	2.74°C/sec	95.0°C	20	-	-	-
PCR Stage (40 cycles)	false	2.74°C/sec	95.0°C	1	-	-	-
	true	2.12°C/sec	60.0°C	20	-	-	-
Melt Stage	false	2.74°C/sec	95.0°C	1	-	-	-
	false	2.12°C/sec	60.0°C	20	-	-	-
	true	0.15°C/sec	95.0°C	1	-	-	-

Primary Analysis Settings

General

PCR Stage/Step	Stage 2, Step 2
Quantification Cycle Method	Baseline Threshold

Target	Auto Threshold	Threshold	Auto Baseline	Baseline Start	Baseline End
DEFAULT	Yes	AUTO	Yes	AUTO	AUTO

Melt

Melt Stage/Step	Stage 3, Step 3

Target	Multi Peak	Threshold Type	Peak Level (%)	Peak Height
DEFAULT	Yes	Percentage	10	-
GAPDH	Yes	Percentage	10	-

QC Alerts

Curve Quality Alert Enabled	No
Results Quality Alert Enabled	Yes

Advanced

Set the Delta-Rn below which curves will be considered Non-Amplified	No
Primary Analysis Variant	N/A

Relative Quantification Settings

General

RQ Min/Max Calculations	Confidence Level (95.0)
Max Allowed EqCq Mean	40
Include Adjusted EqCq Mean	No
Analysis Type	Singleplex

Endo Controls

Normalization Type	Specific endogenous control

Target	Endogenous Control	Auto	Efficiency(%)
GAPDH	Yes	Yes	AUTO

References

Reference Sample	NC

Relative Quantification Results (Sample)

Sample	Target	EqCq Mean	Adjusted EqCq Mean	ΔEqCq Mean	ΔEqCq SD	ΔEqCq SE	ΔΔEqCq	RQ	RQ Min	RQ Max
NC	GAPDH	17.447	17.447	-	-	-	-	-	-	-
NC	miR-138-5p	27.699	27.699	10.252	0.527	0.176	-	1	0.772	1.295
miR-138 mim	GAPDH	16.969	16.969	-	-	-	-	-	-	-
miR-138 mim	miR-138-5p	24.963	24.963	7.994	0.349	0.116	-2.258	4.782	4.032	5.673
miR-NC mim	GAPDH	16.962	16.962	-	-	-	-	-	-	-
miR-NC mim	miR-138-5p	27.908	27.908	10.946	0.411	0.137	0.694	0.618	0.505	0.756

Relative Quantification Plot

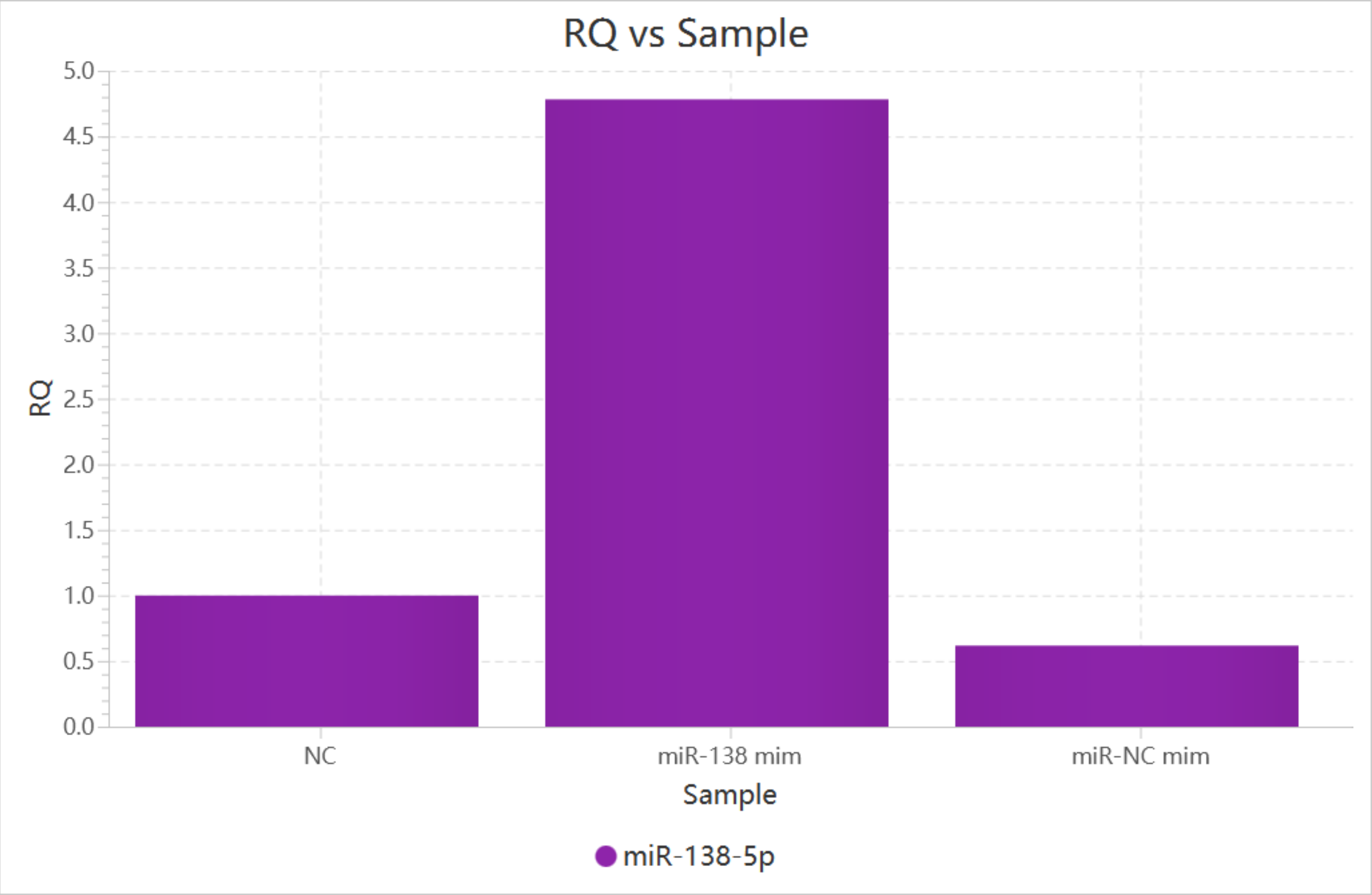

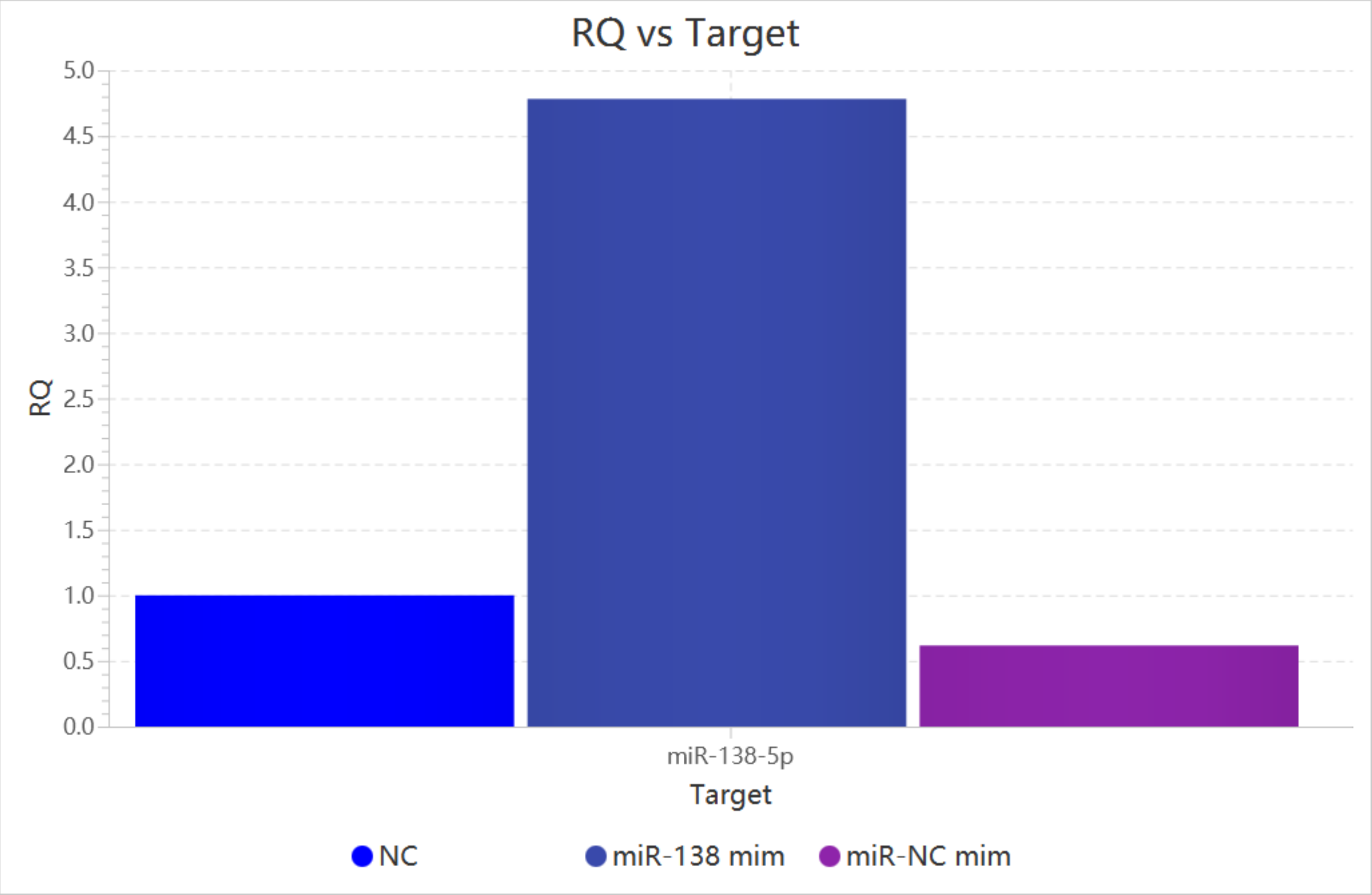

- End of Report -
