# Supplementary figures and images for: miR-138-5p ameliorates intestinal barrier disruption caused by acute superior mesenteric vein thrombosis injury by inhibiting the NLRP3/HMGB1 axis (part 2 of 2)
Source: PeerJ. 2024 Feb 21;12:e16692. doi: 10.7717/peerj.16692 (PMC10893868; doi:10.7717/peerj.16692)

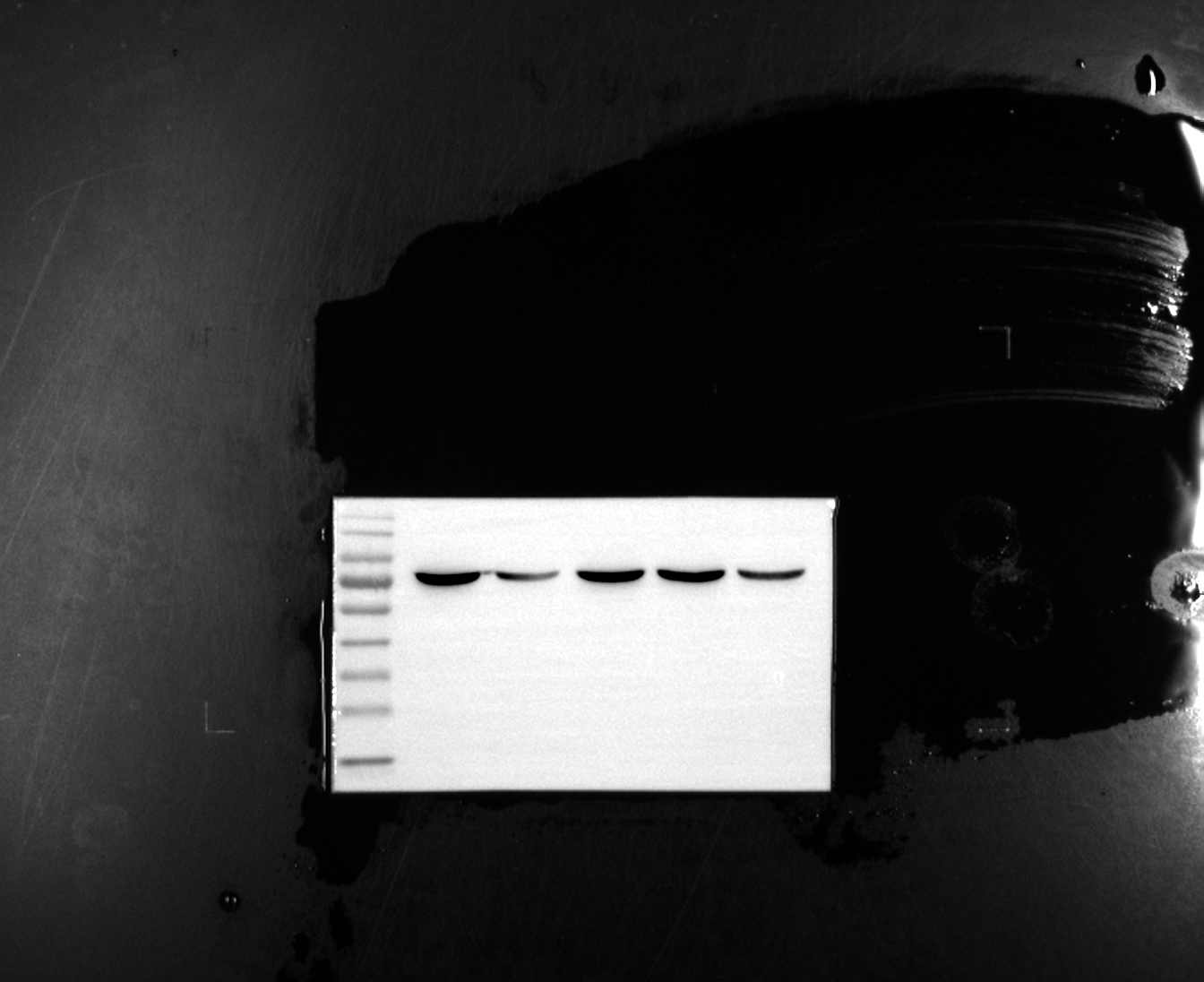

Supplement: Supplemental Information 10 [file peerj-12-16692-s010.zip › original data-figure 5/5B/2.Occludin.tif]

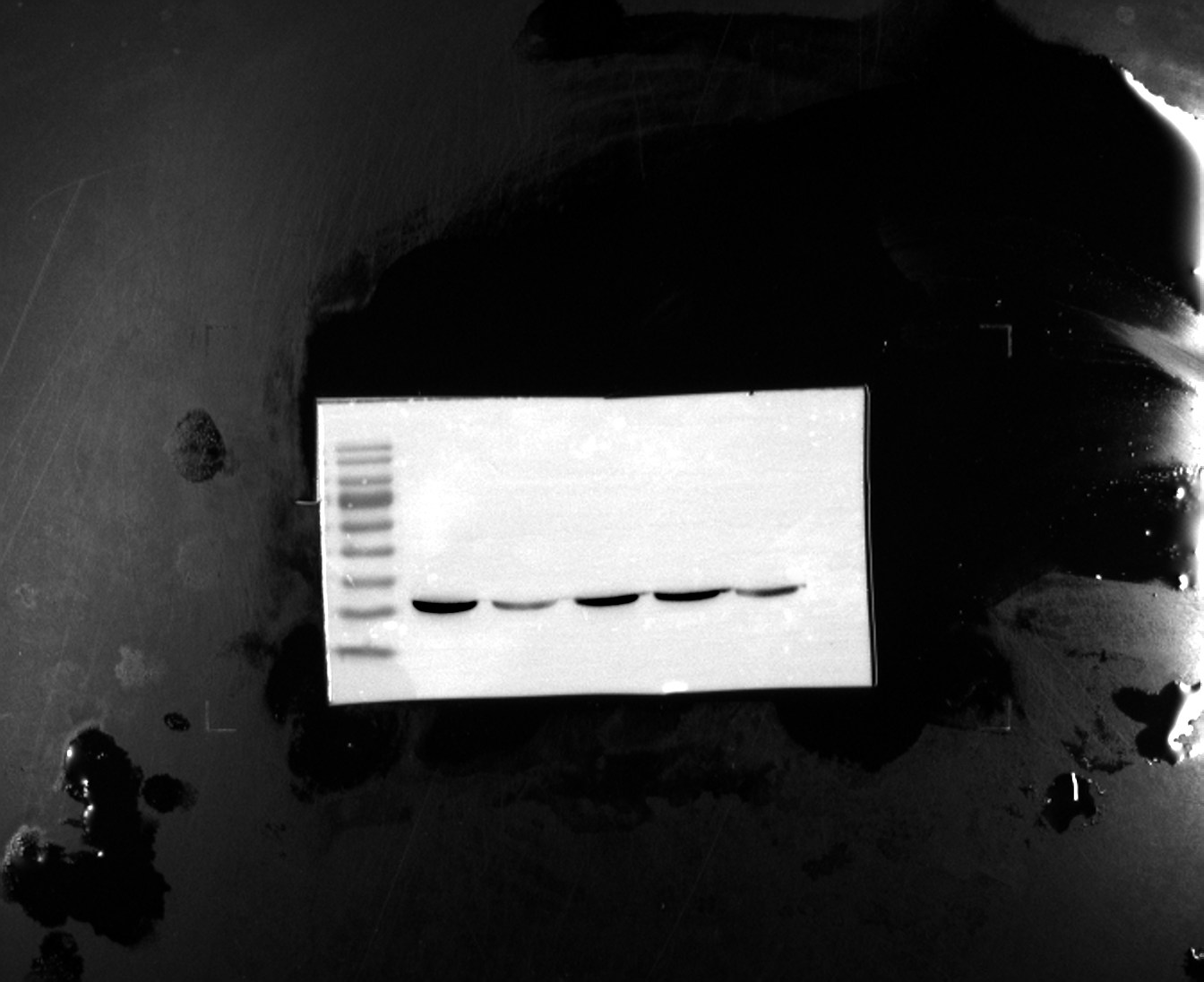

Supplement: Supplemental Information 10 [file peerj-12-16692-s010.zip › original data-figure 5/5B/3.Claudin-1.tif]

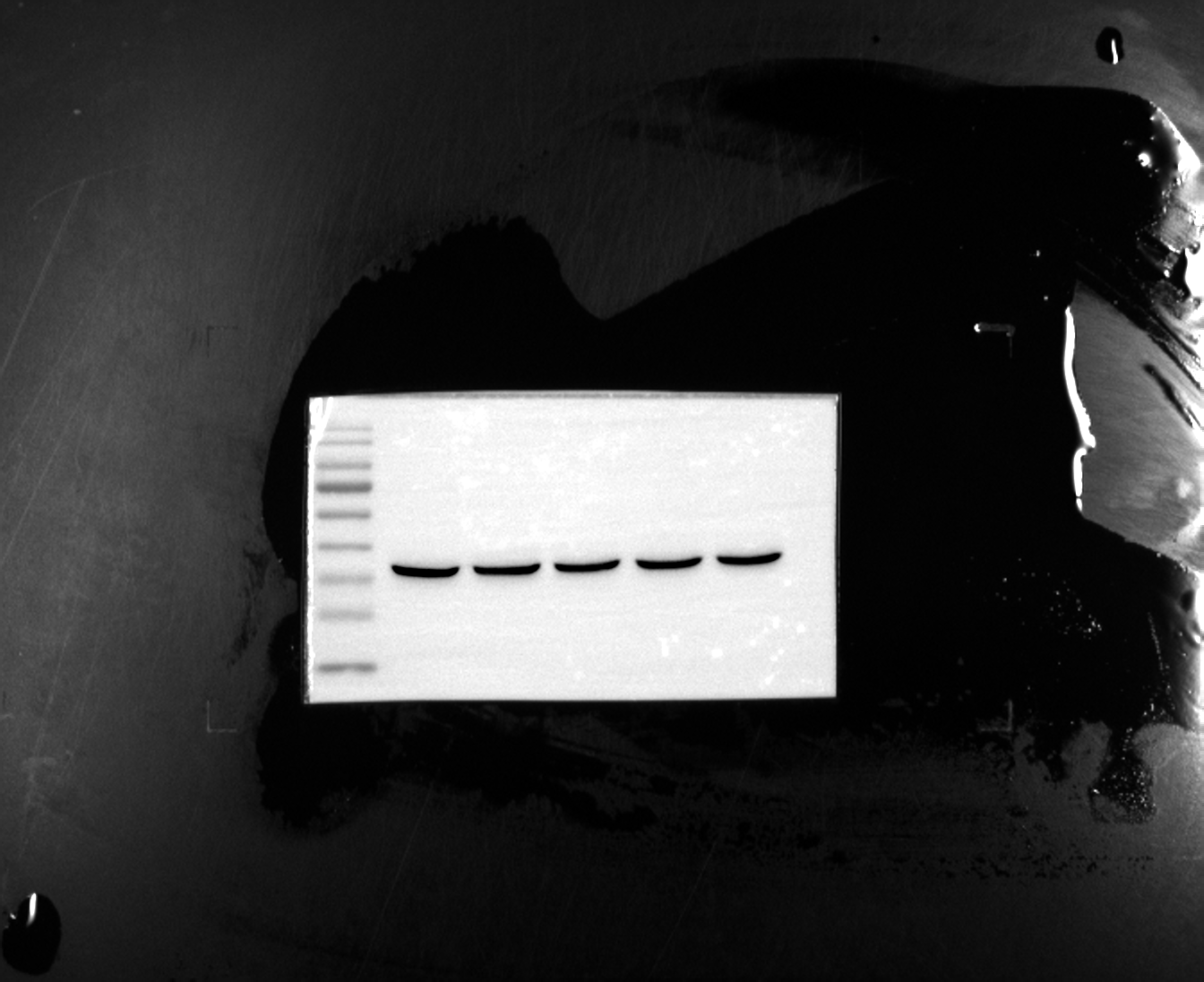

Supplement: Supplemental Information 10 [file peerj-12-16692-s010.zip › original data-figure 5/5B/4.GAPDH.tif]

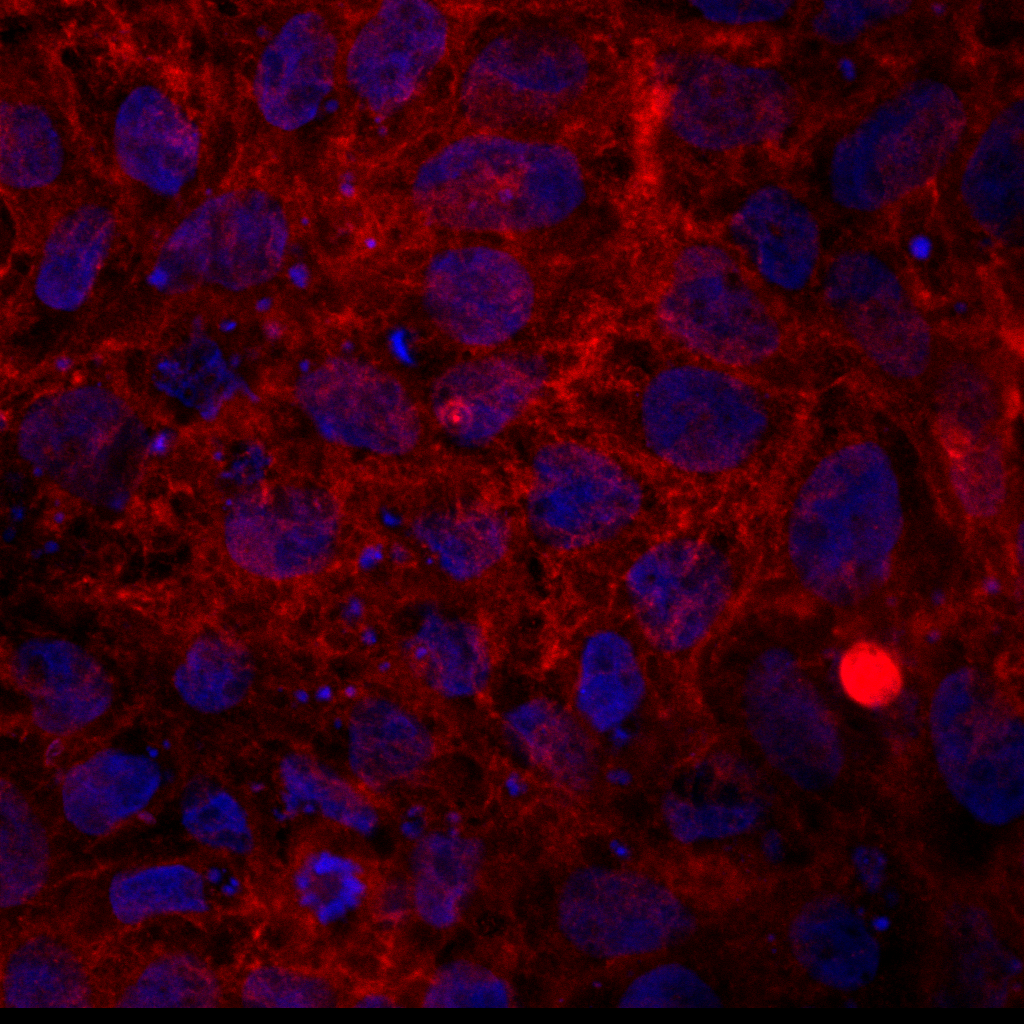

Supplement: Supplemental Information 10 [file peerj-12-16692-s010.zip › original data-figure 5/5C/NC/1.ZO-1.tif]

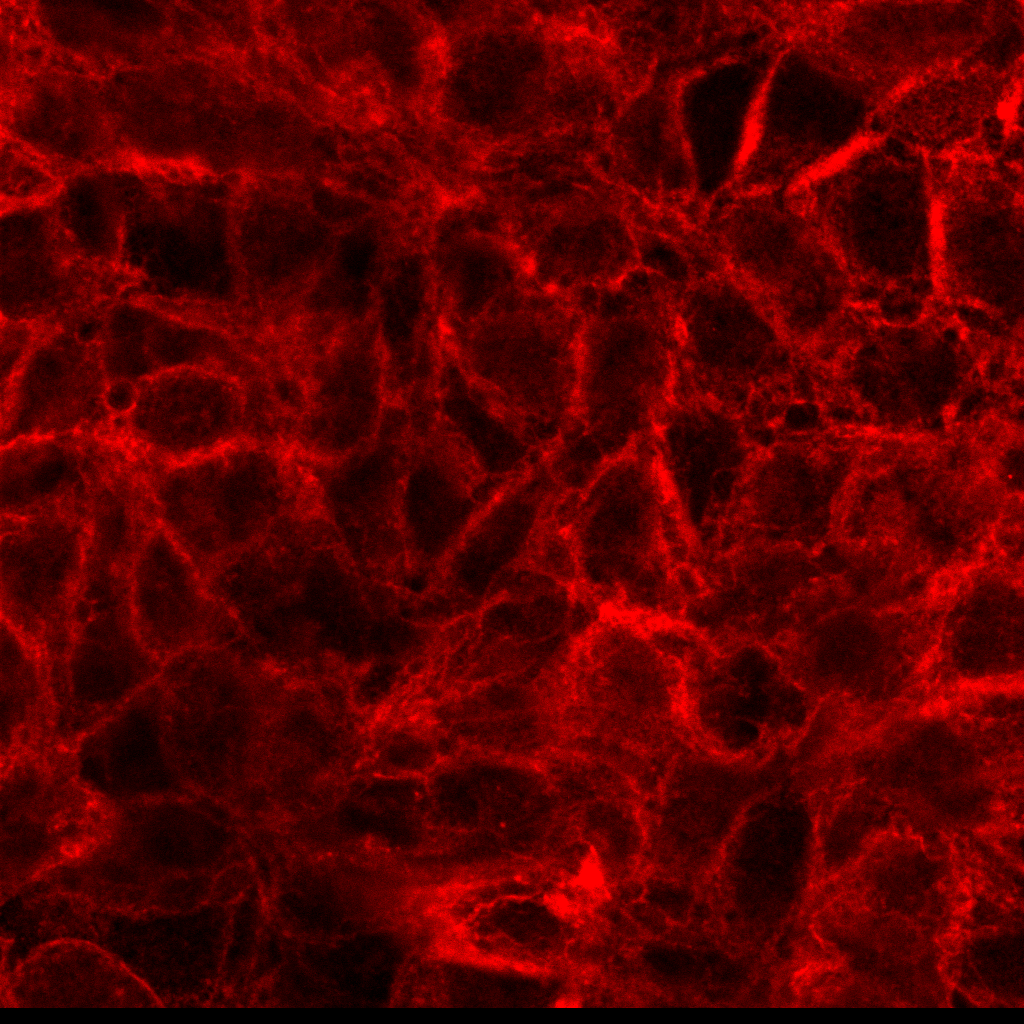

Supplement: Supplemental Information 10 [file peerj-12-16692-s010.zip › original data-figure 5/5C/NC/2.Occludin.tif]

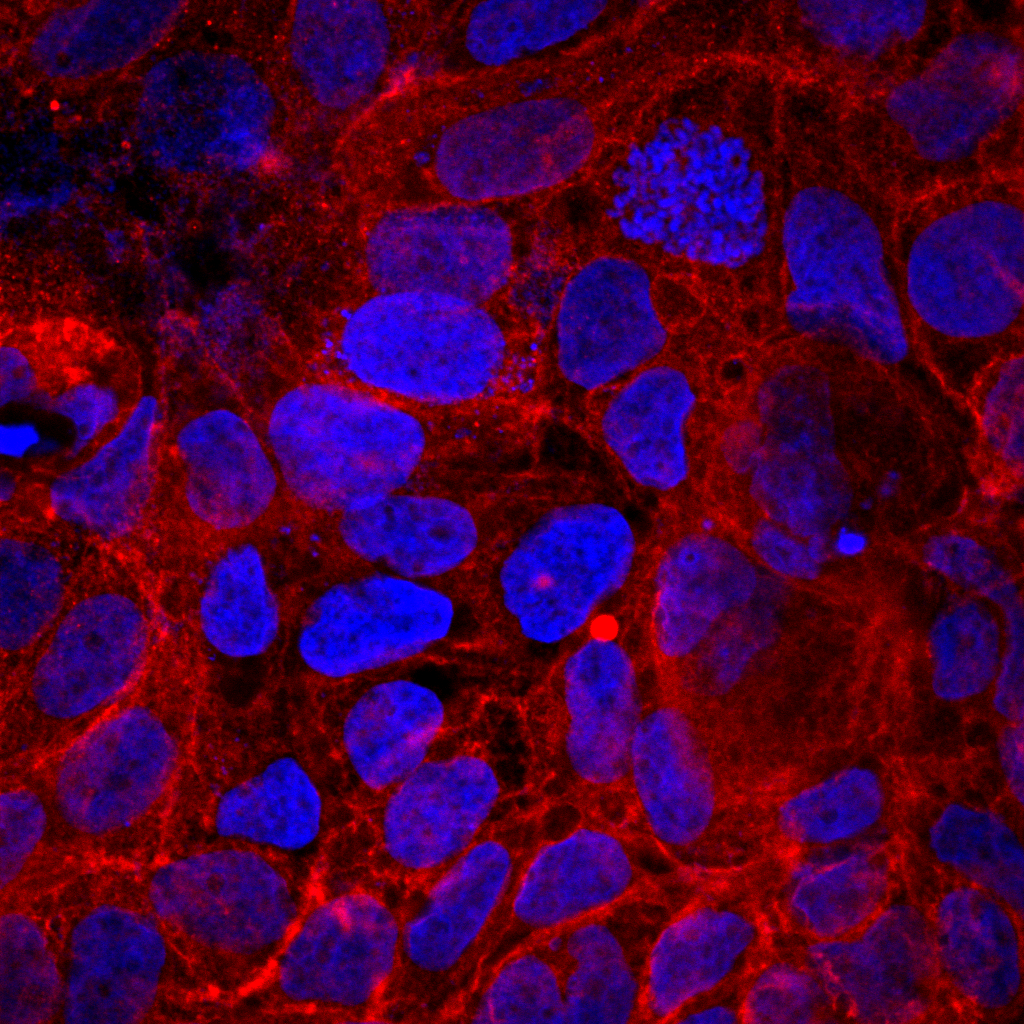

Supplement: Supplemental Information 10 [file peerj-12-16692-s010.zip › original data-figure 5/5C/NC/3.Claudin-1.tif]

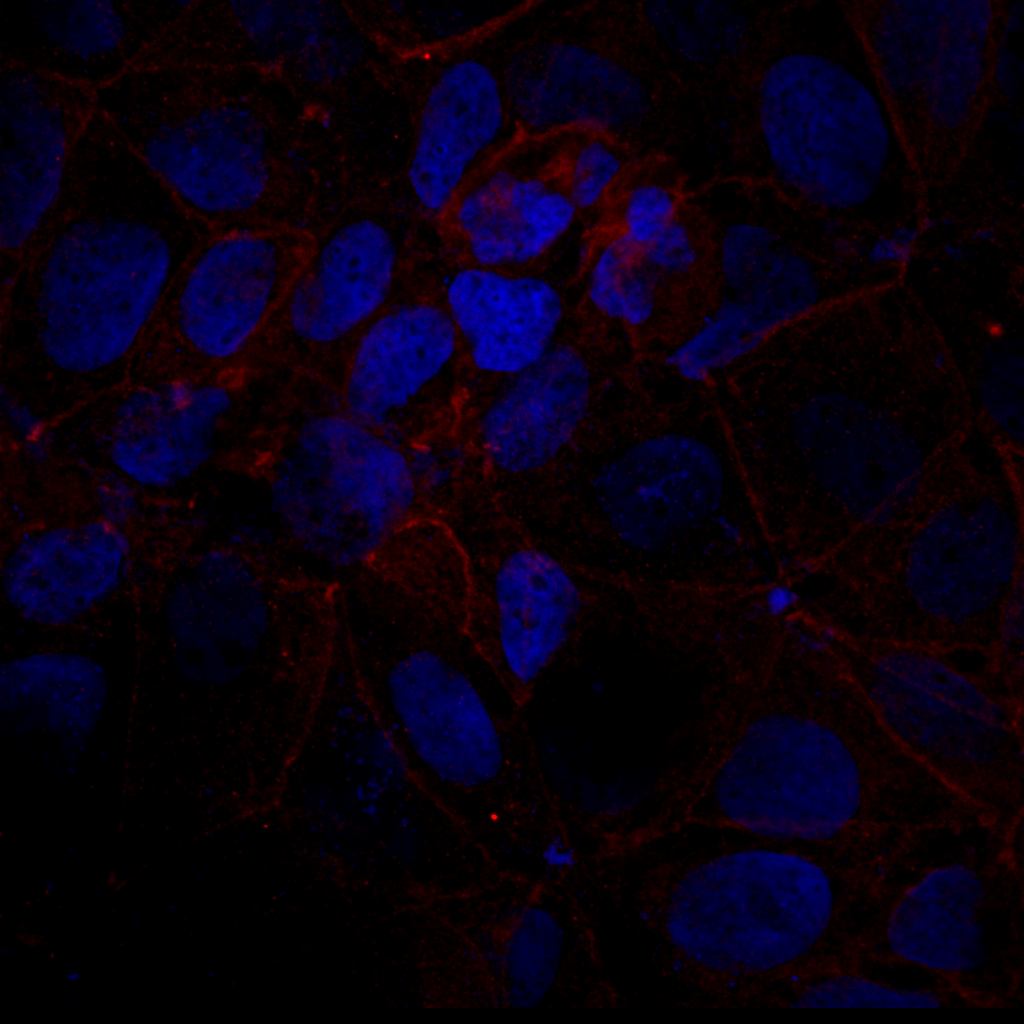

Supplement: Supplemental Information 10 [file peerj-12-16692-s010.zip › original data-figure 5/5C/miR-138 inhi/1.ZO-1.tif]

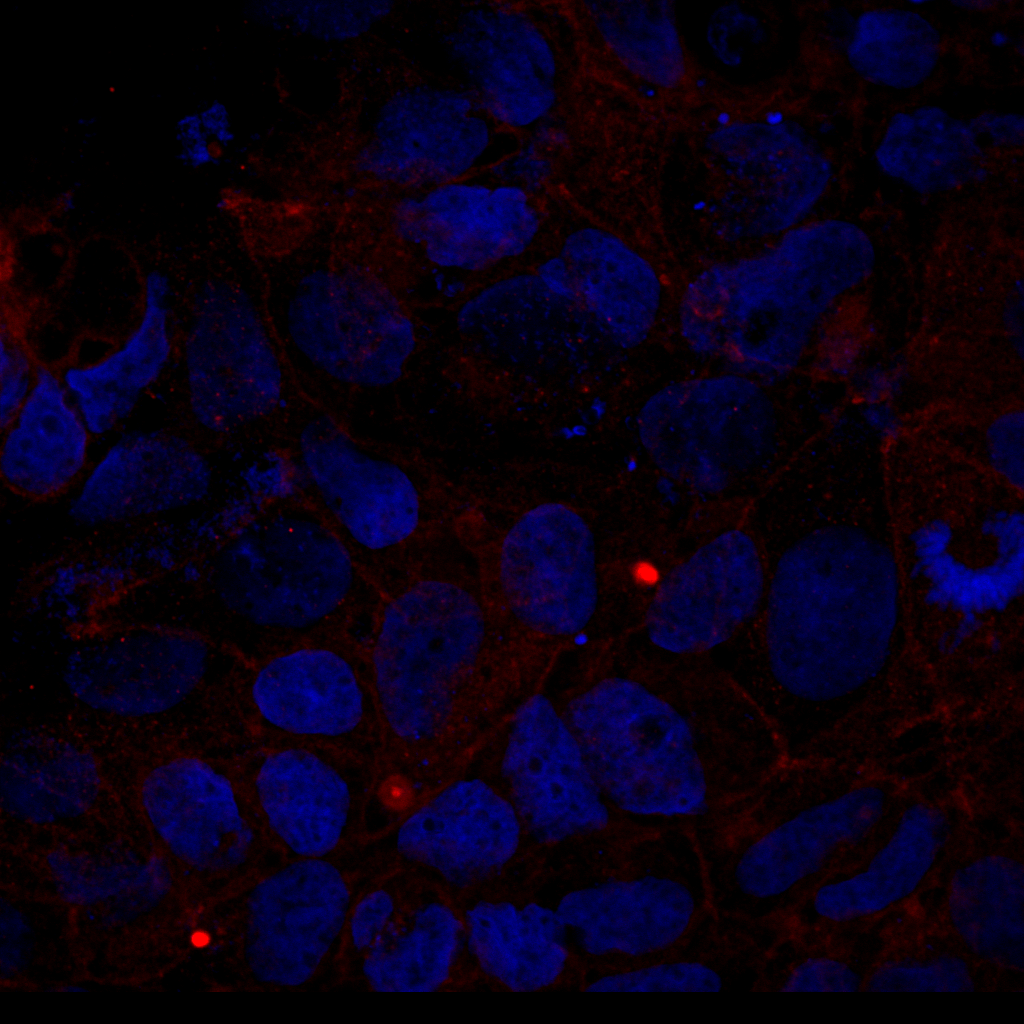

Supplement: Supplemental Information 10 [file peerj-12-16692-s010.zip › original data-figure 5/5C/miR-138 inhi/2.Occludin.tif]

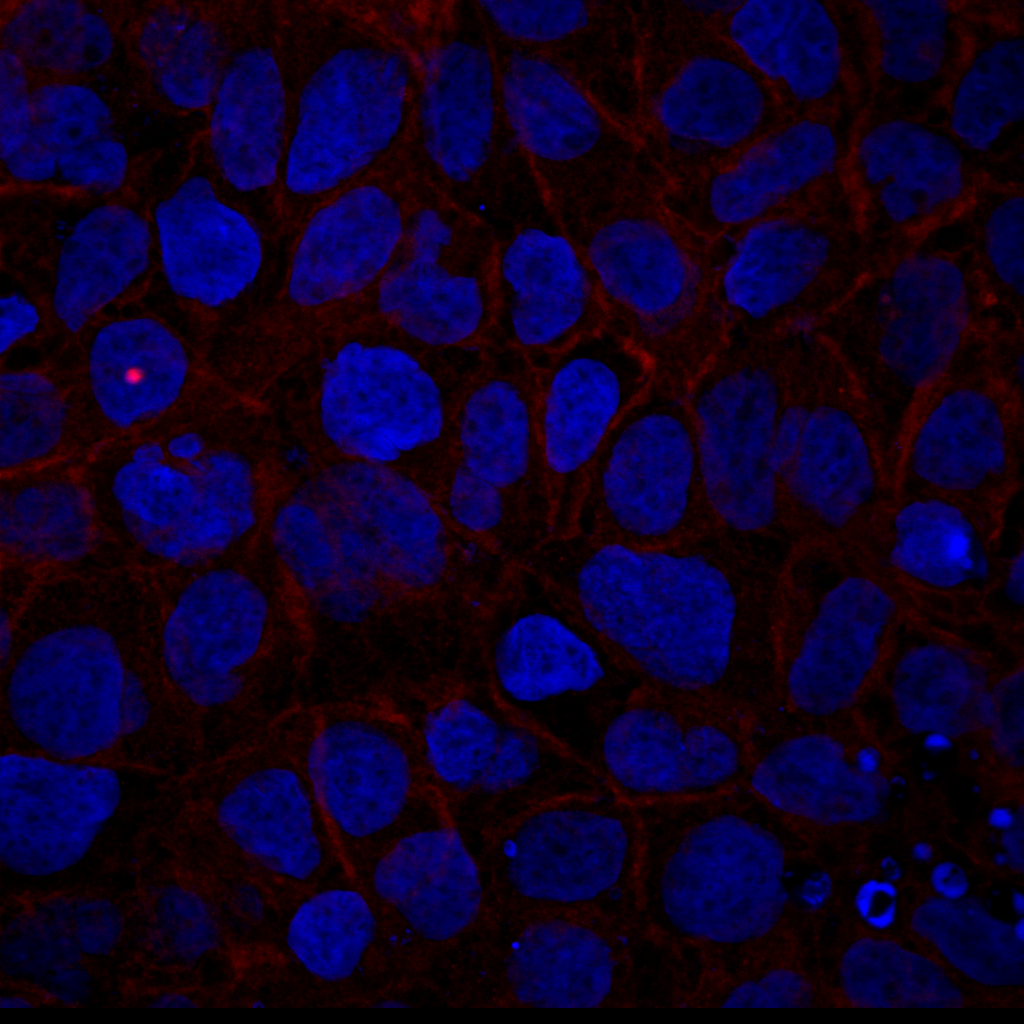

Supplement: Supplemental Information 10 [file peerj-12-16692-s010.zip › original data-figure 5/5C/miR-138 inhi/3.Claudin-1.tif]

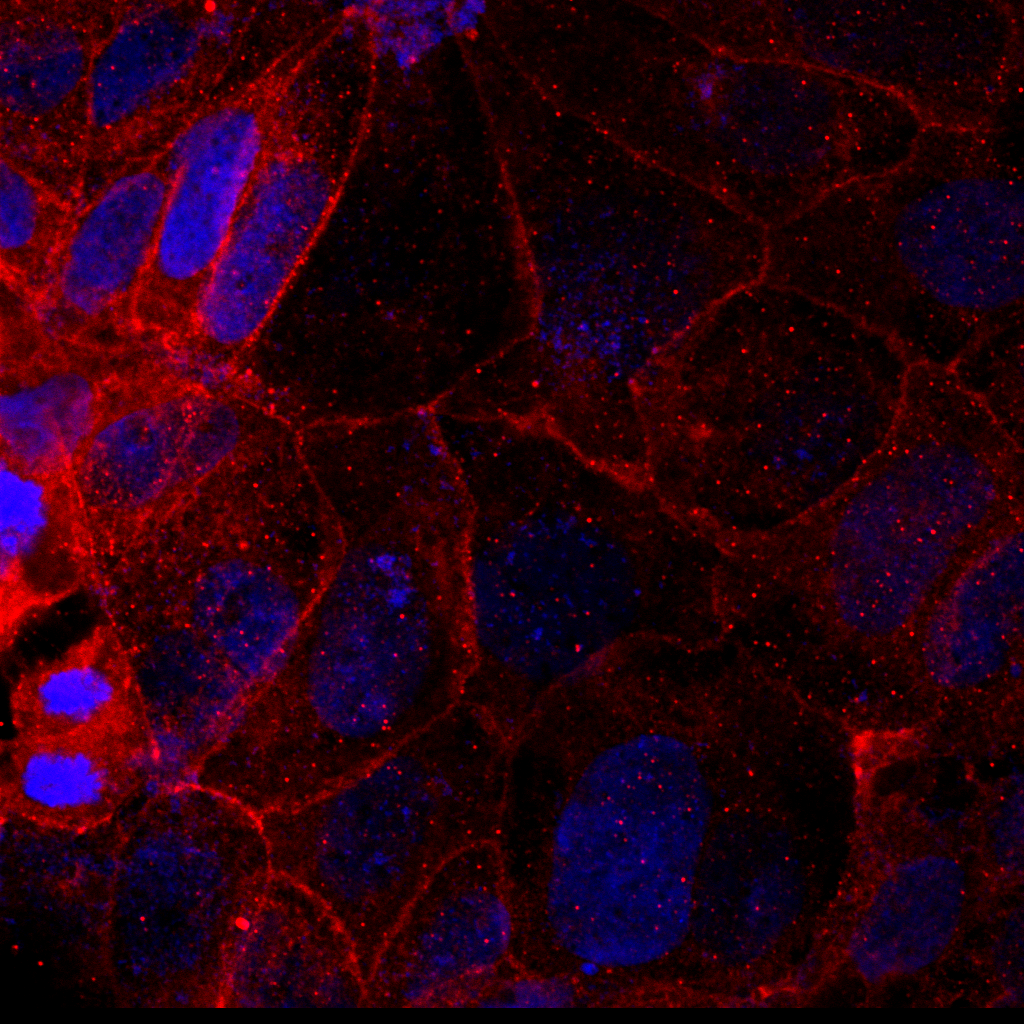

Supplement: Supplemental Information 10 [file peerj-12-16692-s010.zip › original data-figure 5/5C/miR-138 inhi+si-NLRP3/1.ZO-1.tif]

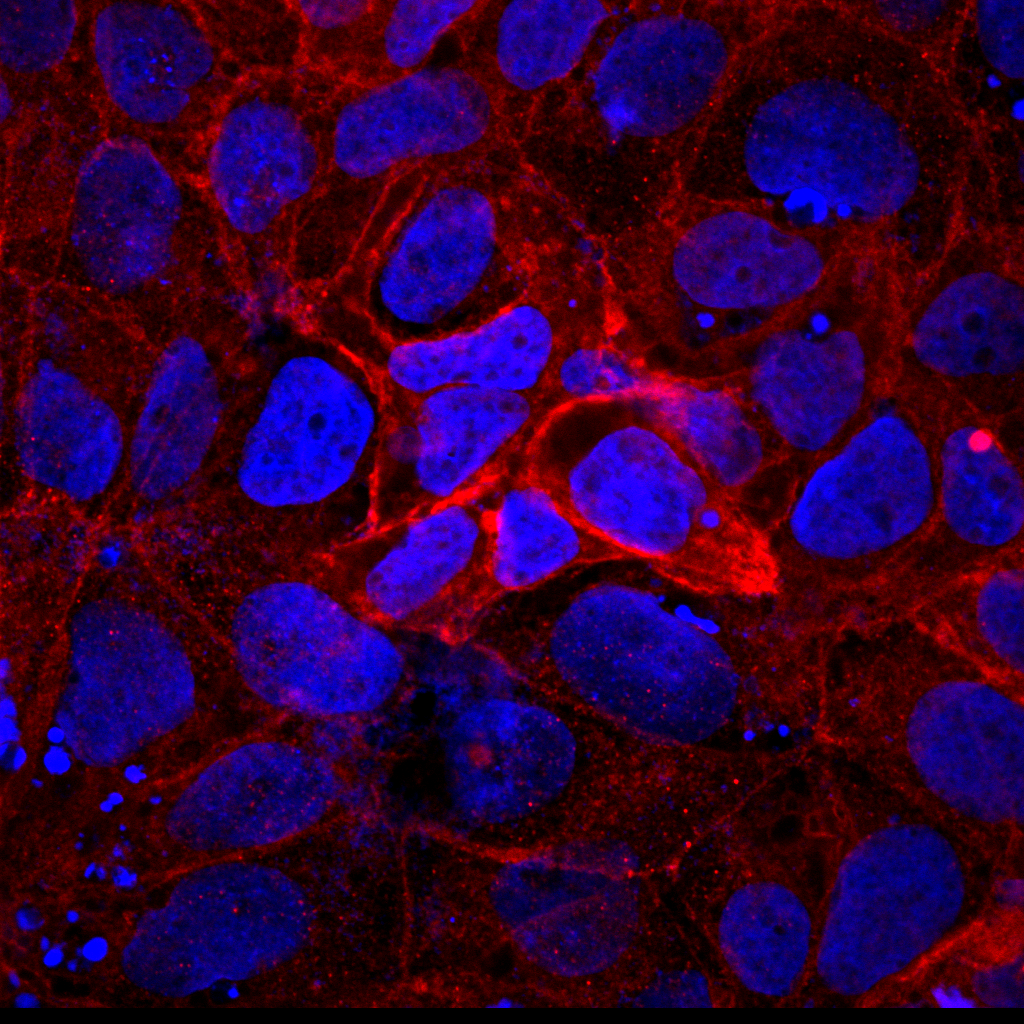

Supplement: Supplemental Information 10 [file peerj-12-16692-s010.zip › original data-figure 5/5C/miR-138 inhi+si-NLRP3/2.Occludin.tif]

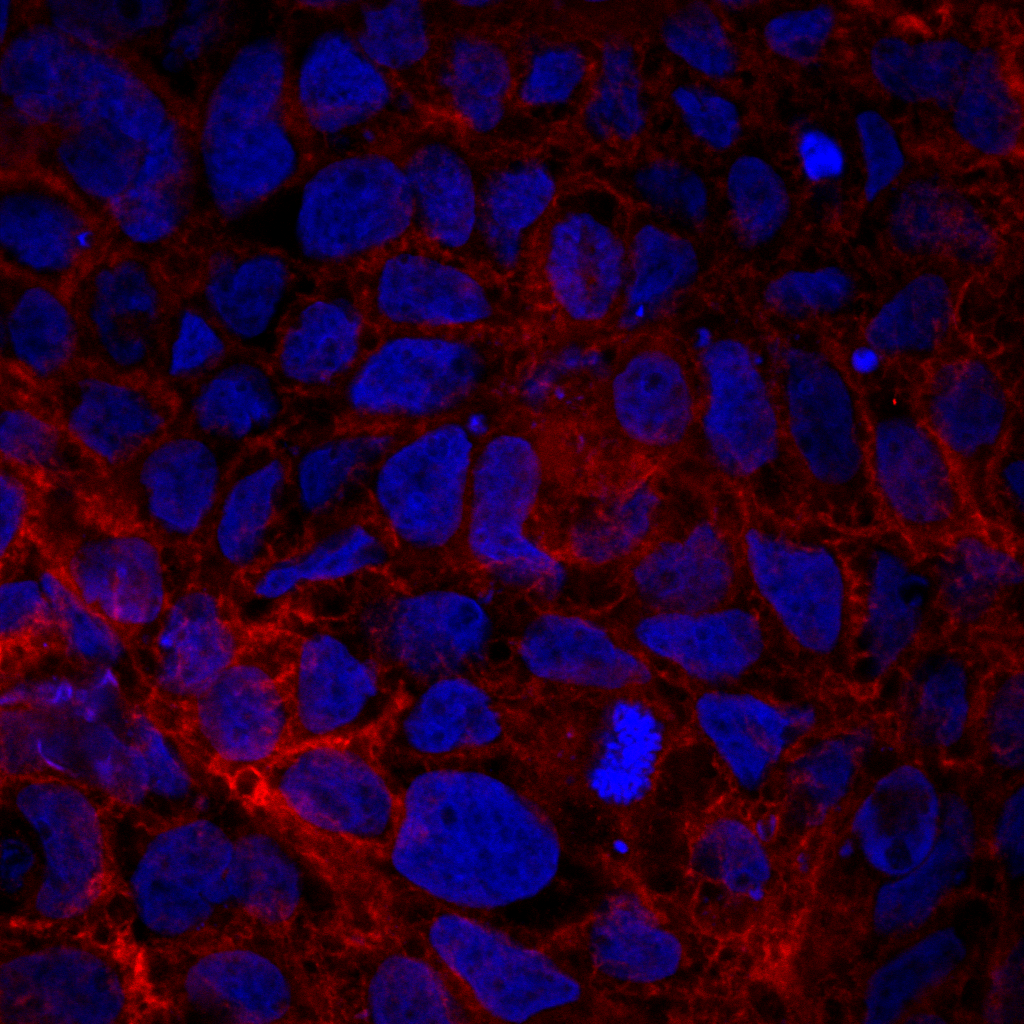

Supplement: Supplemental Information 10 [file peerj-12-16692-s010.zip › original data-figure 5/5C/miR-138 inhi+si-NLRP3/3.Claudin-1.tif]

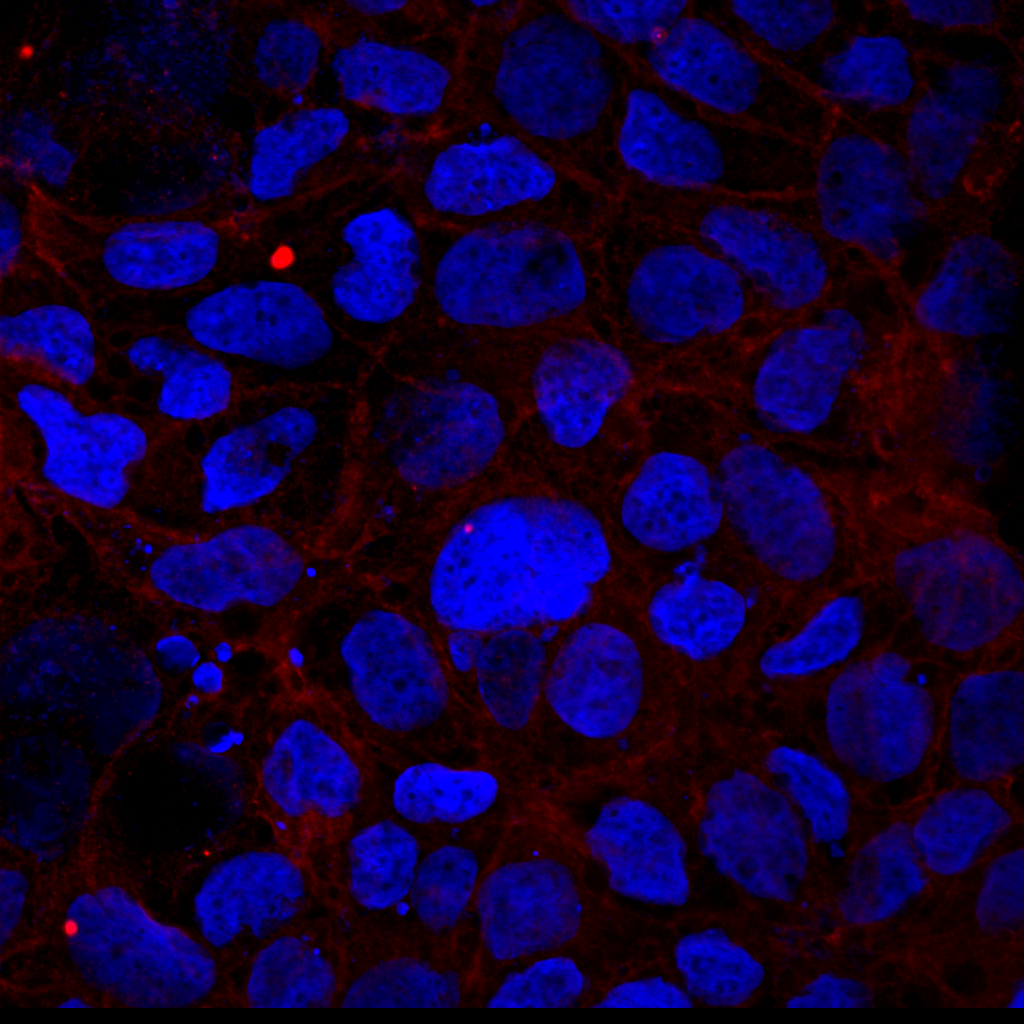

Supplement: Supplemental Information 10 [file peerj-12-16692-s010.zip › original data-figure 5/5C/miR-138 inhi+si-NLRP3+OE-HMGB1/1.ZO-1.tif]

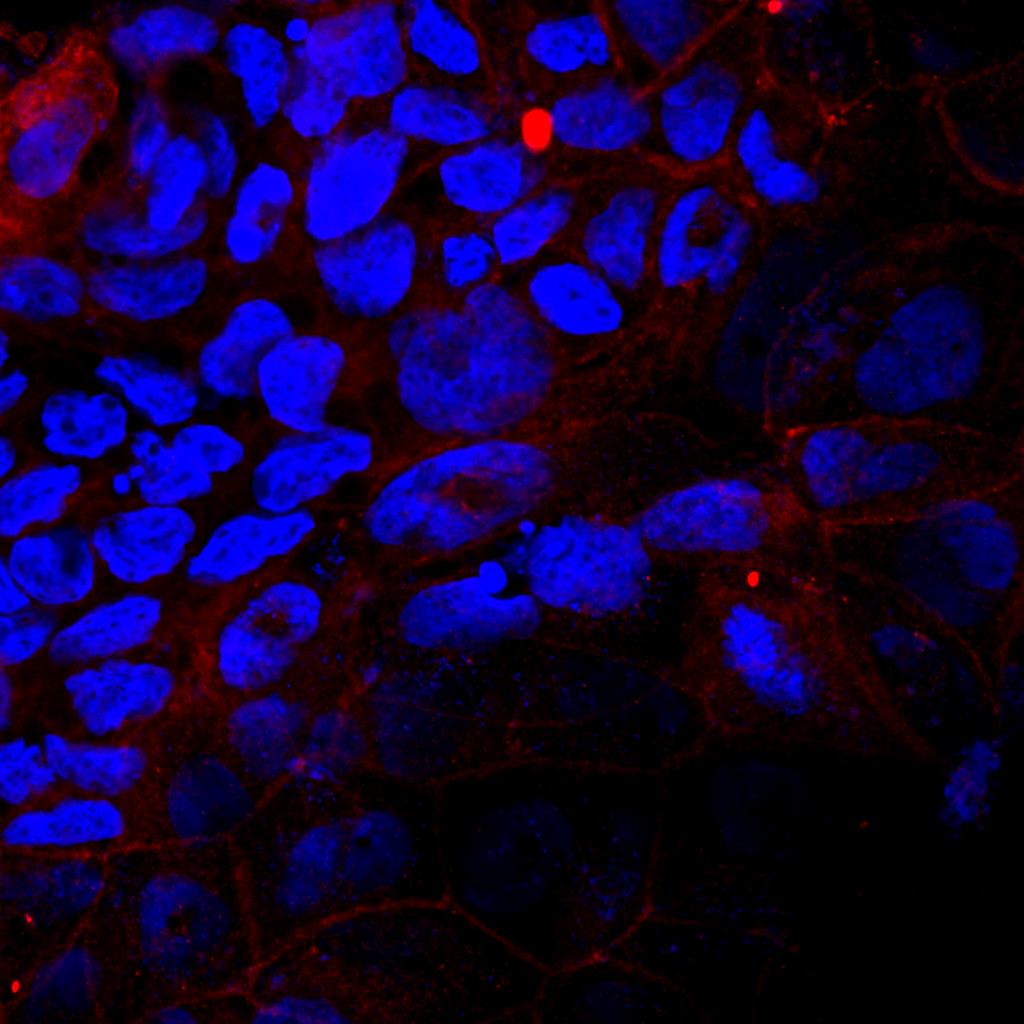

Supplement: Supplemental Information 10 [file peerj-12-16692-s010.zip › original data-figure 5/5C/miR-138 inhi+si-NLRP3+OE-HMGB1/2.Occludin.tif]

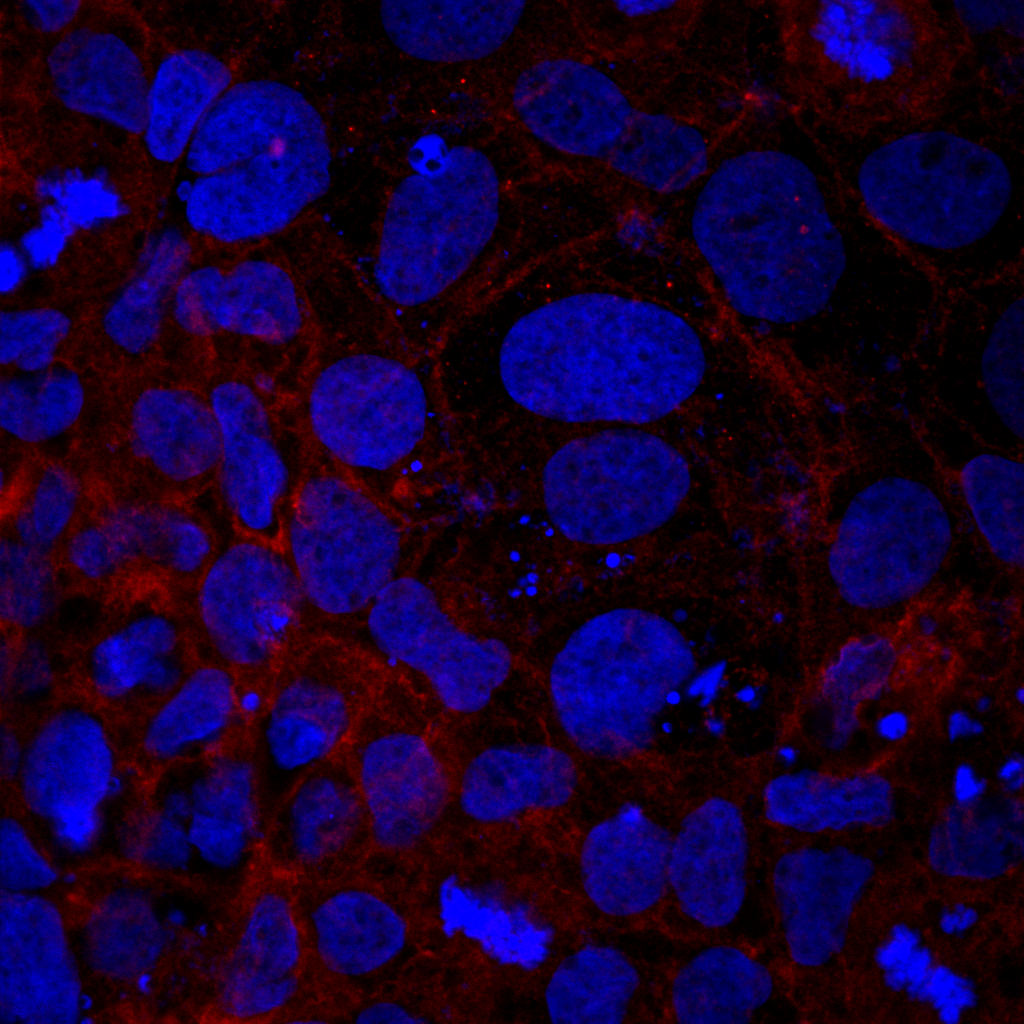

Supplement: Supplemental Information 10 [file peerj-12-16692-s010.zip › original data-figure 5/5C/miR-138 inhi+si-NLRP3+OE-HMGB1/3.Claudin-1.tif]

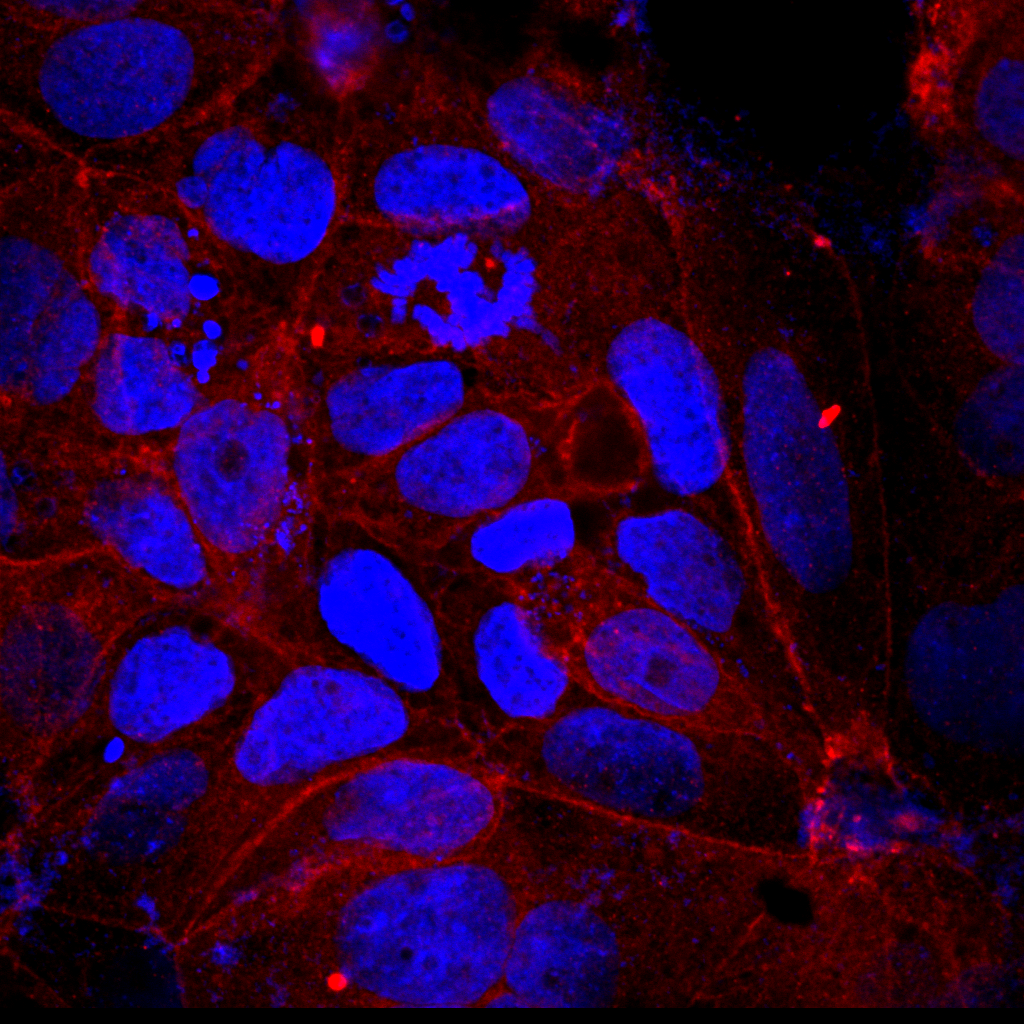

Supplement: Supplemental Information 10 [file peerj-12-16692-s010.zip › original data-figure 5/5C/miR-138 inhi+si-NLRP3+OE-NC/1.ZO-1.tif]

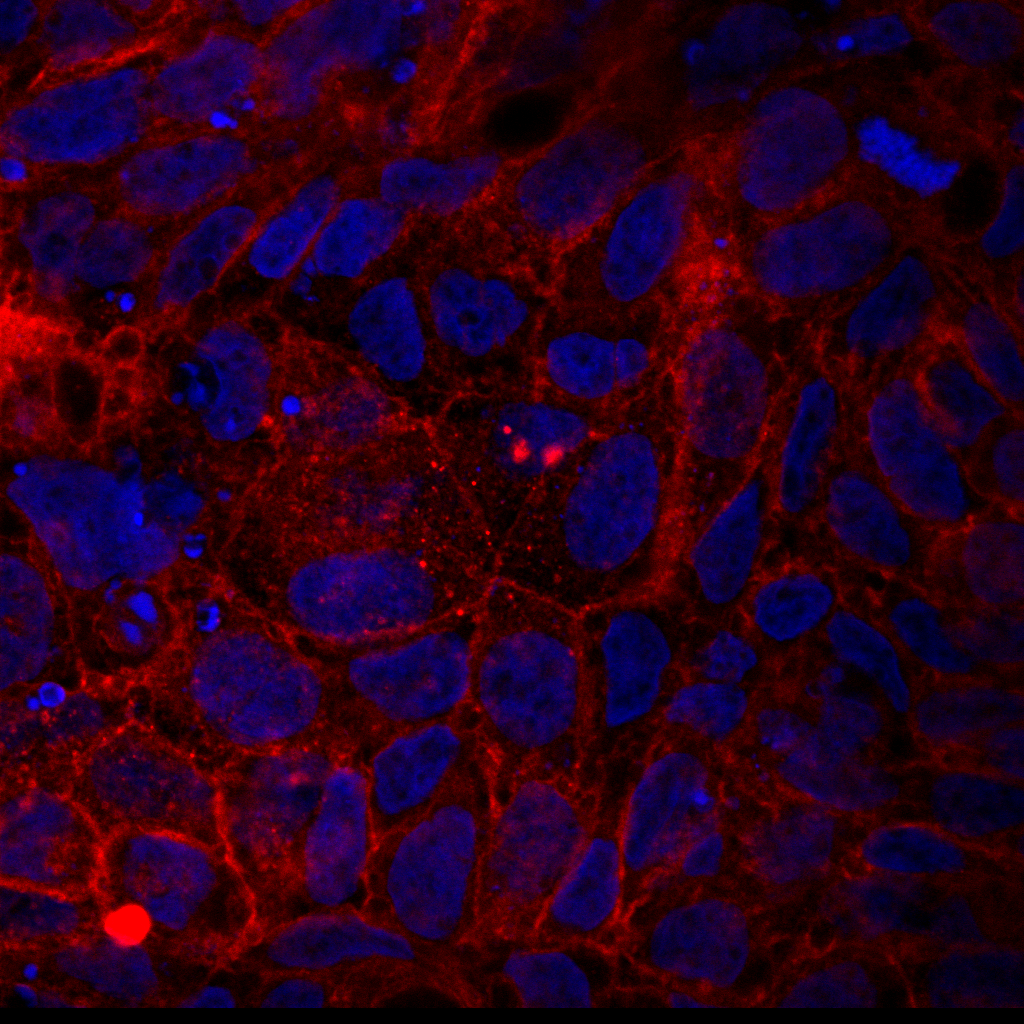

Supplement: Supplemental Information 10 [file peerj-12-16692-s010.zip › original data-figure 5/5C/miR-138 inhi+si-NLRP3+OE-NC/2.Occludin.tif]

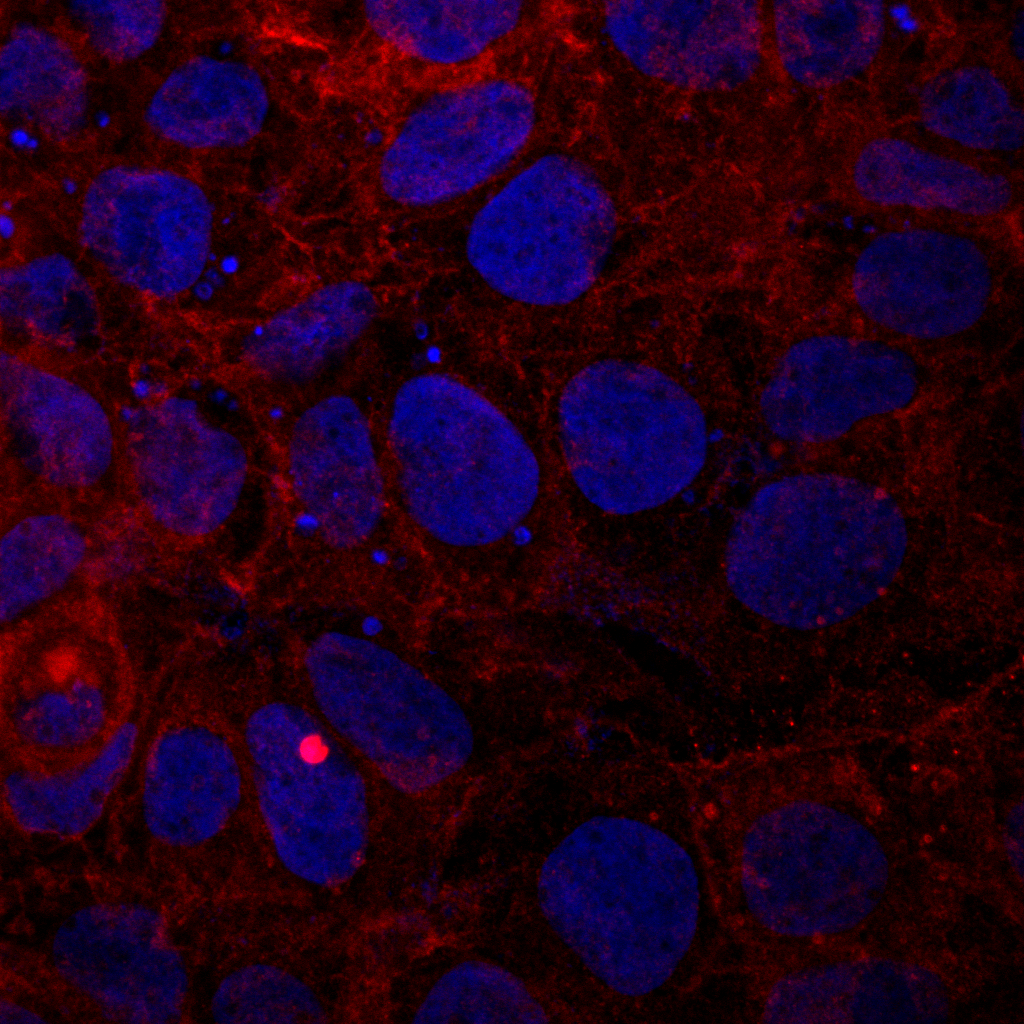

Supplement: Supplemental Information 10 [file peerj-12-16692-s010.zip › original data-figure 5/5C/miR-138 inhi+si-NLRP3+OE-NC/3.Claudin-1.tif]

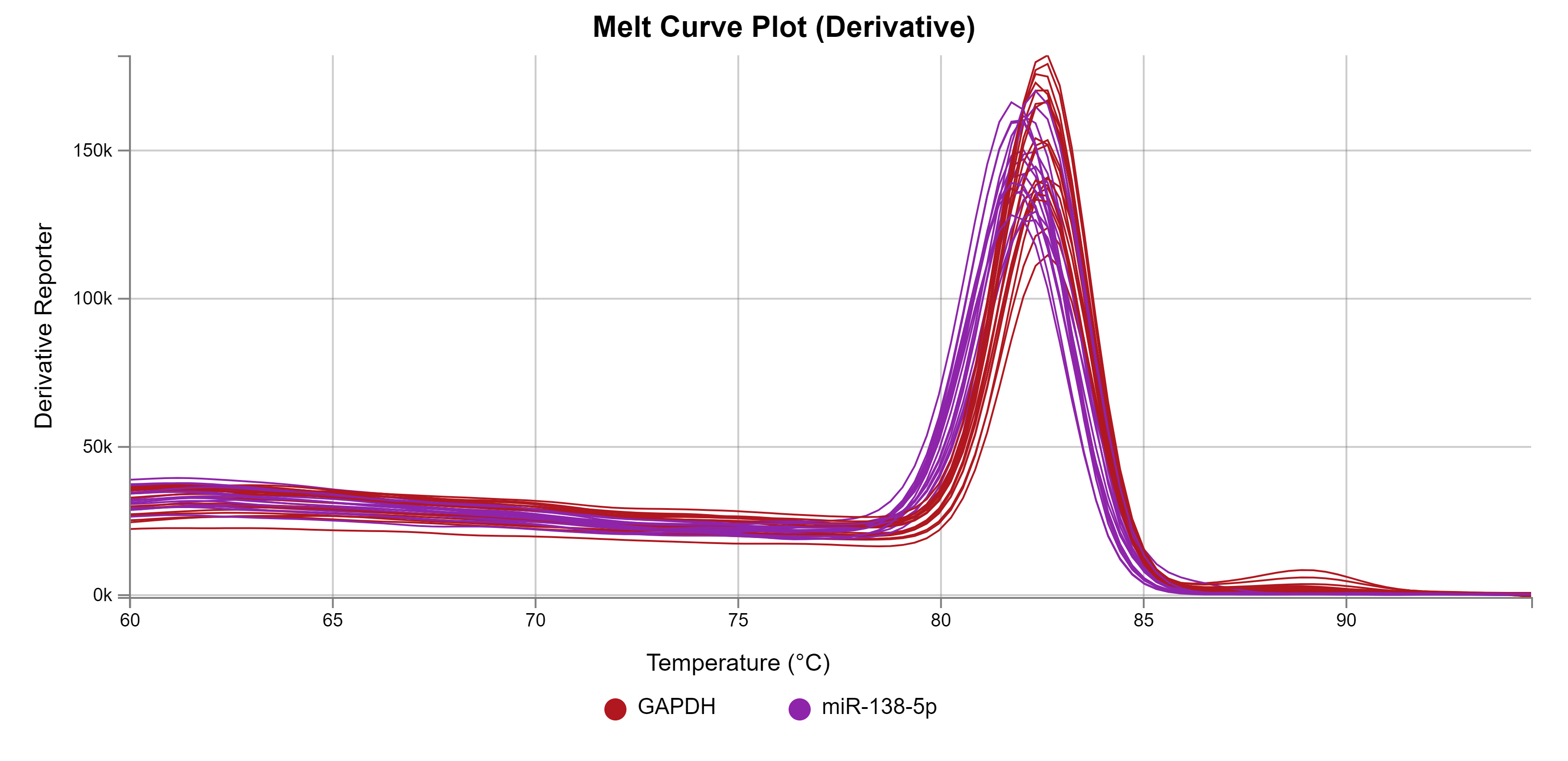

Supplement: Supplemental Information 11 [file peerj-12-16692-s011.zip › original data-figure 6-1/6A/Melt Curve Plot_2023-08-18-95546.png]

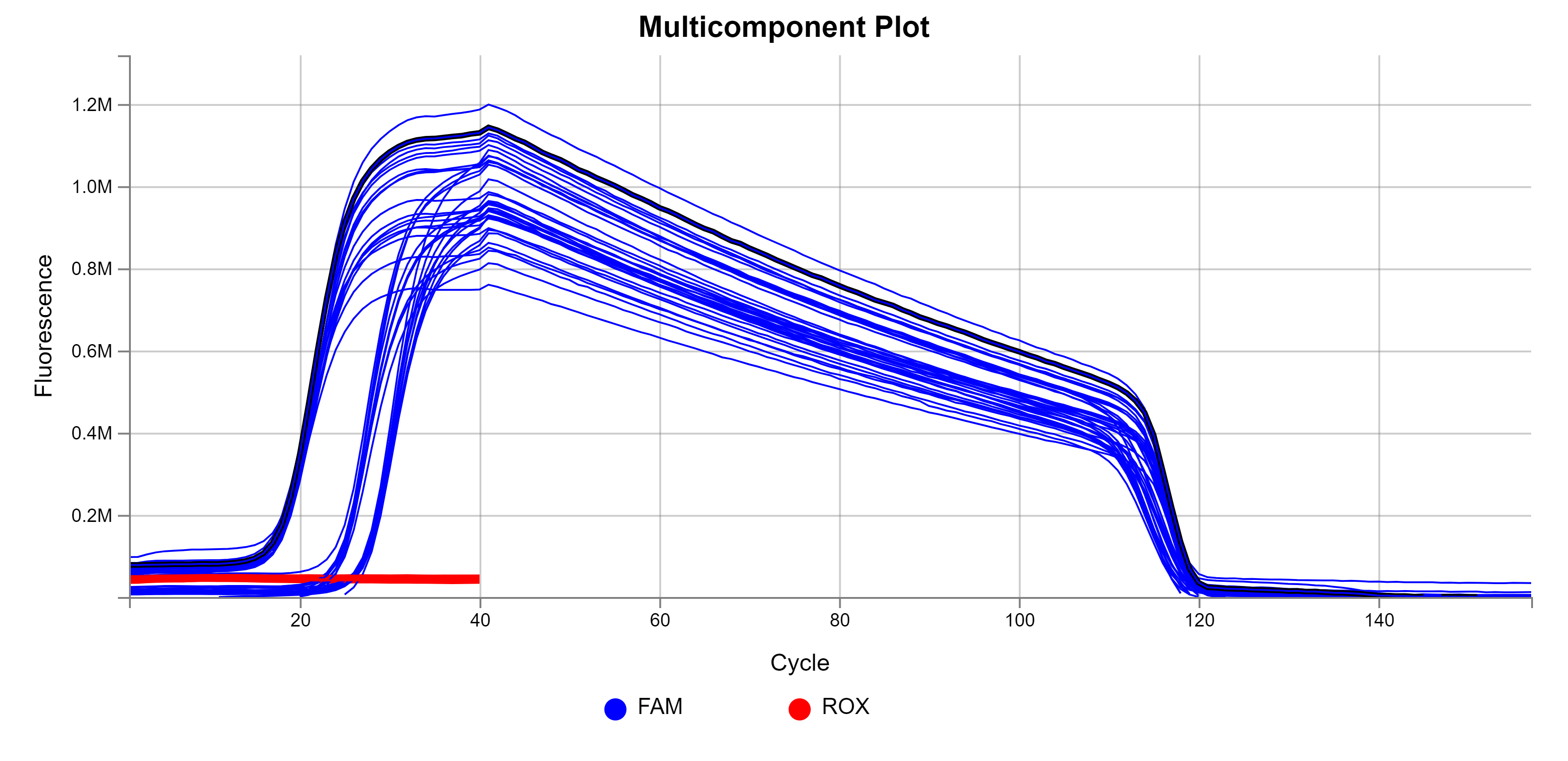

Supplement: Supplemental Information 11 [file peerj-12-16692-s011.zip › original data-figure 6-1/6A/Multicomponent Plot_2023-08-18-9564.png]

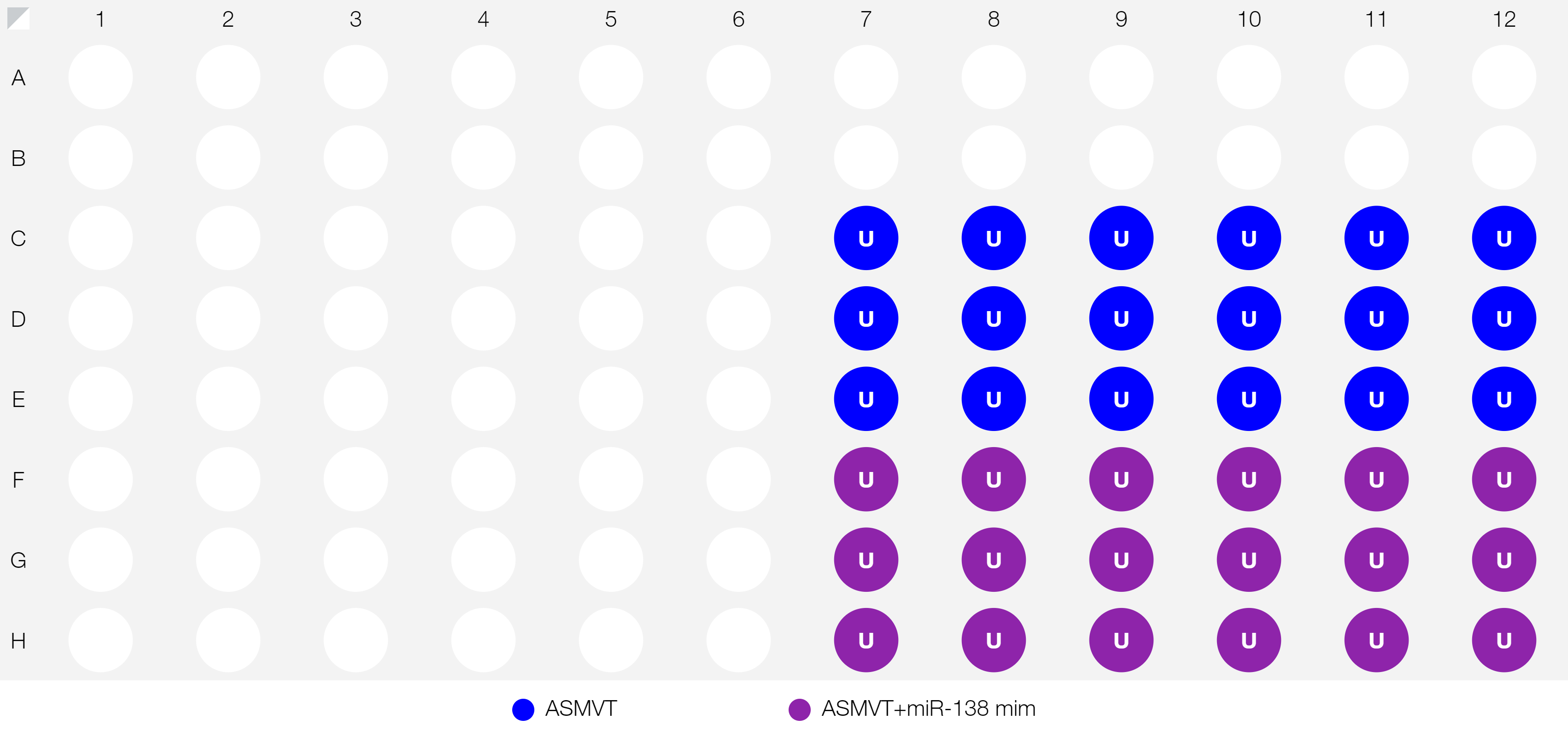

Supplement: Supplemental Information 11 [file peerj-12-16692-s011.zip › original data-figure 6-1/6A/Plate_2023-08-18-95657.png]

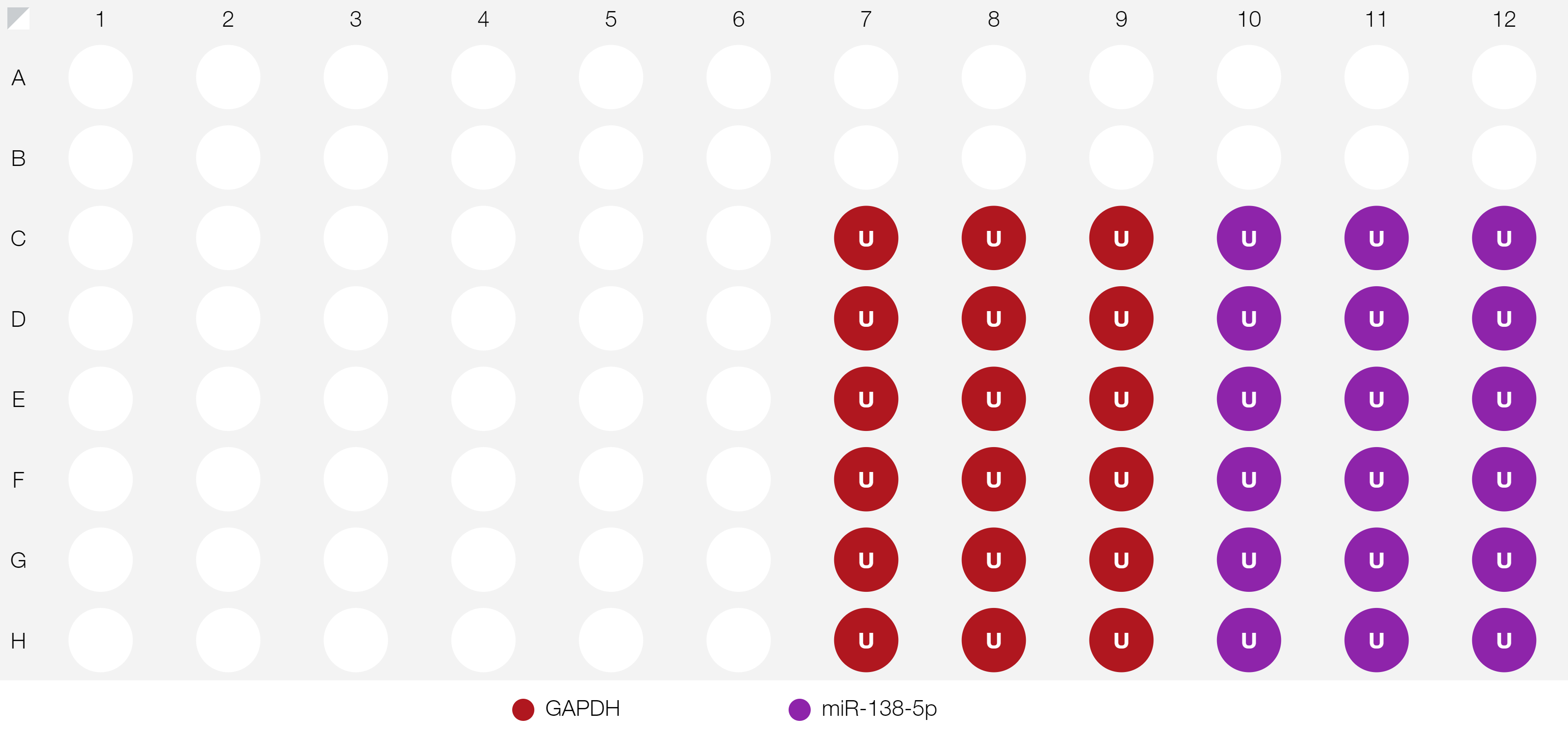

Supplement: Supplemental Information 11 [file peerj-12-16692-s011.zip › original data-figure 6-1/6A/Plate_2023-08-18-95714.png]

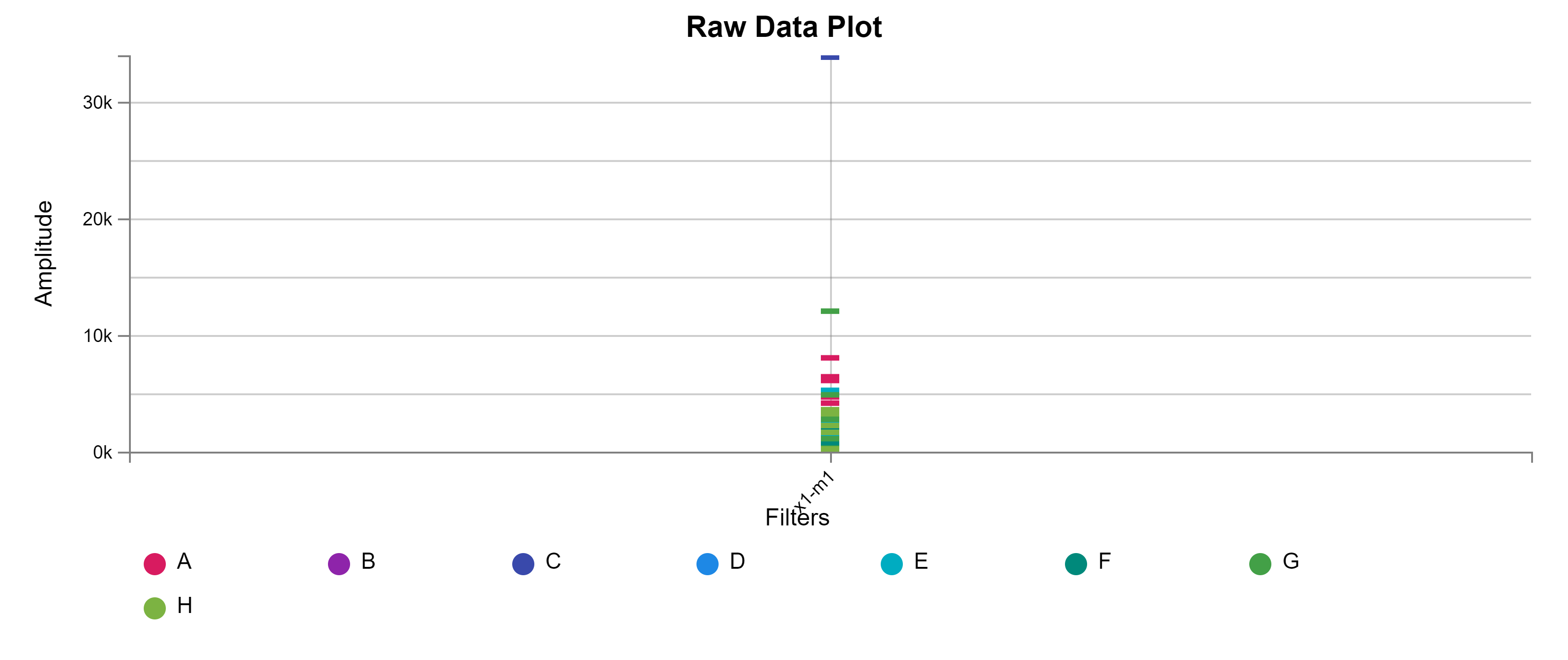

Supplement: Supplemental Information 11 [file peerj-12-16692-s011.zip › original data-figure 6-1/6A/Raw Data Plot_2023-08-18-95641.png]

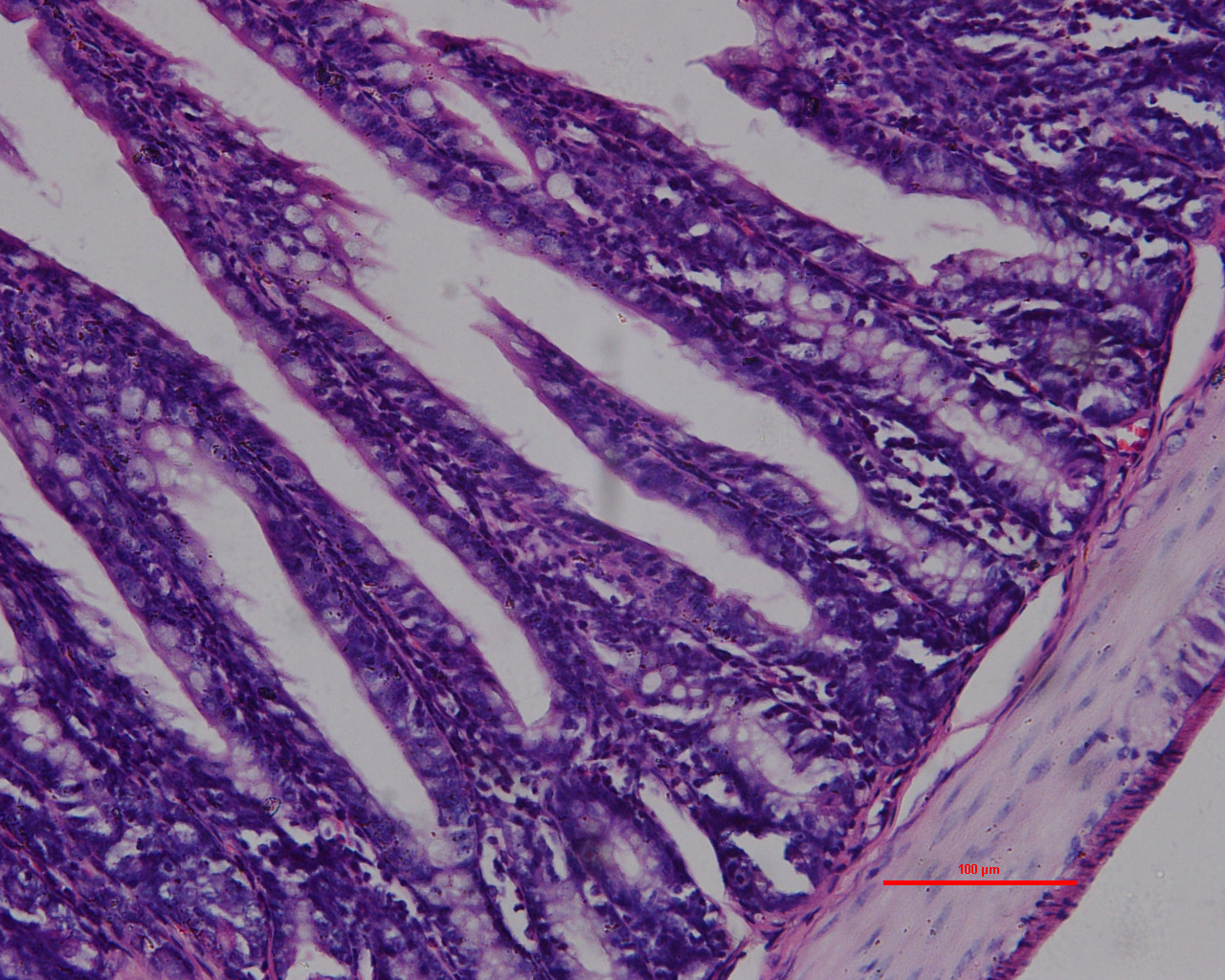

Supplement: Supplemental Information 11 [file peerj-12-16692-s011.zip › original data-figure 6-1/6B/3.ASWVT+miR-138 mim.tif]

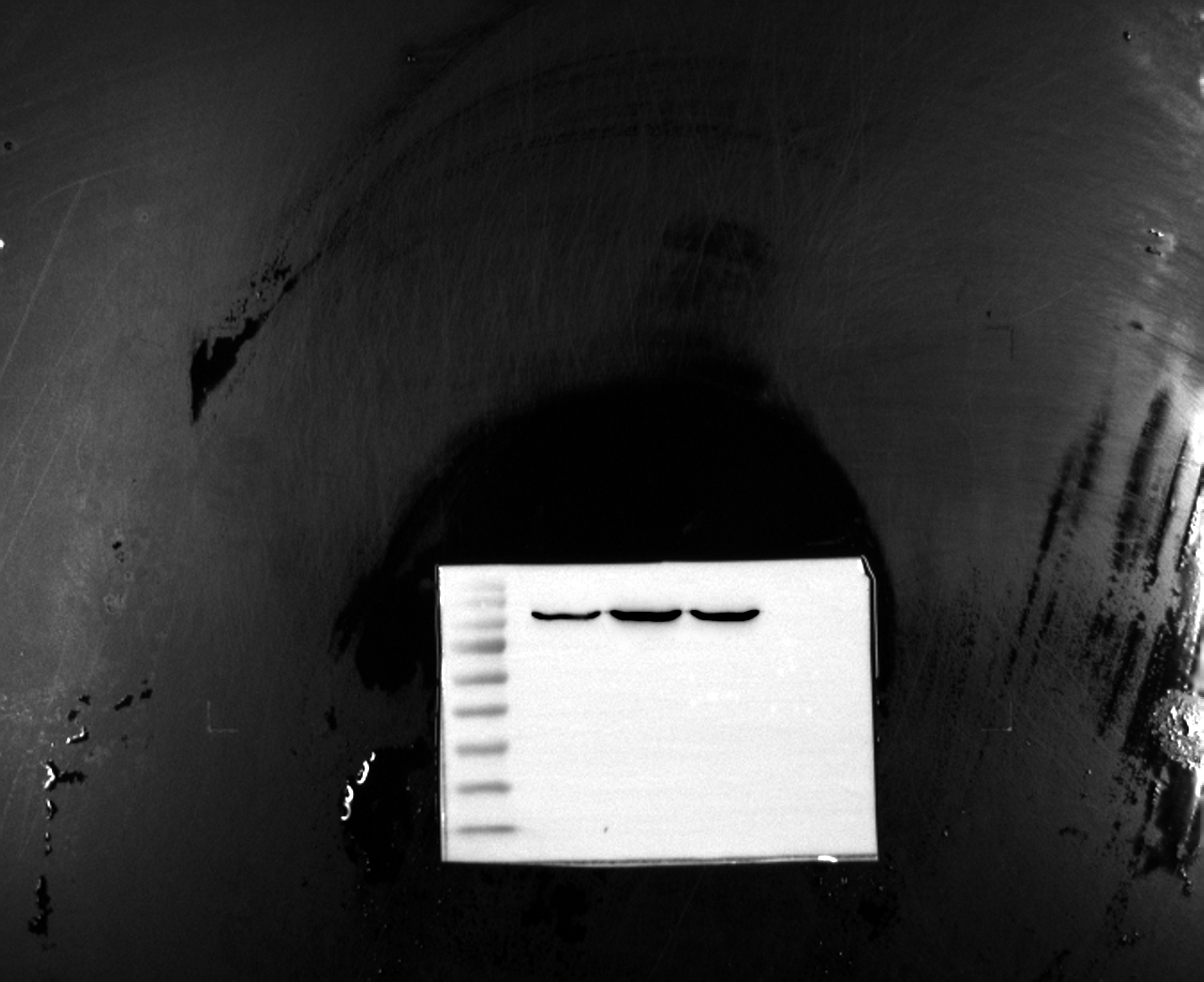

Supplement: Supplemental Information 11 [file peerj-12-16692-s011.zip › original data-figure 6-1/6C/1.NLRP3.tif]

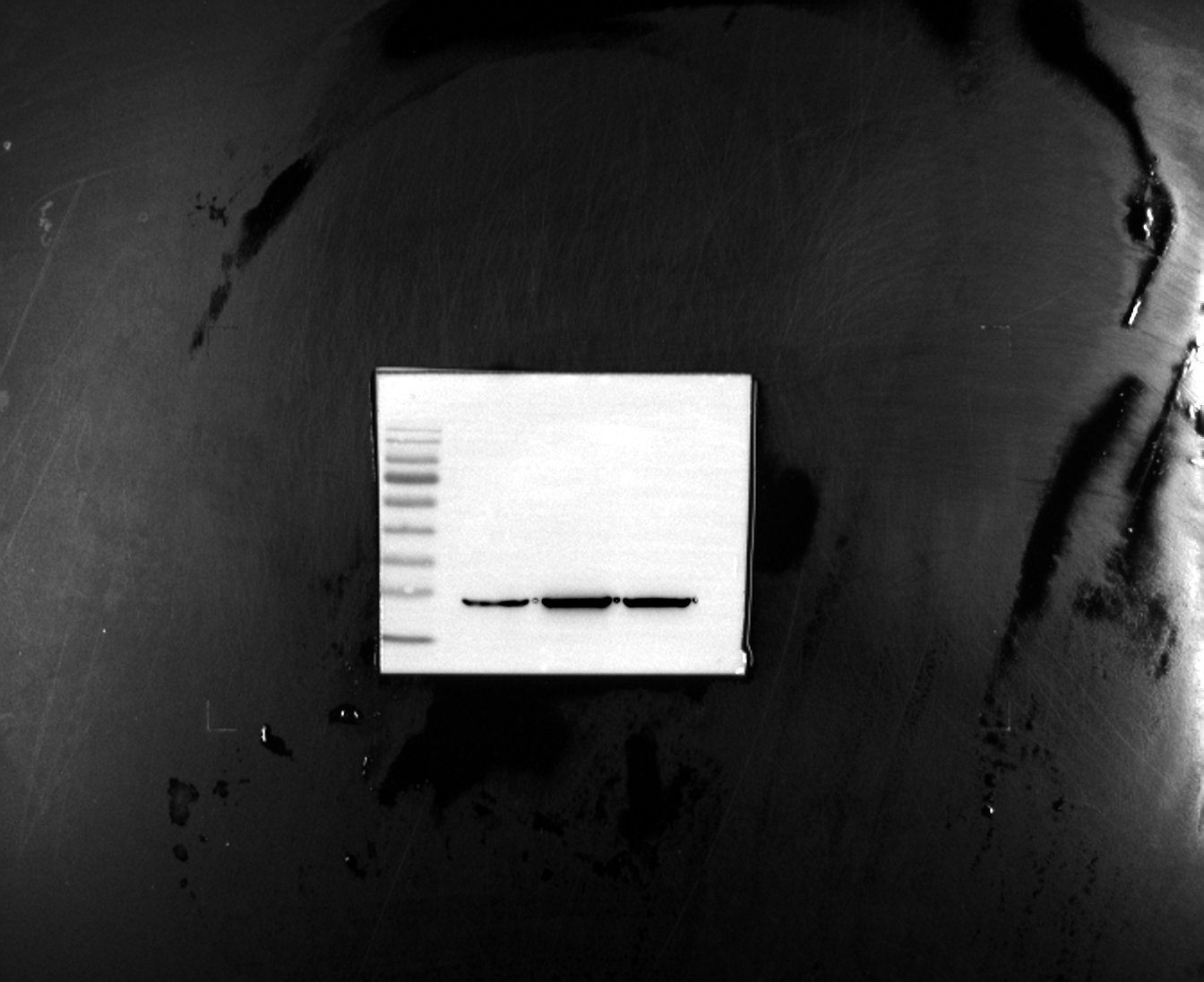

Supplement: Supplemental Information 11 [file peerj-12-16692-s011.zip › original data-figure 6-1/6C/2.ASC.tif]

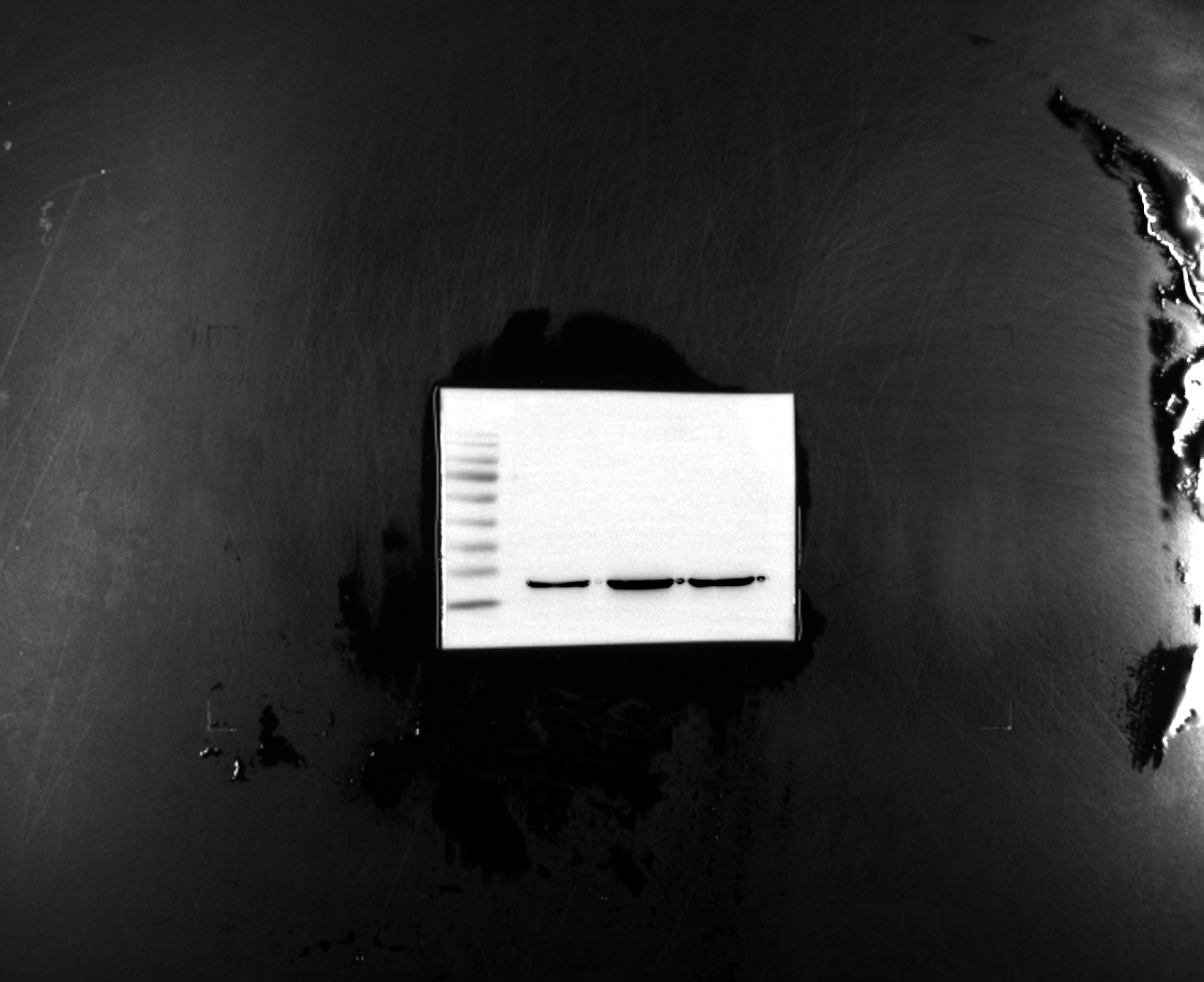

Supplement: Supplemental Information 11 [file peerj-12-16692-s011.zip › original data-figure 6-1/6C/3.Caspase 1.tif]

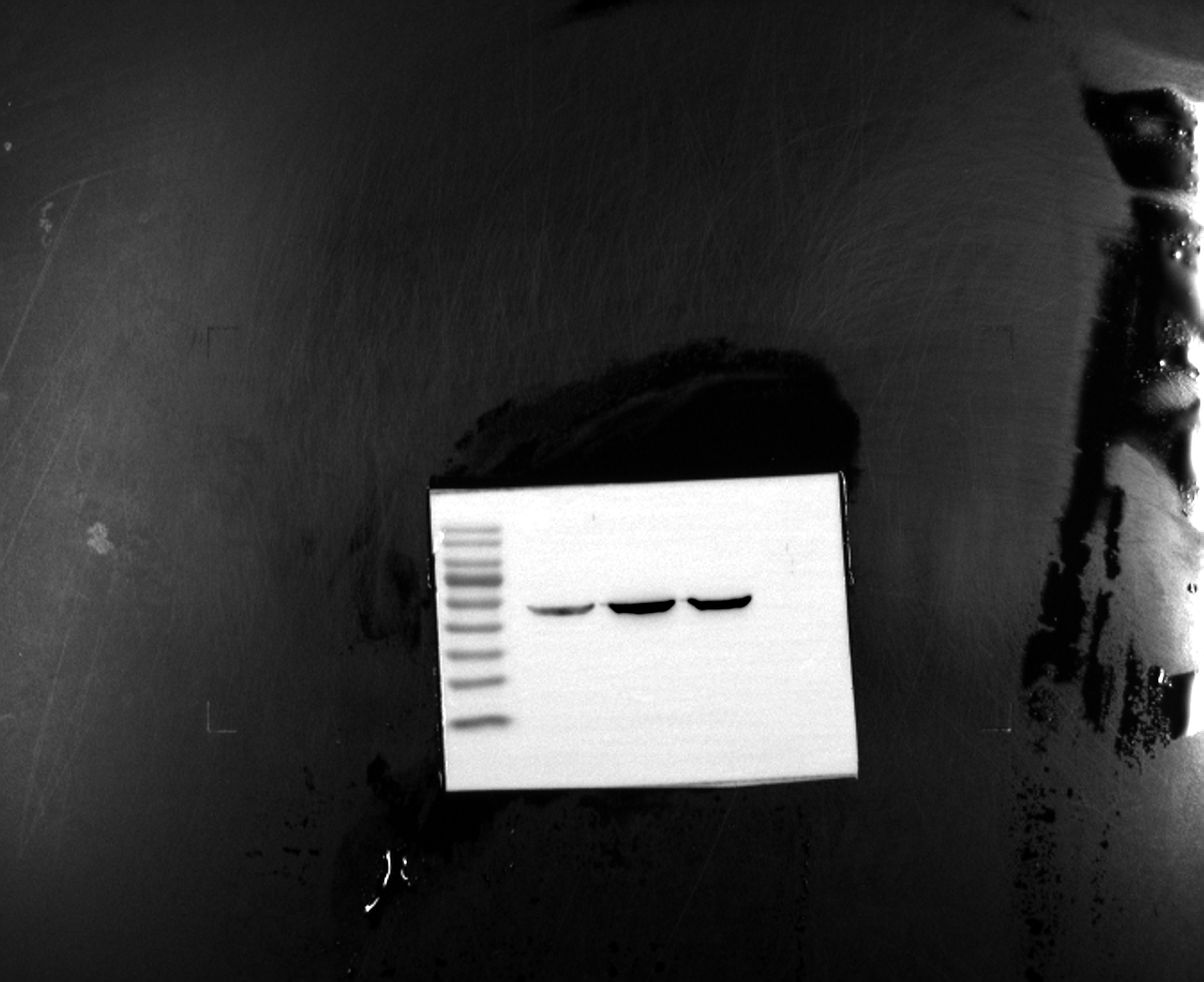

Supplement: Supplemental Information 11 [file peerj-12-16692-s011.zip › original data-figure 6-1/6C/4.GSDMD-N.tif]

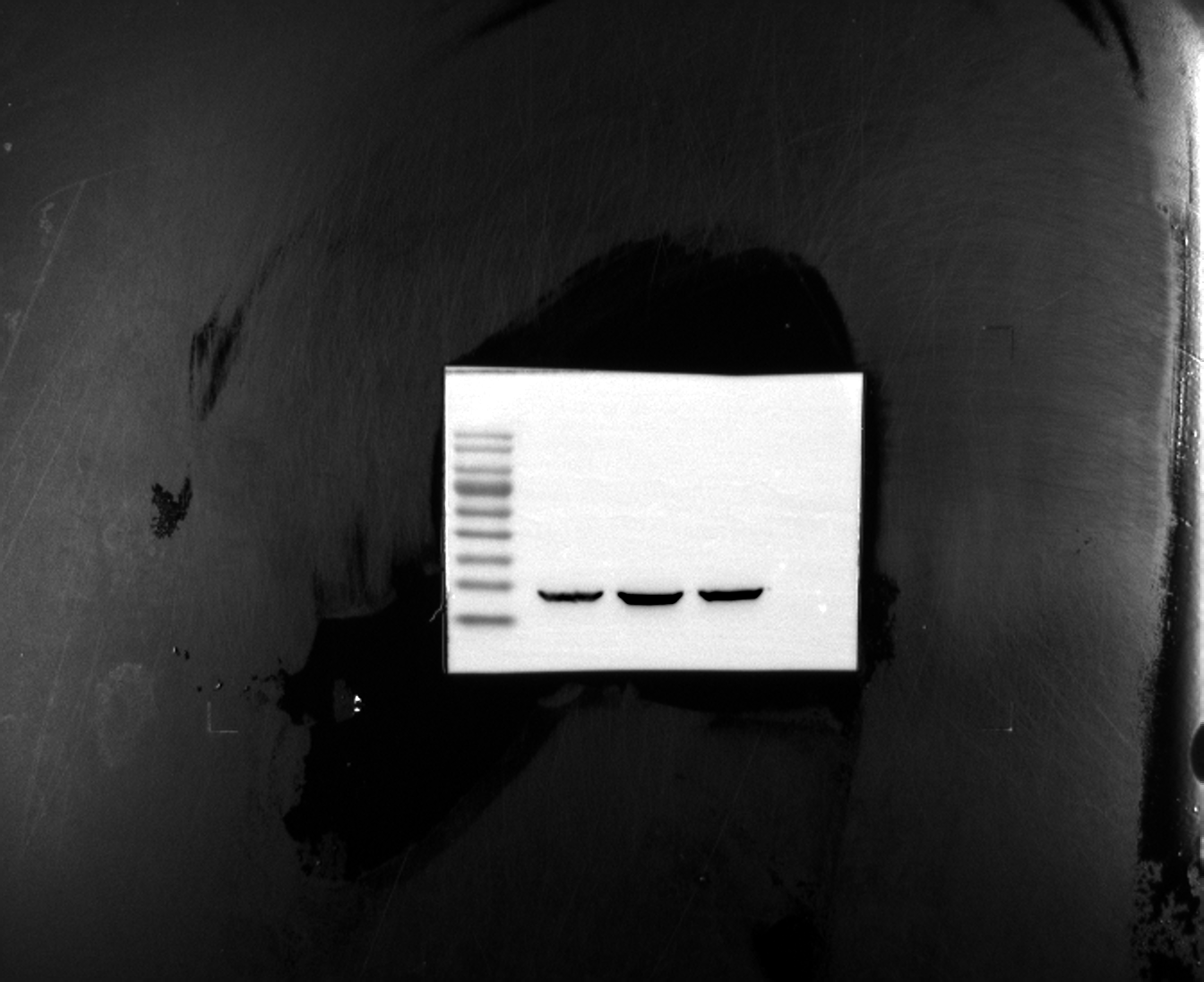

Supplement: Supplemental Information 11 [file peerj-12-16692-s011.zip › original data-figure 6-1/6C/5.IL-18.tif]

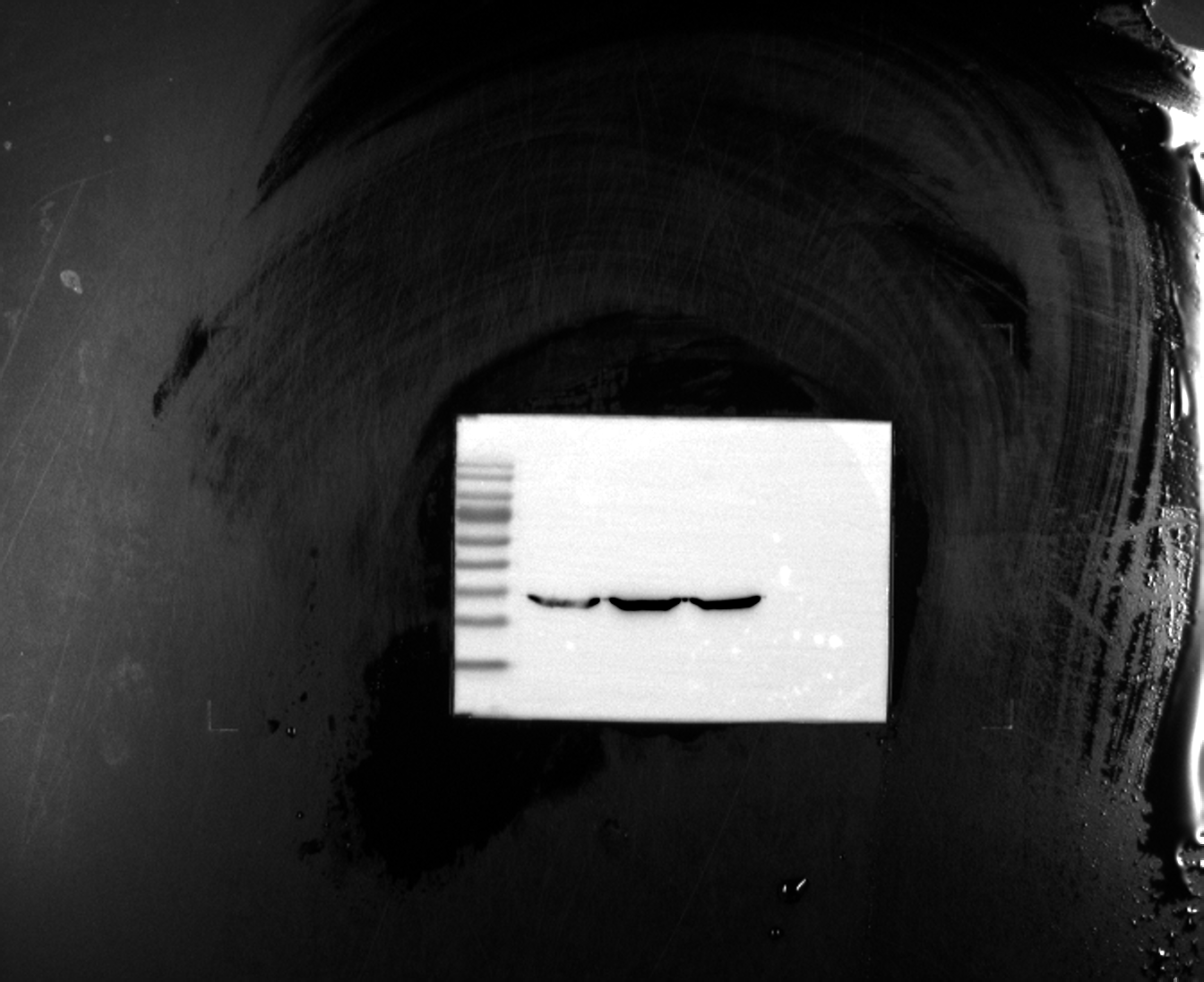

Supplement: Supplemental Information 11 [file peerj-12-16692-s011.zip › original data-figure 6-1/6C/6.IL-1β.tif]

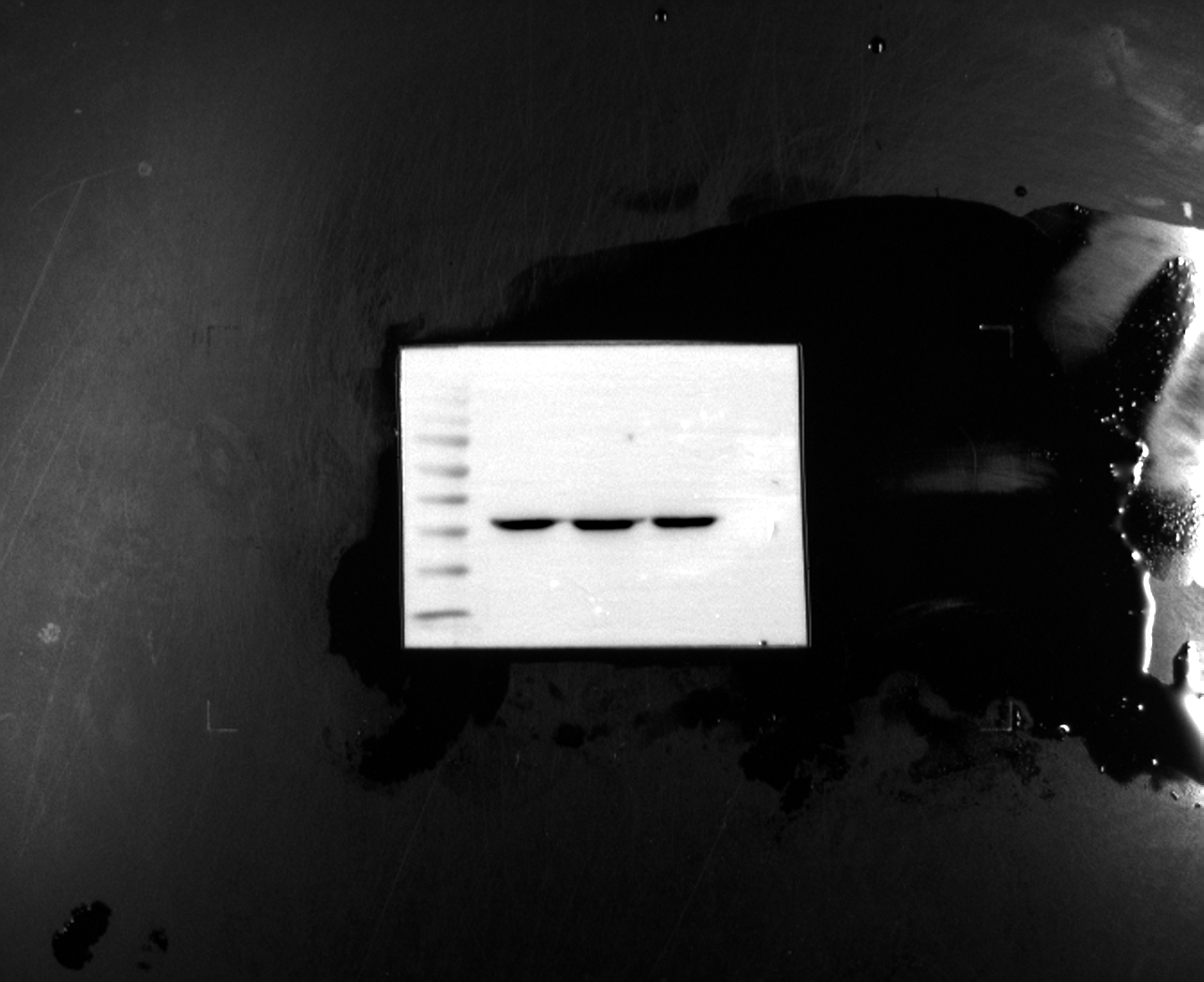

Supplement: Supplemental Information 11 [file peerj-12-16692-s011.zip › original data-figure 6-1/6C/7.GAPDH.tif]

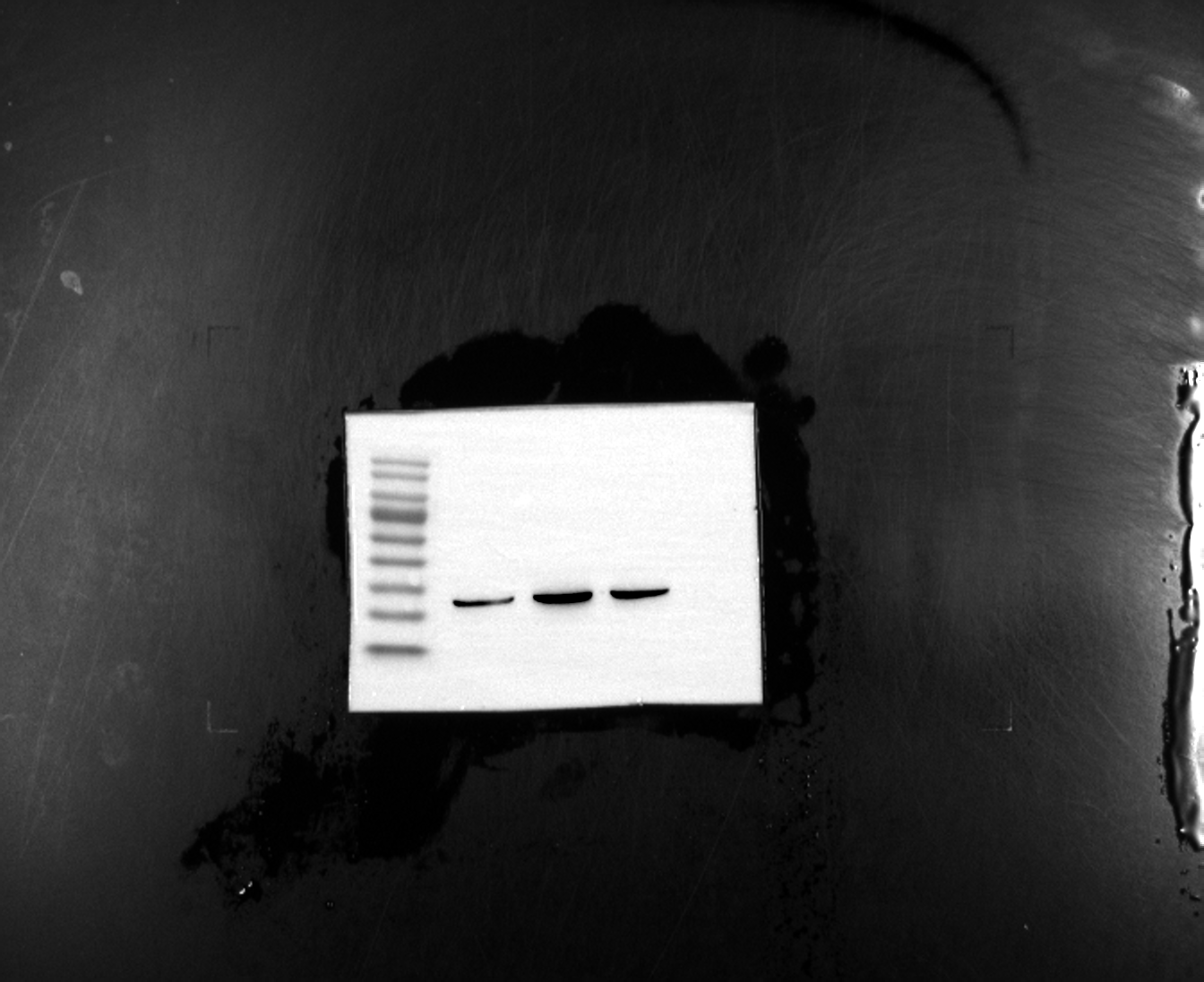

Supplement: Supplemental Information 11 [file peerj-12-16692-s011.zip › original data-figure 6-1/6D/1.HMGB1.tif]

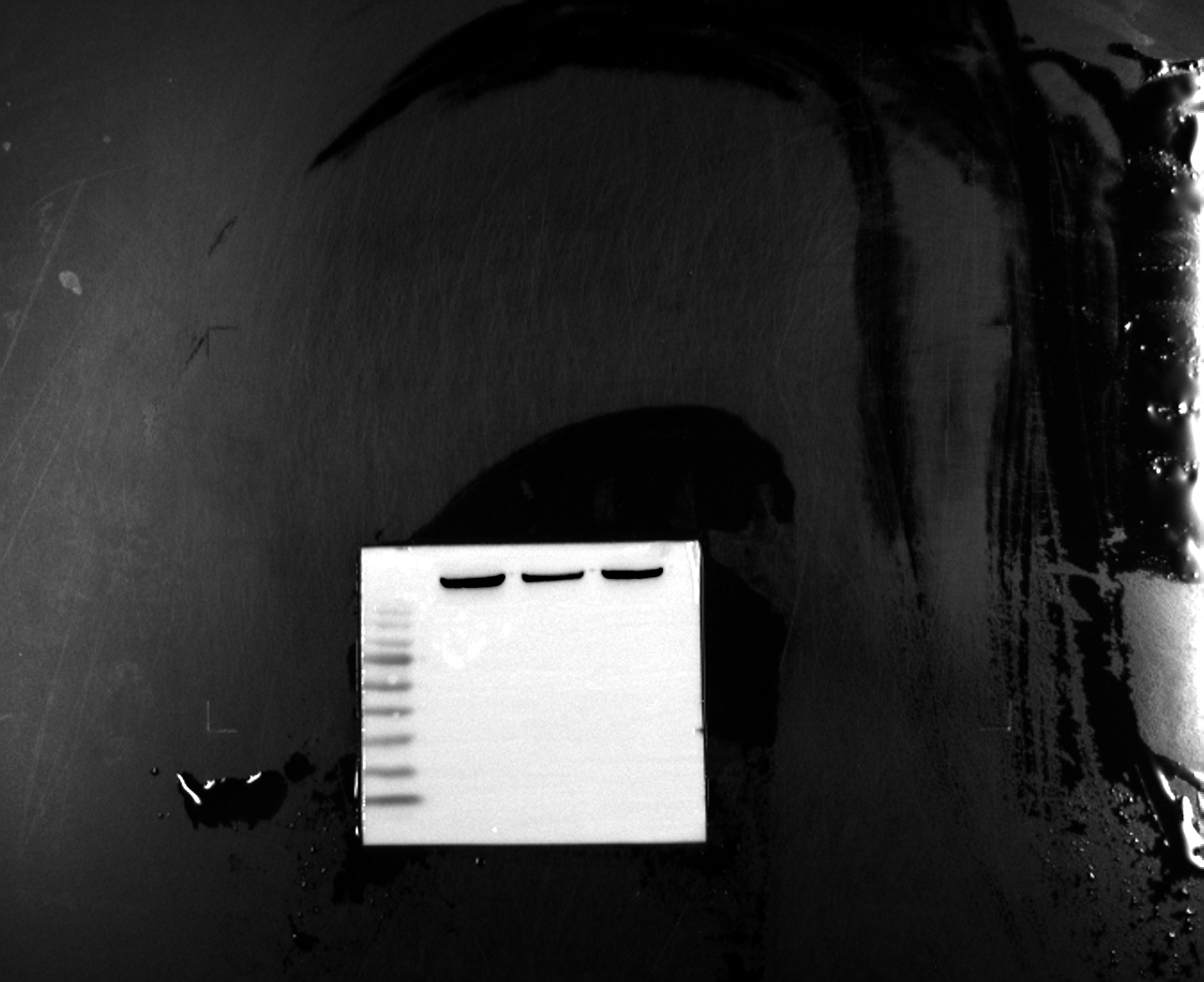

Supplement: Supplemental Information 11 [file peerj-12-16692-s011.zip › original data-figure 6-1/6D/2.ZO-1.tif]

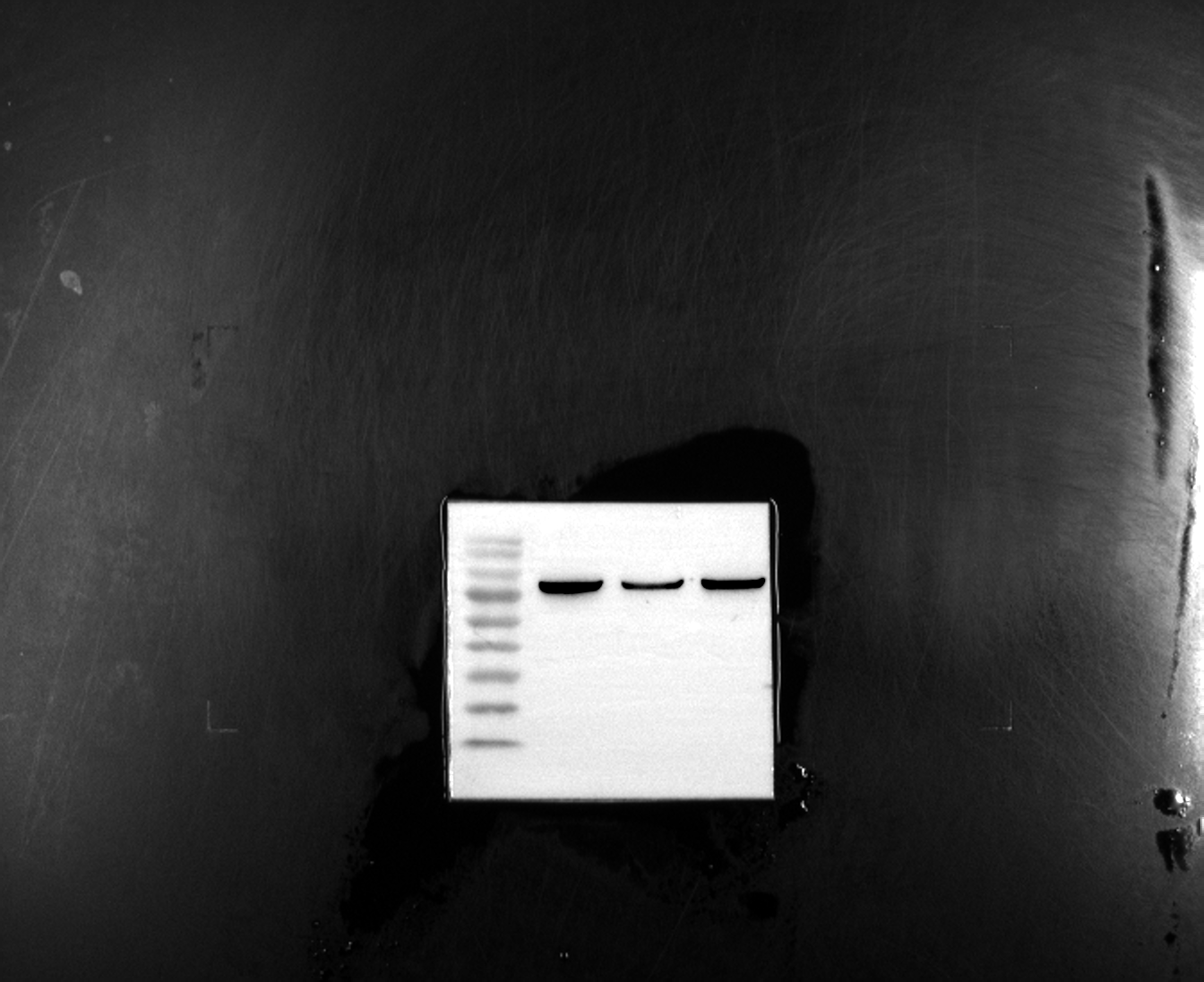

Supplement: Supplemental Information 11 [file peerj-12-16692-s011.zip › original data-figure 6-1/6D/3.Occludin.tif]

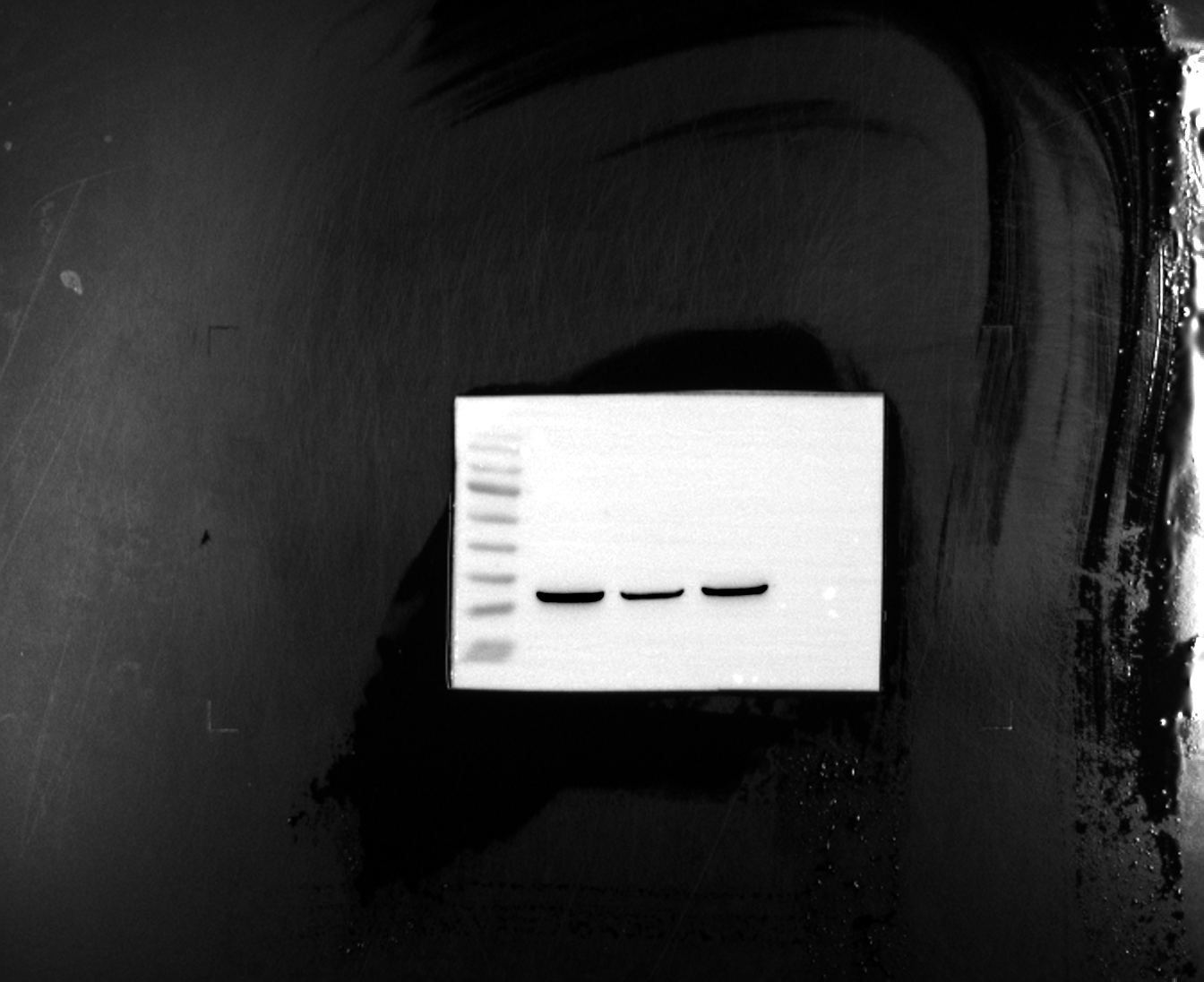

Supplement: Supplemental Information 11 [file peerj-12-16692-s011.zip › original data-figure 6-1/6D/4.Claudin-1.tif]

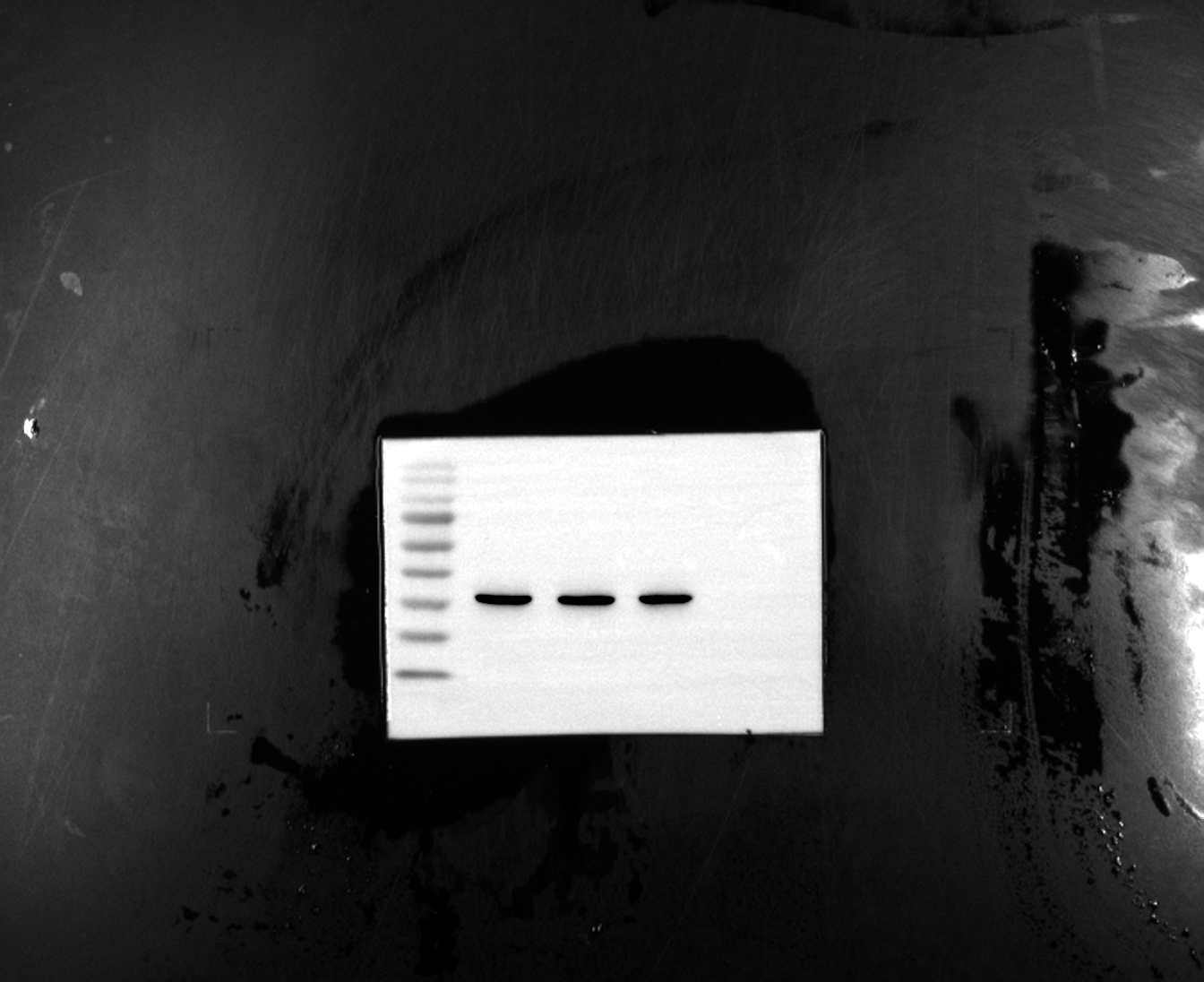

Supplement: Supplemental Information 11 [file peerj-12-16692-s011.zip › original data-figure 6-1/6D/5.GAPDH.tif]

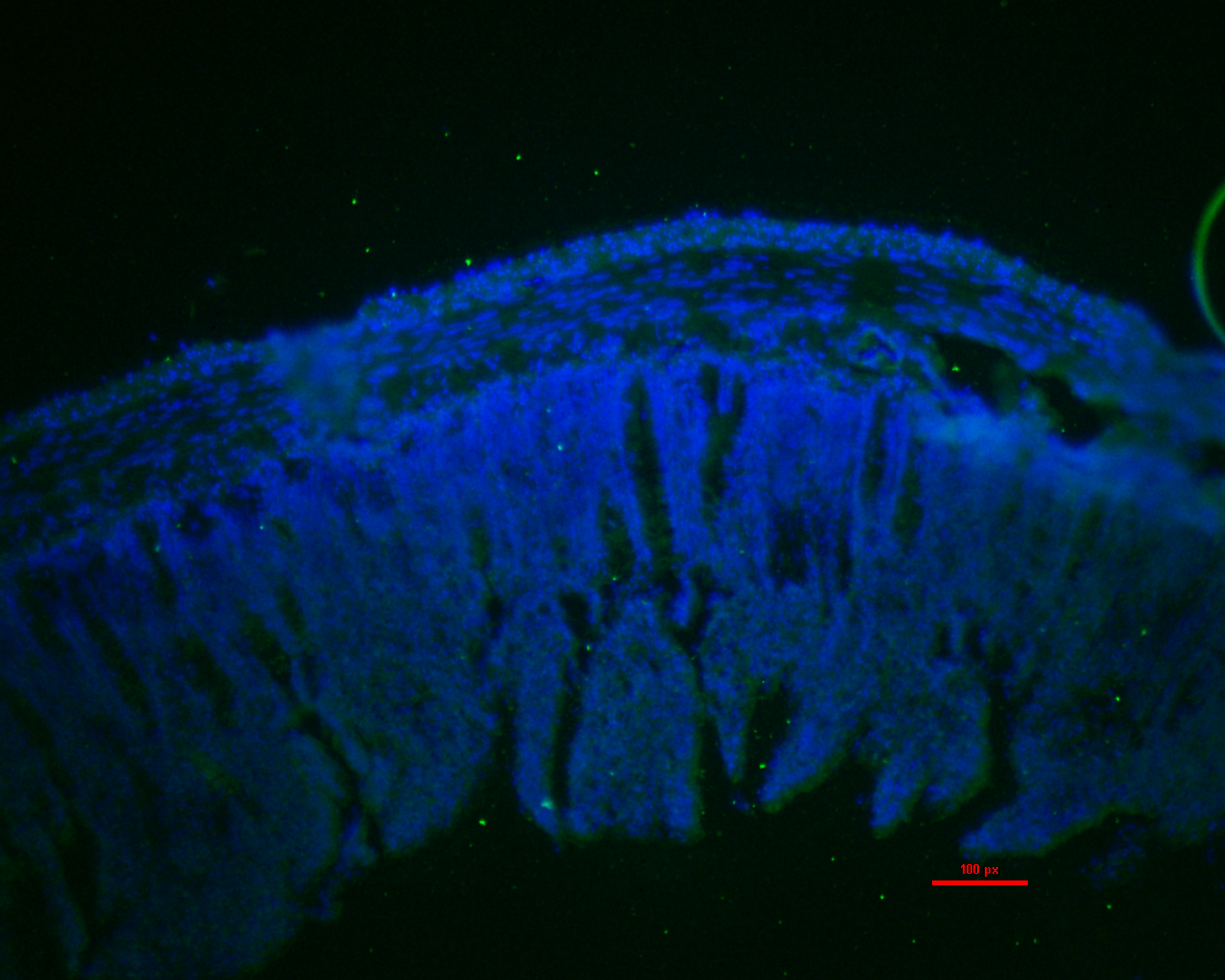

Supplement: Supplemental Information 12 [file peerj-12-16692-s012.zip › original data-figure 6-2/6E/1.Sham/1.NLRP3.tif]

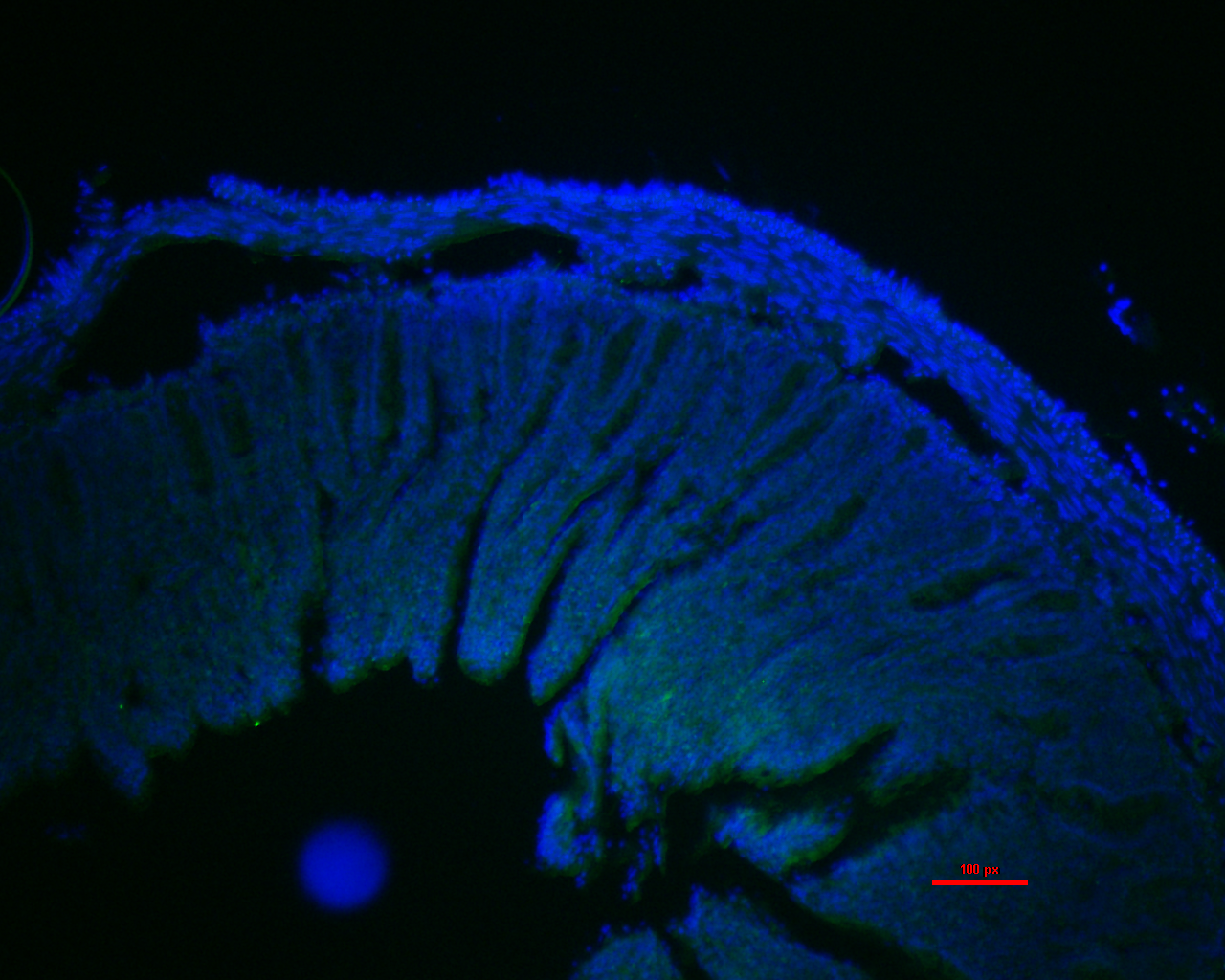

Supplement: Supplemental Information 12 [file peerj-12-16692-s012.zip › original data-figure 6-2/6E/1.Sham/2.HMGB1.tif]

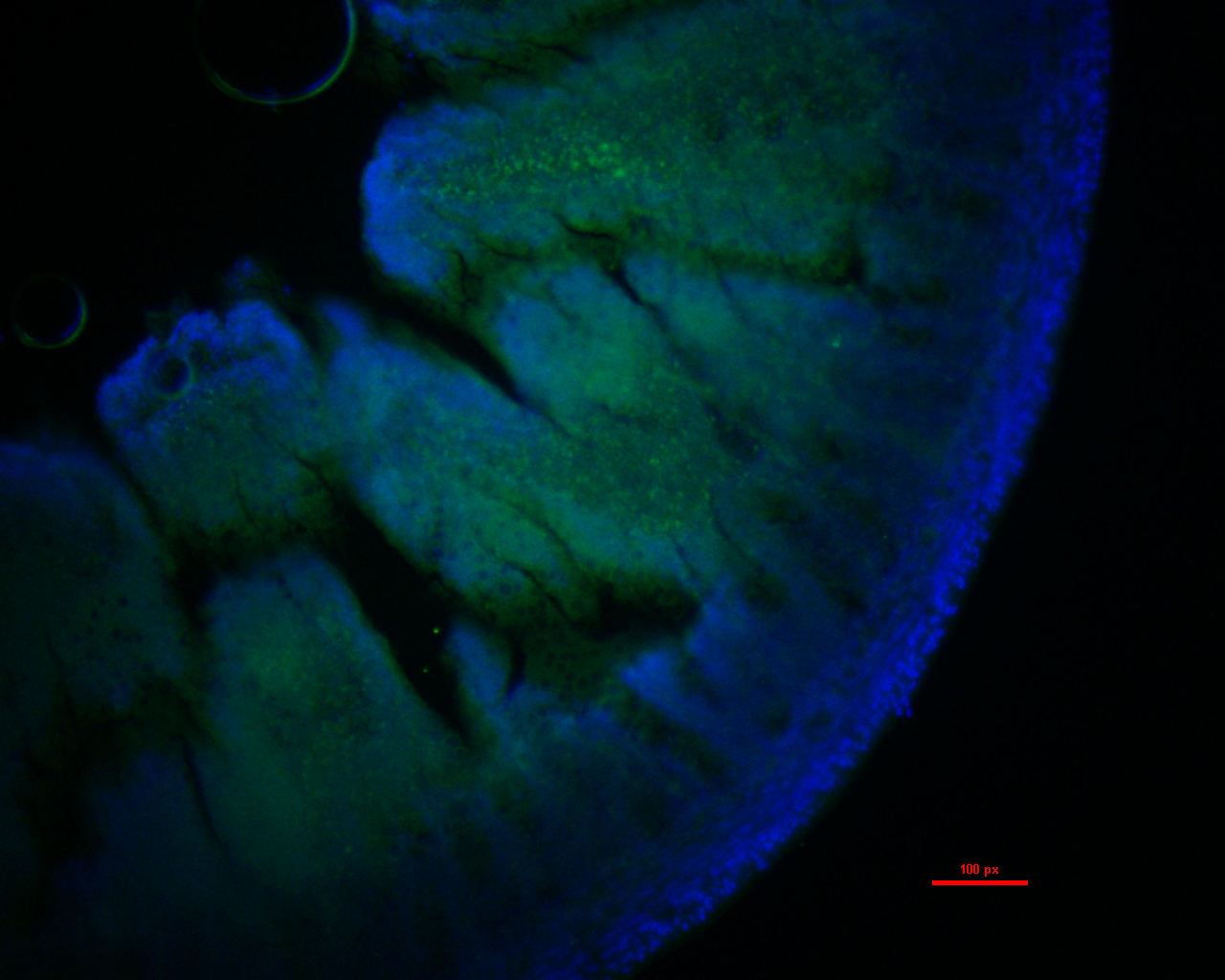

Supplement: Supplemental Information 12 [file peerj-12-16692-s012.zip › original data-figure 6-2/6E/2.ASWVY/1.NLRP3.tif]

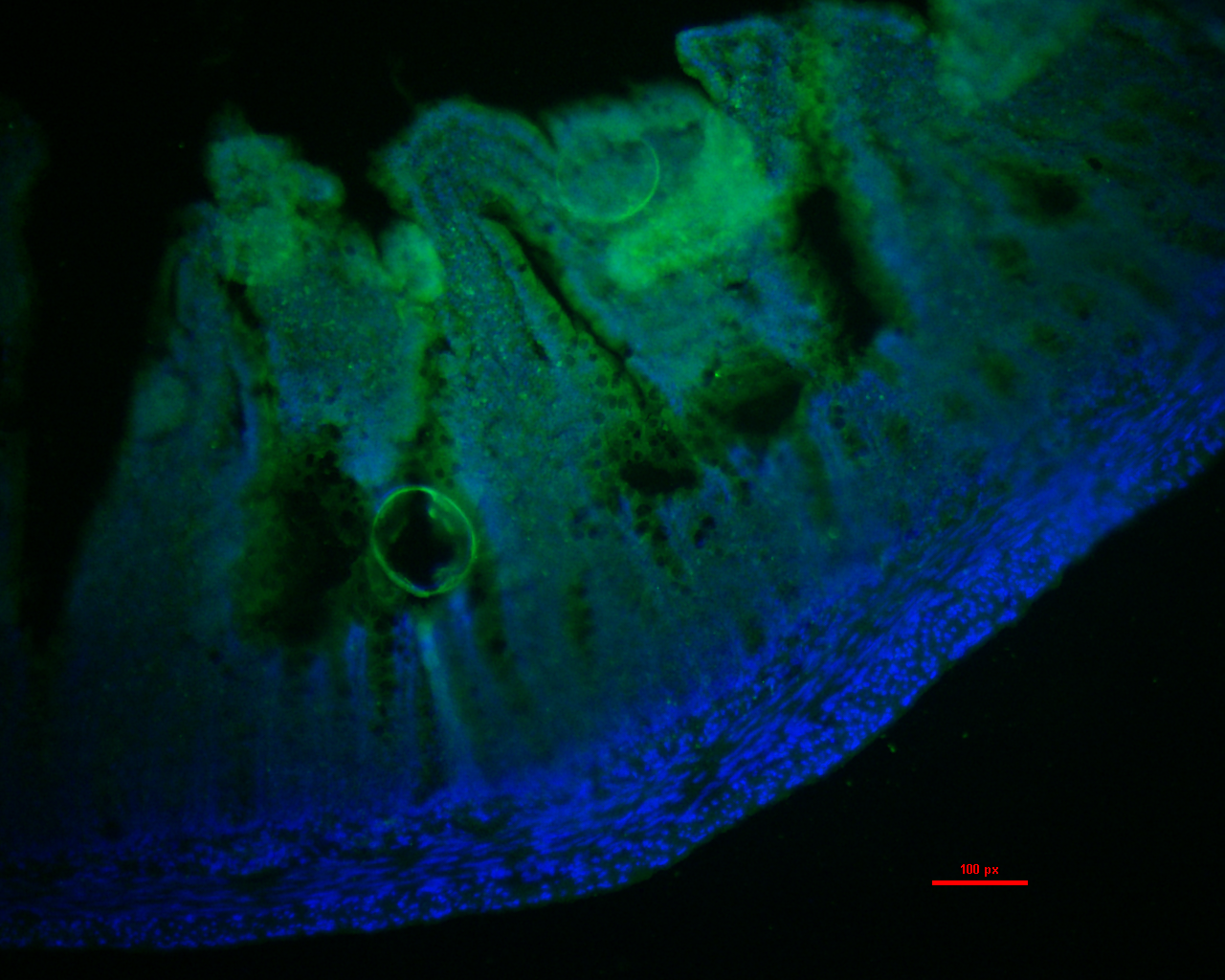

Supplement: Supplemental Information 12 [file peerj-12-16692-s012.zip › original data-figure 6-2/6E/2.ASWVY/2.HMGB1.tif]

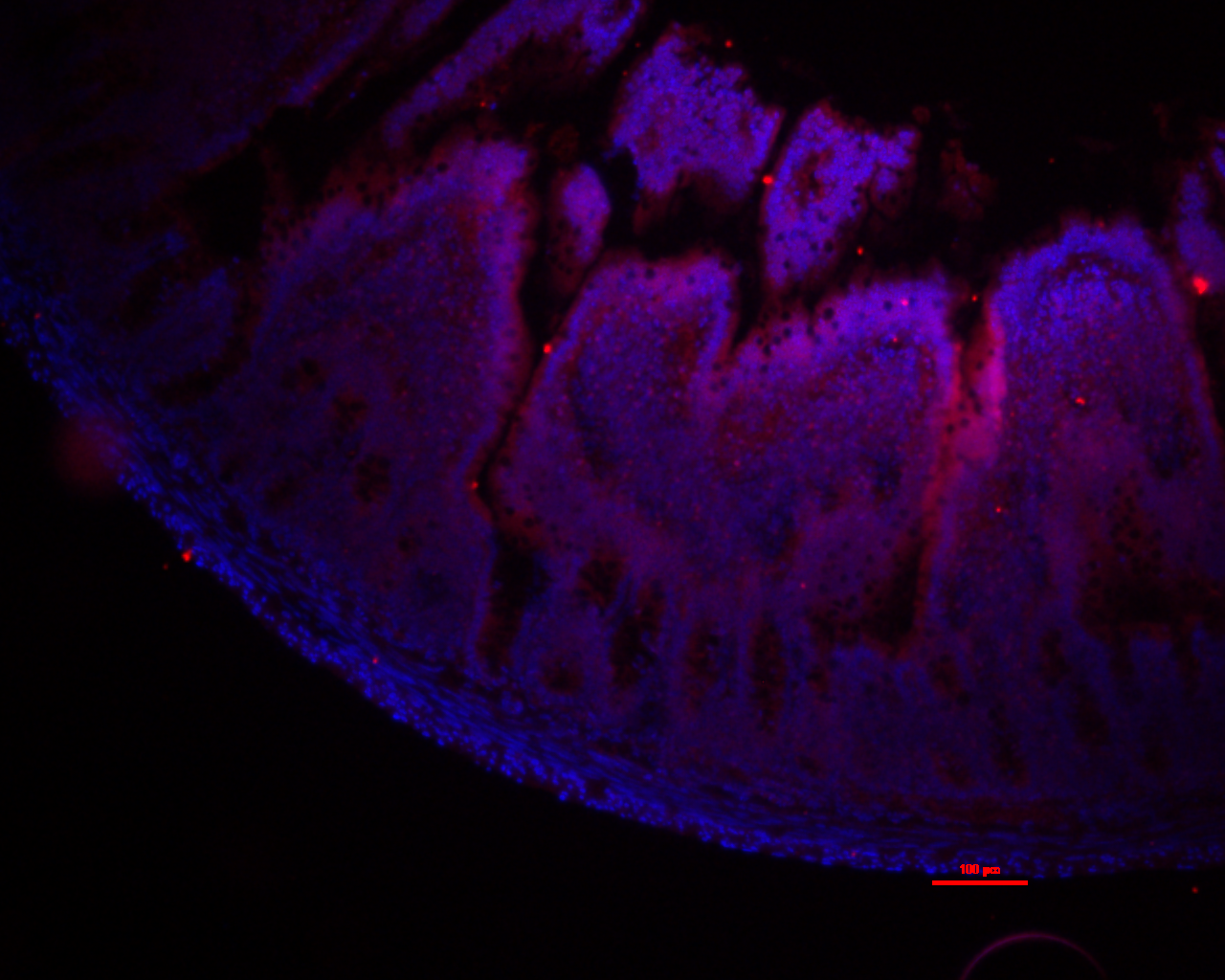

Supplement: Supplemental Information 12 [file peerj-12-16692-s012.zip › original data-figure 6-2/6E/2.ASWVY/3.ZO-1.tif]

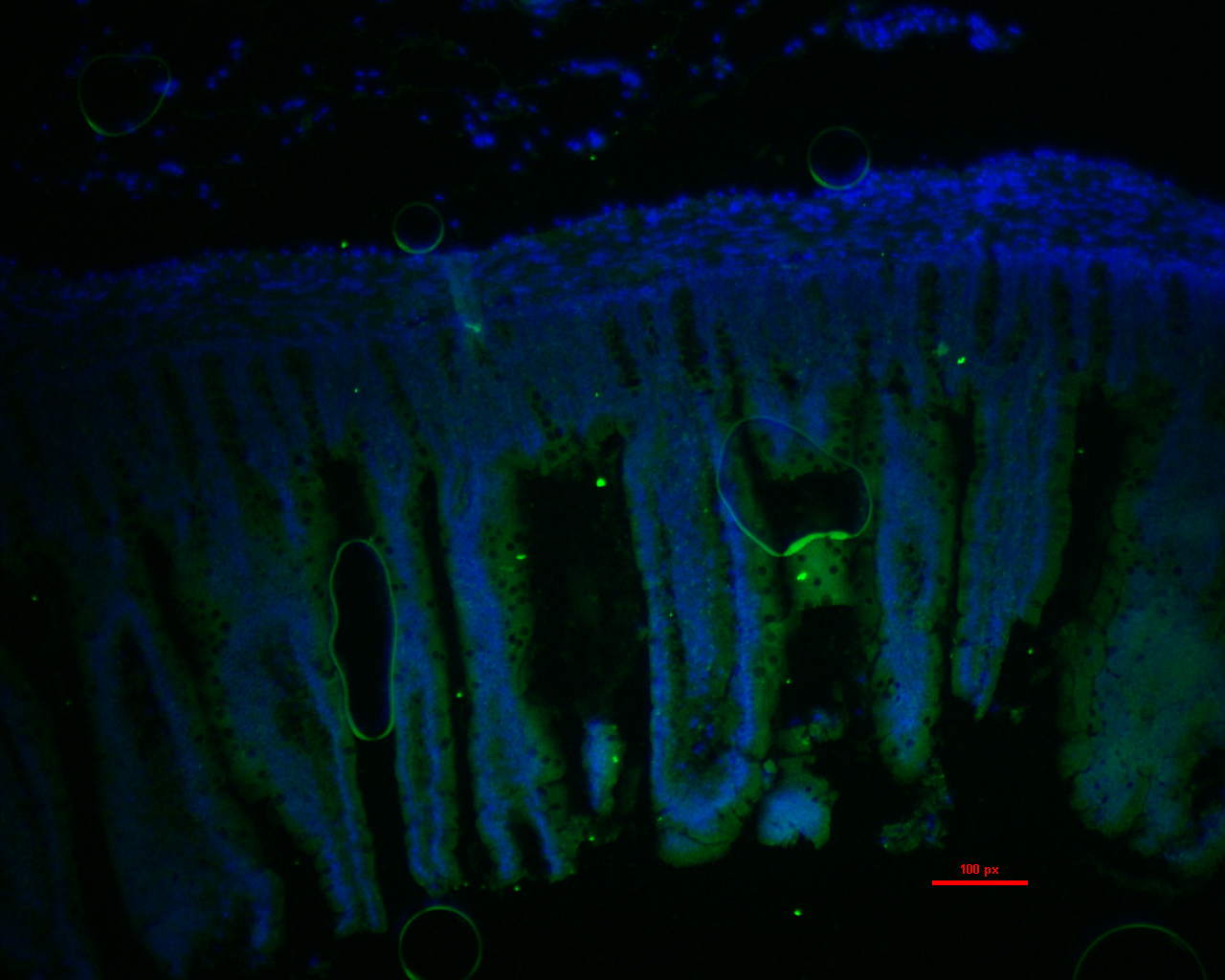

Supplement: Supplemental Information 12 [file peerj-12-16692-s012.zip › original data-figure 6-2/6E/3.ASWVY+miR-138 mim/1.NLRP3.tif]

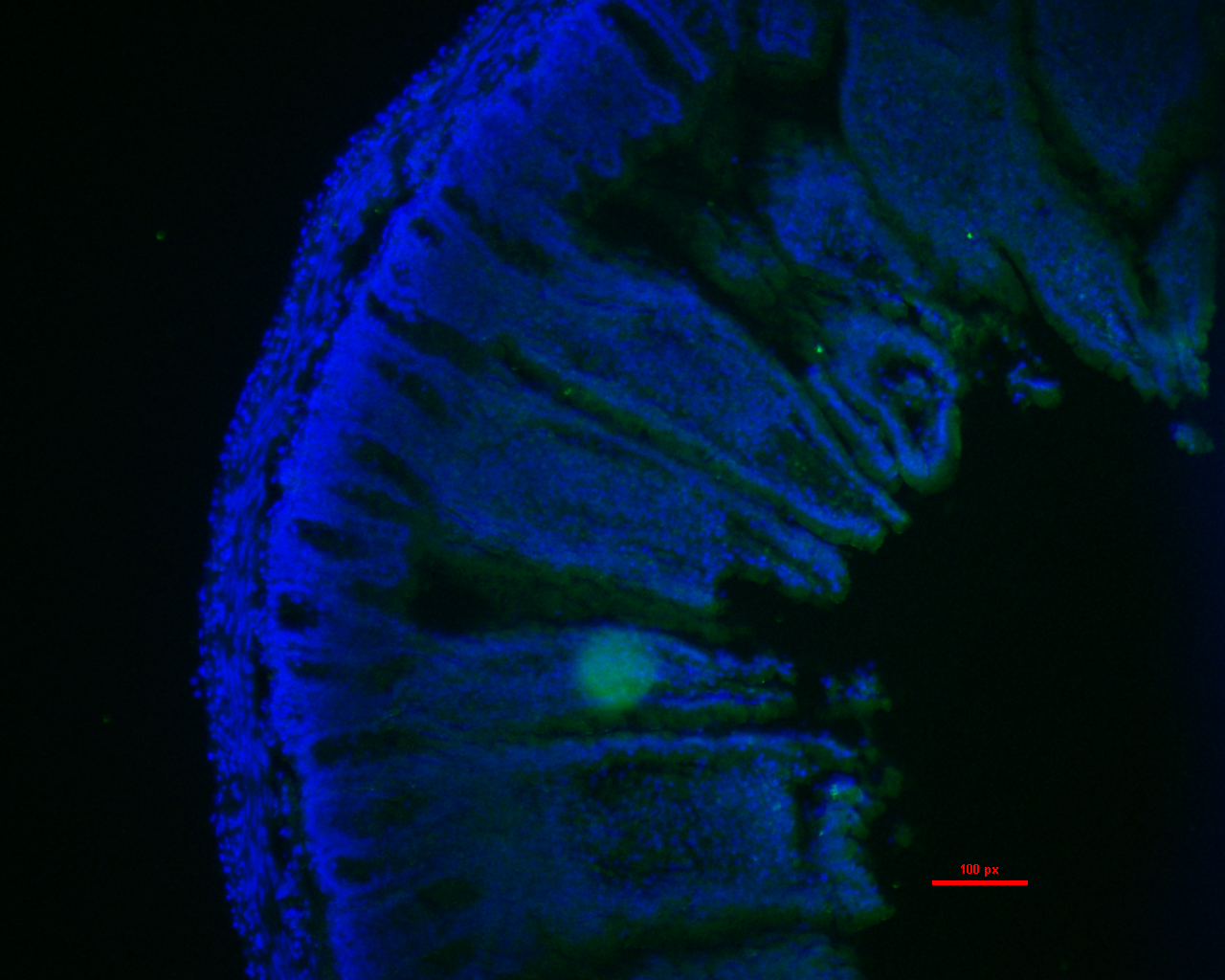

Supplement: Supplemental Information 12 [file peerj-12-16692-s012.zip › original data-figure 6-2/6E/3.ASWVY+miR-138 mim/2.HMGB1.tif]

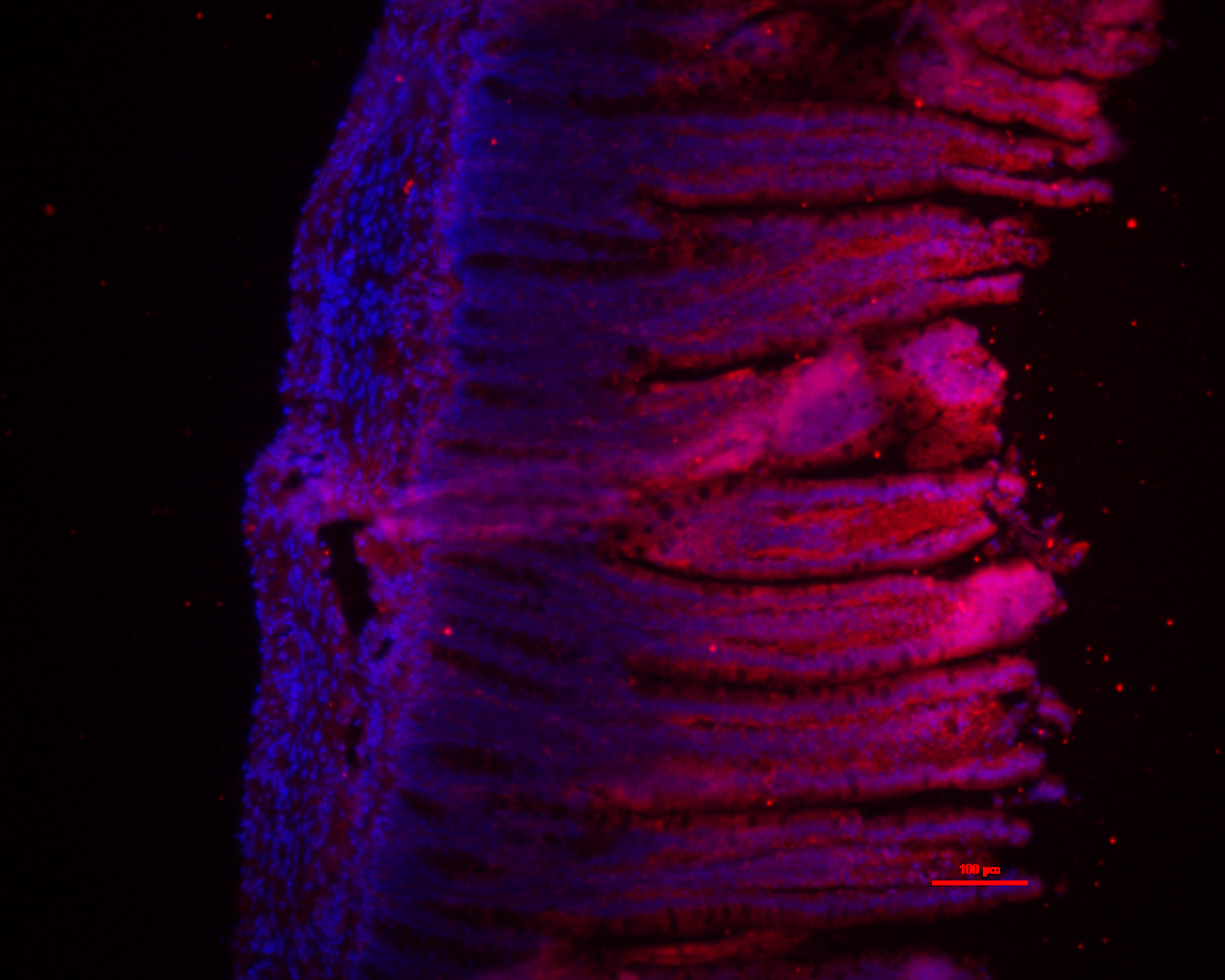

Supplement: Supplemental Information 12 [file peerj-12-16692-s012.zip › original data-figure 6-2/6E/3.ASWVY+miR-138 mim/3.ZO-1.tif]

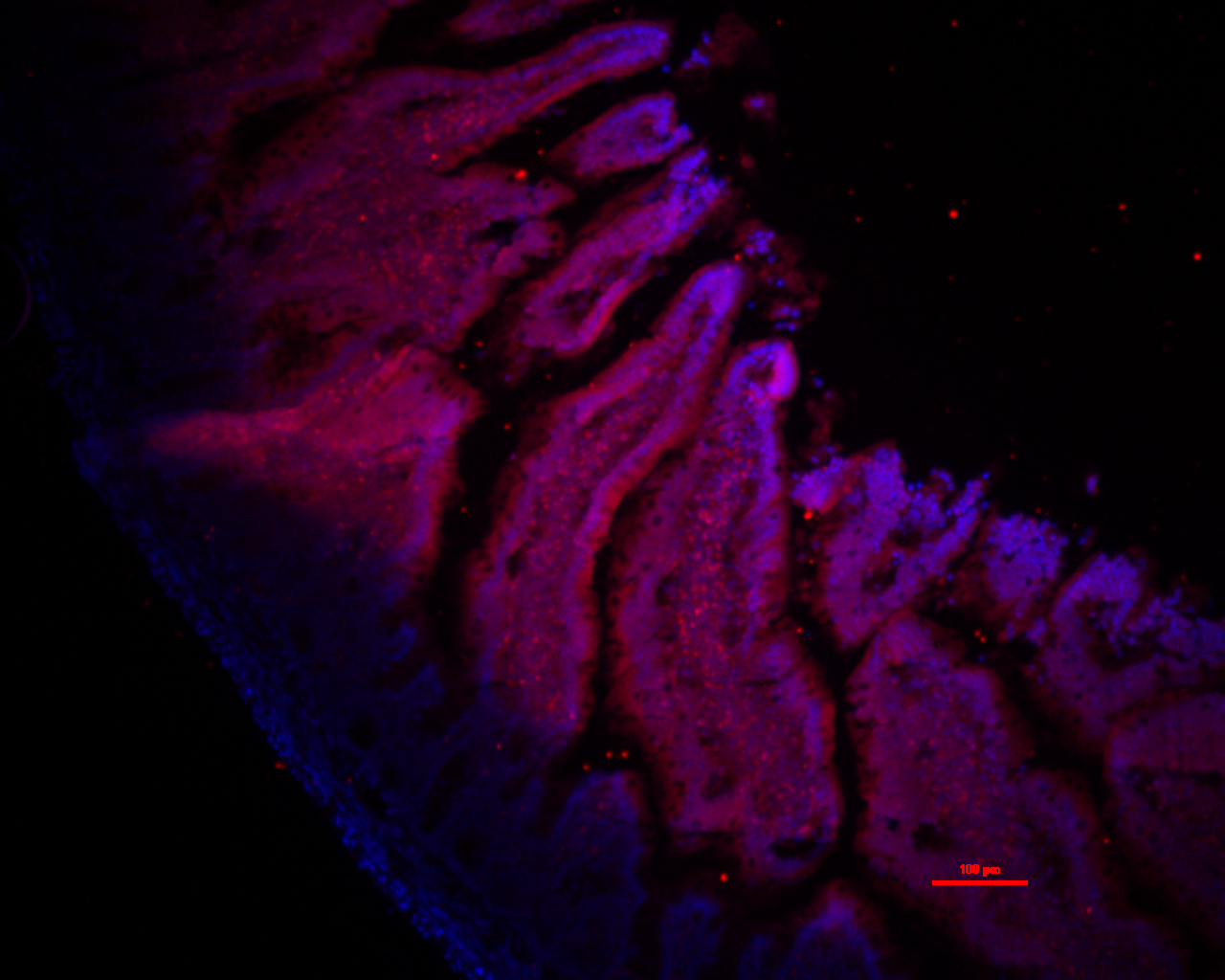

Supplement: Supplemental Information 12 [file peerj-12-16692-s012.zip › original data-figure 6-2/6E/3.ASWVY+miR-138 mim/4.Occludin.tif]

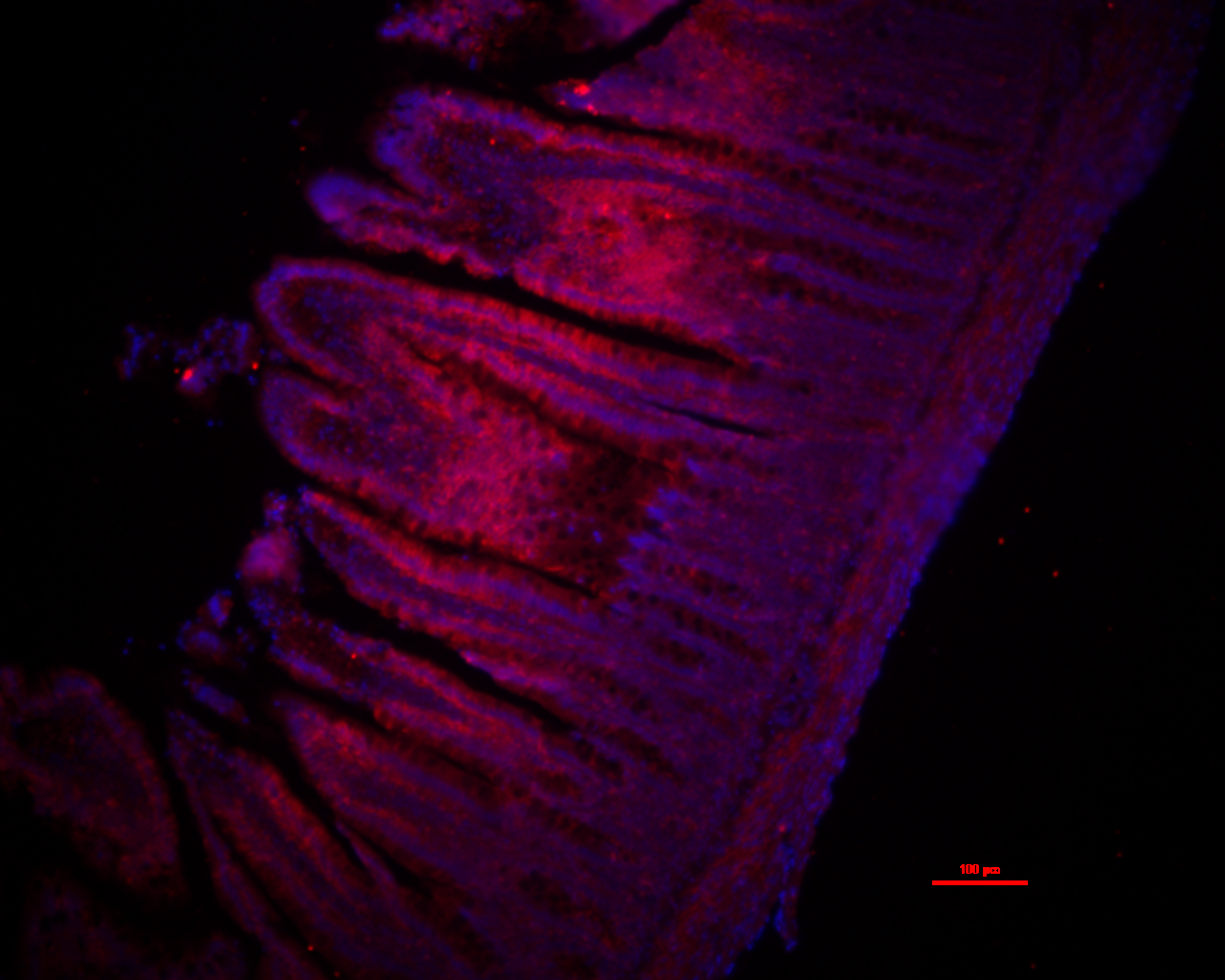

Supplement: Supplemental Information 12 [file peerj-12-16692-s012.zip › original data-figure 6-2/6E/3.ASWVY+miR-138 mim/5.Claudin-1.tif]
