# Supplementary material for: miR-138-5p ameliorates intestinal barrier disruption caused by acute superior mesenteric vein thrombosis injury by inhibiting the NLRP3/HMGB1 axis
Source: PeerJ. 2024 Feb 21;12:e16692. doi: 10.7717/peerj.16692 (PMC10893868; doi:10.7717/peerj.16692)
Supplement: Supplemental Information 11 [file peerj-12-16692-s011.zip › original data-figure 6-1/6A/Results_Report_2023-08-18-95449.pdf]

Plate Results Report

A22-4_Copy.eds

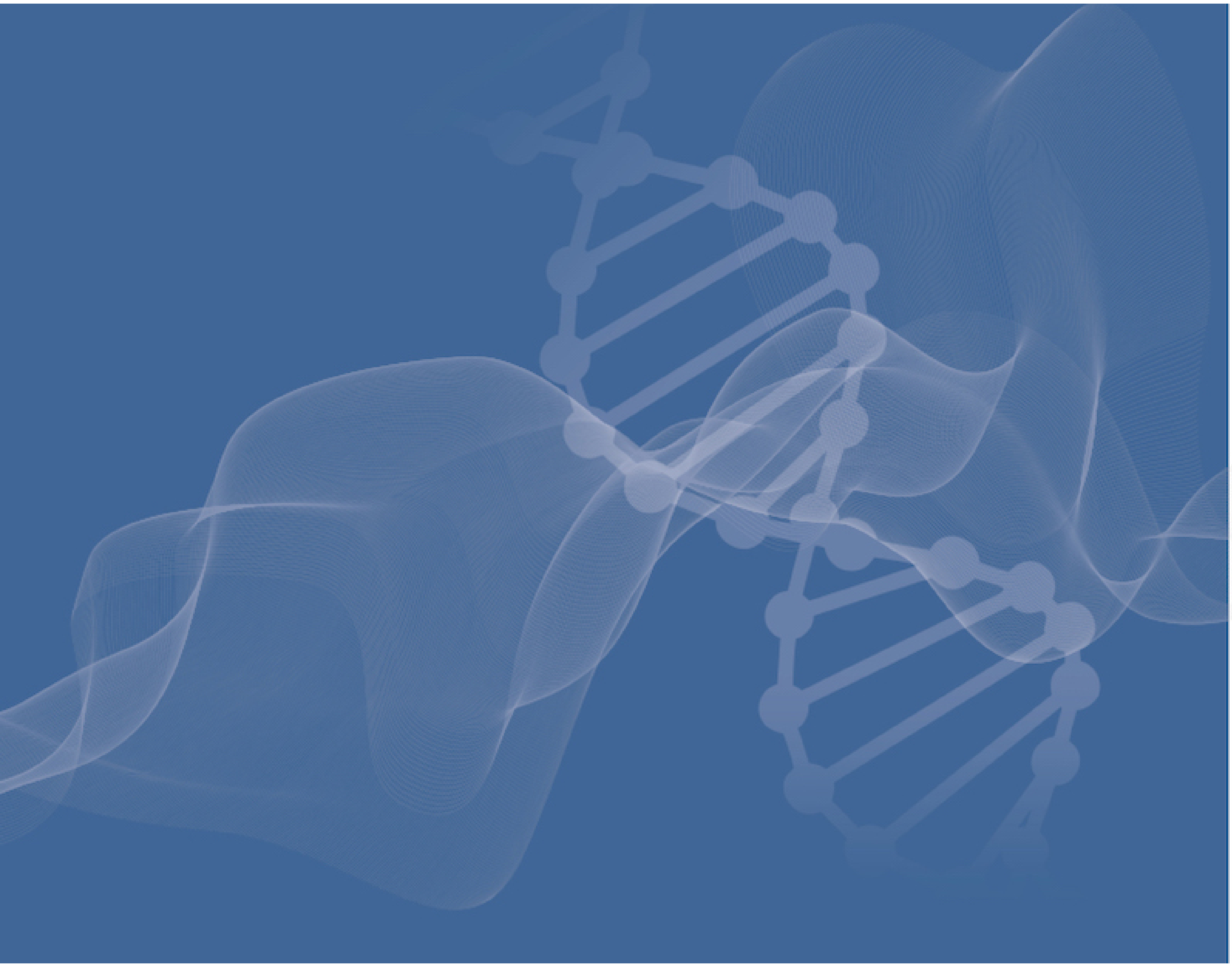

Summary

Property	Details
Bar Code	-
File Name	A22-4_Copy.eds
Run Start Date/Time	Dec 1, 2021 9:11:47 AM
Run End Date/Time	Dec 1, 2021 10:11:34 AM
Run Duration	59 minutes, and 47 seconds
Operator	DEFAULT
Instrument Name	SVT004
Instrument Type	QuantStudio™ 3 System
Instrument Serial Number	SVT004
Block Type	96-Well 0.2-mL
Block Serial Number	41145627
Heated Cover Serial Number	N/A
PCR Stage/Step Number	Stage 2, Step 2
Melt Stage Number	Stage 3
Quantification Cycle Method	Baseline Threshold
Comment	-
Software Name and Version	Design & Analysis Software v2.6.0
Plugin Name and Version	Primary Analysis v1.7.0, Relative Quantification v1.5.0
Analysis Date/Time	Jul 19, 2023 9:19:14 AM

Well Table

Well	Sample	Target	Task	Cq	Cq Confidence	Amp Score	Amp Status	Cq Threshold	Baseline Start/End	Melt Temp
C7	ASMVT	GAPDH	Unknown	17.735	0.986	1.838	AMP	2.123	3-10	82.653
C8	ASMVT	GAPDH	Unknown	18.043	0.963	1.839	AMP	2.123	3-12	82.504
C9	ASMVT	GAPDH	Unknown	17.485	0.986	1.835	AMP	2.123	3-10	82.653
C10	ASMVT	miR-138-5p	Unknown	27.369	0.991	1.859	AMP	1.96	3-18	82.355
C11	ASMVT	miR-138-5p	Unknown	27.355	0.987	1.85	AMP	1.96	3-17	82.058
C12	ASMVT	miR-138-5p	Unknown	27.29	0.99	1.85	AMP	1.96	3-17	82.207
D7	ASMVT	GAPDH	Unknown	18.031	0.983	1.849	AMP	2.123	3-10	82.504
D8	ASMVT	GAPDH	Unknown	17.526	0.989	1.844	AMP	2.123	3-10	82.653
D9	ASMVT	GAPDH	Unknown	17.8	0.968	1.839	AMP	2.123	3-9	82.504
D10	ASMVT	miR-138-5p	Unknown	27.215	0.988	1.865	AMP	1.96	3-18	82.355
D11	ASMVT	miR-138-5p	Unknown	27.315	0.987	1.848	AMP	1.96	3-19	82.058
D12	ASMVT	miR-138-5p	Unknown	27.607	0.939	1.848	AMP	1.96	3-20	81.909
E7	ASMVT	GAPDH	Unknown	18.191	0.985	1.845	AMP	2.123	3-10	82.504
E8	ASMVT	GAPDH	Unknown	17.824	0.991	1.838	AMP	2.123	3-10	82.653
E9	ASMVT	GAPDH	Unknown	18.136	0.987	1.835	AMP	2.123	3-10	82.504
E10	ASMVT	miR-138-5p	Unknown	27.553	0.988	1.851	AMP	1.96	3-19	82.206
E11	ASMVT	miR-138-5p	Unknown	27.507	0.98	1.834	AMP	1.96	3-17	81.759
E12	ASMVT	miR-138-5p	Unknown	27.361	0.988	1.855	AMP	1.96	3-19	81.909
F7	ASMVT+miR-138 mim	GAPDH	Unknown	18.247	0.987	1.852	AMP	2.123	3-9	82.355
F8	ASMVT+miR-138 mim	GAPDH	Unknown	18.413	0.99	1.841	AMP	2.123	3-12	82.504
F9	ASMVT+miR-138 mim	GAPDH	Unknown	18.156	0.988	1.837	AMP	2.123	3-9	82.504
F10	ASMVT+miR-138 mim	miR-138-5p	Unknown	24.494	0.99	1.852	AMP	1.96	3-17	82.355
F11	ASMVT+miR-138 mim	miR-138-5p	Unknown	24.427	0.989	1.858	AMP	1.96	3-15	81.909
F12	ASMVT+miR-138 mim	miR-138-5p	Unknown	24.499	0.99	1.849	AMP	1.96	3-17	82.058
G7	ASMVT+miR-138 mim	GAPDH	Unknown	18.167	0.985	1.837	AMP	2.123	3-9	82.504
G8	ASMVT+miR-138 mim	GAPDH	Unknown	18.091	0.986	1.846	AMP	2.123	3-11	82.504
G9	ASMVT+miR-138 mim	GAPDH	Unknown	17.811	0.992	1.848	AMP	2.123	3-10	82.504
G10	ASMVT+miR-138 mim	miR-138-5p	Unknown	24.615	0.992	1.861	AMP	1.96	3-17	82.355
G11	ASMVT+miR-138 mim	miR-138-5p	Unknown	24.767	0.98	1.835	AMP	1.96	3-17	81.759

Well	Sample	Target	Task	Cq	Cq Confi dence	Amp Score	Amp Status	Cq Thres hold	Baseline Start/End	Melt Temp
G12	ASMVT+miR-138 mim	miR-138-5p	Unknown	24.611	0.988	1.854	AMP	1.96	3-17	81.909
H7	ASMVT+miR-138 mim	GAPDH	Unknown	18.221	0.985	1.849	AMP	2.123	3-10	82.504
H8	ASMVT+miR-138 mim	GAPDH	Unknown	18.019	0.985	1.848	AMP	2.123	3-10	82.504
H9	ASMVT+miR-138 mim	GAPDH	Unknown	18.112	0.98	1.836	AMP	2.123	3-11	82.504
H10	ASMVT+miR-138 mim	miR-138-5p	Unknown	24.845	0.992	1.843	AMP	1.96	3-17	82.355
H11	ASMVT+miR-138 mim	miR-138-5p	Unknown	24.678	0.988	1.852	AMP	1.96	3-17	81.909
H12	ASMVT+miR-138 mim	miR-138-5p	Unknown	24.858	0.992	1.832	AMP	1.96	3-16	81.909

Replicate Group Table

Sample	Target	No. of Replicates	Cq Mean	Cq SD
ASMVT	GAPDH	9	17.864	0.255
ASMVT	miR-138-5p	9	27.397	0.13
ASMVT+miR-138 mim	GAPDH	9	18.137	0.166
ASMVT+miR-138 mim	miR-138-5p	9	24.644	0.156

Plate Layout

	1	2	3	4	5	6	7	8	9	10	11	12
A												
B												
C							ASMVT<br>GAPDH (17.735)	ASMVT<br>GAPDH (18.043)	ASMVT<br>GAPDH (17.485)	ASMVT<br>miR-138-5p (27.369)	ASMVT<br>miR-138-5p (27.355)	ASMVT<br>miR-138-5p (27.29)
D							ASMVT<br>GAPDH (18.031)	ASMVT<br>GAPDH (17.526)	ASMVT<br>GAPDH (17.8)	ASMVT<br>miR-138-5p (27.215)	ASMVT<br>miR-138-5p (27.315)	ASMVT<br>miR-138-5p (27.607)
E							ASMVT<br>GAPDH (18.191)	ASMVT<br>GAPDH (17.824)	ASMVT<br>GAPDH (18.136)	ASMVT<br>miR-138-5p (27.553)	ASMVT<br>miR-138-5p (27.507)	ASMVT<br>miR-138-5p (27.361)
F							ASMVT+miR-13...<br>GAPDH (18.247)	ASMVT+miR-13...<br>GAPDH (18.413)	ASMVT+miR-13...<br>GAPDH (18.156)	ASMVT+miR-13...<br>miR-138-5p (24.494)	ASMVT+miR-13...<br>miR-138-5p (24.427)	ASMVT+miR-13...<br>miR-138-5p (24.499)
G							ASMVT+miR-13...<br>GAPDH (18.167)	ASMVT+miR-13...<br>GAPDH (18.091)	ASMVT+miR-13...<br>GAPDH (17.811)	ASMVT+miR-13...<br>miR-138-5p (24.615)	ASMVT+miR-13...<br>miR-138-5p (24.767)	ASMVT+miR-13...<br>miR-138-5p (24.611)
H							ASMVT+miR-13...<br>GAPDH (18.221)	ASMVT+miR-13...<br>GAPDH (18.019)	ASMVT+miR-13...<br>GAPDH (18.112)	ASMVT+miR-13...<br>miR-138-5p (24.845)	ASMVT+miR-13...<br>miR-138-5p (24.678)	ASMVT+miR-13...<br>miR-138-5p (24.858)

Amplification Plot (dRn)

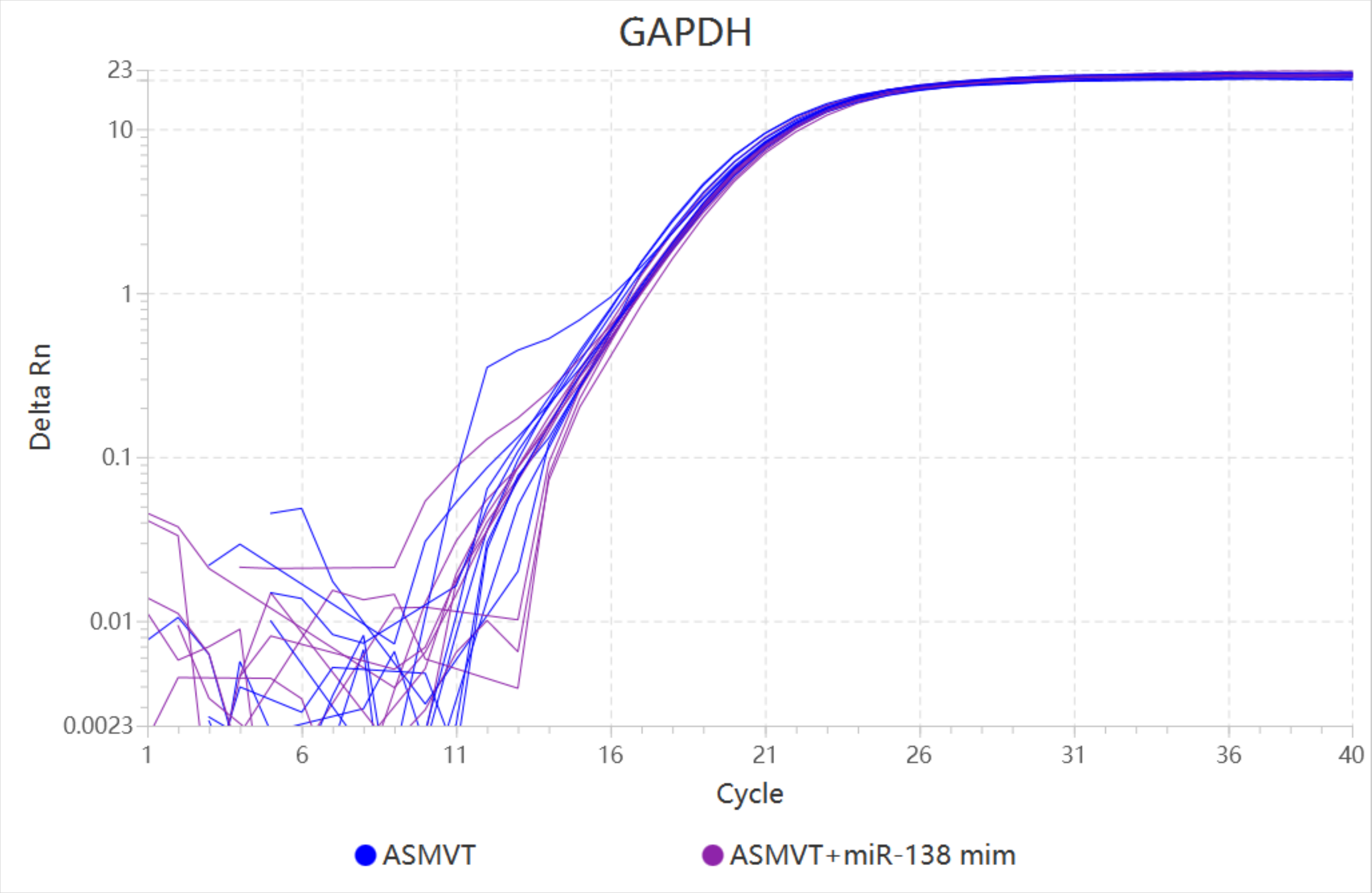

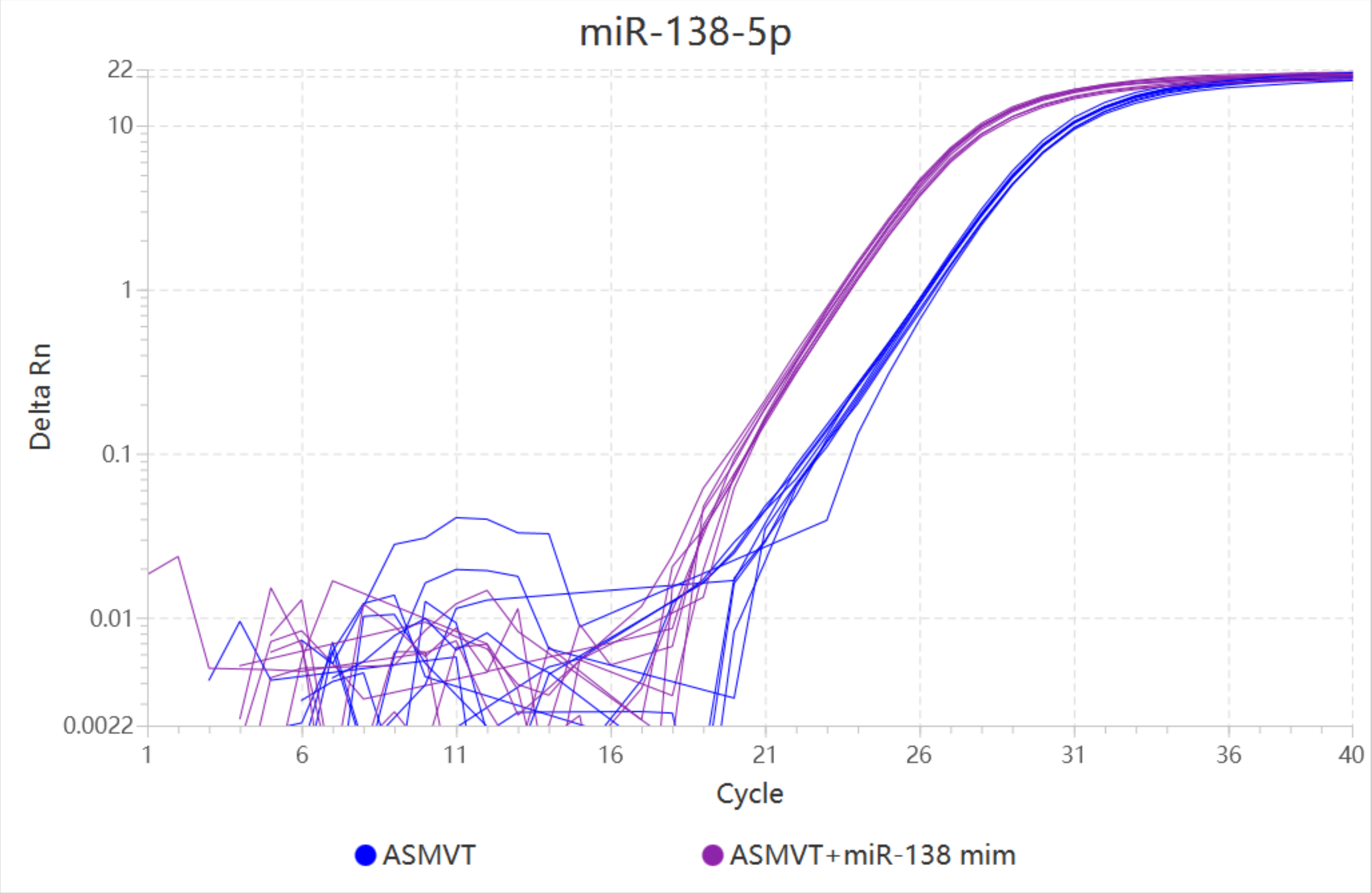

Amplification Plot (Rn)

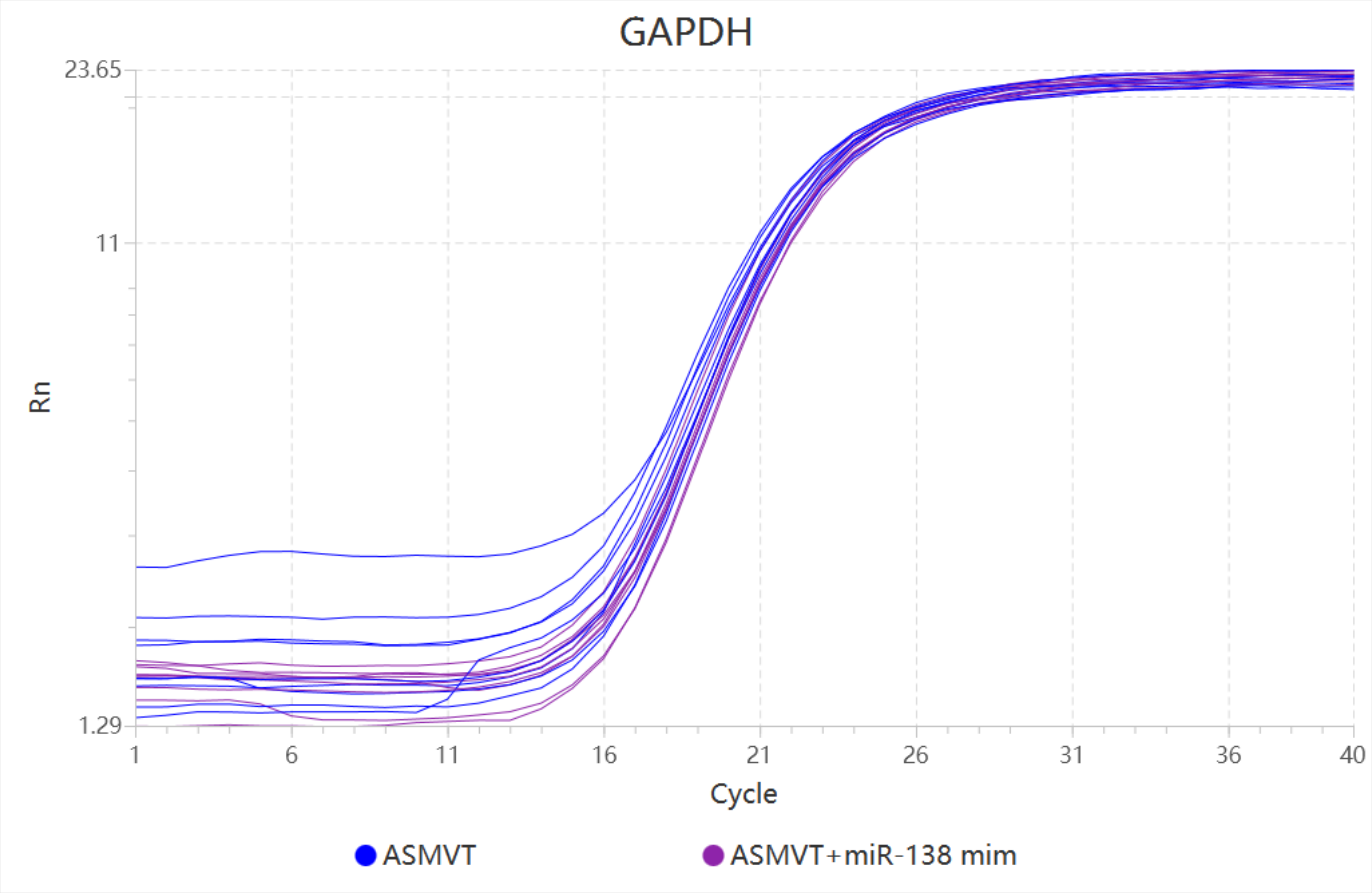

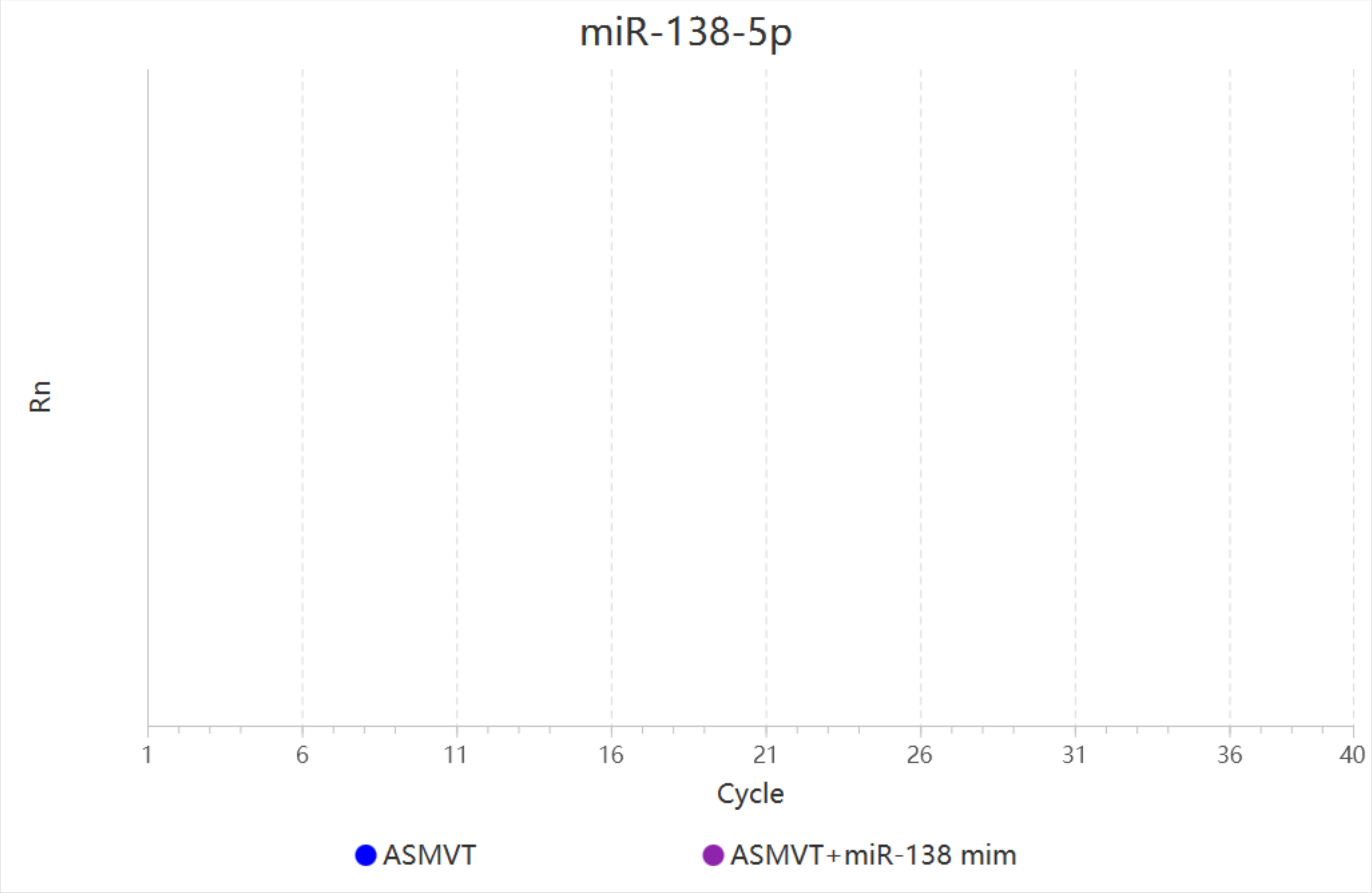

Melt Curve Plot

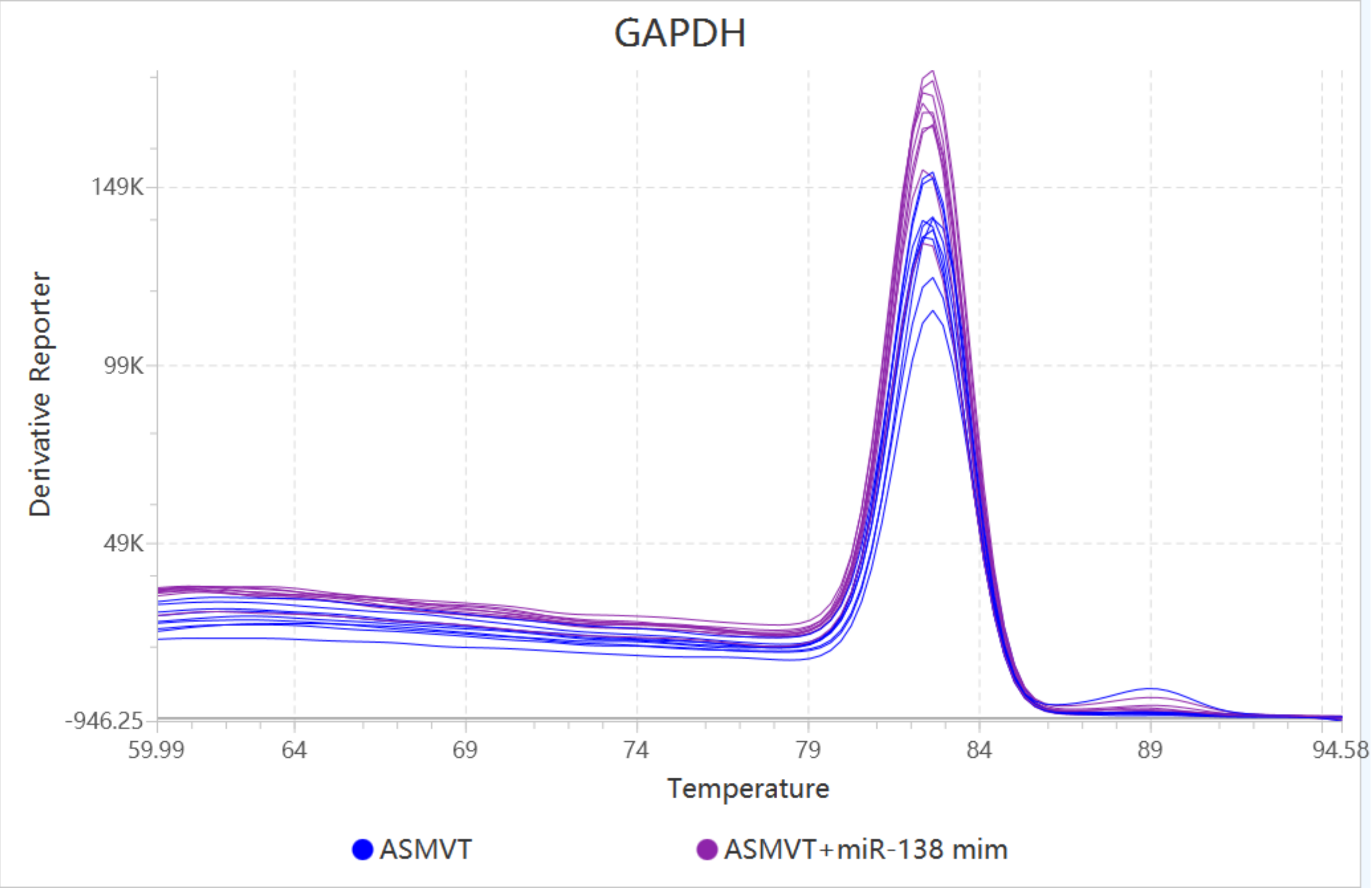

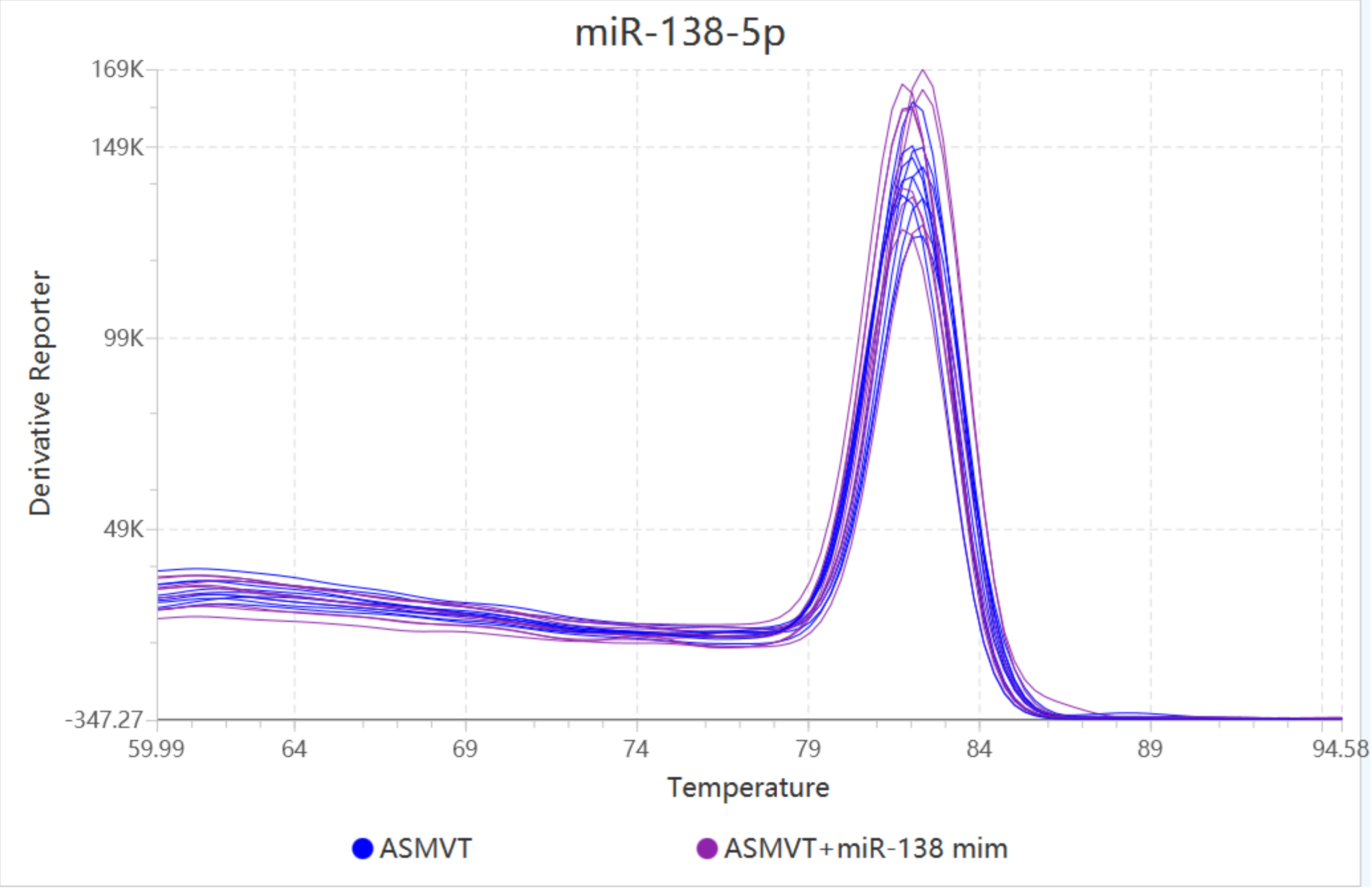

Run Method

Block Type	96-Well 0.2-mL Block
Sample Volume	20.0
Cover Temperature	105.0
Run mode	FAST

Stage	Collection Flag	Ramp Rate	Temperature	Hold Time	Starting Cycle	Auto Delta Temperature	Auto Delta Hold Time
Hold Stage	false	2.74°C/sec	95.0°C	20	-	-	-
PCR Stage (40 cycles)	false	2.74°C/sec	95.0°C	1	-	-	-
	true	2.12°C/sec	60.0°C	20	-	-	-
Melt Stage	false	2.74°C/sec	95.0°C	1	-	-	-
	false	2.12°C/sec	60.0°C	20	-	-	-
	true	0.15°C/sec	95.0°C	1	-	-	-

Primary Analysis Settings

General

PCR Stage/Step Stage 2, Step 2

Quantification Cycle Method Baseline Threshold

Target	Auto Threshold	Threshold	Auto Baseline	Baseline Start	Baseline End
DEFAULT	Yes	AUTO	Yes	AUTO	AUTO

Melt

Melt Stage/Step Stage 3, Step 3

Target	Multi Peak	Threshold Type	Peak Level (%)	Peak Height
DEFAULT	Yes	Percentage	10	-
GAPDH	Yes	Percentage	10	-

QC Alerts

Curve Quality Alert Enabled No

Results Quality Alert Enabled Yes

Advanced

Set the Delta-Rn below which curves will be considered Non-Amplified No

Primary Analysis Variant N/A

Relative Quantification Settings

General

RQ Min/Max Calculations	Confidence Level (95.0)
Max Allowed EqCq Mean	40
Include Adjusted EqCq Mean	No
Analysis Type	Singleplex

Endo Controls

Normalization Type	Specific endogenous control

Target	Endogenous Control	Auto	Efficiency(%)
GAPDH	Yes	Yes	AUTO

References

Reference Sample	ASMVT

Relative Quantification Results (Sample)

Sample	Target	EqCq Mean	Adjusted EqCq Mean	ΔEqCq Mean	ΔEqCq SD	ΔEqCq SE	ΔΔEqCq	RQ	RQ Min	RQ Max
ASMVT	GAPDH	17.864	17.864	-	-	-	-	-	-	-
ASMVT	miR-138-5p	27.397	27.397	9.533	0.287	0.096	-	1	0.869	1.151
ASMVT+miR-138 mim	GAPDH	18.137	18.137	-	-	-	-	-	-	-
ASMVT+miR-138 mim	miR-138-5p	24.644	24.644	6.506	0.228	0.076	-3.027	8.15	7.29	9.112

Relative Quantification Plot

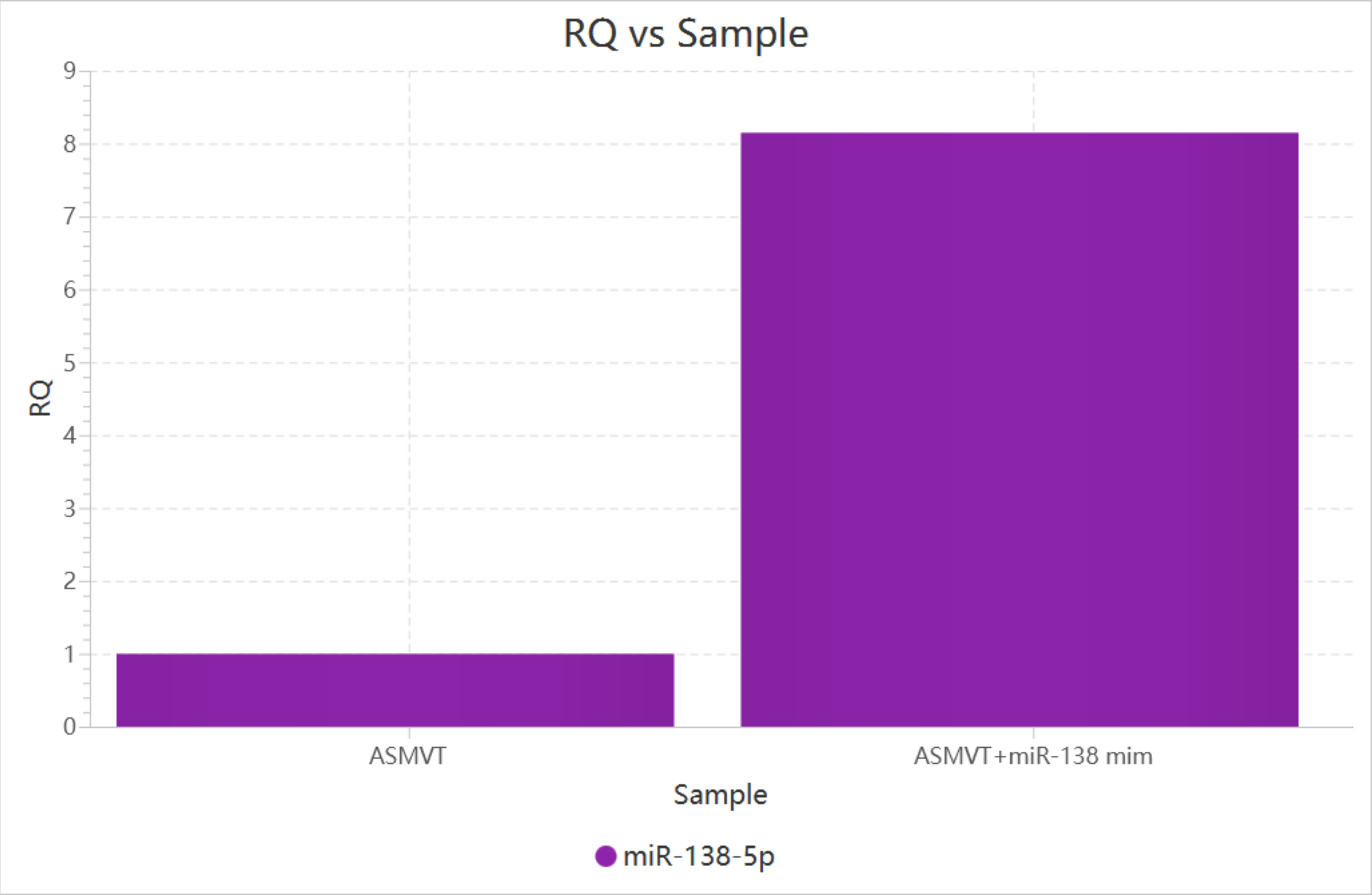

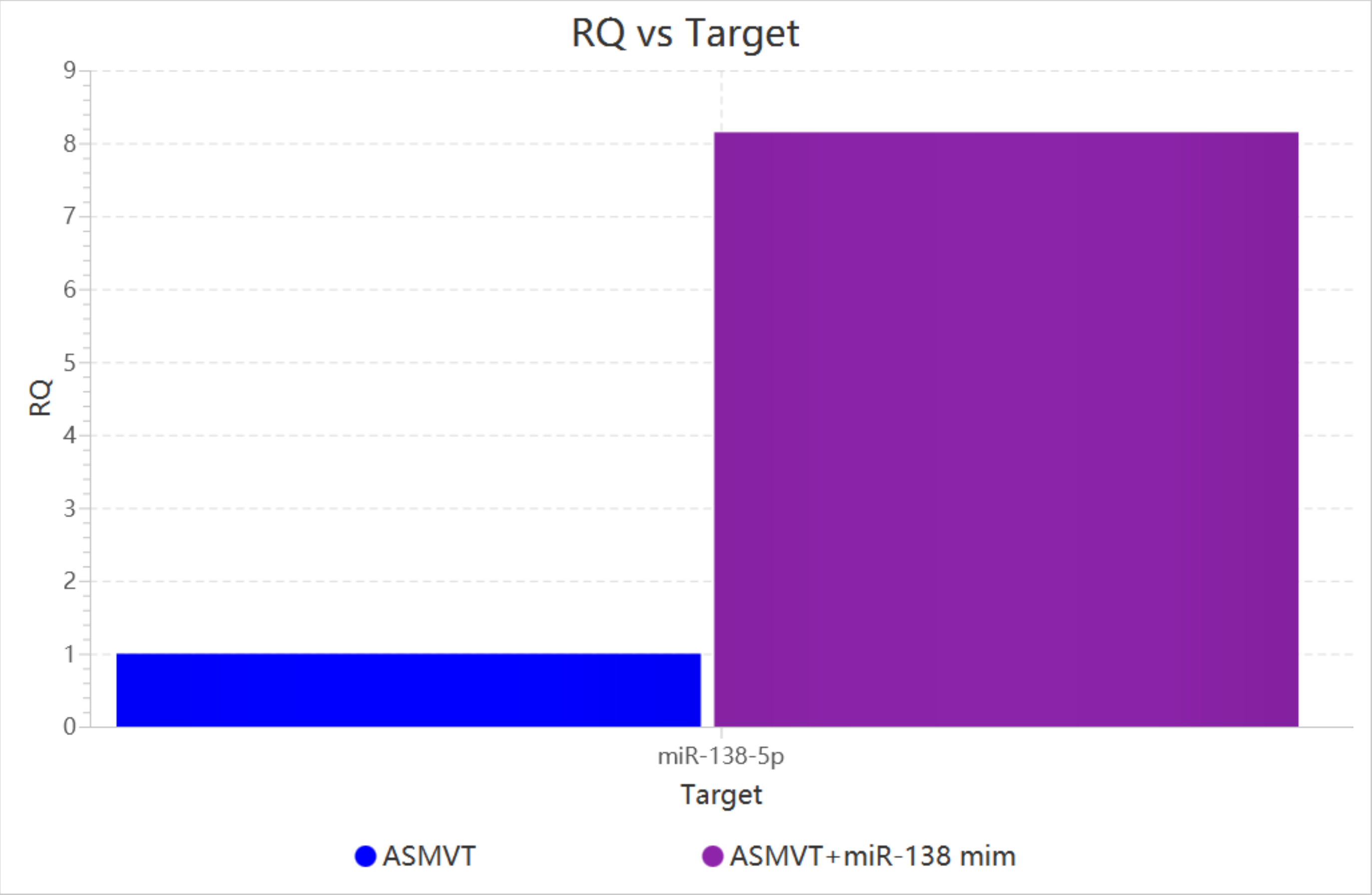

- End of Report -
